# A Phase 1 Study of ^131^I-CLR1404 in Patients with Relapsed or Refractory Advanced Solid Tumors: Dosimetry, Biodistribution, Pharmacokinetics, and Safety

**DOI:** 10.1371/journal.pone.0111652

**Published:** 2014-11-17

**Authors:** Joseph J. Grudzinski, Benjamin Titz, Kevin Kozak, William Clarke, Ernest Allen, LisaAnn Trembath, Michael Stabin, John Marshall, Steve Y. Cho, Terence Z. Wong, Joanne Mortimer, Jamey P. Weichert

**Affiliations:** 1 Cellectar Biosciences, Inc., Madison, WI, United States of America; 2 Department of Medical Physics, University of Wisconsin School of Medicine and Public Health, Madison, WI, United States of America; 3 Department of Radiology, University of Wisconsin, Madison, WI, United States of America; 4 Department of Radiology and Radiological Sciences, Vanderbilt University, Nashville, TN, United States of America; 5 Department of Medicine and Lombardi Comprehensive Cancer Center, Medstar Georgetown University Hospital, Washington, DC, United States of America; 6 Department of Radiology, Johns Hopkins Hospital, Baltimore, MD, United States of America; 7 Department of Radiology, Duke University Medical Center, Durham, NC, United States of America; 8 Department of Medical Oncology and Therapeutics Research, City of Hope, Duarte, CA, United States of America; Glaxo Smith Kline, Denmark

## Abstract

**Introduction:**

^131^I-CLR1404 is a small molecule that combines a tumor-targeting moiety with a therapeutic radioisotope. The primary aim of this phase 1 study was to determine the administered radioactivity expected to deliver 400 mSv to the bone marrow. The secondary aims were to determine the pharmacokinetic (PK) and safety profiles of ^131^I-CLR1404.

**Methods:**

Eight subjects with refractory or relapsed advanced solid tumors were treated with a single injection of 370 MBq of ^131^I-CLR1404. Whole body planar nuclear medicine scans were performed at 15–35 minutes, 4–6, 18–24, 48, 72, 144 hours, and 14 days post injection. Optional single photon emission computed tomography imaging was performed on two patients 6 days post injection. Clinical laboratory parameters were evaluated in blood and urine. Plasma PK was evaluated on ^127^I-CLR1404 mass measurements. To evaluate renal clearance of ^131^I-CLR1404, urine was collected for 14 days post injection. Absorbed dose estimates for target organs were determined using the RADAR method with OLINDA/EXM software.

**Results:**

Single administrations of 370 MBq of ^131^I-CLR1404 were well tolerated by all subjects. No severe adverse events were reported and no adverse event was dose-limiting. Plasma ^127^I-CLR1404 concentrations declined in a bi-exponential manner with a mean t_½_ value of 822 hours. Mean Cmax and AUC(0-t) values were 72.2 ng/mL and 15753 ng•hr/mL, respectively. An administered activity of approximately 740 MBq is predicted to deliver 400 mSv to marrow.

**Conclusions:**

Preliminary data suggest that ^131^I-CLR1404 is well tolerated and may have unique potential as an anti-cancer agent.

**Trial Registration:**

ClinicalTrials.gov NCT00925275

## Introduction

In 2013, it was estimated that there were over 1.6 million new cases of cancer and 580,000 cancer-related deaths in the United States [1]. Worldwide, cancer of all types accounts for 3.5 million deaths annually. Treatment of neoplastic diseases represents a critical, unsatisfied medical need worldwide [2].

Tumor treatment with radioactive isotopes has been used as a fundamental cancer therapy for decades. Selective delivery of effective doses of radioactive isotopes that ablate tumor tissue and spare surrounding normal tissue remains a goal of targeted radiotherapies. CLR1404 is a novel radioiodinated therapeutic that takes advantage of the unique chemistry of alkylphosphocholine analogs (APCs) and their analogs that may achieve this goal [3].

Radioiodinated (^124^I, ^125^I, ^131^I) CLR1404 has been evaluated in both xenograft and transgenic tumor models in mice and rats. Overall, these studies demonstrated specific uptake and retention in over 50 malignant tumor models [3], [4], [5], [6]. In addition, ^131^I-CLR1404 potently inhibited tumor growth in nine murine tumor models (breast, prostate, lung, glioma, ovarian, renal, colorectal, pancreatic and melanoma) confirming it as a potential anti-cancer agent [6]. These promising preclinical results provided motivation for clinical translation of ^131^I-CLR1404.

The primary endpoints of this study were to perform organ and total body dosimetry calculations and to determine the amount of radioactivity (megabecquerel (MBq)) expected to deliver 350-400 mSv to bone marrow – the initial administered radioactivity in subsequent maximum tolerated dose (MTD) phase I studies. The secondary endpoints of the study were to determine the pharmacokinetic (PK) and safety profiles of ^131^I-CLR1404.

## Materials and Methods

The protocol for this trial and supporting TREND checklist are available as supporting information; see Protocol S1 and Checklist S1, respectively.

### Ethical Conduct of the Study

This study was conducted in accordance with International Conference on Harmonisation (ICH) guidance for good clinical practice (GCP) and all applicable regulatory requirements and ethical principles, including the Declaration of Helsinki. Written informed consent from each subject was obtained at screening, prior to the performance of any study-specific procedures.

This multi-center study (Georgetown University, Johns Hopkins University, Duke University, and City of Hope) evaluating dosimetry and safety (clinicaltrials.gov number, NCT00925275) was approved by each institution's Institutional Review Board (IRB).

### Study Population

The study population consisted of eight patients (6 male and 2 female) with refractory or relapsed advance solid tumors who failed standard therapy or for whom no standard therapy existed. Primary tumors and metastatic sites for each patient are listed in Table 1. Subjects had a median age of 59 years (range of 46–71 years) and a median weight of 83.95 kg (range of 61.1–143.7 kg).

**Table 1:** Subject Information.

Subject	Center	Age	Gender	Race or Ethnicity	Primary Solid tumor	ECOG Status Screening	ECOG Status Day 42	Metastatic Sites	Prior Chemotherpy	Prior Hormonal, Immunological, Biological and Other Therapy
101	Georgetown	71	Female	White	Colon	0	1	Ovary	5-FU	bevacizumab
								Abdominal wall	leucovorin	
								Umbilicus	oxaliplatin	
102	Georgetown	46	Male	Black	Colon	1*	0	Lymph nodes	5-FU	bevacizumab
								Kidney	leucovorin	cetuximab
								Liver	oxaliplatin	
								Lung	irinotecan	
									capecitabine	
103	Georgetown	58	Male	White	Colon	0	0	Lymph nodes	5-FU	bevacizumab
									leucovorin	cetuximab
									irinotecan	
									capecitabine	
201	Johns Hopkins	69	Male	White	Esophageal	1	1	Lymph nodes	paclitaxel	
								Liver	cisplatin	
								Lung	irinotecan	
										
301	Duke	54	Male	White	Colon	1	1	Lung	5-FU	CEA(6D) VRP vaccine
									leucovorin	interferon alpha 2B
									oxaliplatin	bevacizumab
									irinotecan	
									capecitabine	
302	Duke	66	Male	White	Prostate	1	1	Bone	docetaxel	leuprolide
										bicalutamide
401	City of Hope	60	Female	Latino	Left Breast	0	0	Spleen		anastrazole
										fulvestrant
402	City of Hope	52	Male	White	Prostate	0	0	Lung		leuprolide(Ongoing)
								Bone		bicalutamide

Details about the subjects enrolled in the study.

^*^ECOG status is for Day 0.

### Study Design

**Figure 1:**
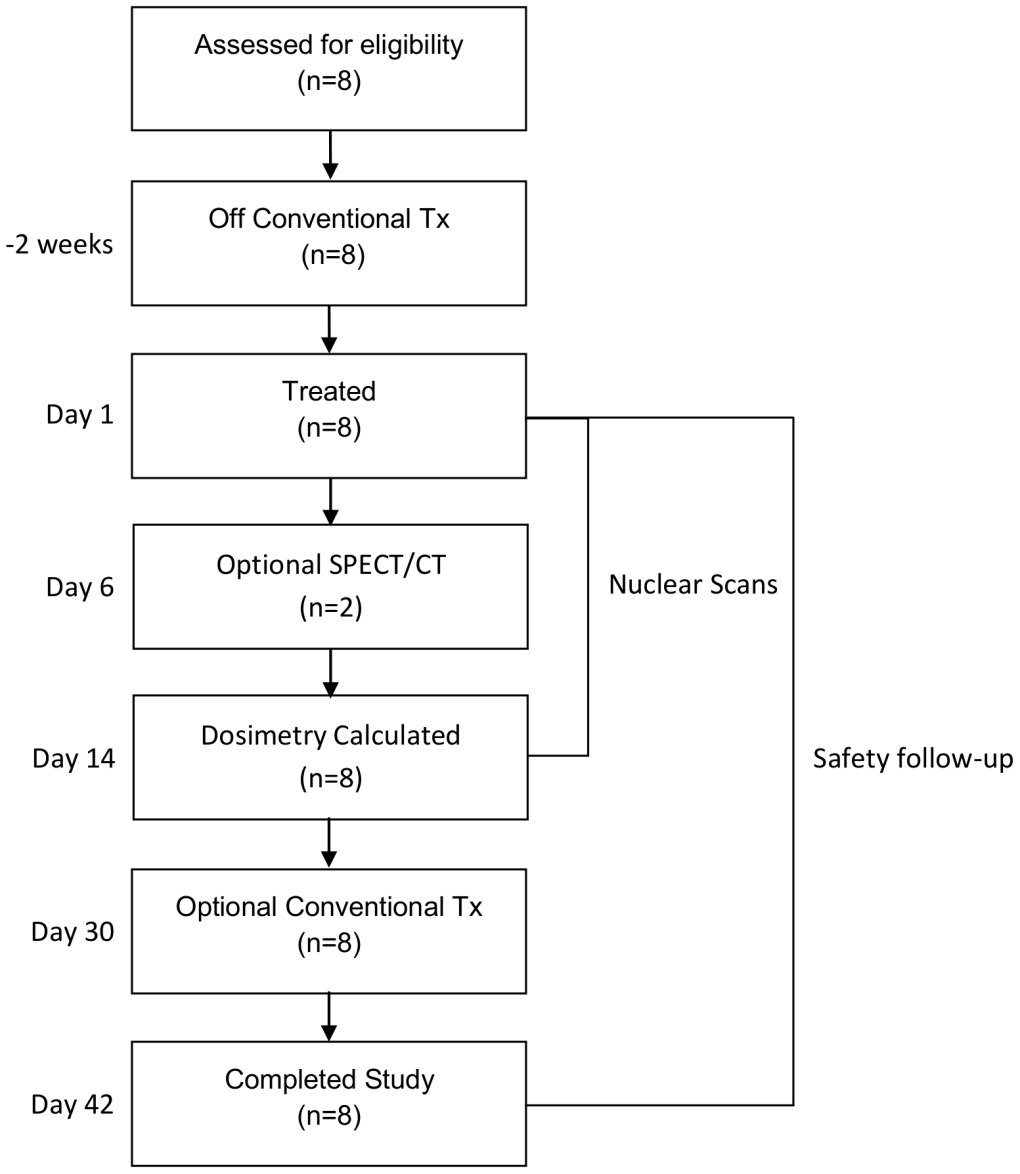
Consort diagram and clinical outcome of enrolled patients. Patients were taken off of conventional therapies from two weeks prior to and 30 days after treatment with ^131^I-CLR1404. After treatment with ^131^I-CLR1404, patients underwent nuclear medicine procedures for 14 days and were followed for safety for 42 days.

Subjects were infused with 370 MBq of ^131^I-CLR1404. All subjects received thyroid protection medication 24 hours prior to injection of the study drug and for 14 days afterwards. Whole body imaging for dosimetry calculations was performed at 15–35 minutes post infusion; 4–6, 18–24, 48, 72, and 144 hours post infusion and on days 6 and 14 post infusion. At the Investigator's discretion, optional single photon emission computed tomography (SPECT) imaging was obtained between day 3 and 14 to further characterize tumor uptake. Vital signs were monitored pre-infusion, 5, 15, 30 and 60 minutes as well as 4–6 hours post-infusion and at all study visits. ECGs were performed at screening, pre-infusion, 5 and 60 minutes post infusion, as well as 4–6 hours, 18–24 hours, day 6 and day 14 post infusion. Blood and urine were collected for clinical laboratory evaluation pre-infusion and on days 6, 14, 30 and 42. Blood was drawn for evaluation of lipids pre-infusion and at 72 hours post infusion. Blood was collected for plasma PK analysis prior to infusion, and at 5, 15, 30, and 60 minutes; 4–6, 18–24, 48, and 72 hours; 6, 14, 30, and 42 days post infusion. To evaluate the radiological clearance of ^131^I-CLR1404 from the renal system, subjects collected all of their urine for 14 days post infusion. This data was also used for radiation dosimetry calculations. Subjects were monitored at each visit for adverse events (AEs) and for the use of concomitant medications. The study design is presented in Figure 1 and the protocol deviations are listed in Table S1.

After all subjects completed the protocol, calculations were performed using Organ Level INternal Dose Assessment (OLINDA)-generated biodistribution curves to predict the administered ^131^I-CLR1404 in MBq that would deliver a radiation absorbed dose of 350–400 mSv to total bone marrow [7]. This dose, as calculated with the OLINDA/EXM software, determined the starting dose for subsequent Phase 1 maximum tolerated dose (MTD) protocols. 350–400 mSv was chosen because it is one-fifth of the assumed tolerance dose of the bone marrow [7].

After reviewing the dosimetry and safety results of the first 8 subjects enrolled, a decision was made to terminate enrollment since analysis of the dosimetry data demonstrated minimal variance from subject to subject with regard to total body and organ dosimetry, specifically the bone marrow.

### Treatments

#### ^131^I-CLR1404 Administration

^131^I-CLR1404 (t_1/2,phys_  = 192 hours) was supplied as a ready-for-use radiopharmaceutical for intravenous dosing consisting of ^131^I-CLR1404 (18-(p-[131I]iodophenyl)octadecyl phosphocholine) and ^127^I-CLR1404 (18-(paraiodophenyl) octadecyl phosphocholine) in sodium chloride injection USP, ethanol USP/NF, polysorbate 20 NF, and sodium ascorbate USP. The study drug was packaged in a single-use glass vial and was passed through a 25 mm, 0.22 or 0.45 micron sterile filter prior to administration. The appropriate volume of study drug sufficient to give 370 MBq was diluted to 10 mL with normal saline and thoroughly mixed prior to administration via slow IV infusion.

Each subject received a single administration of 370 MBq ^131^I-CLR1404 (0.26–0.43 mg mass dose) through a freely-running peripheral intravenous catheter over a period of at least 10 minutes. The selection of 370 MBq was based on organ and total body radiation dose estimates for ^131^I-CLR1404 determined from dosimetry studies performed in mice. These studies indicated that 370 MBq of ^131^I-CLR1404 provided an acceptable range of total body dosimetry, approximately 0.72 mSv/MBq.

To protect the thyroid from radioactive iodine uptake, thyroid protection was required. Thyroid protective agents were initiated at least 24 hours prior to, the day of, and for 14 days after administration of ^131^I-CLR1404. Thyroid protective medications included either a saturated solution of potassium iodide (SSKI) 4 drops orally, Lugol's solution 20 drops orally three times daily, or potassium iodide tablets 130 mg orally once daily. The choice of thyroprotection was left to the discretion of individual study sites.

### Dosimetry and Biodistribution

#### Assessment of radiological clearance

Study subjects were asked to collect all of their urine for the first 14 days of the study. The urine collection began at the first void after the 15–35 minute post-infusion scan. Urine was collected in the following incremental time points: 0–24 hours, 25–48 hours, 49–72 hours, 73–96 hours, Days 4–6, Days 6–10, and Days 10–14 post infusion.

#### Whole Body Imaging

Planar whole body nuclear medicine scans were obtained of each subject, from the anterior and posterior projection, at the following times post injection: 15–35 minutes, 4–6 hours, 18–24 hours, 48±6 hours, 72±6 hours, 144±6 hours, and day 14±1. These images were acquired with a high energy parallel hole collimator, usingon a triple energy window (364 keV ±10%, 298 keV ±7.5%, 436 keV ±7.5%) and a minimum matrix size of 256×1024, for a speed of no faster than 10 cm/min. With the exception of the scan acquired 15 minutes post injection, each subject was asked to void their bladder prior to each imaging session. Using the MedDisplay software program, regionsRegions of interest (ROIs) were then drawn manually over identified geometrical structures in the body, including the liver, spleen, lungs, kidneys and the whole body. Data from the whole body were used to establish a time-activity pattern for activity not accounted for in the primary organs of uptake, with the net difference attributed to renal and/or gastrointestinal excretion. Body thickness in the various regions was estimated by evaluating attenuation as observed in Co-57 flood source scans, using attenuation coefficients determined at each participating site in experiments with ^57^Co and ^131^I sources [8].

Data in all regions were expressed as a percent of the initial activity in the total body from the early (e.g. 15 minute) whole body ROI. Data for subjects were fit to one or more exponential retention functions using the SAAM II software [9]. Time integrals of activity were calculated from the SAAM II results and then expressed as normalized numbers of disintegrations (residence times) in the source organs [10]. Time integrals for urinary bladder were calculated using the data provided for activity excreted in urine and assuming that the bladder was emptied regularly every 4.8 hours. It is unknown whether radioactivity measured within the urine originated from ^131^I-CLR1404, metabolites, or free ^131^I. Organ time-activity integrals were entered into the OLINDA/EXM software [11], using the adult male model. Integrals for major organs were calculated directly from the image data; values for remainder of body were calculated by inference (i.e. “whole body” minus the sum of all other organs).

#### Dosimetry calculation

Biodistribution data were analysed to produce time activity curves for each organ of interest that were integrated to calculate organ-specific residence times. These residence times were used in OLINDA/EXM to generate organ specific radiation absorbed doses from a 370 MBq injection of ^131^I-CLR1404. This data, along with total urine collection for up to 14 days, was used to extrapolate and predict organ specific and total body radiation absorbed doses from projected therapeutic injections of ^131^I-CLR1404.

#### Optional SPECT/CT

The protocol also permitted the acquisition of single-photon emission tomography (SPECT) or SPECT with computed tomography (CT) attenuation (SPECT/CT) for the qualitative imaging of tumor uptake of ^131^I-CLR1404 at the investigator's discretion in subjects with tumors amenable to imaging.

Two patients, 301 and 402, had tumors that were amenable to SPECT imaging. Their respective anatomical regions of interest, chest and pelvis, were imaged on the GE Infinia SPECT/CT camera using a high energy general purpose parallel-hole collimator with counts from the 15% energy window at 364 KeV. For patient 301, a total image of 120 projections was acquired over 360° with an acquisition time of 1 s/frame and an angular step of 3°. For patient 402, a total image of 60 projections was acquired over 360° with an acquisition time of 1 s/frame and angular step of 6°. After Each SPECT acquisition, both patients underwent a low-dose helical CT scan. The non-contrast CT, with acquisition parameters of 140 KeV and 5 mAs, was used for attenuation correction and anatomical conformation. The SPECT images from both patients were reconstructed with a conventional iterative algorithm, ordered subset expectation maximization (OSEM). A workstation providing multiplanar resampled images was used for image display and analysis (Amira; MA, U.S.A.).

### Pharmacokinetics

#### Pharmacokinetic variables

Pharmacokinetic assessment of the plasma concentration of ^127^I-CLR1404 was performed using a validated HPLC method with MS/MS detection (LLOQ 2.00 ng/mL) [12]. Plasma ^127^I-CLR1404 concentration-time data were calculated using non-compartmental methods. The following parameters were defined: maximum plasma concentration (C_max_), area under the concentration curve from 0 to 144 hours [AUC_(0–144hr)_], area under the concentration curve from time 0 to time t (t is the time of last quantifiable concentration; t = 1008 hr) [AUC_(0–1008hr)_], plasma half-life (t_½_), apparent terminal phase rate constant (λz), clearance (CL), volume of distribution (Vd), and volume of distribution at steady-state (Vss).

#### Sampling procedure

For ^127^I-CLR1404 mass measurements, blood was drawn pre-treatment and at 5, 15, 30, 60 minutes, 4–6, 18–24, 48, and 72 hours, 6, 14, 30 and 42 days post-infusion. Samples were frozen and stored until the radioactivity decayed to background levels (approximately 80 days) and then forwarded to Covance Laboratories (Madison, WI) for analysis. CLR1404 has previously been shown to be chemically stable under the storage conditions employed.

#### Analysis

Pharmacokinetic analysis of ^127^I-CLR1404 mass measurements was performed by Covance Laboratories (Madison, WI), using WinNonlin Professional Edition (Pharsight Corporation, Version 5.2). A nominal dose level of 0.3 mg and actual sampling times were used for PK analysis. For PK calculation purposes, all times were normalized to the start of infusion.

### Safety Assessments

Blood was drawn and urine was collected for evaluation of clinical laboratory parameters pre-infusion and on days 6, 14, 30 and 42. Baseline signs and symptoms were recorded. All adverse events (AEs) were recorded at each visit for the duration of the study. The severity of each AE was graded using the National Cancer Institute Common Toxicity Criteria for Adverse Events (NCI CTCAE) version 3.0.

## Results

### Dosimetry and Biodistribution

#### Assessment of bodily clearance

**Figure 2:**
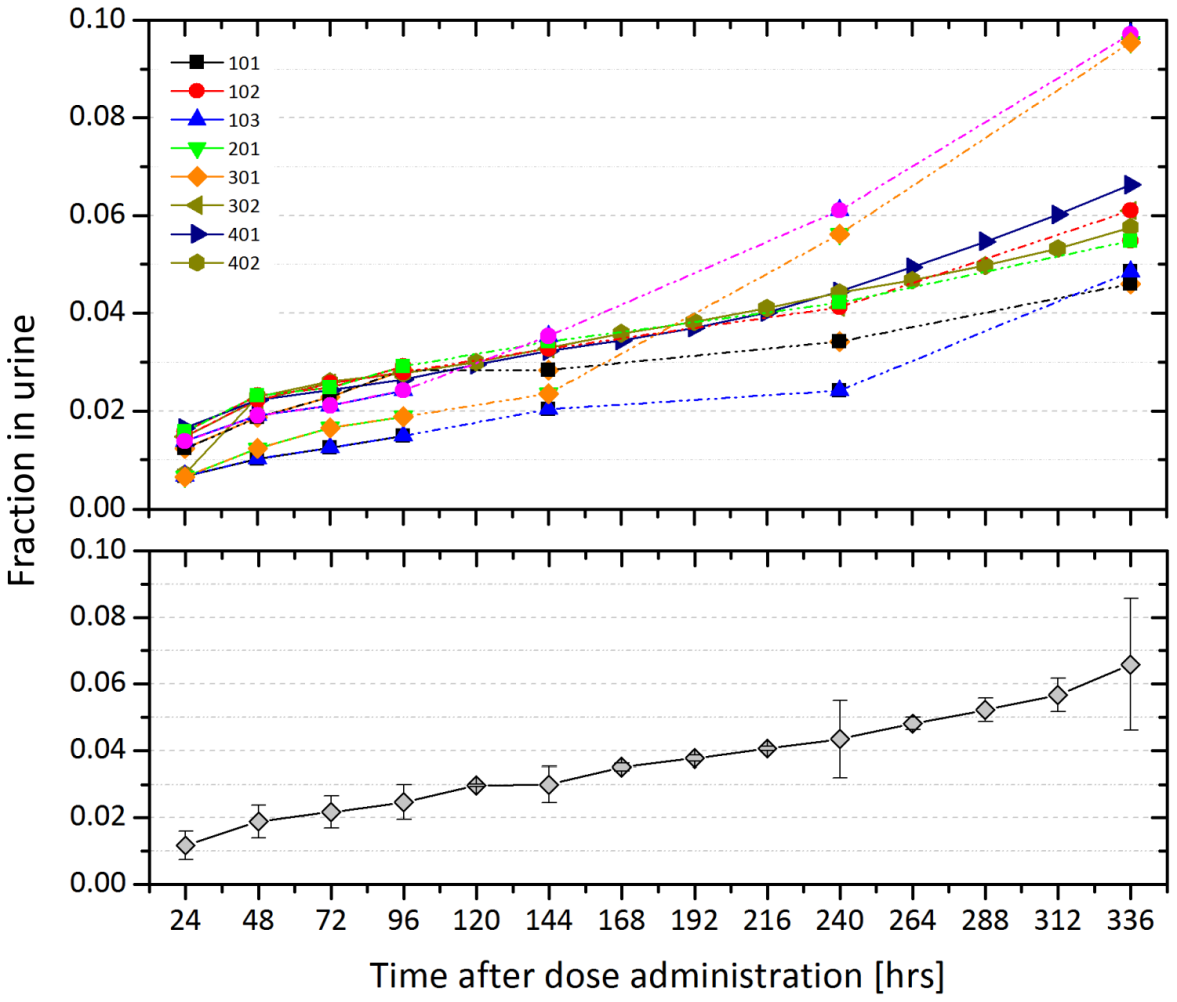
Renal clearance of ^131^I-CLR1404. The cumulative fraction of ^131^I-CLR1404 within the urine is shown following a single dose injection of 370 MBq of ^131^I-CLR1404. The top graph shows each subject individually while the bottom graph shows the average of the group with the standard deviation represented with error bars. These data are used as input into OLINDA/EXM for dosimetry calculations.

Figure 2 shows the fraction of ^131^I-CLR1404 in the urine over time. ^131^I-CLR1404 displayed very slow elimination. Most subjects had only about 5% elimination in the urine over the course of measurement, although two had clearances closer to 10% (Figure 2, top panel).

#### Whole Body Imaging

**Figure 3:**
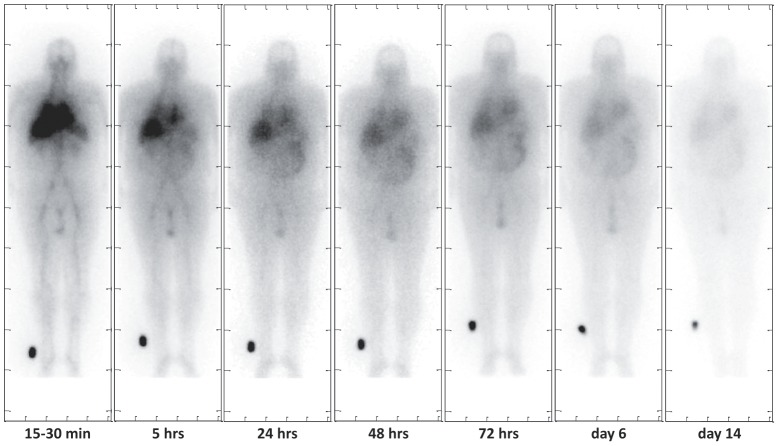
Whole body conjugate-view planar images for subject 301. Images are shown for 15–30 minutes, 4, 24, 48, 72 hours, 6 and 14 days post injection, respectively. Because the agent is metabolized in the hepatobiliary system, there is evidence of ^131^I-CLR1404 within the liver and intestines at relatively late time points.

Figure 3 shows a representative series of whole-body planar images from patient 301 who had colorectal cancer with a lung metastasis. Qualitatively, most of the activity is cleared from the heart and liver within the first 24 hours after injection but is visible past 6 days in the extremities. After 14 days, there is very little activity left in the body.

#### Dosimetry calculation

Table 2 shows the dosimetry results for the eight patients who received 370 MBq of ^131^I-CLR1404. The average red marrow dose for the eight subjects is 0.56 mSv/MBq (2.09 rem/mCi). Based on this, approximately 740 MBq (20 mCi) is predicted to deliver 400 mSv (40 rem) to marrow. Organs involved in metabolism of ^131^I-CLR1404, namely the liver, kidneys, and spleen, exhibited higher doses of 1.09 mSv/MBq, 1.05 mSv/MBq, and 1.60 mSv/MBq, respectively.

**Table 2:** Dosimetry Results Calculated by OLINDA/EXM.

	Average		Std Dev		COV	95% CI (lower upper)			
	mSv/MBq	rem/mCi	mSv/MBq	rem/mCi		mSv/MBq		rem/mCi	
Adrenals	7.41E-01	2.74E+00	4.00E-02	1.47E-01	5.40%	7.13E-01	7.69E-01	2.64E+00	2.84E+00
Brain	5.86E-01	2.17E+00	3.38E-02	1.26E-01	5.80%	5.63E-01	6.09E-01	2.08E+00	2.26E+00
Breasts	5.61E-01	2.08E+00	2.93E-02	1.07E-01	5.20%	5.41E-01	5.81E-01	2.01E+00	2.15E+00
Gallbladder Wall	7.74E-01	2.86E+00	3.53E-02	1.29E-01	4.60%	7.50E-01	7.98E-01	2.77E+00	2.95E+00
LLI Wall	8.88E-01	3.28E+00	2.35E-01	8.64E-01	26.50%	7.25E-01	1.05E+00	2.68E+00	3.88E+00
Small Intestine	7.42E-01	2.75E+00	3.60E-02	1.32E-01	4.80%	7.17E-01	7.67E-01	2.66E+00	2.84E+00
Stomach Wall	7.11E-01	2.63E+00	3.50E-02	1.31E-01	4.90%	6.87E-01	7.35E-01	2.54E+00	2.72E+00
ULI Wall	7.81E-01	2.89E+00	7.91E-02	2.94E-01	10.10%	7.26E-01	8.36E-01	2.69E+00	3.09E+00
Heart Wall	7.11E-01	2.63E+00	3.49E-02	1.29E-01	4.90%	6.87E-01	7.35E-01	2.54E+00	2.72E+00
Kidneys (all subjects)	1.05E+00	3.90E+00	9.83E-01	3.65E+00	93.30%	3.69E-01	1.73E+00	1.37E+00	6.43E+00
Kidneys (excluding 301)	7.11E-01	2.63E+00	1.81E-01	6.71E-01	25.40%	5.77E-01	8.45E-01	2.13E+00	3.13E+00
Liver	1.09E+00	4.03E+00	3.29E-01	1.22E+00	30.30%	8.62E-01	1.32E+00	3.18E+00	4.88E+00
Lungs	9.28E-01	3.43E+00	4.48E-01	1.65E+00	48.30%	6.18E-01	1.24E+00	2.29E+00	4.57E+00
Muscle	6.29E-01	2.33E+00	3.26E-02	1.21E-01	5.20%	6.06E-01	6.52E-01	2.25E+00	2.41E+00
Ovaries	7.35E-01	2.72E+00	4.08E-02	1.52E-01	5.50%	7.07E-01	7.63E-01	2.61E+00	2.83E+00
Pancreas	7.72E-01	2.86E+00	4.17E-02	1.55E-01	5.40%	7.43E-01	8.01E-01	2.75E+00	2.97E+00
Red Marrow	5.63E-01	2.09E+00	2.80E-02	1.04E-01	5.00%	5.44E-01	5.82E-01	2.02E+00	2.16E+00
Osteogenic Cells	1.29E+00	4.77E+00	7.12E-02	2.63E-01	5.50%	1.24E+00	1.34E+00	4.59E+00	4.95E+00
Skin	5.33E-01	1.97E+00	2.90E-02	1.06E-01	5.40%	5.13E-01	5.53E-01	1.90E+00	2.04E+00
Spleen	1.60E+00	5.93E+00	8.52E-01	3.14E+00	53.10%	1.01E+00	2.19E+00	3.75E+00	8.11E+00
Testes	6.20E-01	2.30E+00	3.57E-02	1.34E-01	5.70%	5.95E-01	6.45E-01	2.21E+00	2.39E+00
Thymus	6.59E-01	2.44E+00	3.47E-02	1.27E-01	5.30%	6.35E-01	6.83E-01	2.35E+00	2.53E+00
Thyroid	6.52E-01	2.41E+00	3.68E-02	1.35E-01	5.70%	6.26E-01	6.78E-01	2.32E+00	2.50E+00
Urinary Bladder Wall	7.10E-01	2.63E+00	3.61E-02	1.35E-01	5.10%	6.85E-01	7.35E-01	2.54E+00	2.72E+00
Uterus	7.35E-01	2.72E+00	4.15E-02	1.54E-01	5.70%	7.06E-01	7.64E-01	2.61E+00	2.83E+00
Total Body	6.69E-01	2.47E+00	3.21E-02	1.18E-01	4.80%	6.47E-01	6.91E-01	2.39E+00	2.55E+00
Effective Dose	7.41E-01	2.74E+00	9.74E-02	3.59E-01	13.20%	6.74E-01	8.08E-01	2.49E+00	2.99E+00

The table shows the statistical summary of the eight subjects' dosimetry calculations.

#### Optional SPECT/CT

**Figure 4:**
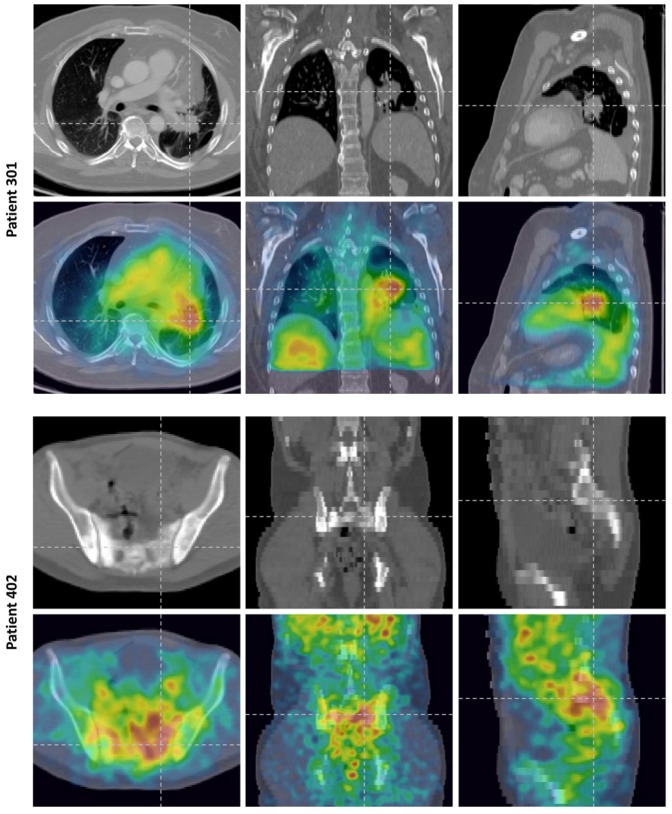
Imaging data from the optional SPECT/CT scans. Top: Day 6 SPECT/CT Images for a subject with colorectal cancer that metastasized to the lung (301). The three columns represent an axial, coronal, and sagittal slice, respectively. The top row shows the diagnostic quality CT alone while the bottom row shows the SPECT data overlayed onto the diagnostic CT. Bottom: Day 6 SPECT/CT Images for a subject with metastatic prostate cancer (402). The top row shows the low dose CT data alone while the bottom row shows the SPECT data overlayed onto the CT data. Note the increased uptake in the osteolytic lesion. The sagittal and coronal slices are of lower resolution compared to the axial plan because the CT was not of diagnostic quality; it was only used for attenuation correction of the SPECT data. A diagnostic quality CT of this subject was unavailable.

Two subjects had tumors that were amenable to SPECT/CT imaging: 301 and 402. Subject 301 had colorectal cancer that had metastasized to the left lung. The subject had undergone a partial left lower lobectomy for removal of metastatic tumor, but residual tumor remained which was surgically unresectable due to proximity to the pulmonary vein. At day 6 after the injection of 370 MBq of ^131^I-CLR1404, a SPECT/CT was performed (Figure 4). The left, center, and right panels from Figure 4 show axial, coronal, and sagittal slices of the patient data, respectively. The top row shows the CT data and the bottom row shows SPECT overlaid onto the CT data. Residual tumor adherent to the left pulmonary vein and in close apposition to the aorta can be seen in the CT scan taken from the SPECT/CT data set. It should be noted that the tumor-to-muscle ratio (TMR), where the ‘muscle’ was defined as a region-of-interest within the healthy pectoral muscle, was as high as 7.6.

Subject 402 had metastatic prostate cancer. Images taken on day 6 after the injection of 370 MBq ^131^I-CLR1404 are presented in Figure 4; accumulation of ^131^I-CLR1404 in the involved areas identified on CT - the soft tissue and bony metastases - is seen in the images. It is also worth noting the relative lack of uptake in bone marrow.

### Pharmacokinetics

After reaching C_max_, plasma concentrations of ^127^I-CLR1404 appeared to decline in a bi-exponential manner, with a mean (± SD) t½ value of 822 (±101) hours (Table 3). The start of the apparent terminal elimination phase generally occurred between 67.5 and 333 hours post administration. Mean (± SD) C_max_ and AUC(0-t) values were 72.2 (±11.5) ng/mL and 15753 (±3598) ng·hr/mL, respectively. The mean (± SD) AUC_0–144_ and λz were 3420 (±574) ng·hr/mL and 0.000855 (±0.000112) hr^−1^, respectively.

**Table 3:** Individual and Mean Pharmacokinetic Parameters following a Single Dose Infusion of 10 mCi of ^131^I-CLR1404.

Subject Number	C_max_ (ng/mL)	AUC_0–144_ (ng hr/mL)	AUC_0–1088_ (ng hr/mL)	AUC_0–∞_ (ng hr/mL)	Λz 1/hr)	t_1/2_ (hr)	CL (L/hr)	Vd (L)	Vss (L)
101	66.9	3848	22208	33565	0.000829	836	0.00894	10.8	11.0
102	54.6	2576	11059	16683	0.001	691	0.018	17.9	17.5
103	91.3	4019	16185	26452	0.00104	665	0.0113	10.9	10.8
201	73.5	3219	14255	25803	0.000791	877	0.0116	14.7	14.5
301	63.5	2599	11560	19851	0.000848	818	0.0151	17.8	17.5
302	70.1	3471	16129	29489	0.000793	874	0.0102	12.8	12.7
401	84	3916	18285	31216	0.00082	846	0.00961	11.7	11.3
402	73.8	3713	16347	30962	0.000712	974	0.00969	13.6	13.2
**Mean**	72.2	3420	15753	26753	0.000855	822	0.0118	13.8	13.6
**SD**	11.5	574	3598	5874	0.000112	101	0.0032	2.9	2.7
**Min**	54.6	2576	11059	16683	0.000712	665	0.00894	10.8	10.8
**Median**	71.8	3592	16157	27970	0.000825	841	0.0108	13.2	12.9
**Max**	91.3	4019	22208	33565	0.00104	974	0.018	17.9	17.5
**CV%**	16	17	23	22	13	12	27	21	20

Note: The extrapolated portion was greater than 30% for all subjects; therefore, results for AUC_0–∞_ and the associated parameters (CL, Vd, and Vss) were provided for informational purposes only.

**Figure 5:**
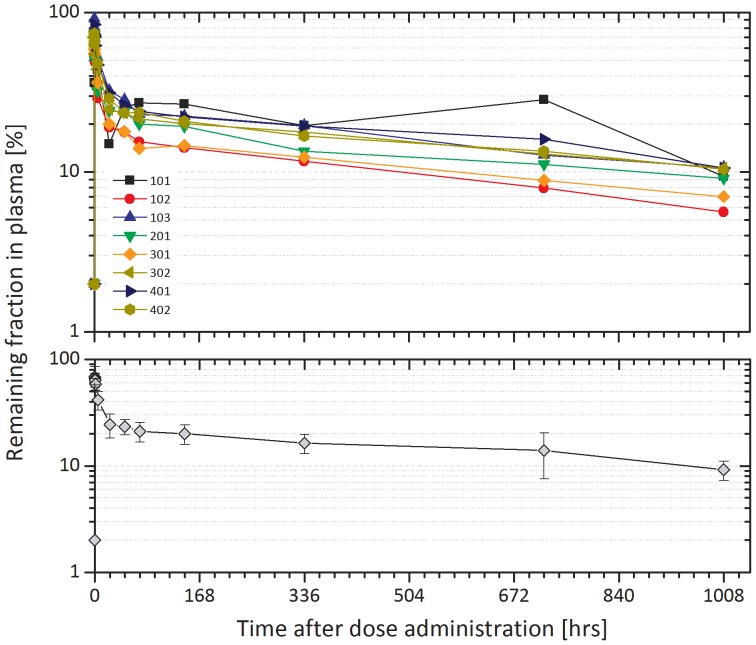
Plasma pharmacokinetics of ^127^I-CLR1404. The remaining fraction of ^127^I-CLR1404 within the plasma is plotted over time. The top graph shows each individual subject while the bottom graph shows the average of the group with the standard deviation represented with error bars.

For all subjects except 101, C_max_ of ^127^I-CLR1404 appeared at the first blood collection (5 minutes after infusion). In the case of 101, the 5 minute blood sample for plasma PK was drawn during the 10 minute infusion of the study drug, rather than 5 minutes after the end of the infusion as all of the other subjects' 5 minute samples were. The mean plasma concentration-time profile is shown in the bottom panel of Figure 5.

For individual subjects, the apparent elimination half-life ranged from 665 to 974 hours. The coefficient of variation (%CV) for C_max_ and area under the curve (AUC) values ranged from 16 to 23%, indicating that the inter-individual variation for exposure to ^127^I-CLR1404 was generally low. The extrapolated portion of the AUC_0√–∞_ was greater than 30% for all subjects; therefore, results for AUC_0–∞_ and the associated parameters (CL, Vd, and Vss) are subject to considerable uncertainty and are provided for completeness. shows the individual and mean pharmacokinetic parameters of ^127^I-CLR1404.

### Adverse Events

There were no, clinically significant changes over time in any hematology, clinical chemistry, or urinalysis parameter during the study. The adverse events (AEs) are listed in Table 4. There were no deaths or serious adverse effects AEs reported during the study. A total of 7 subjects reported 19 AEs during this study, 5 of which were considered related to study medication by the Investigator. Three subjects had treatment-related AEs of CTCAE grade 1 fatigue, while one subject each had CTCAE grade 1 constipation and CTCAE grade 1 sialadenitis. AEs were mostly CTCAE grade 1 (n = 17); two AEs were CTCAE grade 2 neither of which was considered related to ^131^I-CLR1404. Two subjects had ongoing AEs at the completion of the study. Subject 102 had ongoing CTCAE grade 1 AEs of fatigue and constipation which were considered related to study medication administration in the opinion of the Investigator. Subject 201 had ongoing diarrhea (CTCAE grade 1) and back pain (CTCAE grade 2), which were considered unrelated to study medication by the Investigator.

**Table 4:** Adverse Events (AE).

Subject number	AE No.	Preferred Term	CTCAE Grade	Frequency*	Relationship†
101	1	Fatigue	1	2	1
102	1	Fatigue	1	2	1
	2	Constipation	1	1	1
103	None				
201	1	Sialadenitis	1	1	1
	2	Diarrhoea	1	1	2
	3	Back pain	2	1	2
	4	Blood pressure decreased	1	1	2
	5	Skin ulcer	1	3	2
	6	Skin ulcer	1	3	2
	7	Nasal congestion	1	2	2
	8	Gamma-glutamyltransferase increased	2	3	2
301	1	Fatigue	1	2	2
	2	Wheezing	1	2	2
302	1	Fatigue	1	2	1
	2	Hordeolum	1	2	2
401	1	Erythema	1	3	2
402	1	Oedema peripheral	1	3	2
	2	Erythema	1	3	2
	3	Erysipelas	1	3	2

The AE experienced during the study are listed.

^*^Frequency: 1 =  Intermittent, 2 =  Continuous, 3 =  Single Episode.

† Relationship to Study Drug: 1 =  Related, 2  =  Not related.

## Discussion

The activity administered (370 MBq) was sufficient to obtain good quality images. In almost all cases, radioactivity was still clearly seen in the blood pool at the latest imaging time (14 days). ^131^I-CLR1404 had very slow and minor elimination from the body, which is indicative of a long plasma half-life, t_1/2_. Limited renal clearance was identified; generally only about 5% of the injected dose was cleared in urine. In a few cases, moderate gastrointestinal activity was seen; rough approximations were made of the uptake, and dose to the gastrointestinal organs was estimated. No thyroid uptake was visualized on any of the subject images.

There was generally high agreement with organ dose estimates between subjects. However, one subject (301) had a higher kidney uptake and radiation dose than the other subjects, so the analysis was repeated with the seven subjects that had agreement between the organ doses (Table 2). Note that renal clearance was not higher for 301 than for others (Figure 2, top panel). Based on the eight-subject calculated average red marrow dose of 0.56 mSv/MBq (2.09 rem/mCi), an administered activity of approximately 740 MBq (20 mCi) is predicted to deliver 400 mSv (40 rem) to marrow.

Since OLINDA/EXM was designed to compute ‘organ level’ dosimetry of radiopharmaceuticals using a ‘Standard Man’ phantom, it only requires low resolution planar images for input. Hence, tumors in regions of high background signal are often difficult to delineate on planar whole body images and can sometimes incorrectly be included in lung dosimetry calculations within OLINDA/EXM. To avoid this problem, high resolution tomographic imaging such as SPECT/CT should always be used to not only better delineate tumors but also compute dosimetry for radiotherapeutic agents. The relatively low number of patients in this study with lesions amenable for SPECT may have been the result of lesions that were not visible via planar imaging.

370 MBq of ^131^I-CLR1404 was well-tolerated by all subjects. There were no serious AEs or AEs that were CTCAE grade 3 or higher. Only 5 of the 19 AEs recorded in the study were considered related to study medication. All treatment related AEs were CTCAE grade 1. There were no identified changes in clinical laboratory evaluations, ECGs, vital signs, temperature, oxygen saturation, or physical examinations

The single time point non-quantitative SPECT/CT images, taken at day 6 after an administration of 370 MBq of ^131^I-CLR1404, show that there is uptake of ^131^I-CLR1404 in tumors. This uptake occurs in both visceral and bony metastases, shown in the two different malignancies. Conversely, the images also show the relative lack of uptake in adjacent normal tissue or bone marrow.

Estimating the potential tumor dosimetry of ^131^I-CLR1404 will be helpful in understanding its potential as a therapeutic. To estimate the tumor dosimetry from therapeutic administrations of ^131^I-CLR1404, further studies will include quantitative SPECT/CT imaging at multiple points in subjects in an ongoing phase 1b study.

## Conclusion

The results obtained in this Phase 1 study demonstrated that ^131^I-CLR1404 is well tolerated at a dose of 370 MBq and exhibited prolonged retention within solid tumors compared to normal tissues. An administered radioactivity of approximately 740 MBq (20 mCi) is predicted to deliver 400 mSv (40 rem) to the bone marrow, which is the dose limiting organ. This will serve as a reference dose in additional clinical investigations.

## Supporting Information

Table S1 **Protocol Deviations.** Details are listed about each subject's deviations from the protocol.
(DOCX)

Checklist S1 **TREND Checklist.**
(DOCX)

File S1 **Individual Subject Dosimetry Results DCL-08-001.pdf.**
(PDF)

File S2 **Individual Patient Data Listings DCL-08-001.pdf.**
(PDF)

File S3 **Plasma Concentration DCL-08-001.pdf.**
(PDF)

Protocol S1 **Trial Protocol.**
(PDF)
