# Supplementary material for: A Phase 1 Study of 131I-CLR1404 in Patients with Relapsed or Refractory Advanced Solid Tumors: Dosimetry, Biodistribution, Pharmacokinetics, and Safety
Source: PLoS One. 2014 Nov 17;9(11):e111652. doi: 10.1371/journal.pone.0111652 (PMC4234270; doi:10.1371/journal.pone.0111652)
Supplement: Table S1 — Protocol Deviations. Details are listed about each subject's deviations from the protocol. (DOCX) [file pone.0111652.s001.docx]

Table S: Protocol Deviations. Details are listed about each subject’s deviations from the protocol

| Subject | Day | Deviation |
| --- | --- | --- |
| 101 | Screen | Subject’s INR was not measured for screening criteria as required by protocol. |
| 101 | Screen, Day 03, Day 30, Day 42 | Oxygen saturation (Pulse Ox) not done |
| 101, 102 | Screen, Day 06, Day 14, Day 30, Day 42 | Urine bilirubin level not obtained. |
| 101 | Day 0 | Body weight not done pre-dose because it was done at the screening visit within 24 hours prior to the Day 0 visit. Waiver granted by Sponsor. |
| 101, 102, 103 | Day 0 | Physical exam was not performed at Day 0 pre-dose because a physical exam was performed at the screening visit within 24 hours, 4 days and 24 hours prior to the Day 0 visit, respectively for Subjects 101, 102, and 103. |
| 101 | Day 0 | Chemistry, hematology, and urinalysis were not performed pre-dose because they were done at screening within 24 hours prior to the Day 0 visit. |
| 101 | Day 0 | 30 min post injection vitals not done as per protocol |
| 101 | Day 0 | 5 min PK sample was drawn at 15:19 which was during the 10 minute infusion instead of after the infusion |
| 101 | Day 01 | Subject’s vital signs were not recorded |
| 101 | Day 30 | SaO2 was not measured as per protocol; GGT not performed as per protocol |
| 101 | Day 42 | All subject assessments for Day 42 per protocol were performed on Day 56 due to subject being on vacation and unavailable for follow up. |
| 101 | Day 42 | GGT not done at Day 42 visit. Urinalysis not done (except for protein) at Day 42 visit |
| 102 | Day 0 | Vital signs at 60 min time point were taken at 70 minutes, which is outside the specified range (60 +/- 2 min). |
| 102 | Day 0 | 15 min images were acquired at 40 min post injection due to logistics of obtaining all assessments in a short time frame. This exception was approved by Cellectar. |
| 102 | Day 0, Day 01 | 15 min blood sample for biodistribution taken at 18 mins, outside range of 15 +/- 2 min. 60 min blood sample for biodistribution taken at 72 mins, outside range of 60 +/- 2 min. 18-24 hour sample taken at 24 hours 25 mins, outside range of 18-24 hours +/- 2 min. |
| 102 | Day 0 | 5 min blood sample for PK drawn at 13 min, outside range of 5 +/- 2 min. 60 min blood sample for PK drawn at 67 min, outside range of 60 +/- 2 min. |
| 102 | Day 03 | LDL not obtained per protocol |
| 102 | Day 42 | Subject came in on Day 45 which is outside the specified range of 42 +/- 2 days. |
| 102 | Day 42 | Urinalysis not performed at final visit. |
| 102 | Screen, Day 06, Day 14, Day 30, Day 42 | Urinary bilirubin not collected as required |
| 102 | Day 0 | Chemistry and hematology was not performed on Day 0 (Jan 12th) as these labs were done for screening 24 hours prior (Jan 11th). Cellectar was aware of this. |
| 102 | Day 0, Day 03 | LDL not obtained as required by protocol at Day 0 and day 03. |
| 103 | Day 0 | 30 min vitals were acquired at 42 min due to subject being under the scanner. 4-6 hr vitals were taken at 3 hrs 55 min, which is outside the prescribed range of 4-6 hrs +/- 2 min. |
| 103 | Day 0 | 60 min blood sample for biodistribution was drawn at 68 min, outside range of 60 +/- 2 min |
| 103 | Day 0 | 60 min blood sample for PK was drawn at 68 min, outside range of 60 +/- 2 min. |
| 103 | Day 06 | Subject was seen on Day 7 for Day 6 assessments, because Day 6 was a national holiday. Cellectar was aware of this. |
| 103 | Day 42 | Subject completed all Day 42 assessments on Day 38 post injection. Cellectar approved this waiver. |
| 201 | Screen | INR not obtained on this subject as screening criteria per protocol. |
| 201 | Day 0 | 15 min vitals taken outside of range (15 +/- 2 min). This was due to logistics of ECG, blood, and vitals all at the same time point. |
| 201 | Day 0 | 15 min image start time was 35 min post injection. Protocol states “15 +/- 5 min”. Delay was due to logistics of getting 15 & 30 min blood samples. This was discussed with Cellectar and approved. |
| 201 | Day 30 | Day 35 visit was outside the protocol-defined range of 30 +/- 2 days due to subject vacation. This was discussed with Cellectar and approved. |
| 301 | Screen | INR ≥ 2.0 at the screening visit excludes a subject from the study. At subject screening visit on 03 Aug 2009 no INR was reported on the lab report. Waiver to enroll subject on study requested by site and granted by Cellectar. |
| 302 | Day 0 | 30 minutes post infusion vitals, PK blood sample, and biodistribution blood sample was not done due to subject on scanner at the scheduled time point. |
| 302 | Day 6 | Day 6 physical exam not done due to principal Investigator being out of the office for the day. |
| 401 | Day 0 | Respiration rate for 5 minutes and 15 minutes post dose not collected. |
| 401, 402 | Screen | Subjects received waivers from the sponsor’s medical monitor to be enrolled in the study due to being treated with hormonal therapy for their disease. |
| 401 | Screen | Subject received a second waiver from the sponsor’s medical monitor to be enrolled in the study with a history of iodine allergy. |
| 402 | Day 14 | Per protocol, body weight required at Day 14. Body weight not collected as required. |
| 402 | Day 14, Day 30, Day 42 | Per protocol, serum chemistries required at visit Days 14, 30, & 42. Chemistry not done for above 3 visits. |

Abbreviations: ECG = electrocardiogram; GGT = gamma glutamyltransferase; LDL = low-density lipoprotein; INR = International Normalized Ratio; PK = pharmacokinetics; SaO2 = oxygen saturation
