# Supplementary material for: A Phase 1 Study of 131I-CLR1404 in Patients with Relapsed or Refractory Advanced Solid Tumors: Dosimetry, Biodistribution, Pharmacokinetics, and Safety
Source: PLoS One. 2014 Nov 17;9(11):e111652. doi: 10.1371/journal.pone.0111652 (PMC4234270; doi:10.1371/journal.pone.0111652)
Supplement: File S1 — Individual Subject Dosimetry Results DCL-08-001.pdf. (PDF) [file pone.0111652.s003.pdf]

RESULTS

Figure 1: Individual Subject Results

Duke, Subject 301 (Male)

	Total Body	Kidneys	Liver	Spleen	Lungs	GI	Blood
Fraction 1	1.0	-0.0150	0.0552	0.0263	0.0347	5.00%	0.543
$T_{1/2}$-1 (hr)	10080	6.58	6.84	1.15	2.96		5.5
Fraction 2		0.0356	0.0697	0.0207	0.0431		0.322
$T_{1/2}$-2 (hr)		614	1410	567	∞		146
N (Bq-hr/Bq admin)	265.5	7.4	17.5	4.3	12.1		

Kinetic Data: (the form is $f_1 \cdot \exp(-0.693 \cdot t/T_{1/2}\text{-}1) + f_2 \cdot \exp(-0.693 \cdot t/T_{1/2}\text{-}2)$). All $T_{1/2}$ values are biological half-times.

Estimated Radiation Doses:

Target Organ	mSv/MBq	rem/mCi
Adrenals	7.93E-01	2.93E+00
Brain	5.50E-01	2.04E+00
Breasts	5.50E-01	2.04E+00
Gallbladder Wall	8.10E-01	3.00E+00
LLI Wall	1.10E+00	4.06E+00
Small Intestine	7.27E-01	2.69E+00
Stomach Wall	7.16E-01	2.65E+00
ULI Wall	8.39E-01	3.11E+00
Heart Wall	7.19E-01	2.66E+00
Kidneys	3.45E+00	1.28E+01
Liver	1.57E+00	5.83E+00
Lungs	1.74E+00	6.42E+00
Muscle	6.10E-01	2.26E+00
Ovaries	6.95E-01	2.57E+00
Pancreas	8.21E-01	3.04E+00
Red Marrow	5.55E-01	2.05E+00
Osteogenic Cells	1.23E+00	4.53E+00
Skin	5.08E-01	1.88E+00
Spleen	3.34E+00	1.23E+01
Testes	5.82E-01	2.15E+00
Thymus	6.44E-01	2.38E+00
Thyroid	6.18E-01	2.29E+00
Urinary Bladder Wall	6.64E-01	2.46E+00
Uterus	6.92E-01	2.56E+00
Total Body	6.87E-01	2.54E+00
Effective Dose Equivalent	1.20E+00	4.46E+00
Effective Dose	9.25E-01	3.42E+00

Duke, Subject 302 (Male)

Kinetic Data:

	Total Body	Kidneys	Liver	Spleen	Lungs	GI	Blood
Fraction 1	1.000	0.018	0.030	0.019	0.0068	---	0.68
$T_{1/2}$-1 (hr)	5065	71.4	6.1	0.1	3.73		3.7
Fraction 2			0.037	0.0174	0.029		0.362
$T_{1/2}$-2 (hr)			208	30	∞		133
N (Bq-hr/Bq admin)	251	1.31	5.59	0.66	7.94		

Estimated Radiation Doses:

Target Organ	mSv/MBq	rem/mCi
Adrenals	7.47E-01	2.77E+00
Brain	6.18E-01	2.29E+00
Breasts	5.91E-01	2.19E+00
Gallbladder Wall	7.60E-01	2.81E+00
LLI Wall	7.52E-01	2.78E+00
Small Intestine	7.66E-01	2.83E+00
Stomach Wall	7.31E-01	2.71E+00
ULI Wall	7.54E-01	2.79E+00
Heart Wall	7.40E-01	2.74E+00
Kidneys	8.33E-01	3.08E+00
Liver	6.45E-01	2.39E+00
Lungs	1.22E+00	4.50E+00
Muscle	6.58E-01	2.44E+00
Ovaries	7.72E-01	2.86E+00
Pancreas	7.74E-01	2.86E+00
Red Marrow	5.88E-01	2.18E+00
Osteogenic Cells	1.35E+00	5.01E+00
Skin	5.59E-01	2.07E+00
Spleen	7.43E-01	2.75E+00
Testes	6.53E-01	2.42E+00
Thymus	6.95E-01	2.57E+00
Thyroid	6.88E-01	2.54E+00
Urinary Bladder Wall	7.33E-01	2.71E+00
Uterus	7.73E-01	2.86E+00
Total Body	6.87E-01	2.54E+00
Effective Dose Equivalent	7.94E-01	2.94E+00
Effective Dose	7.38E-01	2.73E+00

Hope, Subject 401 (Female)

Kinetic Data:

	Total Body	Kidneys	Liver	Spleen	Lungs	GI	Blood
Fraction 1	1.0	0.0280	0.0433	0.0045	0.0183	4.20%	0.711
$T_{1/2}$-1 (hr)	4538	0.9273	3.873	21.45	2.095		2.3
Fraction 2		0.0038	0.0507	0.0097	0.0165		0.289
$T_{1/2}$-2 (hr)		2234	∞	1083	∞		169
N (Bq-hr/Bq admin)	263.1	1.0	14.3	2.4	4.6		

Estimated Radiation Doses:

Target Organ	mSv/MBq	rem/mCi
Adrenals	7.52E-01	2.78E+00
Brain	5.91E-01	2.19E+00
Breasts	5.65E-01	2.09E+00
Gallbladder Wall	7.95E-01	2.94E+00
LLI Wall	1.07E+00	3.95E+00
Small Intestine	7.48E-01	2.77E+00
Stomach Wall	7.19E-01	2.66E+00
ULI Wall	8.36E-01	3.09E+00
Heart Wall	7.19E-01	2.66E+00
Kidneys	7.32E-01	2.71E+00
Liver	1.30E+00	4.82E+00
Lungs	8.20E-01	3.03E+00
Muscle	6.34E-01	2.35E+00
Ovaries	7.35E-01	2.72E+00
Pancreas	7.88E-01	2.92E+00
Red Marrow	5.67E-01	2.10E+00
Osteogenic Cells	1.30E+00	4.80E+00
Skin	5.37E-01	1.99E+00
Spleen	1.96E+00	7.24E+00
Testes	6.25E-01	2.31E+00
Thymus	6.63E-01	2.45E+00
Thyroid	6.57E-01	2.43E+00
Urinary Bladder Wall	7.05E-01	2.61E+00
Uterus	7.35E-01	2.72E+00
Total Body	6.78E-01	2.51E+00
Effective Dose Equivalent	8.51E-01	3.15E+00
Effective Dose	7.73E-01	2.86E+00

Hope, Subject 402 (Male)

Kinetic Data:

	Total Body	Kidneys	Liver	Spleen	Lungs	GI	Blood
Fraction 1	1.0	0.0302	0.0390	0.0069	0.0076	----	0.58
$T_{1/2}$-1 (hr)	3651	0.2181	4.272	1.58	21.840		3.3
Fraction 2		0.0061	0.0367	0.0085	0.0063		0.362
$T_{1/2}$-2 (hr)		1542		3500			162
N (Bq-hr/Bq admin)	263.2	1.5	8.9	2.3	2.0		

Estimated Radiation Doses:

Target Organ	mSv/MBq	rem/mCi
Adrenals	7.51E-01	2.78E+00
Brain	6.13E-01	2.27E+00
Breasts	5.76E-01	2.13E+00
Gallbladder Wall	7.81E-01	2.89E+00
LLI Wall	7.48E-01	2.77E+00
Small Intestine	7.65E-01	2.83E+00
Stomach Wall	7.36E-01	2.72E+00
ULI Wall	7.55E-01	2.79E+00
Heart Wall	7.20E-01	2.66E+00
Kidneys	9.29E-01	3.44E+00
Liver	8.90E-01	3.29E+00
Lungs	4.98E-01	1.84E+00
Muscle	6.52E-01	2.41E+00
Ovaries	7.68E-01	2.84E+00
Pancreas	7.92E-01	2.93E+00
Red Marrow	5.81E-01	2.15E+00
Osteogenic Cells	1.34E+00	4.96E+00
Skin	5.54E-01	2.05E+00
Spleen	1.89E+00	7.00E+00
Testes	6.48E-01	2.40E+00
Thymus	6.76E-01	2.50E+00
Thyroid	6.77E-01	2.51E+00
Urinary Bladder Wall	7.38E-01	2.73E+00
Uterus	7.69E-01	2.84E+00
Total Body	6.81E-01	2.52E+00
Effective Dose Equivalent	7.85E-01	2.91E+00
Effective Dose	6.88E-01	2.55E+00

JHU, Subject 201 (Male)

Kinetic Data:

	Total Body	Kidneys	Liver	Spleen	Lungs	GI	Blood
Fraction 1	1.0	0.0	0.0197	0.0023	0.0158	7.00%	0.66
$T_{1/2}$-1 (hr)	2471		3.588	0.26	4.468		2.5
Fraction 2			0.0655	0.0063	0.0178		0.5
$T_{1/2}$-2 (hr)			1863	1935	∞		780
N (Bq-hr/Bq admin)	257.0		16.6	1.6	5.0		

Estimated Radiation Doses:

Target Organ	mSv/MBq	rem/mCi
Adrenals	7.37E-01	2.73E+00
Brain	5.71E-01	2.11E+00
Breasts	5.49E-01	2.03E+00
Gallbladder Wall	7.94E-01	2.94E+00
LLI Wall	1.30E+00	4.79E+00
Small Intestine	7.50E-01	2.78E+00
Stomach Wall	6.96E-01	2.57E+00
ULI Wall	9.13E-01	3.38E+00
Heart Wall	7.01E-01	2.59E+00
Kidneys	6.94E-01	2.57E+00
Liver	1.48E+00	5.46E+00
Lungs	8.63E-01	3.19E+00
Muscle	6.15E-01	2.28E+00
Ovaries	7.21E-01	2.67E+00
Pancreas	7.63E-01	2.82E+00
Red Marrow	5.52E-01	2.04E+00
Osteogenic Cells	1.26E+00	4.65E+00
Skin	5.20E-01	1.92E+00
Spleen	1.39E+00	5.14E+00
Testes	6.05E-01	2.24E+00
Thymus	6.43E-01	2.38E+00
Thyroid	6.34E-01	2.35E+00
Urinary Bladder Wall	7.07E-01	2.62E+00
Uterus	7.16E-01	2.65E+00
Total Body	6.63E-01	2.45E+00
Effective Dose Equivalent	8.41E-01	3.11E+00
Effective Dose	7.78E-01	2.88E+00

GTU, Subject 101 (Female)

Kinetic Data:

	Total Body	Kidneys	Liver	Spleen	Lungs	GI	Blood
Fraction 1	1.0	0.0125	0.0113	0.0101	0.0143	0.00%	0.503
$T_{1/2}$-1 (hr)	5935	0.0699	4.62	43.6	3.90		2.5
Fraction 2		0.0062	0.0387	0.0057	0.0310		0.497
$T_{1/2}$-2 (hr)		533	495	∞	3779		177
N (Bq-hr/Bq admin)	265.7	1.3	7.8	2.1	8.2		

Estimated Radiation Doses:

Target Organ	mSv/MBq	rem/mCi
Adrenals	7.50E-01	2.78E+00
Brain	6.06E-01	2.24E+00
Breasts	5.83E-01	2.16E+00
Gallbladder Wall	7.67E-01	2.84E+00
LLI Wall	7.38E-01	2.73E+00
Small Intestine	7.55E-01	2.79E+00
Stomach Wall	7.31E-01	2.71E+00
ULI Wall	7.44E-01	2.75E+00
Heart Wall	7.35E-01	2.72E+00
Kidneys	8.23E-01	3.05E+00
Liver	8.14E-01	3.01E+00
Lungs	1.26E+00	4.65E+00
Muscle	6.49E-01	2.40E+00
Ovaries	7.58E-01	2.80E+00
Pancreas	7.86E-01	2.91E+00
Red Marrow	5.80E-01	2.15E+00
Osteogenic Cells	1.33E+00	4.92E+00
Skin	5.50E-01	2.03E+00
Spleen	1.75E+00	6.49E+00
Testes	6.40E-01	2.37E+00
Thymus	6.85E-01	2.53E+00
Thyroid	6.75E-01	2.50E+00
Urinary Bladder Wall	7.20E-01	2.66E+00
Uterus	7.59E-01	2.81E+00
Total Body	6.85E-01	2.53E+00
Effective Dose Equivalent	8.54E-01	3.16E+00
Effective Dose	7.67E-01	2.84E+00

GTU, Subject 102 (Male)

Kinetic Data:

	Total Body	Kidneys	Liver	Spleen	Lungs	GI	Blood
Fraction 1	1.0	0.0177	0.0116	0.0023	0.0065	0.00%	0.423
$T_{1/2}$-1 (hr)	6967	0.758	9	2.72	0.206		3.1
Fraction 2		0.0024	0.0434	0.0029	0.0069		0.577
$T_{1/2}$-2 (hr)		∞	867	∞	1364		86.3
N (Bq-hr/Bq admin)	263	0.7	10.0	0.8	1.7		

Estimated Radiation Doses:

Target Organ	mSv/MBq	rem/mCi
Adrenals	7.45E-01	2.76E+00
Brain	6.15E-01	2.28E+00
Breasts	5.77E-01	2.13E+00
Gallbladder Wall	7.88E-01	2.91E+00
LLI Wall	7.50E-01	2.77E+00
Small Intestine	7.66E-01	2.83E+00
Stomach Wall	7.27E-01	2.69E+00
ULI Wall	7.56E-01	2.80E+00
Heart Wall	7.21E-01	2.67E+00
Kidneys	5.78E-01	2.14E+00
Liver	9.70E-01	3.59E+00
Lungs	4.59E-01	1.70E+00
Muscle	6.53E-01	2.42E+00
Ovaries	7.70E-01	2.85E+00
Pancreas	7.75E-01	2.87E+00
Red Marrow	5.81E-01	2.15E+00
Osteogenic Cells	1.35E+00	4.98E+00
Skin	5.56E-01	2.06E+00
Spleen	8.30E-01	3.07E+00
Testes	6.51E-01	2.41E+00
Thymus	6.78E-01	2.51E+00
Thyroid	6.80E-01	2.52E+00
Urinary Bladder Wall	7.58E-01	2.81E+00
Uterus	7.72E-01	2.86E+00
Total Body	6.79E-01	2.51E+00
Effective Dose Equivalent	7.13E-01	2.64E+00
Effective Dose	6.60E-01	2.44E+00

GTU, Subject 103(Male)

Kinetic Data:

	Total Body	Kidneys	Liver	Spleen	Lungs	GI	Blood
Fraction 1	1.0	0.0467	0.0621	0.0129	0.0181	0.00%	0.469
$T_{1/2}$-1 (hr)	2716	0.1617	0.160	0.16	0.158		1.9
Fraction 2		0.0033	0.0514	0.0076	0.0118		0.531
$T_{1/2}$-2 (hr)		99	669	171	1135		228
N (Bq-hr/Bq admin)	229.8	0.32	11.1	1.0	2.8		

Estimated Radiation Doses:

Target Organ	mSv/MBq	rem/mCi
Adrenals	6.51E-01	2.41E+00
Brain	5.27E-01	1.95E+00
Breasts	4.99E-01	1.85E+00
Gallbladder Wall	6.96E-01	2.58E+00
LLI Wall	6.42E-01	2.38E+00
Small Intestine	6.59E-01	2.44E+00
Stomach Wall	6.30E-01	2.33E+00
ULI Wall	6.52E-01	2.41E+00
Heart Wall	6.29E-01	2.33E+00
Kidneys	3.90E-01	1.44E+00
Liver	1.03E+00	3.81E+00
Lungs	5.65E-01	2.09E+00
Muscle	5.62E-01	2.08E+00
Ovaries	6.60E-01	2.44E+00
Pancreas	6.78E-01	2.51E+00
Red Marrow	5.02E-01	1.86E+00
Osteogenic Cells	1.15E+00	4.27E+00
Skin	4.77E-01	1.77E+00
Spleen	9.28E-01	3.43E+00
Testes	5.57E-01	2.06E+00
Thymus	5.86E-01	2.17E+00
Thyroid	5.83E-01	2.16E+00
Urinary Bladder Wall	6.53E-01	2.41E+00
Uterus	6.62E-01	2.45E+00
Total Body	5.92E-01	2.19E+00
Effective Dose Equivalent	6.60E-01	2.44E+00
Effective Dose	5.99E-01	2.22E+00
