# Supplementary material for: A Phase 1 Study of 131I-CLR1404 in Patients with Relapsed or Refractory Advanced Solid Tumors: Dosimetry, Biodistribution, Pharmacokinetics, and Safety
Source: PLoS One. 2014 Nov 17;9(11):e111652. doi: 10.1371/journal.pone.0111652 (PMC4234270; doi:10.1371/journal.pone.0111652)
Supplement: File S2 — Individual Patient Data Listings DCL-08-001.pdf. (PDF) [file pone.0111652.s004.pdf]

Appendix 16.4
Cellectar, Inc.
Protocol No. DCL-08-001
INDIVIDUAL PATIENT DATA LISTINGS

Listing 1
Inclusion Criteria

Criteria Number	Criteria
1	Patient has relapsed or refractory advanced solid tumors and has exhausted standard treatment options or no standard therapy exists.
2	Patient has a sufficient window of time to complete the washout period of 2 weeks (if patient has received standard therapy) or 4 weeks (if patient has received experimental therapy), dosimetry data acquisition (14 days), and the follow-up safety assessment period (4 weeks) prior to beginning another cycle of a therapeutic regimen.
3	Patient is ambulatory with an ECOG performance status of 0 or 1 and an estimated life expectancy of => 4 months.
4	Patient is 18 years or older.
5	Patient is judged by the Investigator to have the initiative and means to be compliant with the protocol and is within geographical proximity to make the required study visits.
6	Patient or his/her legal representatives have the ability to read, understand and provide written informed consent for the initiation of any study related procedures.
7	Patient is of childbearing potential and has a negative serum pregnancy test within 24 hours of start of treatment.
8	Patient agrees to use an effective method of contraception (e.g., oral contraceptives, double-barrier methods such as a condom and a diaphragm,intrauterine device, Norplant, Depo-Provera) during the study and for 90 days following the infusion of study medication.
9	Patient has brain metastasis and his/her clinical condition has been stable for at least one month.

Listing 1
Inclusion Criteria

		Inclusion Criteria								
Center	Patient	1	2	3	4	5	6	7	8	9
XVO_001	101	Yes	Yes	Yes	Yes	Yes	Yes	NA	NA	NA
	102	Yes	Yes	Yes	Yes	Yes	Yes	NA	Yes	NA
	103	Yes	Yes	Yes	Yes	Yes	Yes	NA	Yes	NA
XVO_003	201	Yes	Yes	Yes	Yes	Yes	Yes	NA	Yes	NA
XVO_005	301	Yes	Yes	Yes	Yes	Yes	Yes	NA	Yes	NA
	302	Yes	Yes	Yes	Yes	Yes	Yes	NA	Yes	NA
XVO_007	401	Yes	Yes	Yes	Yes	Yes	Yes	NA	NA	NA
	402	No	Yes	Yes	Yes	Yes	Yes	NA	Yes	NA

Listing 2
Exclusion Criteria

Criteria Number	Criteria
1	Patient or physician plans concomitant chemotherapy, therapeutic radiation treatment, and/or biological treatment for cancer including immunotherapy while on study.
2	Patient has had more than 25% of the total bone marrow irradiated.
3	Patient has diffuse lung disease or interstitial spread of carcinoma.
4	Patient’s bladder or rectum is within a prior radiation therapy field and a dose of greater than 45 Gy was administered.
5	Patients with total therapeutic radiation dose in excess of 25 Gy to the kidney, 45 Gy to brain, 45 Gy to stomach, 18 Gy to lung, or 25 Gy to liver.
6	Patient has received total-body irradiation in the past.
7	Patient has extradural tumor in contact with the spinal cord, or tumor(s) located where swelling in response to therapy may impinge upon the spinal cord.
8	Patient has received prior radiation therapy or chemotherapy within 2 weeks of the start of the study.
9	Patient has another active medical condition(s) or organ disease(s) that may either compromise his/her safety or interfere with the safety and/or outcome evaluation of the study drug.
10a	The Patient has WBC < 3000/uL.
10b	The Patient has absolute neutrophil count < 1500/uL.
10c	The Patient has platelets < 150,000/uL.
10d	The Patient has hemoglobin <= 11.0 gm/dL.
10e	The Patient has total bilirubin > 1.5 x upper limit of normal for age.
10f	The Patient has SGOT or SGPT > 3 x upper limit of normal for age if no liver metastases or > 5 x upper limit of normal for age in the presence of liver metastases.
10g	The Patient has serum creatinine > 1.5 x upper limit of normal for age.
10h	The Patient has INR => 2.
11	Patient has been treated with an investigational drug, investigational biologic, or investigational therapeutic device within 30 days of initiating study treatment.
12	Patient has received severely marrow toxic drugs (e.g. nitrosoureas, mitomycin).
13	Patient has received prior treatment with Iodine-131 in the past five years.
14	Patient is currently receiving hemodialysis.
15	Patient has received blood transfusion (s) within 60 days of study start.
16	Patient has received growth factor therapy within 60 days of study start.
17	Patient has received prior stem cell transplantation.
18	Patient has clinically evident ascites or peritoneal carcinomatosis.

Note: The original text of criteria 5 - Patient has received therapeutic radiation dose in the past year in excess of 25 Gy to the kidney, 45 Gy to brain, 45 Gy to stomach, 18 Gy to lung, or 25 Gy to liver - was presented on the CRF.
Note: Blank = Not Done.

Listing 2
Exclusion Criteria

Criteria Number	Criteria
19	Patient has clinically significant cardiac co-morbidities including: congestive heart failure (New York Heart Association class III-IV heart disease), a LVEF< 40%,unstable angina pectoris, serious cardiac arrhythmia requiring medication or a pacemaker, myocardial infarction within the past six months.
20	Patient has clinically significant pulmonary impairment defined as a SaO2 on room air of 93% or less.
21	Patient is currently or has recently (within 1 month) been treated with thrombolytic agents, or full-dose anticoagulants (except to maintain patency of preexisting, permanent indwelling IV catheters). Of note, therapy with low-molecular weight heparin is acceptable as long as the INR < 2.0.
22	Patient has uncontrolled hypertension as defined by SBP > 150 mmHg, DBP > 100 mmHg or patient has uncontrolled diabetes that would compromise his/her safety or interfere with the safety and/or outcome evaluation of the study drug.
23	Patient has Grade II-IV peripheral vascular disease or has had peripheral vascular surgery within the past year.
24	Patient has had major surgery within 4 weeks of the start of the study.
25	Patient is known positive for HIV, Hepatitis C (active, previously treated or both), or is Hepatitis B core antigen positive.
26	Patient is currently taking more than 325 mg/day of aspirin.
27	Patient is pregnant or lactating.
28	Patient has a colostomy/ileostomy.
29	Patient has poor venous access and will be unable to receive study drug into a peripheral venous catheter.
30	Patient has had prior allergic reactions to iodine, or other study agents.
31	Patient has had a significant traumatic injury within the past 4 weeks.
32	Patient has an ongoing or active infection requiring antibiotics or with a fever > 38.1oC (>101º F) within 3 days of the first scheduled day of dosing.
33	Patient is hospitalized.

Note: The original text of criteria 5 - Patient has received therapeutic radiation dose in the past year in excess of 25 Gy to the kidney, 45 Gy to brain, 45 Gy to stomach, 18 Gy to lung, or 25 Gy to liver - was presented on the CRF.
Note: Blank = Not Done.

Listing 2
Exclusion Criteria

Center	Patient	Exclusion Criteria 1	2	3	4	5	6	7	8	9	10a	10b	10c	10d	10e	10f	10g	10h	11	12	13	14
XVO_001	101	No	No	No	No	No	No	No	No	No	No	No	No	No	No	No	No		No	No	No	No
	102	No	No	No	No	No	No	No	No	No	No	No	No	No	No	No	No	No	No	No	No	No
	103	No	No	No	No	No	No	No	No	No	No	No	No	No	No	No	No	No	No	No	No	No
XVO_003	201	No	No	No	No	No	No	No	No	No	No	No	No	No	No	No	No		No	No	No	No
XVO_005	301	No	No	No	No	No	No	No	No	No	No	No	No	No	No	No	No		No	No	No	No
	302	No	No	No	No	No	No	No	No	No	No	No	No	No	No	No	No	No	No	No	No	No
XVO_007	401	No	No	No	No	No	No	No	No	No	No	No	No	No	No	No	No	No	No	No	No	No
	402	No	No	No	No	No	No	No	No	No	No	No	No	No	No	No	No	No	No	No	No	No

Note: The original text of criteria 5 - Patient has received therapeutic radiation dose in the past year in excess of 25 Gy to the kidney, 45 Gy to brain, 45 Gy to stomach, 18 Gy to lung, or 25 Gy to liver - was presented on the CRF.
Note: Blank = Not Done.

Listing 2
Exclusion Criteria

		Exclusion Criteria																		
Center	Patient	15	16	17	18	19	20	21	22	23	24	25	26	27	28	29	30	31	32	33
XVO_001	101	No	No	No	No	No	No	No	No	No	No	No	No	NA	No	No	No	No	No	No
	102	No	No	No	No	No	No	No	No	No	No	No	No	NA	No	No	No	No	No	No
	103	No	No	No	No	No	No	No	No	No	No	No	No	NA	No	No	No	No	No	No
XVO_003	201	No	No	No	No	No	No	No	No	No	No	No	No	NA	No	No	No	No	No	No
XVO_005	301	No	No	No	No	No	No	No	No	No	No	No	No	NA	No	No	No	No	No	No
	302	No	No	No	No	No	No	No	No	No	No	No	No	NA	No	No	No	No	No	No
XVO_007	401	No	No	No	No	No	No	No	No	No	No	No	No	NA	No	No	Yes	No	No	No
	402	No	No	No	No	No	No	No	No	No	No	No	No	NA	No	No	No	No	No	No

Note: The original text of criteria 5 - Patient has received therapeutic radiation dose in the past year in excess of 25 Gy to the kidney, 45 Gy to brain, 45 Gy to stomach, 18 Gy to lung, or 25 Gy to liver - was presented on the CRF.
Note: Blank = Not Done.

Listing 3
Informed Consent and Eligibility Criteria Exemption

Center	Patient	Informed Consent Date	All Study Entry Criteria Met?	If no, specify	Protocol Exception Date	Sponsor Representative
XVO_001	101	09NOV2009	No	INR MEASUREMENT WAS NOT OBTAINED IN SCREENING PROCESS. THIS IS A PROTOCOL VIOLATION - NO EXCEPTION WAS GRANTED.		
	102	11DEC2009	Yes			
	103	11JAN2010	Yes			
XVO_003	201	10NOV2009	No	INR NOT OBTAINED PER PROTOCOL AT SCREENING		
XVO_005	301	03AUG2009	No	INR LAB WAS NOT DRAWN AS PART OF THE SCREENING PROCEDURES ON 03/AUG/2009	04AUG2009	ERNEST ALLEN
	302	16NOV2009	Yes			
XVO_007	401	20OCT2009	No	PER EXCL CRITERIA#30 THE PT. MEDT HIS INDICATED THAT SHE IS ALLERGIC TO IODINE WHICH IS ACTUALLY AN ALLERGY TO CT CONTRAST MEDIA. SPONSOR AND FDA CONTACTED FOR WAIVER TO ENROLL PT WITH PREMEDICATION.	09NOV2009	ERNEST ALLEN
	402	20NOV2009	No	THE PT HAS DENOVO METASTATIC PROSTATE CANCER & IS ON HIS 1ST LINE OF THERAPY WITH CASODEX& LUPRON.THERE ARE NO SAFETY CONCERNS ENROLLING THE PT ON THE DOSIMETRY CLINICAL TRIAL PER THE SPONSOR MM	30NOV2009	ROBERT SHEPARD.M.D

Listing 4
Demographics

Center	Patient	Date of Birth	Age (Years)	Gender	Ethnicity	Race
XVO_001	101	15JAN1938	71	Female		White
	102	18SEP1963	46	Male		Black or African American
	103	31JUL1951	58	Male		White
XVO_003	201	20AUG1940	69	Male		White
XVO_005	301	16FEB1955	54	Male		White
	302	16MAR1943	66	Male		White
XVO_007	401	05MAY1949	60	Female	Hispanic or Latino	
	402	02NOV1957	52	Male		White

Listing 5
Medical / Surgical History

Center	Patient	Diagnosis/Condition/Surgery	Diagnosis/ Surgery Date	Ongoing	CTCAE Grade [a]	Comments
XVO_001	101	ANXIETY	UNKNOWN	Yes	1	
		ATELECTASIS	--NOV2008			
		CELLULITIS	--NOV2008			
		EPISTAXIS	UNKNOWN	Yes	1	
		HYPERTENSION	UNKNOWN	Yes	2	
		KLEBSIELLA PNEUMONIA	--NOV2008			
		MASS LOWERPOLE RIGHT KIDNEY	27FEB2009	Yes	1	
		MILD DARKNESS IN DIP/PIP JOINTS	UNKNOWN			-RESOLVED DOCUMENTED ON 09-NOV-2009
		OVARIAN CYST REMOVAL	-----2008			
		PAIN BILAT KNEES FALL ON RIGHT KNEE	-----2008	Yes	1	
		PULMONARY EMBOLUS	-----2008			
		THYROID ABLATION	UNKNOWN			
		UTI	--NOV2008			
	102	EPISTAXIS	-----2009	Yes	1	
		HYPERTENSION	UNKNOWN	Yes	1	
		ACNEIFORM RASH: THORAX	UNKNOWN	Yes	1	
		ANOREXIA POST CHEMO	-----2009			
		DRY COUGH	-----2009			
		DYSGEUSIA POST CHEMO	-----2009			
		FATIGUE POST CHEMO	-----2009			
		HYPERCHOLESTEROLEMIA	-----2008	Yes	1	
		HYPO-THYROIDISM	-----1997	Yes	1	
		INTERMITTENT CONSTIPATION	UNKNOWN	Yes	1	
		MILD RIGHT KNEE PAIN	UNKNOWN	Yes	1	
		MYOCARDIAL INFARCTION INF WALL	UNKNOWN			

Note: [a] Only recorded if ongoing.
-- =Date unknown or partially unknown.

Listing 5
Medical / Surgical History

Center	Patient	Diagnosis/Condition/Surgery	Diagnosis/ Surgery Date	Ongoing	CTCAE Grade [a]	Comments
XVO_001	102	OBESITY	UNKNOWN	Yes	4	
		PARONYCHIA GREAT TOE NAIL	-----2009	Yes	2	
		PARONYCHIA HANDS	-----2009			
		PELVIC ABSCESS	-----2007			SURGERY
		PROLONGED QT INTERVAL	UNKNOWN	Yes	1	
		RASH ON FACE, ARM, BACK	UNKNOWN	Yes	1	
		RECTAL BLEEDING	-----2006			COLONOSCOPY PERFORMED
		RT CALF DISCOMFORT	-----2008	Yes	1	
		SEVERE SENSORY NEUROPATHY SECONDARY TO OXALIPLATIN	-----2007			
		SKIN DISCOLORATION BILATERAL FEET	-----2008			
	103	HYPERTENSION	-----2004	Yes	1	
		PULMONARY EMBOLUS	15AUG2007			
		ANASTOMOSIS IN RECTUM	13JUL2009			
		BACK STIFFNESS (MILD)	UNKNOWN	Yes	1	
		DIVERTICULOSIS	13JUL2009			
		MACULAR DEGENERATION	-----2009	Yes	1	
		MEDIPORT PLACEMENT	-----2007	Yes		
		MILD FATIGUE	UNKNOWN	Yes	1	
		PRIMARY HYPERCOAGULABLE STATE	UNKNOWN	Yes	1	
		R INGUINAL HERNIORRHAPHY	-----1999			
XVO_003	201	ATRIAL FIBRILLATION POST-OP	25OCT2005			RESOLVED JULY 2006
		BENIGN PROSTATIC HYPERTROPHY	-----2004	Yes	2	ON MEDICAL THERAPY
		CHRONIC EMPHYSEMA/COPD	UNKNOWN	Yes	1	PATIENT NOTED THIS MENTIONED ON PRIOR CT; NO OXYGEN REQUIREMENT
		CHRONIC RHINITIS	16JUN2008	Yes	1	
		CLAUDICATION	16JUN2008	Yes	1	

Note: [a] Only recorded if ongoing.
-- =Date unknown or partially unknown.

Listing 5
Medical / Surgical History

Center	Patient	Diagnosis/Condition/Surgery	Diagnosis/ Surgery Date	Ongoing	CTCAE Grade [a]	Comments
XVO_003	201	COLON BIOPSY	-----2002			BENIGN
		COLON POLYPS/COLONOSCOPY	-----2002			BENIGN
		COUGH WITH CLEAR SPUTUM	16JUN2008			
		DIARRHEA	-----2005	Yes	1	INTERMITTENT LOOSE STOOLS 1-2 PER WEEK
		DIZZINESS	-----2005			
		DYSPHAGIA	-----2005			
		DYSPNEA DURING CHEMOTHERAPY	16JUN2008			
		ELEVATED GGT	10NOV2009	Yes	1	
		EMPYEMA	--NOV2005			POST-OP
		ESOPHAGOSCOPY WITH DILATATION	18MAY2006			LAST OF SERIES OF DILATATION ON 06MAY2008
		FATIGUE DURING CHEMOTHERAPY	21MAY2009			
		HEMORRHOIDS	16JUN2008	Yes	1	
		HOARSENESS	-----2005	Yes	1	
		HYPERLIPIDEMIA	UNKNOWN	Yes	1	IMPROVED AFTER CHEMOTHERAPY AND WEIGHT LOSS
		JEJUNOSTOMY TUBE REPLACEMENT	10APR2006			
		LEFT HIP FRACTURE	--SEP2008			HEALED
		LOW BACK PAIN	27AUG2009	Yes	2	
		METHICILLIN-RESISTANT STAPHYLOCOCCUS AUREUS INFECTION POST-OPERATIVE	--APR2006			
		NEUROPATHY	21MAY2009	Yes	1	
		ORTHOSTATIC HYPOTENSION	25SEP2005	Yes	1	
		OSTEOPENIA	14MAY2009	Yes	1	
		OSTEOPOROSIS				
		POST-OPERATIVE COMPLICATIONS	-----2005			ANASTOMOTIC LEAK AND STRICTURE
		PROSTATE BIOPSY	-----2004			BENIGN
		SPONDYLOSIS	14MAY2009	Yes	1	

Note: [a] Only recorded if ongoing.
-- =Date unknown or partially unknown.

Listing 5
Medical / Surgical History

Center	Patient	Diagnosis/Condition/Surgery	Diagnosis/ Surgery Date	Ongoing	CTCAE Grade [a]	Comments
XVO_003	201	URINARY FREQUENCY	-----2004	Yes	1	
		VASCULAR ATHEROSCLEROSIS	UNKNOWN	Yes	1	NOT TREATED
XVO_005	301	OBESITY	UNKNOWN	Yes	3	
		NEUROPATHY	UNKNOWN	Yes	1	
		COUGH	--JUL2006	Yes	1	
		DIABETES	UNKNOWN	Yes	2	
		ESOPHAGITIS	12DEC2006			
		GASTROESOPHAGEAL REFLUX	UNKNOWN	Yes	2	
		HIGH BLOOD PRESSURE	UNKNOWN	Yes	2	
		HIGH CHOLESTEROL	02APR2009	Yes	1	CONTROL BY MEDICATION
		IDIOPATHIC HIVES	-----2005			NOT PRESENT AT SCREENING
		LEFT WEDGE RESECTION OF LUNG (LINGULAR)	17NOV2006			
		NASAL DRAINAGE	03AUG2009	Yes	1	
		PHLEGM PRODUCTION	13MAR2007	Yes	2	
		RIGHT HEMICOLECTOMY	08NOV2004			
		RIGHT UPPER EXTREMITY DEEP VEIN THROMBOSIS	13FEB2007			
		SCIATICA PAIN	UNKNOWN	Yes	2	
		SHORTNESS OF BREATH	17NOV2006	Yes	1	
		UMBILICAL HERNIA	--NOV2004			UNDERWENT REPAIR
		VENTRAL HERNIA	UNKNOWN	Yes	2	
		WHEEZING	--MAY2009	Yes	1	
	302	HYPERCHOLESTEROLEMIA	22SEP2008	Yes	1	
		ANKLE FRACTURE	-----2003			
		BACK PAIN	-----2005	Yes	1	
		LEFT ANKLE SURGERY	31MAR2003			

Note: [a] Only recorded if ongoing.
-- =Date unknown or partially unknown.

Listing 5
Medical / Surgical History

Center	Patient	Diagnosis/Condition/Surgery	Diagnosis/ Surgery Date	Ongoing	CTCAE Grade [a]	Comments
XVO_005	302	SENSORY NEUROPATHY (LEFT HEEL)	13JUL2009	Yes	1	
		SKULL FRACTURE	-----1991			
		URINARY OBSTRUCTION	14DEC2009	Yes	2	
		VERTEBRAE FRACTURE	-----2005			
XVO_007	401	HYPERTENSION	UNKNOWN			
		HYPERCHOLESTEROLEMIA	UNKNOWN	Yes	1	
		OBESITY	UNKNOWN	Yes	4	
		DEPRESSION	-----2008			
	402	CONSTIPATION	UNKNOWN	Yes	1	
		FREQUENT URINATION	UNKNOWN	Yes	1	
		HERNIA REPAIR	-----1992			
		JOINT PAIN (HIP AND BACK)	UNKNOWN	Yes	1	
		URINARY INCONTINENCE	UNKNOWN	Yes	2	

Note: [a] Only recorded if ongoing.
-- =Date unknown or partially unknown.

Listing 6.1
Past Treatment For Cancer: Radiation Therapy

Center	Patient	Start Date	Stop Date	Site(s)	Dose (Gy)
XVO_003	201	03AUG2005	07SEP2005	DISTAL ESOPHAGUS	45
XVO_005	301	12DEC2006	10JAN2007	LEFT LUNG, T4-T8 VERTEBRAL BODY	40

Listing 6.2
Past Treatment For Cancer: Radiation Therapy - Cumulative Non-Target Exposure

		Median Dose (Gy) to						
Center	Patient	Bladder	Rectum	Kidneys	Brain	Stomach	Lungs	Liver
XVO_003	201	0	0	0	0	0	7.6	12.7
XVO_005	301					0.6	2	

Listing 6.3
Past Treatment For Cancer: Surgery

Center	Patient	Date	Surgery
XVO_001	101	18NOV2008	HEMICOLECTOMY
		18NOV2008	OVARIAN CYST REMOVAL
		18NOV2008	UMBILICAL TUMOR RESECTION
	102	--FEB2007	PARTIAL SIGMOID RESECTION
	103	18SEP2007	SIGMOID COLECTOMY WITH LYMPH NODE DISSECTION
		18SEP2007	SIGMOIDOSCOPY
XVO_003	201	25OCT2005	TRANSHIATAL ESOPHAGECTOMY
XVO_005	301	20APR2009	LEFT LOWER LOBECTOMY
		17NOV2006	LEFT WEDGE RESECTION OF LUNG (LINGULAR)
		08NOV2004	RIGHT HEMICOLECTOMY

-- =Date unknown or partially unknown

Listing 6.4
Past Treatment For Cancer: Chemotherapy

Center	Patient	Start Date	Stop Date	Treatment/Regimen
XVO_001	101	09JAN2009	09JUN2009	FOLFOX 8 CYCLES
	102	--FEB2007	--APR2007	FOLFOX + OXALIPATIN
		--APR2007	-----2007	FOLFOX + AVASTIN
		-----2007	--FEB2008	5-FU + LEUCOVARIN + AVASTIN
		--FEB2008	--APR2008	XELODA + AVASTIN
		--JUL2009	13NOV2009	IRINOTECAN + AVASTIN + ERBITUX
		18JUN2008	--JUL2009	ERBITUX + IRINOTECAN + RAD
	103	13NOV2007	19FEB2008	FOLFIRI, AVASTIN, ERBITUX
		04MAR2008	--DEC2008	XELODA
XVO_003	201	01AUG2005	29AUG2005	TAXOL AND CISPLATIN (CONCOMMITANT WITH RADIATION THERAPY)
		19JUN2008	02SEP2008	CISPLATIN AND IRINOTECAN
		04DEC2008	14MAY2009	IRINOTECAN
XVO_005	301	12DEC2006	12JAN2007	XELODA
		20DEC2004	--JUN2005	FOLFOX (LEUCOVORIN/5-FU/OXALIPLATIN)

-- =Date unknown or partially unknown

Listing 6.4
Past Treatment For Cancer: Chemotherapy

Center	Patient	Start Date	Stop Date	Treatment/Regimen
XVO_005	301	13MAR2007	15MAY2007	XELODA
		20FEB2007	10MAR2009	AVASTIN
		13MAR2007	15MAY2007	IRINOTECAN HCL
		22MAY2007	06FEB2008	FLUOROURACIL
	302	24JUL2008	16NOV2009	ZOMETA
		16FEB2009	24AUG2009	DOCETAXEL

-- =Date unknown or partially unknown

Listing 6.5
Past Treatment For Cancer: Hormonal, Immunological, Biological and Other Therapy

Center	Patient	Start Date	Stop Date	Treatment/Regimen
XVO_001	101	09JAN2009	21APR2009	AVASTIN 7 CYCLES
XVO_005	301	17JUN2008	26AUG2008	AVX701 (INVESTIGATIONAL TREATMENT)
		--SEP2008	--OCT2008	INTERFERON ALPHA 2B
	302	08AUG2007	--FEB2008	ELIGARD
		01AUG2007	19DEC2008	CASODEX
		21AUG2008		LEUPROLIDE ACETATE (CONTINUING)
XVO_007	401	15FEB2008		ANASTROZOLE, FASLODEX (ONGOING)
	402	06NOV2009		CASODEX (ONGOING)
		06NOV2009		LUPRON (ONGOING)

-- =Date unknown or partially unknown

Listing 7
Primary Tumor

Center	Patient	Primary Solid Tumor	Histological Type	Method of Diagnosis	Date of First Diagnosis	Metastatic Disease	Current Cancer Stage	Sites of Metastasis
XVO_001	101	COLON	ADENO CARCINOMA	HISTOLOGICAL	19DEC2008	YES	IV	Ovary, Abdominal wall, Other (UMBILICUS)
	102	COLON	UNK	HISTOLOGICAL	--DEC2006	YES	IV T3 N1 M1	Lymph node, Kidney, Liver, Lung
	103	COLON	ADENOCARCINOMA	HISTOLOGICAL	-----2007	YES	XT1N2MX	Lymph node
XVO_003	201	ESOPHAGEAL	MODERATELY DIFFERENTIATED ADENOCARCINOMA	HISTOLOGICAL	05JUL2005	YES	T4	Lymph node, Liver, Lung
XVO_005	301	COLON	ADENOCARCINOMA	HISTOLOGICAL	12NOV2004	YES	4	Lung
	302	PROSTATIC	ADENOCARCINOMA	HISTOLOGICAL	08AUG2007	YES	4	Bone
XVO_007	401	LEFT BREAST CANCER	LOBULAR CARCINOMA	HISTOLOGICAL	15JAN2008	YES	IV	Spleen
	402	PROSTATE CANCER	ADENOCARCINOMA	HISTOLOGICAL	01JUL2009	YES	IV	Lung, Bone

-- =Date unknown or partially unknown
UNK = Unknown

Listing 8
I-131-CLR1401 Dosing Details

		Pre infusion Syringe		Total Residual Activity		Total Injected Dose				
Center	Patient	Activity (mCi)	Time Assayed	Activity (mCi)	Time Assayed	Activity (mCi)	Infusion Start Time	Infusion Stop Time	Dose Delivered As Per Protocol	Reason
XVO_001	101	10.7	13:35	0.4	15:28	10.3	15:14	15:24	Yes	
	102	10.2	12:40	0.1	12:56	10.1	12:45	12:55	Yes	
	103	10.6	12:45	0.2	13:03	10.4	12:51	13:01	Yes	
XVO_003	201	9.6	12:59	0.2	13:34	9.4	13:15	13:25	Yes	
XVO_005	301	10.0	11:20	0.1	16:26	9.9	12:09	12:20	Yes	
	302	10.3	10:45	0.1	13:48	10.2	12:03	12:13	Yes	
XVO_007	401	10.2	11:09	0.1	13:55	10.1	12:25	12:36	Yes	
	402	11.1	10:46	0.1	13:04	11.0	12:24	12:34	Yes	

Listing 9
Adverse Events

Center	Patient	AE No [a]	System Organ Class / Preferred Term / Investigator Text	Dates of Onset/ Resolution	CTCAE Grade	Frequency [b]	Action Taken: Study Drug [c]	Action Taken: Other [d]	SAE	Outcome [e]	Relationship [f]
XVO_001	101	1 *	GENERAL DISORDERS AND ADMINISTRATION SITE CONDITIONS/ FATIGUE/ FATIGUE	16NOV2009/ 11DEC2009	1	2	1	1	No	1	1
	102	1 *	GENERAL DISORDERS AND ADMINISTRATION SITE CONDITIONS/ FATIGUE/ FATIGUE	29DEC2009/ CONT	1	2	1	1	No	5	1
		2 *	GASTROINTESTINAL DISORDERS/ CONSTIPATION/ CONSTIPATION	29DEC2009/ CONT	1	1	1	5	No	5	1
XVO_003	201	1 *	INFECTIONS AND INFESTATIONS/ SIALOADENITIS/ MILD BILATERAL SUBMANDIBULAR GLAND TENDERNESS	19NOV2009/ 22DEC2009	1	1	1	1	No	1	1
		2 *	GASTROINTESTINAL DISORDERS/ DIARRHOEA/ DIAHRRHEA	17NOV2009/ CONT	1	1	1	6	No	5	2

Note: [a] * Indicates the AE is treatment emergent.
[b] Frequency: 1=Intermittent 2=Continuous 3=Single Episode
[c] Action Taken, Study Drug: 1=None 2=Dosing stopped permanently due to AE 3=Dosing interrupted
[d] Action Taken, Other: 1=None 2=Procedure or physical therapy 3=Blood or blood products 4=Withdrawn from study due to AE 5=Prescription drug therapy 6=Non-prescription drug therapy 7=Hospitalization 8=IV fluids given 99=Other
[e] Outcome: 1=Resolved 2=resolved with sequelae 3=Death 4=Unknown/lost to follow-up 5=AE persisting 6=AE persisting with change in grade
[f] Relationship to Study Drug: 1=Related 2=Not related

Listing 9
Adverse Events

Center	Patient	AE No [a]	System Organ Class / Preferred Term / Investigator Text	Dates of Onset/ Resolution	CTCAE Grade	Frequency [b]	Action Taken: Study Drug [c]	Action Taken: Other [d]	SAE	Outcome [e]	Relationship [f]
XVO_003	201	3 *	MUSCULOSKELETAL AND CONNECTIVE TISSUE DISORDERS/ BACK PAIN/ LOW BACK PAIN	24NOV2009/ CONT	2	1	1	6	No	5	2
		4 *	INVESTIGATIONS/ BLOOD PRESSURE DECREASED/ DECREASE IN BP	17NOV2009/ 17NOV2009	1	1	1	1	No	1	2
		5 *	SKIN AND SUBCUTANEOUS TISSUE DISORDERS/ SKIN ULCER/ LEFT ELBOW ULCERATION	21NOV2009/ 02DEC2009	1	3	1	1	No	1	2
		6 *	SKIN AND SUBCUTANEOUS TISSUE DISORDERS/ SKIN ULCER/ RIGHT ELBOW ULCERATION	02DEC2009/ 22DEC2009	1	3	1	1	No	1	2
		7 *	RESPIRATORY, THORACIC AND MEDIASTINAL DISORDERS/ NASAL CONGESTION/ NASAL CONGESTION	23NOV2009/ 24NOV2009	1	2	1	6	No	1	2

Note: [a] * Indicates the AE is treatment emergent.
[b] Frequency: 1=Intermittent 2=Continuous 3=Single Episode
[c] Action Taken, Study Drug: 1=None 2=Dosing stopped permanently due to AE 3=Dosing interrupted
[d] Action Taken, Other: 1=None 2=Procedure or physical therapy 3=Blood or blood products 4=Withdrawn from study due to AE 5=Prescription drug therapy 6=Non-prescription drug therapy 7=Hospitalization 8=IV fluids given 99=Other
[e] Outcome: 1=Resolved 2=resolved with sequelae 3=Death 4=Unknown/lost to follow-up 5=AE persisting 6=AE persisting with change in grade
[f] Relationship to Study Drug: 1=Related 2=Not related

Listing 9
Adverse Events

Center	Patient	AE No [a]	System Organ Class / Preferred Term / Investigator Text	Dates of Onset/ Resolution	CTCAE Grade	Frequency [b]	Action Taken: Study Drug [c]	Action Taken: Other [d]	SAE	Outcome [e]	Relationship [f]
XVO_003	201	8 *	INVESTIGATIONS/ GAMMA-GLUTAMYLTRANSFERASE INCREASED/ ELEVATED GGT	23NOV2009/ 02DEC2009	2	3	1	1	No	1	2
XVO_005	301	1 *	GENERAL DISORDERS AND ADMINISTRATION SITE CONDITIONS/ FATIGUE/ FATIGUE	06AUG2009/ 08SEP2009	1	2	1	1	No	1	2
		2 *	RESPIRATORY, THORACIC AND MEDIASTINAL DISORDERS/ WHEEZING/ WHEEZING	15SEP2009/ 22DEC2009	1	2	1	1	No	1	2
	302	1 *	GENERAL DISORDERS AND ADMINISTRATION SITE CONDITIONS/ FATIGUE/ FATIGUE	16DEC2009/ 25JAN2010	1	2	1	1	No	1	1
		2 *	INFECTIONS AND INFESTATIONS/ HORDEOLUM/ STYE RIGHT LOWER EYELID	18DEC2009/ 14JAN2010	1	2	1	1	No	1	2

Note: [a] * Indicates the AE is treatment emergent.
[b] Frequency: 1=Intermittent 2=Continuous 3=Single Episode
[c] Action Taken, Study Drug: 1=None 2=Dosing stopped permanently due to AE 3=Dosing interrupted
[d] Action Taken, Other: 1=None 2=Procedure or physical therapy 3=Blood or blood products 4=Withdrawn from study due to AE 5=Prescription drug therapy 6=Non-prescription drug therapy 7=Hospitalization 8=IV fluids given 99=Other
[e] Outcome: 1=Resolved 2=resolved with sequelae 3=Death 4=Unknown/lost to follow-up 5=AE persisting 6=AE persisting with change in grade
[f] Relationship to Study Drug: 1=Related 2=Not related

Listing 9
Adverse Events

Center	Patient	AE No [a]	System Organ Class / Preferred Term / Investigator Text	Dates of Onset/ Resolution	CTCAE Grade	Frequency [b]	Action Taken: Study Drug [c]	Action Taken: Other [d]	SAE	Outcome [e]	Relationship [f]
XVO_007	401	1 *	SKIN AND SUBCUTANEOUS TISSUE DISORDERS/ ERYTHEMA/ ERTHEMATOUS CHEST	08DEC2009/ 22DEC2009	1	3	1	1	No	1	2
	402	1 *	GENERAL DISORDERS AND ADMINISTRATION SITE CONDITIONS/ OEDEMA PERIPHERAL/ PRETIBIAL EDEMA-LEFT	09JAN2010/ 02FEB2010	1	3	1	1	No	1	2
		2 *	SKIN AND SUBCUTANEOUS TISSUE DISORDERS/ ERYTHEMA/ ERYTHEMA LEFT LEG	09JAN2010/ 02FEB2010	1	3	1	1	No	1	2
		3 *	INFECTIONS AND INFESTATIONS/ ERYSIPELAS/ SUPERFICIAL CELLULITIS LEFT LEG	09JAN2010/ 02FEB2010	1	3	1	5	No	1	2

Note: [a] * Indicates the AE is treatment emergent.
[b] Frequency: 1=Intermittent 2=Continuous 3=Single Episode
[c] Action Taken, Study Drug: 1=None 2=Dosing stopped permanently due to AE 3=Dosing interrupted
[d] Action Taken, Other: 1=None 2=Procedure or physical therapy 3=Blood or blood products 4=Withdrawn from study due to AE 5=Prescription drug therapy 6=Non-prescription drug therapy 7=Hospitalization 8=IV fluids given 99=Other
[e] Outcome: 1=Resolved 2=resolved with sequelae 3=Death 4=Unknown/lost to follow-up 5=AE persisting 6=AE persisting with change in grade
[f] Relationship to Study Drug: 1=Related 2=Not related

Listing 10.1
Laboratory Data: Chemistry

Center	Patient	Visit	Assessment Date	Parameter	Recorded Result	Recorded Units	Converted Result	Converted Units
XVO_001	101	Screening	09NOV2009	ALBUMIN	3.9	G/DL	3900	MG/DL
				ALKALINE PHOSPHATASE	63	IU/L	63	U/L
				ALT (SGPT)	19	IU/L	19	U/L
				AST (SGOT)	23	IU/L	23	U/L
				BICARBONATE	26	MMOL/L	26	MEQ/L
				BUN	13	MG/DL	13	MG/DL
				CALCIUM	9.3	MG/DL	9.3	MG/DL
				CHLORIDE	103	MMOL/L	103	MEQ/L
				CREATININE	0.60	MG/DL	0.60	MG/DL
				GGT	11	IU/L	11	U/L
				GLUCOSE	86	MG/DL	86	MG/DL
				POTASSIUM	3.9	MMOL/L	3.9	MEQ/L
				SODIUM	137	MMOL/L	137	MEQ/L
				TOTAL BILIRUBIN	0.8	MG/DL	0.8	MG/DL
				TOTAL PROTEIN	7.0	GM/DL	7.0	G/DL
		Day 6	16NOV2009	ALBUMIN	3.8	G/DL	3800	MG/DL
				ALKALINE PHOSPHATASE	58	IU/L	58	U/L
				ALT (SGPT)	17	U/L	17	U/L
				AST (SGOT)	23	IU/L	23	U/L
				BICARBONATE	25	MMOL/L	25	MEQ/L
				BUN	10	MG/DL	10	MG/DL
				CALCIUM	9.3	MG/DL	9.3	MG/DL
				CHLORIDE	103	MMOL/L	103	MEQ/L
				CREATININE	0.70	MG/DL	0.70	MG/DL
				GGT	8	IU/L	8	U/L
				GLUCOSE	100	MG/DL	100	MG/DL
				POTASSIUM	4.2	MMOL/L	4.2	MEQ/L
				SODIUM	133	MMOL/L	133	MEQ/L
				TOTAL BILIRUBIN	0.7	MG/DL	0.7	MG/DL
				TOTAL PROTEIN	6.8	GM/DL	6.8	G/DL

Listing 10.1
Laboratory Data: Chemistry

Center	Patient	Visit	Assessment Date	Parameter	Recorded Result	Recorded Units	Converted Result	Converted Units
XVO_001	101	Day 14	24NOV2009	ALBUMIN	3.7	G/DL	3700	MG/DL
				ALKALINE PHOSPHATASE	60	IU/L	60	U/L
				ALT (SGPT)	17	U/L	17	U/L
				AST (SGOT)	21	IU/L	21	U/L
				BICARBONATE	28	MMOL/L	28	MEQ/L
				BUN	14	MG/DL	14	MG/DL
				CALCIUM	9.5	MG/DL	9.5	MG/DL
				CHLORIDE	106	MMOL/L	106	MEQ/L
				CREATININE	0.60	MG/DL	0.60	MG/DL
				GGT	12	IU/L	12	U/L
				GLUCOSE	93	MG/DL	93	MG/DL
				POTASSIUM	4.4	MMOL/L	4.4	MEQ/L
				SODIUM	141	MMOL/L	141	MEQ/L
				TOTAL BILIRUBIN	0.5	MG/DL	0.5	MG/DL
				TOTAL PROTEIN	6.6	GM/DL	6.6	G/DL
		Day 30	11DEC2009	ALBUMIN	3.8	G/DL	3800	MG/DL
				ALKALINE PHOSPHATASE	62	IU/L	62	U/L
				ALT (SGPT)	15	U/L	15	U/L
				AST (SGOT)	19	IU/L	19	U/L
				BICARBONATE	25	MMOL/L	25	MEQ/L
				BUN	14	MG/DL	14	MG/DL
				CALCIUM	9.2	MG/DL	9.2	MG/DL
				CHLORIDE	102	MMOL/L	102	MEQ/L
				CREATININE	0.60	MG/DL	0.60	MG/DL
				GGT	12	IU/L	12	U/L
				GLUCOSE	91	MG/DL	91	MG/DL
				POTASSIUM	4.10	MMOL/L	4.10	MEQ/L
				SODIUM	135	MMOL/L	135	MEQ/L
				TOTAL BILIRUBIN	0.7	MG/DL	0.7	MG/DL
				TOTAL PROTEIN	6.50	GM/DL	6.50	G/DL

Listing 10.1
Laboratory Data: Chemistry

Center	Patient	Visit	Assessment Date	Parameter	Recorded Result	Recorded Units	Converted Result	Converted Units
XVO_001	101	Day 42	05JAN2010	ALBUMIN	4.1	G/DL	4100	MG/DL
				ALKALINE PHOSPHATASE	69	IU/L	69	U/L
				ALT (SGPT)	21	U/L	21	U/L
				AST (SGOT)	24	IU/L	24	U/L
				BICARBONATE	26	MMOL/L	26	MEQ/L
				BUN	12	MG/DL	12	MG/DL
				CALCIUM	9.4	MG/DL	9.4	MG/DL
				CHLORIDE	104	MMOL/L	104	MEQ/L
				CREATININE	.60	MG/DL	.60	MG/DL
				GLUCOSE	99	MG/DL	99	MG/DL
				POTASSIUM	3.9	MMOL/L	3.9	MEQ/L
				SODIUM	136	MMOL/L	136	MEQ/L
				TOTAL BILIRUBIN	0.8	MG/DL	0.8	MG/DL
				TOTAL PROTEIN	7.2	GM/DL	7.2	G/DL
	102	Screening	11DEC2009	ALBUMIN	3.5	G/DL	3500	MG/DL
				ALKALINE PHOSPHATASE	76	U/L	76	U/L
				ALT (SGPT)	27	IU/L	27	U/L
				AST (SGOT)	28	IU/L	28	U/L
				BICARBONATE	28	MMOL/L	28	MEQ/L
				BUN	5	MG/DL	5	MG/DL
				CALCIUM	9.3	MG/DL	9.3	MG/DL
				CHLORIDE	105	MMOL/L	105	MEQ/L
				CREATININE	.90	MG/DL	.90	MG/DL
				GGT	63	IU/L	63	U/L
				GLUCOSE	81	MG/DL	81	MG/DL
				POTASSIUM	3.9	MMOL/L	3.9	MEQ/L
				SODIUM	140	MMOL/L	140	MEQ/L
				TOTAL BILIRUBIN	0.8	MG/DL	0.8	MG/DL
				TOTAL PROTEIN	6.2	GM/DL	6.2	G/DL
		Day 0 Pre-dose	15DEC2009	ALBUMIN	3.5	G/DL	3500	MG/DL

Listing 10.1
Laboratory Data: Chemistry

Center	Patient	Visit	Assessment Date	Parameter	Recorded Result	Recorded Units	Converted Result	Converted Units
XVO_001	102	Day 0 Pre-dose	15DEC2009	ALKALINE PHOSPHATASE	76	IU/L	76	U/L
				ALT (SGPT)	22	U/L	22	U/L
				AST (SGOT)	27	IU/L	27	U/L
				BICARBONATE	27	MMOL/L	27	MEQ/L
				BUN	7	MG/DL	7	MG/DL
				CALCIUM	9.0	MG/DL	9.0	MG/DL
				CHLORIDE	103	MMOL/L	103	MEQ/L
				CREATININE	1.00	MG/DL	1.00	MG/DL
				GGT	51	IU/L	51	U/L
				GLUCOSE	109	MG/DL	109	MG/DL
				POTASSIUM	3.4	MMOL/L	3.4	MEQ/L
				SODIUM	139	MMOL/L	139	MEQ/L
				TOTAL BILIRUBIN	0.9	MG/DL	0.9	MG/DL
				TOTAL PROTEIN	6.1	GM/DL	6.1	G/DL
		Day 6	21DEC2009	ALBUMIN	3.5	G/DL	3500	MG/DL
				ALKALINE PHOSPHATASE	58	IU/L	58	U/L
				ALT (SGPT)	20	U/L	20	U/L
				AST (SGOT)	32	IU/L	32	U/L
				BICARBONATE	30	MMOL/L	30	MEQ/L
				BUN	5	MG/DL	5	MG/DL
				CALCIUM	8.9	MG/DL	8.9	MG/DL
				CHLORIDE	103	MMOL/L	103	MEQ/L
				CREATININE	1.10	MG/DL	1.10	MG/DL
				GGT	43	IU/L	43	U/L
				GLUCOSE	105	MG/DL	105	MG/DL
				POTASSIUM	3.5	MMOL/L	3.5	MEQ/L
				SODIUM	140	MMOL/L	140	MEQ/L
				TOTAL BILIRUBIN	0.8	MG/DL	0.8	MG/DL
				TOTAL PROTEIN	6.5	GM/DL	6.5	G/DL
		Day 14	29DEC2009	ALBUMIN	3.3	G/DL	3300	MG/DL

Listing 10.1
Laboratory Data: Chemistry

Center	Patient	Visit	Assessment Date	Parameter	Recorded Result	Recorded Units	Converted Result	Converted Units
XVO_001	102	Day 14	29DEC2009	ALKALINE PHOSPHATASE	61	IU/L	61	U/L
				ALT (SGPT)	17	U/L	17	U/L
				AST (SGOT)	28	IU/L	28	U/L
				BICARBONATE	30	MMOL/L	30	MEQ/L
				BUN	5	MG/DL	5	MG/DL
				CALCIUM	8.7	MG/DL	8.7	MG/DL
				CHLORIDE	101	MMOL/L	101	MEQ/L
				CREATININE	1.0	MG/DL	1.0	MG/DL
				GGT	34	IU/L	34	U/L
				GLUCOSE	85	MG/DL	85	MG/DL
				POTASSIUM	3.8	MMOL/L	3.8	MEQ/L
				SODIUM	138	MMOL/L	138	MEQ/L
				TOTAL BILIRUBIN	0.6	MG/DL	0.6	MG/DL
				TOTAL PROTEIN	6.2	GM/DL	6.2	G/DL
		Day 30	15JAN2010	ALBUMIN	3.5	G/DL	3500	MG/DL
				ALKALINE PHOSPHATASE	62	IU/L	62	U/L
				ALT (SGPT)	15	U/L	15	U/L
				AST (SGOT)	21	IU/L	21	U/L
				BICARBONATE	29	MMOL/L	29	MEQ/L
				BUN	5	MG/DL	5	MG/DL
				CALCIUM	8.9	MG/DL	8.9	MG/DL
				CHLORIDE	102	MMOL/L	102	MEQ/L
				CREATININE	1.10	MG/DL	1.10	MG/DL
				GGT	32	IU/L	32	U/L
				GLUCOSE	98	MG/DL	98	MG/DL
				POTASSIUM	3.9	MMOL/L	3.9	MEQ/L
				SODIUM	138	MMOL/L	138	MEQ/L
				TOTAL BILIRUBIN	0.7	MG/DL	0.7	MG/DL
				TOTAL PROTEIN	6.5	GM/DL	6.5	G/DL
		Day 42	29JAN2010	ALBUMIN	3.7	G/DL	3700	MG/DL

Listing 10.1
Laboratory Data: Chemistry

Center	Patient	Visit	Assessment Date	Parameter	Recorded Result	Recorded Units	Converted Result	Converted Units
XVO_001	102	Day 42	29JAN2010	ALKALINE PHOSPHATASE	68	IU/L	68	U/L
				ALT (SGPT)	17	U/L	17	U/L
				AST (SGOT)	23	IU/L	23	U/L
				BICARBONATE	29	MMOL/L	29	MEQ/L
				BUN	7	MG/DL	7	MG/DL
				CALCIUM	9.0	MG/DL	9.0	MG/DL
				CHLORIDE	97	MMOL/L	97	MEQ/L
				CREATININE	1.00	MG/DL	1.00	MG/DL
				GGT	24	IU/L	24	U/L
				GLUCOSE	87	MG/DL	87	MG/DL
				POTASSIUM	4.1	MMOL/L	4.1	MEQ/L
				SODIUM	135	MMOL/L	135	MEQ/L
				TOTAL BILIRUBIN	0.8	MG/DL	0.8	MG/DL
				TOTAL PROTEIN	6.7	GM/DL	6.7	G/DL
	103	Screening	11JAN2010	ALBUMIN	3.7	G/DL	3700	MG/DL
				ALKALINE PHOSPHATASE	78	U/L	78	U/L
				ALT (SGPT)	37	IU/L	37	U/L
				AST (SGOT)	20	IU/L	20	U/L
				BICARBONATE	25	MMOL/L	25	MEQ/L
				BUN	18	MG/DL	18	MG/DL
				CALCIUM	9.1	MG/DL	9.1	MG/DL
				CHLORIDE	106	MMOL/L	106	MEQ/L
				CREATININE	1.20	MG/DL	1.20	MG/DL
				GGT	68	IU/L	68	U/L
				GLUCOSE	86	MG/DL	86	MG/DL
				POTASSIUM	4.5	MMOL/L	4.5	MEQ/L
				SODIUM	140	MMOL/L	140	MEQ/L
				TOTAL BILIRUBIN	0.9	MG/DL	0.9	MG/DL
				TOTAL PROTEIN	6.7	GM/DL	6.7	G/DL
		Day 0 Pre-dose	12JAN2010	GGT	68	U/L	68	U/L

Listing 10.1
Laboratory Data: Chemistry

Center	Patient	Visit	Assessment Date	Parameter	Recorded Result	Recorded Units	Converted Result	Converted Units
XVO_001	103	Day 6	19JAN2010	ALBUMIN	3.9	G/DL	3900	MG/DL
				ALKALINE PHOSPHATASE	76	U/L	76	U/L
				ALT (SGPT)	36	IU/L	36	U/L
				AST (SGOT)	23	IU/L	23	U/L
				BICARBONATE	26	MMOL/L	26	MEQ/L
				BUN	17	MG/DL	17	MG/DL
				CALCIUM	9.1	MG/DL	9.1	MG/DL
				CHLORIDE	102	MMOL/L	102	MEQ/L
				CREATININE	1.30	MG/DL	1.30	MG/DL
				GGT	58	IU/L	58	U/L
				GLUCOSE	94	MG/DL	94	MG/DL
				POTASSIUM	4.4	MMOL/L	4.4	MEQ/L
				SODIUM	137	MMOL/L	137	MEQ/L
				TOTAL BILIRUBIN	1.0	MG/DL	1.0	MG/DL
				TOTAL PROTEIN	6.7	GM/DL	6.7	G/DL
		Day 14	26JAN2010	ALBUMIN	3.8	G/DL	3800	MG/DL
				ALKALINE PHOSPHATASE	75	U/L	75	U/L
				ALT (SGPT)	34	IU/L	34	U/L
				AST (SGOT)	24	IU/L	24	U/L
				BICARBONATE	28	MMOL/L	28	MEQ/L
				BUN	14	MG/DL	14	MG/DL
				CALCIUM	9.0	MG/DL	9.0	MG/DL
				CHLORIDE	105	MMOL/L	105	MEQ/L
				CREATININE	1.20	MG/DL	1.20	MG/DL
				GGT	51	IU/L	51	U/L
				GLUCOSE	72	MG/DL	72	MG/DL
				POTASSIUM	4.5	MMOL/L	4.5	MEQ/L
				SODIUM	138	MMOL/L	138	MEQ/L
				TOTAL BILIRUBIN	0.9	MG/DL	0.9	MG/DL
				TOTAL PROTEIN	6.5	GM/DL	6.5	G/DL

Listing 10.1
Laboratory Data: Chemistry

Center	Patient	Visit	Assessment Date	Parameter	Recorded Result	Recorded Units	Converted Result	Converted Units
XVO_001	103	Day 30	09FEB2010	ALBUMIN	4.0	GM/DL	4000	MG/DL
				ALKALINE PHOSPHATASE	77	U/L	77	U/L
				ALT (SGPT)	49	IU/L	49	U/L
				AST (SGOT)	29	IU/L	29	U/L
				BICARBONATE	26	MMOL/L	26	MEQ/L
				BUN	17	MG/DL	17	MG/DL
				CALCIUM	8.9	MG/DL	8.9	MG/DL
				CHLORIDE	101	MMOL/L	101	MEQ/L
				CREATININE	1.30	MG/DL	1.30	MG/DL
				GGT	63	IU/L	63	U/L
				GLUCOSE	91	MG/DL	91	MG/DL
				POTASSIUM	4.2	MMOL/L	4.2	MEQ/L
				SODIUM	137	MMOL/L	137	MEQ/L
				TOTAL BILIRUBIN	1.0	MG/DL	1.0	MG/DL
				TOTAL PROTEIN	7.0	GM/DL	7.0	G/DL
		Day 42	19FEB2010	ALBUMIN	3.7	G/DL	3700	MG/DL
				ALKALINE PHOSPHATASE	76	U/L	76	U/L
				ALT (SGPT)	40	IU/L	40	U/L
				AST (SGOT)	23	IU/L	23	U/L
				BICARBONATE	29	MMOL/L	29	MEQ/L
				BUN	12	MG/DL	12	MG/DL
				CALCIUM	8.7	MG/DL	8.7	MG/DL
				CHLORIDE	102	MMOL/L	102	MEQ/L
				CREATININE	1.10	MG/DL	1.10	MG/DL
				GGT	55	IU/L	55	U/L
				GLUCOSE	102	MG/DL	102	MG/DL
				POTASSIUM	4.4	MMOL/L	4.4	MEQ/L
				SODIUM	139	MMOL/L	139	MEQ/L
				TOTAL BILIRUBIN	0.8	MG/DL	0.8	MG/DL
				TOTAL PROTEIN	6.6	GM/DL	6.6	G/DL

Listing 10.1
Laboratory Data: Chemistry

Center	Patient	Visit	Assessment Date	Parameter	Recorded Result	Recorded Units	Converted Result	Converted Units
XVO_003	201	Screening	10NOV2009	ALBUMIN	4.1	G/DL	4100	MG/DL
				ALKALINE PHOSPHATASE	106	U/L	106	U/L
				ALT (SGPT)	12	U/L	12	U/L
				AST (SGOT)	20	U/L	20	U/L
				BICARBONATE	21	MEQ/L	21	MEQ/L
				BUN	27	MG/DL	27	MG/DL
				CALCIUM	9.5	MG/DL	9.5	MG/DL
				CHLORIDE	103	MEQ/L	103	MEQ/L
				CREATININE	0.9	MG/DL	0.9	MG/DL
				GGT	61	U/L	61	U/L
				GLUCOSE	100	MG/DL	100	MG/DL
				POTASSIUM	4.8	MEQ/L	4.8	MEQ/L
				SODIUM	138	MEQ/L	138	MEQ/L
				TOTAL BILIRUBIN	0.3	MG/DL	0.3	MG/DL
				TOTAL PROTEIN	6.7	G/DL	6.7	G/DL
		Day 0 Pre-dose	17NOV2009	ALBUMIN	4.0	G/DL	4000	MG/DL
				ALKALINE PHOSPHATASE	113	U/L	113	U/L
				ALT (SGPT)	11	U/L	11	U/L
				AST (SGOT)	23	U/L	23	U/L
				BICARBONATE	23	MEQ/L	23	MEQ/L
				BUN	28	MG/DL	28	MG/DL
				CALCIUM	9.4	MG/DL	9.4	MG/DL
				CHLORIDE	100	MEQ/L	100	MEQ/L
				CREATININE	1.0	MG/DL	1.0	MG/DL
				GGT	60	U/L	60	U/L
				GLUCOSE	94	MG/DL	94	MG/DL
				POTASSIUM	4.8	MEQ/L	4.8	MEQ/L
				SODIUM	136	MEQ/L	136	MEQ/L
				TOTAL BILIRUBIN	0.5	MG/DL	0.5	MG/DL
				TOTAL PROTEIN	6.5	G/DL	6.5	G/DL

Listing 10.1
Laboratory Data: Chemistry

Center	Patient	Visit	Assessment Date	Parameter	Recorded Result	Recorded Units	Converted Result	Converted Units
XVO_003	201	Day 6	23NOV2009	ALBUMIN	4.0	G/DL	4000	MG/DL
				ALKALINE PHOSPHATASE	153	U/L	153	U/L
				ALT (SGPT)	18	U/L	18	U/L
				AST (SGOT)	34	U/L	34	U/L
				BICARBONATE	22	MEQ/L	22	MEQ/L
				BUN	27	MG/DL	27	MG/DL
				CALCIUM	8.9	MG/DL	8.9	MG/DL
				CHLORIDE	100	MEQ/L	100	MEQ/L
				CREATININE	1.1	MG/DL	1.1	MG/DL
				GGT	163	U/L	163	U/L
				GLUCOSE	130	MG/DL	130	MG/DL
				POTASSIUM	4.9	MEQ/L	4.9	MEQ/L
				SODIUM	134	MEQ/L	134	MEQ/L
				TOTAL BILIRUBIN	0.4	MG/DL	0.4	MG/DL
				TOTAL PROTEIN	6.8	G/DL	6.8	G/DL
		Day 14	02DEC2009	ALBUMIN	3.8	G/DL	3800	MG/DL
				ALKALINE PHOSPHATASE	106	U/L	106	U/L
				ALT (SGPT)	10	U/L	10	U/L
				AST (SGOT)	23	U/L	23	U/L
				BICARBONATE	22	MEQ/L	22	MEQ/L
				BUN	21	MG/DL	21	MG/DL
				CALCIUM	9.0	MG/DL	9.0	MG/DL
				CHLORIDE	100	MEQ/L	100	MEQ/L
				CREATININE	1.0	MG/DL	1.0	MG/DL
				GGT	74	U/L	74	U/L
				GLUCOSE	102	MG/DL	102	MG/DL
				POTASSIUM	4.4	MEQ/L	4.4	MEQ/L
				SODIUM	134	MEQ/L	134	MEQ/L
				TOTAL BILIRUBIN	0.4	MG/DL	0.4	MG/DL
				TOTAL PROTEIN	6.2	G/DL	6.2	G/DL

Listing 10.1
Laboratory Data: Chemistry

Center	Patient	Visit	Assessment Date	Parameter	Recorded Result	Recorded Units	Converted Result	Converted Units
XVO_003	201	Day 30	22DEC2009	ALBUMIN	4.1	G/DL	4100	MG/DL
				ALKALINE PHOSPHATASE	107	U/L	107	U/L
				ALT (SGPT)	13	U/L	13	U/L
				AST (SGOT)	26	U/L	26	U/L
				BICARBONATE	24	MEQ/L	24	MEQ/L
				BUN	22	MG/DL	22	MG/DL
				CALCIUM	8.8	MG/DL	8.8	MG/DL
				CHLORIDE	102	MEQ/L	102	MEQ/L
				CREATININE	0.8	MG/DL	0.8	MG/DL
				GGT	74	U/L	74	U/L
				GLUCOSE	89	MG/DL	89	MG/DL
				POTASSIUM	4.9	MEQ/L	4.9	MEQ/L
				SODIUM	136	MEQ/L	136	MEQ/L
				TOTAL BILIRUBIN	0.3	MG/DL	0.3	MG/DL
				TOTAL PROTEIN	6.7	G/DL	6.7	G/DL
		Day 42	29DEC2009	ALBUMIN	3.9	G/DL	3900	MG/DL
				ALKALINE PHOSPHATASE	102	U/L	102	U/L
				ALT (SGPT)	11	U/L	11	U/L
				AST (SGOT)	31	U/L	31	U/L
				BICARBONATE	21	MEQ/L	21	MEQ/L
				BUN	27	MG/DL	27	MG/DL
				CALCIUM	8.9	MG/DL	8.9	MG/DL
				CHLORIDE	102	MEQ/L	102	MEQ/L
				CREATININE	1.0	MG/DL	1.0	MG/DL
				GGT	74	U/L	74	U/L
				GLUCOSE	205	MG/DL	205	MG/DL
				POTASSIUM	4.9	MEQ/L	4.9	MEQ/L
				SODIUM	136	MEQ/L	136	MEQ/L
				TOTAL BILIRUBIN	0.4	MG/DL	0.4	MG/DL
				TOTAL PROTEIN	6.6	G/DL	6.6	G/DL

Listing 10.1
Laboratory Data: Chemistry

Center	Patient	Visit	Assessment Date	Parameter	Recorded Result	Recorded Units	Converted Result	Converted Units
XVO_005	301	Screening	03AUG2009	ALBUMIN	3.9	G/DL	3900	MG/DL
				ALKALINE PHOSPHATASE	50	U/L	50	U/L
				ALT (SGPT)	17	U/L	17	U/L
				AST (SGOT)	17	U/L	17	U/L
				BICARBONATE	28	MMOL/L	28	MEQ/L
				BUN	23	MG/DL	23	MG/DL
				CALCIUM	9.4	MG/DL	9.4	MG/DL
				CHLORIDE	102	MMOL/L	102	MEQ/L
				CREATININE	1.2	MG/DL	1.2	MG/DL
				GGT	37	U/L	37	U/L
				GLUCOSE	161	MG/DL	161	MG/DL
				POTASSIUM	4.0	MMOL/L	4.0	MEQ/L
				SODIUM	138	MMOL/L	138	MEQ/L
				TOTAL BILIRUBIN	0.6	MG/DL	0.6	MG/DL
				TOTAL PROTEIN	6.8	G/DL	6.8	G/DL
		Day 0 Pre-dose	04AUG2009	ALBUMIN	3.7	G/DL	3700	MG/DL
				ALKALINE PHOSPHATASE	52	U/L	52	U/L
				ALT (SGPT)	19	U/L	19	U/L
				AST (SGOT)	18	U/L	18	U/L
				BICARBONATE	24	MMOL/L	24	MEQ/L
				BUN	22	MG/DL	22	MG/DL
				CALCIUM	9.3	MG/DL	9.3	MG/DL
				CHLORIDE	103	MMOL/L	103	MEQ/L
				CREATININE	1.1	MG/DL	1.1	MG/DL
				GGT	37	U/L	37	U/L
				GLUCOSE	157	MG/DL	157	MG/DL
				POTASSIUM	4.3	MMOL/L	4.3	MEQ/L
				SODIUM	138	MMOL/L	138	MEQ/L
				TOTAL BILIRUBIN	0.9	MG/DL	0.9	MG/DL
				TOTAL PROTEIN	7.0	G/DL	7.0	G/DL

Listing 10.1
Laboratory Data: Chemistry

Center	Patient	Visit	Assessment Date	Parameter	Recorded Result	Recorded Units	Converted Result	Converted Units
XVO_005	301	Day 6	10AUG2009	ALBUMIN	3.9	G/DL	3900	MG/DL
				ALKALINE PHOSPHATASE	48	U/L	48	U/L
				ALT (SGPT)	16	U/L	16	U/L
				AST (SGOT)	17	U/L	17	U/L
				BICARBONATE	23	MMOL/L	23	MEQ/L
				BUN	22	MG/DL	22	MG/DL
				CALCIUM	9.5	MG/DL	9.5	MG/DL
				CHLORIDE	103	MMOL/L	103	MEQ/L
				CREATININE	1.3	MG/DL	1.3	MG/DL
				GGT	36	U/L	36	U/L
				GLUCOSE	200	MG/DL	200	MG/DL
				POTASSIUM	3.8	MMOL/L	3.8	MEQ/L
				SODIUM	137	MMOL/L	137	MEQ/L
				TOTAL BILIRUBIN	0.4	MG/DL	0.4	MG/DL
				TOTAL PROTEIN	7	G/DL	7	G/DL
		Day 14	18AUG2009	ALBUMIN	3.8	G/DL	3800	MG/DL
				ALKALINE PHOSPHATASE	51	U/L	51	U/L
				ALT (SGPT)	13	U/L	13	U/L
				AST (SGOT)	14	U/L	14	U/L
				BICARBONATE	24	MMOL/L	24	MEQ/L
				BUN	24	MG/DL	24	MG/DL
				CALCIUM	9.3	MG/DL	9.3	MG/DL
				CHLORIDE	101	MMOL/L	101	MEQ/L
				CREATININE	1.2	MG/DL	1.2	MG/DL
				GGT	32	U/L	32	U/L
				GLUCOSE	190	MG/DL	190	MG/DL
				POTASSIUM	4.1	MMOL/L	4.1	MEQ/L
				SODIUM	137	MMOL/L	137	MEQ/L
				TOTAL BILIRUBIN	0.5	MG/DL	0.5	MG/DL
				TOTAL PROTEIN	7	G/DL	7	G/DL

Listing 10.1
Laboratory Data: Chemistry

Center	Patient	Visit	Assessment Date	Parameter	Recorded Result	Recorded Units	Converted Result	Converted Units
XVO_005	301	Day 30	04SEP2009	ALBUMIN	3.9	G/DL	3900	MG/DL
				ALKALINE PHOSPHATASE	61	U/L	61	U/L
				ALT (SGPT)	16	U/L	16	U/L
				AST (SGOT)	17	U/L	17	U/L
				BICARBONATE	30	MMOL/L	30	MEQ/L
				BUN	17	MG/DL	17	MG/DL
				CALCIUM	9.3	MG/DL	9.3	MG/DL
				CHLORIDE	99	MMOL/L	99	MEQ/L
				CREATININE	1.2	MG/DL	1.2	MG/DL
				GGT	40	U/L	40	U/L
				GLUCOSE	144	MG/DL	144	MG/DL
				POTASSIUM	4.1	MMOL/L	4.1	MEQ/L
				SODIUM	138	MMOL/L	138	MEQ/L
				TOTAL BILIRUBIN	0.7	MG/DL	0.7	MG/DL
				TOTAL PROTEIN	7.1	G/DL	7.1	G/DL
		Day 42	15SEP2009	ALBUMIN	3.8	G/DL	3800	MG/DL
				ALKALINE PHOSPHATASE	53	U/L	53	U/L
				ALT (SGPT)	15	U/L	15	U/L
				AST (SGOT)	16	U/L	16	U/L
				BICARBONATE	24	MMOL/L	24	MEQ/L
				BUN	21	MG/DL	21	MG/DL
				CALCIUM	9.4	MG/DL	9.4	MG/DL
				CHLORIDE	102	MMOL/L	102	MEQ/L
				CREATININE	1.1	MG/DL	1.1	MG/DL
				GGT	34	U/L	34	U/L
				GLUCOSE	200	MG/DL	200	MG/DL
				POTASSIUM	4.1	MMOL/L	4.1	MEQ/L
				SODIUM	138	MMOL/L	138	MEQ/L
				TOTAL BILIRUBIN	0.6	MG/DL	0.6	MG/DL
				TOTAL PROTEIN	7.1	G/DL	7.1	G/DL

Listing 10.1
Laboratory Data: Chemistry

Center	Patient	Visit	Assessment Date	Parameter	Recorded Result	Recorded Units	Converted Result	Converted Units
XVO_005	302	Screening	14DEC2009	ALBUMIN	3.8	G/DL	3800	MG/DL
				ALKALINE PHOSPHATASE	177	U/L	177	U/L
				ALT (SGPT)	13	U/L	13	U/L
				AST (SGOT)	22	U/L	22	U/L
				BICARBONATE	23	MMOL/L	23	MEQ/L
				BUN	15	MG/DL	15	MG/DL
				CALCIUM	9.2	MG/DL	9.2	MG/DL
				CHLORIDE	111	MMOL/L	111	MEQ/L
				CREATININE	1.6	MG/DL	1.6	MG/DL
				GGT	18	U/L	18	U/L
				GLUCOSE	128	MG/DL	128	MG/DL
				POTASSIUM	4.0	MMOL/L	4.0	MEQ/L
				SODIUM	140	MMOL/L	140	MEQ/L
				TOTAL BILIRUBIN	0.4	MG/DL	0.4	MG/DL
				TOTAL PROTEIN	6.7	G/DL	6.7	G/DL
		Day 0 Pre-dose	15DEC2009	ALBUMIN	3.7	G/DL	3700	MG/DL
				ALKALINE PHOSPHATASE	183	U/L	183	U/L
				ALT (SGPT)	14	U/L	14	U/L
				AST (SGOT)	25	U/L	25	U/L
				BICARBONATE	21	MMOL/L	21	MEQ/L
				BUN	14	MG/DL	14	MG/DL
				CALCIUM	9.4	MG/DL	9.4	MG/DL
				CHLORIDE	111	MMOL/L	111	MEQ/L
				CREATININE	1.2	MG/DL	1.2	MG/DL
				GGT	22	U/L	22	U/L
				GLUCOSE	102	MG/DL	102	MG/DL
				POTASSIUM	4.2	MMOL/L	4.2	MEQ/L
				SODIUM	138	MMOL/L	138	MEQ/L
				TOTAL BILIRUBIN	0.5	MG/DL	0.5	MG/DL
				TOTAL PROTEIN	6.9	G/DL	6.9	G/DL

Listing 10.1
Laboratory Data: Chemistry

Center	Patient	Visit	Assessment Date	Parameter	Recorded Result	Recorded Units	Converted Result	Converted Units
XVO_005	302	Day 6	21DEC2009	ALBUMIN	3.6	G/DL	3600	MG/DL
				ALKALINE PHOSPHATASE	199	U/L	199	U/L
				ALT (SGPT)	14	U/L	14	U/L
				AST (SGOT)	23	U/L	23	U/L
				BICARBONATE	23	MMOL/L	23	MEQ/L
				BUN	12	MG/DL	12	MG/DL
				CALCIUM	9.4	MG/DL	9.4	MG/DL
				CHLORIDE	111	MMOL/L	111	MEQ/L
				CREATININE	1.3	MG/DL	1.3	MG/DL
				GGT	22	U/L	22	U/L
				GLUCOSE	77	MG/DL	77	MG/DL
				POTASSIUM	3.7	MMOL/L	3.7	MEQ/L
				SODIUM	140	MMOL/L	140	MEQ/L
				TOTAL BILIRUBIN	0.7	MG/DL	0.7	MG/DL
				TOTAL PROTEIN	6.6	G/DL	6.6	G/DL
		Day 14	29DEC2009	ALBUMIN	3.3	G/DL	3300	MG/DL
				ALKALINE PHOSPHATASE	220	U/L	220	U/L
				ALT (SGPT)	11	U/L	11	U/L
				AST (SGOT)	24	U/L	24	U/L
				BICARBONATE	23	MMOL/L	23	MEQ/L
				BUN	13	MG/DL	13	MG/DL
				CALCIUM	9.2	MG/DL	9.2	MG/DL
				CHLORIDE	110	MMOL/L	110	MEQ/L
				CREATININE	1.3	MG/DL	1.3	MG/DL
				GGT	22	U/L	22	U/L
				GLUCOSE	79	MG/DL	79	MG/DL
				POTASSIUM	4	MMOL/L	4	MEQ/L
				SODIUM	139	MMOL/L	139	MEQ/L
				TOTAL BILIRUBIN	0.6	MG/DL	0.6	MG/DL
				TOTAL PROTEIN	6.5	G/DL	6.5	G/DL

Listing 10.1
Laboratory Data: Chemistry

Center	Patient	Visit	Assessment Date	Parameter	Recorded Result	Recorded Units	Converted Result	Converted Units
XVO_005	302	Day 30	15JAN2010	ALBUMIN	4.0	G/DL	4000	MG/DL
				ALKALINE PHOSPHATASE	285.0	U/L	285.0	U/L
				ALT (SGPT)	11.0	U/L	11.0	U/L
				AST (SGOT)	24.0	U/L	24.0	U/L
				BICARBONATE	21.0	MMOL/L	21.0	MEQ/L
				BUN	21	MG/DL	21	MG/DL
				CALCIUM	8.5	MG/DL	8.5	MG/DL
				CHLORIDE	108.0	MMOL/L	108.0	MEQ/L
				CREATININE	1.3	MG/DL	1.3	MG/DL
				GGT	23.0	U/L	23.0	U/L
				GLUCOSE	85.0	MG/DL	85.0	MG/DL
				POTASSIUM	4.1	MMOL/L	4.1	MEQ/L
				SODIUM	134	MMOL/L	134	MEQ/L
				TOTAL BILIRUBIN	0.5	MG/DL	0.5	MG/DL
				TOTAL PROTEIN	7.0	G/DL	7.0	G/DL
		Day 42	25JAN2010	ALBUMIN	3.5	G/DL	3500	MG/DL
				ALKALINE PHOSPHATASE	298	U/L	298	U/L
				ALT (SGPT)	12	U/L	12	U/L
				AST (SGOT)	32	U/L	32	U/L
				BICARBONATE	21	MMOL/L	21	MEQ/L
				BUN	16	MG/DL	16	MG/DL
				CALCIUM	8.8	MG/DL	8.8	MG/DL
				CHLORIDE	112	MMOL/L	112	MEQ/L
				CREATININE	1.2	MG/DL	1.2	MG/DL
				GGT	22	U/L	22	U/L
				GLUCOSE	97	MG/DL	97	MG/DL
				POTASSIUM	5.1	MMOL/L	5.1	MEQ/L
				SODIUM	139	MMOL/L	139	MEQ/L
				TOTAL BILIRUBIN	0.5	MG/DL	0.5	MG/DL
				TOTAL PROTEIN	6.4	G/DL	6.4	G/DL

Listing 10.1
Laboratory Data: Chemistry

Center	Patient	Visit	Assessment Date	Parameter	Recorded Result	Recorded Units	Converted Result	Converted Units
XVO_007	401	Screening	04NOV2009	ALBUMIN	3.7	G/DL	3700	MG/DL
				ALKALINE PHOSPHATASE	85	U/L	85	U/L
				ALT (SGPT)	9	U/L	9	U/L
				AST (SGOT)	25	U/L	25	U/L
				BICARBONATE	29	MEQ/L	29	MEQ/L
				BUN	13	MG/DL	13	MG/DL
				CALCIUM	8.8	MG/DL	8.8	MG/DL
				CHLORIDE	104	MEQ/L	104	MEQ/L
				CREATININE	0.65	MG/DL	0.65	MG/DL
				GGT	20	U/L	20	U/L
				GLUCOSE	122	MG/DL	122	MG/DL
				POTASSIUM	4.0	MEQ/L	4.0	MEQ/L
				SODIUM	139	MEQ/L	139	MEQ/L
				TOTAL BILIRUBIN	0.5	MG/DL	0.5	MG/DL
				TOTAL PROTEIN	7.4	G/DL	7.4	G/DL
		Day 0 Pre-dose	10NOV2009	ALBUMIN	3.8	G/DL	3800	MG/DL
				ALKALINE PHOSPHATASE	89	U/L	89	U/L
				ALT (SGPT)	17	U/L	17	U/L
				AST (SGOT)	27	U/L	27	U/L
				BICARBONATE	31	MEQ/L	31	MEQ/L
				BUN	17	MG/DL	17	MG/DL
				CALCIUM	9.1	MG/DL	9.1	MG/DL
				CHLORIDE	102	MEQ/L	102	MEQ/L
				CREATININE	0.62	MG/DL	0.62	MG/DL
				GGT	22	U/L	22	U/L
				GLUCOSE	102	MG/DL	102	MG/DL
				POTASSIUM	4.2	MEQ/L	4.2	MEQ/L
				SODIUM	139	MEQ/L	139	MEQ/L
				TOTAL BILIRUBIN	0.5	MG/DL	0.5	MG/DL
				TOTAL PROTEIN	7.7	G/DL	7.7	G/DL

Listing 10.1
Laboratory Data: Chemistry

Center	Patient	Visit	Assessment Date	Parameter	Recorded Result	Recorded Units	Converted Result	Converted Units
XVO_007	401	Day 6	16NOV2009	ALBUMIN	3.6	G/DL	3600	MG/DL
				ALKALINE PHOSPHATASE	92	U/L	92	U/L
				ALT (SGPT)	19	U/L	19	U/L
				AST (SGOT)	24	U/L	24	U/L
				BICARBONATE	28	MEQ/L	28	MEQ/L
				BUN	16	MG/DL	16	MG/DL
				CALCIUM	8.8	MG/DL	8.8	MG/DL
				CHLORIDE	105	MEQ/L	105	MEQ/L
				CREATININE	0.6	MG/DL	0.6	MG/DL
				GGT	21	U/L	21	U/L
				GLUCOSE	105	MG/DL	105	MG/DL
				POTASSIUM	4.2	MEQ/L	4.2	MEQ/L
				SODIUM	140	MEQ/L	140	MEQ/L
				TOTAL BILIRUBIN	0.5	MG/DL	0.5	MG/DL
				TOTAL PROTEIN	7.0	G/DL	7.0	G/DL
		Day 14	24NOV2009	ALBUMIN	3.7	G/DL	3700	MG/DL
				ALKALINE PHOSPHATASE	85	U/L	85	U/L
				ALT (SGPT)	15	U/L	15	U/L
				AST (SGOT)	24	U/L	24	U/L
				BICARBONATE	27	MEQ/L	27	MEQ/L
				BUN	16	MG/DL	16	MG/DL
				CALCIUM	8.8	MG/DL	8.8	MG/DL
				CHLORIDE	105	MEQ/L	105	MEQ/L
				CREATININE	0.58	MG/DL	0.58	MG/DL
				GGT	21	U/L	21	U/L
				GLUCOSE	101	MG/DL	101	MG/DL
				POTASSIUM	4.1	MEQ/L	4.1	MEQ/L
				SODIUM	139	MEQ/L	139	MEQ/L
				TOTAL BILIRUBIN	0.5	MG/DL	0.5	MG/DL
				TOTAL PROTEIN	7.3	G/DL	7.3	G/DL

Listing 10.1
Laboratory Data: Chemistry

Center	Patient	Visit	Assessment Date	Parameter	Recorded Result	Recorded Units	Converted Result	Converted Units
XVO_007	401	Day 30	08DEC2009	ALBUMIN	3.8	GM/DL	3800	MG/DL
				ALKALINE PHOSPHATASE	84	U/L	84	U/L
				ALT (SGPT)	8	U/L	8	U/L
				AST (SGOT)	28	U/L	28	U/L
				BICARBONATE	29	MEQ/L	29	MEQ/L
				BUN	16	MG/DL	16	MG/DL
				CALCIUM	9.1	MG/DL	9.1	MG/DL
				CHLORIDE	102	MEQ/L	102	MEQ/L
				CREATININE	0.67	MG/DL	0.67	MG/DL
				GGT	21	U/L	21	U/L
				GLUCOSE	103	MG/DL	103	MG/DL
				POTASSIUM	3.9	MEQ/L	3.9	MEQ/L
				SODIUM	140	MEQ/L	140	MEQ/L
				TOTAL BILIRUBIN	0.6	MG/DL	0.6	MG/DL
				TOTAL PROTEIN	7.5	G/DL	7.5	G/DL
		Day 42	22DEC2009	ALBUMIN	3.7	G/DL	3700	MG/DL
				ALKALINE PHOSPHATASE	82	U/L	82	U/L
				ALT (SGPT)	10	U/L	10	U/L
				AST (SGOT)	22	U/L	22	U/L
				BICARBONATE	28	MEQ/L	28	MEQ/L
				BUN	14	MG/DL	14	MG/DL
				CALCIUM	8.9	MG/DL	8.9	MG/DL
				CHLORIDE	104	MEQ/L	104	MEQ/L
				CREATININE	0.60	MG/DL	0.60	MG/DL
				GGT	18	U/L	18	U/L
				GLUCOSE	86	MG/DL	86	MG/DL
				POTASSIUM	3.7	MEQ/L	3.7	MEQ/L
				SODIUM	137	MEQ/L	137	MEQ/L
				TOTAL BILIRUBIN	0.5	MG/DL	0.5	MG/DL
				TOTAL PROTEIN	7.2	G/DL	7.2	G/DL

Listing 10.1
Laboratory Data: Chemistry

Center	Patient	Visit	Assessment Date	Parameter	Recorded Result	Recorded Units	Converted Result	Converted Units
XVO_007	402	Screening	03DEC2009	ALBUMIN	4.2	G/DL	4200	MG/DL
				ALKALINE PHOSPHATASE	297	U/L	297	U/L
				ALT (SGPT)	16	U/L	16	U/L
				AST (SGOT)	52	U/L	52	U/L
				BICARBONATE	28	MEQ/L	28	MEQ/L
				BUN	20	MG/DL	20	MG/DL
				CALCIUM	9.6	MG/DL	9.6	MG/DL
				CHLORIDE	102	MEQ/L	102	MEQ/L
				CREATININE	0.93	MG/DL	0.93	MG/DL
				GGT	51	U/L	51	U/L
				GLUCOSE	83	MG/DL	83	MG/DL
				POTASSIUM	4.6	MEQ/L	4.6	MEQ/L
				SODIUM	139	MEQ/L	139	MEQ/L
				TOTAL BILIRUBIN	0.2	MG/DL	0.2	MG/DL
				TOTAL PROTEIN	7.9	G/DL	7.9	G/DL
		Day 0 Pre-dose	08DEC2009	ALBUMIN	4.2	G/DL	4200	MG/DL
				ALKALINE PHOSPHATASE	256	U/L	256	U/L
				ALT (SGPT)	29	U/L	29	U/L
				AST (SGOT)	40	U/L	40	U/L
				BICARBONATE	27	MEQ/L	27	MEQ/L
				BUN	28	MG/DL	28	MG/DL
				CALCIUM	10.2	MG/DL	10.2	MG/DL
				CHLORIDE	105	MEQ/L	105	MEQ/L
				CREATININE	0.94	MG/DL	0.94	MG/DL
				GGT	49	U/L	49	U/L
				GLUCOSE	108	MG/DL	108	MG/DL
				POTASSIUM	5.4	MEQ/L	5.4	MEQ/L
				SODIUM	141	MEQ/L	141	MEQ/L
				TOTAL BILIRUBIN	0.5	MG/DL	0.5	MG/DL
				TOTAL PROTEIN	7.4	G/DL	7.4	G/DL

Listing 10.1
Laboratory Data: Chemistry

Center	Patient	Visit	Assessment Date	Parameter	Recorded Result	Recorded Units	Converted Result	Converted Units
XVO_007	402	Day 6	14DEC2009	ALBUMIN	3.9	G/DL	3900	MG/DL
				ALKALINE PHOSPHATASE	171	U/L	171	U/L
				ALT (SGPT)	25	U/L	25	U/L
				AST (SGOT)	43	U/L	43	U/L
				BICARBONATE	25	MEQ/L	25	MEQ/L
				BUN	19	MG/DL	19	MG/DL
				CALCIUM	9.4	MG/DL	9.4	MG/DL
				CHLORIDE	103	MEQ/L	103	MEQ/L
				CREATININE	0.81	MG/DL	0.81	MG/DL
				GGT	43	U/L	43	U/L
				GLUCOSE	111	MG/DL	111	MG/DL
				POTASSIUM	4.6	MEQ/L	4.6	MEQ/L
				SODIUM	137	MEQ/L	137	MEQ/L
				TOTAL BILIRUBIN	0.2	MG/DL	0.2	MG/DL
				TOTAL PROTEIN	7.1	G/DL	7.1	G/DL
		Day 14	22DEC2009	GGT	53	U/L	53	U/L
		Day 30	05JAN2010	GGT	47	U/L	47	U/L
		Day 42	19JAN2010	GGT	32	U/L	32	U/L

Listing 10.2
Laboratory Data: Hematology

Center	Patient	Visit	Assessment Date	Parameter	Recorded Result	Recorded Units	Converted Result	Converted Units
XVO_001	101	Screening	09NOV2009	ANC	3.60	K/UL	3.60	10^3/UL
				BASOPHILS	.5	%	.5	%
				EOSINOPHILS	1.5	%	1.5	%
				HEMATOCRIT(PCV)	40.1	%	40.1	%
				HEMOGLOBIN	13.4	G/DL	13.4	G/DL
				LYMPHOCYTES	30.9	%	30.9	%
				MCH	32.7	PG	32.7	PG
				MCHC	33.5	GM/DL	33.5	PG
				MCV	97.7	FL	97.7	FL
				MONOCYTES	7.3	%	7.3	%
				PLATELETS	209	10^3/UL	209	10^3/UL
				RED BLOOD COUNT	4.11	MILLION/UL	4.11	10^6/UL
				WHITE BLOOD COUNT	6.0	K/UL	6.0	10^3/UL
		Day 6	16NOV2009	ANC	2.70	K/UL	2.70	10^3/UL
				BASOPHILS	0.5	%	0.5	%
				EOSINOPHILS	1.4	%	1.4	%
				HEMATOCRIT(PCV)	38.7	%	38.7	%
				HEMOGLOBIN	13.2	GM/DL	13.2	G/DL
				LYMPHOCYTES	29.4	%	29.4	%
				MCH	32.9	PG	32.9	PG
				MCHC	34.1	GM/DL	34.1	PG
				MCV	96.3	FL	96.3	FL
				MONOCYTES	7.8	%	7.8	%
				PLATELETS	206	K/UL	206	10^3/UL
				RED BLOOD COUNT	4.02	10^6/UL	4.02	10^6/UL
				WHITE BLOOD COUNT	4.5	K/UL	4.5	10^3/UL

Listing 10.2
Laboratory Data: Hematology

Center	Patient	Visit	Assessment Date	Parameter	Recorded Result	Recorded Units	Converted Result	Converted Units
XVO_001	101	Day 14	24NOV2009	ANC	2.80	K/UL	2.80	10^3/UL
				BASOPHILS	0.6	%	0.6	%
				EOSINOPHILS	1.6	%	1.6	%
				HEMATOCRIT(PCV)	37.9	%	37.9	%
				HEMOGLOBIN	13.1	GM/DL	13.1	G/DL
				LYMPHOCYTES	21.7	%	21.7	%
				MCH	33.3	PG	33.3	PG
				MCHC	34.7	GM/DL	34.7	PG
				MCV	95.9	FL	95.9	FL
				MONOCYTES	8.4	%	8.4	%
				PLATELETS	186	K/UL	186	10^3/UL
				RED BLOOD COUNT	3.95	10^6/UL	3.95	10^6/UL
				WHITE BLOOD COUNT	4.1	K/UL	4.1	10^3/UL
		Day 30	11DEC2009	ANC	3.3	K/UL	3.3	10^3/UL
				BASOPHILS	0.4	%	0.4	%
				EOSINOPHILS	0.9	%	0.9	%
				HEMATOCRIT(PCV)	37.8	%	37.8	%
				HEMOGLOBIN	12.9	G/DL	12.9	G/DL
				LYMPHOCYTES	23.9	%	23.9	%
				MCH	33.2	PG	33.2	PG
				MCHC	34.2	GM/DL	34.2	PG
				MCV	97.0	FL	97.0	FL
				MONOCYTES	8.0	%	8.0	%
				PLATELETS	184	KUL	184	10^3/UL
				RED BLOOD COUNT	3.89	10^6/UL	3.89	10^6/UL
				WHITE BLOOD COUNT	5.0	K/UL	5.0	10^3/UL

Listing 10.2
Laboratory Data: Hematology

Center	Patient	Visit	Assessment Date	Parameter	Recorded Result	Recorded Units	Converted Result	Converted Units
XVO_001	101	Day 42	05JAN2010	ANC	3.0	K/UL	3.0	10^3/UL
				BASOPHILS	0.4	%	0.4	%
				EOSINOPHILS	1.4	%	1.4	%
				HEMATOCRIT(PCV)	40.7	%	40.7	%
				HEMOGLOBIN	13.6	GM/DL	13.6	G/DL
				LYMPHOCYTES	25.8	%	25.8	%
				MCH	31.8	PG	31.8	PG
				MCHC	33.3	GM/DL	33.3	PG
				MCV	95.5	FL	95.5	FL
				MONOCYTES	7.2	%	7.2	%
				PLATELETS	192	K/UL	192	10^3/UL
				RED BLOOD COUNT	4.26	10^6/UL	4.26	10^6/UL
				WHITE BLOOD COUNT	4.6	K/UL	4.6	10^3/UL
	102	Screening	11DEC2009	ANC	3.5	K/UL	3.5	10^3/UL
				BASOPHILS	0.5	%	0.5	%
				EOSINOPHILS	5.4	%	5.4	%
				HEMATOCRIT(PCV)	42.9	%	42.9	%
				HEMOGLOBIN	13.8	GM/DL	13.8	G/DL
				LYMPHOCYTES	31.3	%	31.3	%
				MCH	26.6	PG	26.6	PG
				MCHC	32.2	GM/DL	32.2	PG
				MCV	82.6	FL	82.6	FL
				MONOCYTES	9.9	%	9.9	%
				PLATELETS	356	K/UL	356	10^3/UL
				RED BLOOD COUNT	5.19	10^6/UL	5.19	10^6/UL
				WHITE BLOOD COUNT	6.6	K/UL	6.6	10^3/UL

Listing 10.2
Laboratory Data: Hematology

Center	Patient	Visit	Assessment Date	Parameter	Recorded Result	Recorded Units	Converted Result	Converted Units
XVO_001	102	Day 0 Pre-dose	15DEC2009	ANC	4.4	K/UL	4.4	10^3/UL
				BASOPHILS	0.5	%	0.5	%
				EOSINOPHILS	4.2	%	4.2	%
				HEMATOCRIT(PCV)	43.2	%	43.2	%
				HEMOGLOBIN	14.0	GM/DL	14.0	G/DL
				LYMPHOCYTES	24.2	%	24.2	%
				MCH	27.0	PG	27.0	PG
				MCHC	32.5	GM/DL	32.5	PG
				MCV	83.2	FL	83.2	FL
				MONOCYTES	9.7	%	9.7	%
				PLATELETS	375	K/UL	375	10^3/UL
				RED BLOOD COUNT	5.18	10^6/UL	5.18	10^6/UL
				WHITE BLOOD COUNT	7.2	K/UL	7.2	10^3/UL
		Day 6	21DEC2009	ANC	4.9	K/UL	4.9	10^3/UL
				BASOPHILS	0.7	%	0.7	%
				EOSINOPHILS	3.8	%	3.8	%
				HEMATOCRIT(PCV)	41.0	%	41.0	%
				HEMOGLOBIN	13.4	GM/DL	13.4	G/DL
				LYMPHOCYTES	22.9	%	22.9	%
				MCH	27.1	PG	27.1	PG
				MCHC	32.6	GM/DL	32.6	PG
				MCV	83.3	FL	83.3	FL
				MONOCYTES	10.2	%	10.2	%
				PLATELETS	375	K/UL	375	10^3/UL
				RED BLOOD COUNT	4.93	10^6/UL	4.93	10^6/UL
				WHITE BLOOD COUNT	7.8	K/UL	7.8	10^3/UL

Listing 10.2
Laboratory Data: Hematology

Center	Patient	Visit	Assessment Date	Parameter	Recorded Result	Recorded Units	Converted Result	Converted Units
XVO_001	102	Day 14	29DEC2009	ANC	3.5	K/UL	3.5	10^3/UL
				BASOPHILS	0.5	%	0.5	%
				EOSINOPHILS	6.0	%	6.0	%
				HEMATOCRIT(PCV)	42.0	%	42.0	%
				HEMOGLOBIN	13.2	GM/DL	13.2	G/DL
				LYMPHOCYTES	27.7	%	27.7	%
				MCH	25.8	PG	25.8	PG
				MCHC	31.5	GM/DL	31.5	PG
				MCV	81.8	FL	81.8	FL
				MONOCYTES	9.2	%	9.2	%
				PLATELETS	361	K/UL	361	10^3/UL
				RED BLOOD COUNT	5.14	10^6/UL	5.14	10^6/UL
				WHITE BLOOD COUNT	6.2	K/UL	6.2	10^3/UL
		Day 30	15JAN2010	ANC	3.7	K/UL	3.7	10^3/UL
				BASOPHILS	0.4	%	0.4	%
				EOSINOPHILS	3.6	%	3.6	%
				HEMATOCRIT(PCV)	43.0	%	43.0	%
				HEMOGLOBIN	13.6	GM/DL	13.6	G/DL
				LYMPHOCYTES	27.2	%	27.2	%
				MCH	26.0	PG	26.0	PG
				MCHC	31.7	GM/DL	31.7	PG
				MCV	82.0	FL	82.0	FL
				MONOCYTES	8.3	%	8.3	%
				PLATELETS	303	K/UL	303	10^3/UL
				RED BLOOD COUNT	5.24	10^6/UL	5.24	10^6/UL
				WHITE BLOOD COUNT	6.1	K/UL	6.1	10^3/UL

Listing 10.2
Laboratory Data: Hematology

Center	Patient	Visit	Assessment Date	Parameter	Recorded Result	Recorded Units	Converted Result	Converted Units
XVO_001	102	Day 42	29JAN2010	ANC	3.5	K/UL	3.5	10^3/UL
				BASOPHILS	0.7	%	0.7	%
				EOSINOPHILS	3.6	%	3.6	%
				HEMATOCRIT(PCV)	43.8	%	43.8	%
				HEMOGLOBIN	13.8	GM/DL	13.8	G/DL
				LYMPHOCYTES	28.5	%	28.5	%
				MCH	26.0	PG	26.0	PG
				MCHC	31.6	GM/DL	31.6	PG
				MCV	82.3	FL	82.3	FL
				MONOCYTES	8.4	%	8.4	%
				PLATELETS	289	K/UL	289	10^3/UL
				RED BLOOD COUNT	5.33	10^6/UL	5.33	10^6/UL
				WHITE BLOOD COUNT	5.9	K/UL	5.9	10^3/UL
	103	Screening	11JAN2010	ANC	4.1	K/UL	4.1	10^3/UL
				BASOPHILS	1.5	%	1.5	%
				EOSINOPHILS	4.7	%	4.7	%
				HEMATOCRIT(PCV)	42.8	%	42.8	%
				HEMOGLOBIN	15.0	GM/DL	15.0	G/DL
				LYMPHOCYTES	33.1	%	33.1	%
				MCH	32.6	PG	32.6	PG
				MCHC	35.1	GM/DL	35.1	PG
				MCV	92.8	FL	92.8	FL
				MONOCYTES	10.5	%	10.5	%
				PLATELETS	277	K/UL	277	10^3/UL
				RED BLOOD COUNT	4.61	10^6/UL	4.61	10^6/UL
				WHITE BLOOD COUNT	8.2	K/UL	8.2	10^3/UL

Listing 10.2
Laboratory Data: Hematology

Center	Patient	Visit	Assessment Date	Parameter	Recorded Result	Recorded Units	Converted Result	Converted Units
XVO_001	103	Day 6	19JAN2010	ANC	4.2	K/UL	4.2	10^3/UL
				BASOPHILS	1.4	%	1.4	%
				EOSINOPHILS	4.8	%	4.8	%
				HEMATOCRIT(PCV)	44.5	%	44.5	%
				HEMOGLOBIN	14.8	GM/DL	14.8	G/DL
				LYMPHOCYTES	29.9	%	29.9	%
				MCH	31.1	PG	31.1	PG
				MCHC	33.3	GM/DL	33.3	PG
				MCV	93.4	FL	93.4	FL
				MONOCYTES	8.0	%	8.0	%
				PLATELETS	297	K/UL	297	10^3/UL
				RED BLOOD COUNT	4.76	10^6/UL	4.76	10^6/UL
				WHITE BLOOD COUNT	7.6	K/UL	7.6	10^3/UL
		Day 14	26JAN2010	ANC	4.1	K/UL	4.1	10^3/UL
				BASOPHILS	0.6	%	0.6	%
				EOSINOPHILS	4.3	%	4.3	%
				HEMATOCRIT(PCV)	42.5	%	42.5	%
				HEMOGLOBIN	14.2	GM/DL	14.2	G/DL
				LYMPHOCYTES	27.6	%	27.6	%
				MCH	31.4	PG	31.4	PG
				MCHC	33.5	GM/DL	33.5	PG
				MCV	93.9	FL	93.9	FL
				MONOCYTES	9.8	%	9.8	%
				PLATELETS	293	K/UL	293	10^3/UL
				RED BLOOD COUNT	4.53	10^6/UL	4.53	10^6/UL
				WHITE BLOOD COUNT	7.2	K/UL	7.2	10^3/UL

Listing 10.2
Laboratory Data: Hematology

Center	Patient	Visit	Assessment Date	Parameter	Recorded Result	Recorded Units	Converted Result	Converted Units
XVO_001	103	Day 30	09FEB2010	ANC	4.3	K/UL	4.3	10^3/UL
				BASOPHILS	1.4	%	1.4	%
				EOSINOPHILS	5.7	%	5.7	%
				HEMATOCRIT(PCV)	44.9	%	44.9	%
				HEMOGLOBIN	14.6	GM/DL	14.6	G/DL
				LYMPHOCYTES	31.4	%	31.4	%
				MCH	30.8	PG	30.8	PG
				MCHC	32.6	GM/DL	32.6	PG
				MCV	94.5	FL	94.5	FL
				MONOCYTES	10.6	%	10.6	%
				PLATELETS	261	K/UL	261	10^3/UL
				RED BLOOD COUNT	4.74	10^6/UL	4.74	10^6/UL
				WHITE BLOOD COUNT	8.4	K/UL	8.4	10^3/UL
		Day 42	19FEB2010	ANC	3.5	K/UL	3.5	10^3/UL
				BASOPHILS	1.4	%	1.4	%
				EOSINOPHILS	6.9	%	6.9	%
				HEMATOCRIT(PCV)	44.0	%	44.0	%
				HEMOGLOBIN	14.6	GM/DL	14.6	G/DL
				LYMPHOCYTES	26.5	%	26.5	%
				MCH	31.6	PG	31.6	PG
				MCHC	33.2	GM/DL	33.2	PG
				MCV	95.2	FL	95.2	FL
				MONOCYTES	8.3	%	8.3	%
				PLATELETS	226	K/UL	226	10^3/UL
				RED BLOOD COUNT	4.62	10^6/UL	4.62	10^6/UL
				WHITE BLOOD COUNT	6.1	K/UL	6.1	10^3/UL

Listing 10.2
Laboratory Data: Hematology

Center	Patient	Visit	Assessment Date	Parameter	Recorded Result	Recorded Units	Converted Result	Converted Units
XVO_003	201	Screening	10NOV2009	ANC	4350	/CU MM	4.35	10^3/UL
				BASOPHILS	0.9	%	0.9	%
				EOSINOPHILS	0.9	%	0.9	%
				HEMATOCRIT(PCV)	38.1	%	38.1	%
				HEMOGLOBIN	12.6	G/DL	12.6	G/DL
				LYMPHOCYTES	19.0	%	19.0	%
				MCH	28.1	PG	28.1	PG
				MCHC	33.1	G/DL	33.1	PG
				MCV	84.9	FL	84.9	FL
				MONOCYTES	15.7	%	15.7	%
				PLATELETS	280	10^3/CU MM	280	10^3/UL
				RED BLOOD COUNT	4.49	10^6/CU MM	4.49	10^6/UL
				WHITE BLOOD COUNT	6860	/CU MM	6.86	10^3/UL
		Day 0 Pre-dose	17NOV2009	ANC	3440	/CU MM	3.44	10^3/UL
				BASOPHILS	0.8	%	0.8	%
				EOSINOPHILS	4.2	%	4.2	%
				HEMATOCRIT(PCV)	36.3	%	36.3	%
				HEMOGLOBIN	12.1	G/DL	12.1	G/DL
				LYMPHOCYTES	20.7	%	20.7	%
				MCH	28.2	PG	28.2	PG
				MCHC	33.3	G/DL	33.3	PG
				MCV	84.6	FL	84.6	FL
				MONOCYTES	15.6	%	15.6	%
				PLATELETS	282	K/CU MM	282	10^3/UL
				RED BLOOD COUNT	4.29	M/CU MM	4.29	10^6/UL
				WHITE BLOOD COUNT	5890	/CU MM	5.89	10^3/UL

Listing 10.2
Laboratory Data: Hematology

Center	Patient	Visit	Assessment Date	Parameter	Recorded Result	Recorded Units	Converted Result	Converted Units
XVO_003	201	Day 6	23NOV2009	ANC	6490	/CU MM	6.49	10^3/UL
				BASOPHILS	0.4	%	0.4	%
				EOSINOPHILS	1.1	%	1.1	%
				HEMATOCRIT(PCV)	35.9	%	35.9	%
				HEMOGLOBIN	12.2	G/DL	12.2	G/DL
				LYMPHOCYTES	7.3	%	7.3	%
				MCH	28.8	PG	28.8	PG
				MCHC	34.0	G/DL	34.0	PG
				MCV	84.7	FL	84.7	FL
				MONOCYTES	13.6	%	13.6	%
				PLATELETS	272	K/CU MM	272	10^3/UL
				RED BLOOD COUNT	4.24	M/CU MM	4.24	10^6/UL
				WHITE BLOOD COUNT	8390	/CU MM	8.39	10^3/UL
		Day 14	02DEC2009	ANC	3360	/CU MM	3.36	10^3/UL
				BASOPHILS	1.0	%	1.0	%
				EOSINOPHILS	2.9	%	2.9	%
				HEMATOCRIT(PCV)	34.6	%	34.6	%
				HEMOGLOBIN	11.6	G/DL	11.6	G/DL
				LYMPHOCYTES	14.8	%	14.8	%
				MCH	28.7	PG	28.7	PG
				MCHC	33.5	G/DL	33.5	PG
				MCV	85.6	FL	85.6	FL
				MONOCYTES	24.5	%	24.5	%
				PLATELETS	317	K/CU MM	317	10^3/UL
				RED BLOOD COUNT	4.04	M/CU MM^3	4.04	10^6/UL
				WHITE BLOOD COUNT	5960	/CU MM	5.96	10^3/UL

Listing 10.2
Laboratory Data: Hematology

Center	Patient	Visit	Assessment Date	Parameter	Recorded Result	Recorded Units	Converted Result	Converted Units
XVO_003	201	Day 30	22DEC2009	ANC	3120	/CU MM	3.12	10^3/UL
				BASOPHILS	1.4	%	1.4	%
				EOSINOPHILS	2.8	%	2.8	%
				HEMATOCRIT(PCV)	38.2	%	38.2	%
				HEMOGLOBIN	12.7	G/DL	12.7	G/DL
				LYMPHOCYTES	15.7	%	15.7	%
				MCH	28.3	PG	28.3	PG
				MCHC	33.2	G/DL	33.2	PG
				MCV	85.1	FL	85.1	FL
				MONOCYTES	18.5	%	18.5	%
				PLATELETS	232	K/CU MM	232	10^3/UL
				RED BLOOD COUNT	4.49	M/CU MM	4.49	10^6/UL
				WHITE BLOOD COUNT	5090	/CU MM	5.09	10^3/UL
		Day 42	29DEC2009	ANC	5380	/CU MM	5.38	10^3/UL
				BASOPHILS	0.7	%	0.7	%
				EOSINOPHILS	1.3	%	1.3	%
				HEMATOCRIT(PCV)	37.3	%	37.3	%
				HEMOGLOBIN	12.5	G/DL	12.5	G/DL
				LYMPHOCYTES	10.8	%	10.8	%
				MCH	28.3	PG	28.3	PG
				MCHC	33.5	G/DL	33.5	PG
				MCV	84.4	FL	84.4	FL
				MONOCYTES	10.7	%	10.7	%
				PLATELETS	228	K/CU MM	228	10^3/UL
				RED BLOOD COUNT	4.42	M/CU MM	4.42	10^6/UL
				WHITE BLOOD COUNT	7040	/CU MM	7.04	10^3/UL

Listing 10.2
Laboratory Data: Hematology

Center	Patient	Visit	Assessment Date	Parameter	Recorded Result	Recorded Units	Converted Result	Converted Units
XVO_005	301	Screening	03AUG2009	ANC	4.7	X10^9	4.7	10^3/UL
				BASOPHILS	0.6	%	0.6	%
				EOSINOPHILS	6.0	%	6.0	%
				HEMATOCRIT(PCV)	0.40	L/L	40	%
				HEMOGLOBIN	13.7	G/DL	13.7	G/DL
				LYMPHOCYTES	19.7	%	19.7	%
				MCH	29.3	PG	29.3	PG
				MCHC	33.9	%	33.9	PG
				MCV	86	FL	86	FL
				MONOCYTES	7.0	%	7.0	%
				PLATELETS	185	X10^9	185	10^3/UL
				RED BLOOD COUNT	4.68	X10^12	4.68	10^6/UL
				WHITE BLOOD COUNT	7.0	X10^9	7.0	10^3/UL
		Day 0 Pre-dose	04AUG2009	ANC	4.5	X10^9	4.5	10^3/UL
				BASOPHILS	0.5	%	0.5	%
				EOSINOPHILS	6.8	%	6.8	%
				HEMATOCRIT(PCV)	0.4	L/L	40	%
				HEMOGLOBIN	13.8	G/DL	13.8	G/DL
				LYMPHOCYTES	15.5	%	15.5	%
				MCH	29	PG	29	PG
				MCHC	34.5	%	34.5	PG
				MCV	84	FL	84	FL
				MONOCYTES	7.7	%	7.7	%
				PLATELETS	169	X10^9	169	10^3/UL
				RED BLOOD COUNT	4.76	X10^12	4.76	10^6/UL
				WHITE BLOOD COUNT	6.5	X10^9	6.5	10^3/UL

Listing 10.2
Laboratory Data: Hematology

Center	Patient	Visit	Assessment Date	Parameter	Recorded Result	Recorded Units	Converted Result	Converted Units
XVO_005	301	Day 6	10AUG2009	ANC	6.2	X10^9	6.2	10^3/UL
				BASOPHILS	0.5	%	0.5	%
				EOSINOPHILS	6.2	%	6.2	%
				HEMATOCRIT(PCV)	0.42	L/L	42	%
				HEMOGLOBIN	13.8	G/DL	13.8	G/DL
				LYMPHOCYTES	12.6	%	12.6	%
				MCH	28.7	PG	28.7	PG
				MCHC	33.1	%	33.1	PG
				MCV	87	FL	87	FL
				MONOCYTES	7.3	%	7.3	%
				PLATELETS	197	X10^9	197	10^3/UL
				RED BLOOD COUNT	4.81	X10^12	4.81	10^6/UL
				WHITE BLOOD COUNT	8.5	X10^9	8.5	10^3/UL
		Day 14	18AUG2009	ANC	5.2	X10^9	5.2	10^3/UL
				BASOPHILS	0.7	%	0.7	%
				EOSINOPHILS	6.2	%	6.2	%
				HEMATOCRIT(PCV)	0.40	L/L	40	%
				HEMOGLOBIN	13.5	G/DL	13.5	G/DL
				LYMPHOCYTES	15.1	%	15.1	%
				MCH	29.1	PG	29.1	PG
				MCHC	33.7	%	33.7	PG
				MCV	86	FL	86	FL
				MONOCYTES	7.4	%	7.4	%
				PLATELETS	190	X10^9	190	10^3/UL
				RED BLOOD COUNT	4.64	X10^12	4.64	10^6/UL
				WHITE BLOOD COUNT	7.4	X10^9	7.4	10^3/UL

Listing 10.2
Laboratory Data: Hematology

Center	Patient	Visit	Assessment Date	Parameter	Recorded Result	Recorded Units	Converted Result	Converted Units
XVO_005	301	Day 42	15SEP2009	ANC	5.6	X10^9	5.6	10^3/UL
				BASOPHILS	0.3	%	0.3	%
				EOSINOPHILS	6.2	%	6.2	%
				HEMATOCRIT(PCV)	0.40	L/L	40	%
				HEMOGLOBIN	13.6	G/DL	13.6	G/DL
				LYMPHOCYTES	15.4	%	15.4	%
				MCH	29.4	PG	29.4	PG
				MCHC	34.2	%	34.2	PG
				MCV	86	FL	86	FL
				MONOCYTES	7.6	%	7.6	%
				PLATELETS	210	X10^9	210	10^3/UL
				RED BLOOD COUNT	4.62	X10^12	4.62	10^6/UL
				WHITE BLOOD COUNT	7.9	X10^9	7.9	10^3/UL
	302	Screening	14DEC2009	ANC	7	X10^9	7	10^3/UL
				BASOPHILS	0.4	%	0.4	%
				EOSINOPHILS	6.9	%	6.9	%
				HEMATOCRIT(PCV)	0.36	L/L	36	%
				HEMOGLOBIN	12	G/DL	12	G/DL
				LYMPHOCYTES	20.5	%	20.5	%
				MCH	30.2	PG	30.2	PG
				MCHC	33	%	33	PG
				MCV	92	FL	92	FL
				MONOCYTES	6.2	%	6.2	%
				PLATELETS	266	X10^9	266	10^3/UL
				RED BLOOD COUNT	3.97	X10^12	3.97	10^6/UL
				WHITE BLOOD COUNT	10.7	X10^9	10.7	10^3/UL

Listing 10.2
Laboratory Data: Hematology

Center	Patient	Visit	Assessment Date	Parameter	Recorded Result	Recorded Units	Converted Result	Converted Units
XVO_005	302	Day 0 Pre-dose	15DEC2009	ANC	8.7	X10^9	8.7	10^3/UL
				BASOPHILS	0.3	%	0.3	%
				EOSINOPHILS	6.6	%	6.6	%
				HEMATOCRIT(PCV)	0.39	L/L	39	%
				HEMOGLOBIN	12.8	G/DL	12.8	G/DL
				LYMPHOCYTES	16	%	16	%
				MCH	29.9	PG	29.9	PG
				MCHC	14.2	%	14.2	PG
				MCV	91	FL	91	FL
				MONOCYTES	8.1	%	8.1	%
				PLATELETS	242	X10^9	242	10^3/UL
				RED BLOOD COUNT	4.28	X10^12	4.28	10^6/UL
				WHITE BLOOD COUNT	12.6	X10^9	12.6	10^3/UL
		Day 6	21DEC2009	ANC	6.3	X10^9	6.3	10^3/UL
				BASOPHILS	0.6	%	0.6	%
				EOSINOPHILS	7.3	%	7.3	%
				HEMATOCRIT(PCV)	0.34	L/L	34	%
				HEMOGLOBIN	11.5	G/DL	11.5	G/DL
				LYMPHOCYTES	19	%	19	%
				MCH	30	PG	30	PG
				MCHC	33.4	%	33.4	PG
				MCV	90	FL	90	FL
				MONOCYTES	8.2	%	8.2	%
				PLATELETS	332	X10^9	332	10^3/UL
				RED BLOOD COUNT	3.83	X10^12	3.83	10^6/UL
				WHITE BLOOD COUNT	9.7	X10^9	9.7	10^3/UL

Listing 10.2
Laboratory Data: Hematology

Center	Patient	Visit	Assessment Date	Parameter	Recorded Result	Recorded Units	Converted Result	Converted Units
XVO_005	302	Day 14	29DEC2009	ANC	6	X10^9	6	10^3/UL
				BASOPHILS	0.8	%	0.8	%
				EOSINOPHILS	7.9	%	7.9	%
				HEMATOCRIT(PCV)	0.35	L/L	35	%
				HEMOGLOBIN	11.4	G/DL	11.4	G/DL
				LYMPHOCYTES	16.9	%	16.9	%
				MCH	29.7	PG	29.7	PG
				MCHC	33	%	33	PG
				MCV	90	FL	90	FL
				MONOCYTES	9.4	%	9.4	%
				PLATELETS	338	X10^9	338	10^3/UL
				RED BLOOD COUNT	3.84	X10^12	3.84	10^6/UL
				WHITE BLOOD COUNT	9.2	X10^9	9.2	10^3/UL
		Day 30	15JAN2010	ANC	6.3	X10^9	6.3	10^3/UL
				BASOPHILS	0.4	%	0.4	%
				EOSINOPHILS	6.2	%	6.2	%
				HEMATOCRIT(PCV)	0.33	L/L	33	%
				HEMOGLOBIN	11.1	G/DL	11.1	G/DL
				LYMPHOCYTES	18.2	%	18.2	%
				MCH	29.8	PG	29.8	PG
				MCHC	33.6	%	33.6	PG
				MCV	89.0	FL	89.0	FL
				MONOCYTES	12.2	%	12.2	%
				PLATELETS	225.0	X10^9	225.0	10^3/UL
				RED BLOOD COUNT	3.72	X10^12	3.72	10^6/UL
				WHITE BLOOD COUNT	10.0	X10^9	10.0	10^3/UL

Listing 10.2
Laboratory Data: Hematology

Center	Patient	Visit	Assessment Date	Parameter	Recorded Result	Recorded Units	Converted Result	Converted Units
XVO_005	302	Day 42	25JAN2010	ANC	6.0	X10^9	6.0	10^3/UL
				BASOPHILS	0.4	%	0.4	%
				EOSINOPHILS	7.3	%	7.3	%
				HEMATOCRIT(PCV)	0.33	L/L	33	%
				HEMOGLOBIN	10.8	G/DL	10.8	G/DL
				LYMPHOCYTES	19.4	%	19.4	%
				MCH	29.4	PG	29.4	PG
				MCHC	32.6	%	32.6	PG
				MCV	90	FL	90	FL
				MONOCYTES	6.7	%	6.7	%
				PLATELETS	258	10^3/UL	258	10^3/UL
				RED BLOOD COUNT	3.67	X10^12	3.67	10^6/UL
				WHITE BLOOD COUNT	9.1	X10^9	9.1	10^3/UL
XVO_007	401	Screening	04NOV2009	ANC	2.2	K/UL	2.2	10^3/UL
				BASOPHILS	0.3	%	0.3	%
				EOSINOPHILS	2.9	%	2.9	%
				HEMATOCRIT(PCV)	38.3	%	38.3	%
				HEMOGLOBIN	13.1	G/DL	13.1	G/DL
				LYMPHOCYTES	37.6	%	37.6	%
				MCH	31.9	PG	31.9	PG
				MCHC	34.3	PG	34.3	PG
				MCV	92.9	FL	92.9	FL
				MONOCYTES	7.4	%	7.4	%
				PLATELETS	202	K/UL	202	10^3/UL
				RED BLOOD COUNT	4.12	M/UL	4.12	10^6/UL
				WHITE BLOOD COUNT	4.3	K/UL	4.3	10^3/UL

Listing 10.2
Laboratory Data: Hematology

Center	Patient	Visit	Assessment Date	Parameter	Recorded Result	Recorded Units	Converted Result	Converted Units
XVO_007	401	Day 0 Pre-dose	10NOV2009	ANC	2.2	K/UL	2.2	10^3/UL
				BASOPHILS	0.7	%	0.7	%
				EOSINOPHILS	3.7	%	3.7	%
				HEMATOCRIT(PCV)	40.0	%	40.0	%
				HEMOGLOBIN	13.6	G/DL	13.6	G/DL
				LYMPHOCYTES	39.2	%	39.2	%
				MCH	31.7	PG	31.7	PG
				MCHC	33.9	PG	33.9	PG
				MCV	93.6	FL	93.6	FL
				MONOCYTES	8.4	%	8.4	%
				PLATELETS	221	K/UL	221	10^3/UL
				RED BLOOD COUNT	4.27	M/UL	4.27	10^6/UL
				WHITE BLOOD COUNT	4.6	K/UL	4.6	10^3/UL
		Day 6	16NOV2009	ANC	2.4	K/UL	2.4	10^3/UL
				BASOPHILS	0.6	%	0.6	%
				EOSINOPHILS	4.7	%	4.7	%
				HEMATOCRIT(PCV)	37.9	%	37.9	%
				HEMOGLOBIN	12.9	G/DL	12.9	G/DL
				LYMPHOCYTES	30.9	%	30.9	%
				MCH	31.4	PG	31.4	PG
				MCHC	34.1	PG	34.1	PG
				MCV	92.1	FL	92.1	FL
				MONOCYTES	8.0	%	8.0	%
				PLATELETS	204	K/UL	204	10^3/UL
				RED BLOOD COUNT	4.11	M/UL	4.11	10^6/UL
				WHITE BLOOD COUNT	4.3	K/UL	4.3	10^3/UL

Listing 10.2
Laboratory Data: Hematology

Center	Patient	Visit	Assessment Date	Parameter	Recorded Result	Recorded Units	Converted Result	Converted Units
XVO_007	401	Day 14	24NOV2009	ANC	2.6	K/UL	2.6	10^3/UL
				BASOPHILS	0.2	%	0.2	%
				EOSINOPHILS	2.7	%	2.7	%
				HEMATOCRIT(PCV)	37.7	%	37.7	%
				HEMOGLOBIN	13.2	G/DL	13.2	G/DL
				LYMPHOCYTES	28.1	%	28.1	%
				MCH	32.4	PG	32.4	PG
				MCHC	34.9	PG	34.9	PG
				MCV	92.6	FL	92.6	FL
				MONOCYTES	7.3	%	7.3	%
				PLATELETS	209	K/UL	209	10^3/UL
				RED BLOOD COUNT	4.07	M/UL	4.07	10^6/UL
				WHITE BLOOD COUNT	4.2	K/UL	4.2	10^3/UL
		Day 30	08DEC2009	ANC	2.0	K/UL	2.0	10^3/UL
				BASOPHILS	0.7	%	0.7	%
				EOSINOPHILS	3.5	%	3.5	%
				HEMATOCRIT(PCV)	40.0	%	40.0	%
				HEMOGLOBIN	13.7	G/DL	13.7	G/DL
				LYMPHOCYTES	32.6	%	32.6	%
				MCH	32.2	PG	32.2	PG
				MCHC	34.3	PG	34.3	PG
				MCV	94.0	FL	94.0	FL
				MONOCYTES	8.1	%	8.1	%
				PLATELETS	198	K/UL	198	10^3/UL
				RED BLOOD COUNT	4.25	M/UL	4.25	10^6/UL
				WHITE BLOOD COUNT	3.7	K/UL	3.7	10^3/UL

Listing 10.2
Laboratory Data: Hematology

Center	Patient	Visit	Assessment Date	Parameter	Recorded Result	Recorded Units	Converted Result	Converted Units
XVO_007	401	Day 42	22DEC2009	ANC	2.8	K/UL	2.8	10^3/UL
				BASOPHILS	0.5	%	0.5	%
				EOSINOPHILS	1.4	%	1.4	%
				HEMATOCRIT(PCV)	38.6	%	38.6	%
				HEMOGLOBIN	13.3	G/DL	13.3	G/DL
				LYMPHOCYTES	24.5	%	24.5	%
				MCH	32.6	PG	32.6	PG
				MCHC	34.6	PG	34.6	PG
				MCV	94.2	FL	94.2	FL
				MONOCYTES	8.5	%	8.5	%
				PLATELETS	194	K/UL	194	10^3/UL
				RED BLOOD COUNT	4.09	M/UL	4.09	10^6/UL
				WHITE BLOOD COUNT	4.4	K/UL	4.4	10^3/UL
	402	Screening	03DEC2009	ANC	3.2	K/UL	3.2	10^3/UL
				BASOPHILS	0.5	%	0.5	%
				EOSINOPHILS	1.5	%	1.5	%
				HEMATOCRIT(PCV)	41.1	%	41.1	%
				HEMOGLOBIN	13.6	G/DL	13.6	G/DL
				LYMPHOCYTES	31.3	%	31.3	%
				MCH	29.3	PG	29.3	PG
				MCHC	33.0	PG	33.0	PG
				MCV	88.9	FL	88.9	FL
				MONOCYTES	8.6	%	8.6	%
				PLATELETS	341	K/UL	341	10^3/UL
				RED BLOOD COUNT	4.62	M/UL	4.62	10^6/UL
				WHITE BLOOD COUNT	5.6	K/UL	5.6	10^3/UL

Listing 10.2
Laboratory Data: Hematology

Center	Patient	Visit	Assessment Date	Parameter	Recorded Result	Recorded Units	Converted Result	Converted Units
XVO_007	402	Day 0 Pre-dose	08DEC2009	ANC	7.8	K/UL	7.8	10^3/UL
				BASOPHILS	0.3	%	0.3	%
				EOSINOPHILS	1.5	%	1.5	%
				HEMATOCRIT(PCV)	43.2	%	43.2	%
				HEMOGLOBIN	14.6	G/DL	14.6	G/DL
				LYMPHOCYTES	19.2	%	19.2	%
				MCH	30.1	PG	30.1	PG
				MCHC	33.8	PG	33.8	PG
				MCV	89.1	FL	89.1	FL
				MONOCYTES	5.8	%	5.8	%
				PLATELETS	317	K/UL	317	10^3/UL
				RED BLOOD COUNT	4.85	M/UL	4.85	10^6/UL
				WHITE BLOOD COUNT	10.7	K/UL	10.7	10^3/UL
		Day 6	14DEC2009	ANC	4.7	K/UL	4.7	10^3/UL
				BASOPHILS	0.6	%	0.6	%
				EOSINOPHILS	0.9	%	0.9	%
				HEMATOCRIT(PCV)	38.0	%	38.0	%
				HEMOGLOBIN	12.8	G/DL	12.8	G/DL
				LYMPHOCYTES	21.7	%	21.7	%
				MCH	29.7	PG	29.7	PG
				MCHC	33.8	PG	33.8	PG
				MCV	87.7	FL	87.7	FL
				MONOCYTES	7.5	%	7.5	%
				PLATELETS	284	K/UL	284	10^3/UL
				RED BLOOD COUNT	4.33	M/UL	4.33	10^6/UL
				WHITE BLOOD COUNT	6.8	K/UL	6.8	10^3/UL

Listing 10.2
Laboratory Data: Hematology

Center	Patient	Visit	Assessment Date	Parameter	Recorded Result	Recorded Units	Converted Result	Converted Units
XVO_007	402	Day 14	22DEC2009	ANC	4.8	K/UL	4.8	10^3/UL
				BASOPHILS	0.4	%	0.4	%
				EOSINOPHILS	1.2	%	1.2	%
				HEMATOCRIT(PCV)	39.8	%	39.8	%
				HEMOGLOBIN	13.6	G/DL	13.6	G/DL
				LYMPHOCYTES	21.6	%	21.6	%
				MCH	29.8	PG	29.8	PG
				MCHC	34.1	PG	34.1	PG
				MCV	87.2	FL	87.2	FL
				MONOCYTES	8.0	%	8.0	%
				PLATELETS	305	K/UL	305	10^3/UL
				RED BLOOD COUNT	4.57	M/UL	4.57	10^6/UL
				WHITE BLOOD COUNT	6.9	K/UL	6.9	10^3/UL
		Day 30	05JAN2010	ANC	6.5	K/UL	6.5	10^3/UL
				BASOPHILS	0.4	%	0.4	%
				EOSINOPHILS	0.8	%	0.8	%
				HEMATOCRIT(PCV)	40.5	%	40.5	%
				HEMOGLOBIN	13.9	G/DL	13.9	G/DL
				LYMPHOCYTES	15.1	%	15.1	%
				MCH	30.0	PG	30.0	PG
				MCHC	34.2	PG	34.2	PG
				MCV	87.7	FL	87.7	FL
				MONOCYTES	8.1	%	8.1	%
				PLATELETS	270	K/UL	270	10^3/UL
				RED BLOOD COUNT	4.61	M/UL	4.61	10^6/UL
				WHITE BLOOD COUNT	8.6	K/UL	8.6	10^3/UL

Listing 10.2
Laboratory Data: Hematology

Center	Patient	Visit	Assessment Date	Parameter	Recorded Result	Recorded Units	Converted Result	Converted Units
XVO_007	402	Day 42	19JAN2010	ANC	4.9	K/UL	4.9	10^3/UL
				BASOPHILS	0.8	%	0.8	%
				EOSINOPHILS	1.7	%	1.7	%
				HEMATOCRIT(PCV)	40.2	%	40.2	%
				HEMOGLOBIN	13.7	G/DL	13.7	G/DL
				LYMPHOCYTES	23.8	%	23.8	%
				MCH	30.0	PG	30.0	PG
				MCHC	34.2	PG	34.2	PG
				MCV	87.7	FL	87.7	FL
				MONOCYTES	8.9	%	8.9	%
				PLATELETS	277	K/UL	277	10^3/UL
				RED BLOOD COUNT	4.58	M/UL	4.58	10^6/UL
				WHITE BLOOD COUNT	7.5	K/UL	7.5	10^3/UL

Listing 10.3
Laboratory Data: Urinalysis

Center	Patient	Visit	Assessment Date	Parameter	Recorded Result	Recorded Units	Converted Result	Converted Units
XVO_001	101	Screening	09NOV2009	BILIRUBIN	ND		ND	
				CASTS	NEGATIVE	/LPF	NEGATIVE	/LPF
				CRYSTALS	NEGATIVE	/LPF	NEGATIVE	/LPF
				EPITHELIAL CELLS	ND	/HPF	ND	/HPF
				GLUCOSE	NORMAL		NORMAL	
				HEMOGLOBIN	NEGATIVE		NEGATIVE	
				KETONES	(-) NEGATIVE		(-) NEGATIVE	MG/DL
				PH	5.5		5.5	
				PROTEIN	(-) NEGATIVE		(-) NEGATIVE	MG/DL
				RED BLOOD CELLS	1	/HPF	1	/UL
				SPECIFIC GRAVITY	1.005		1.005	
				WHITE BLOOD CELLS	9	/HPF	9	/UL
		Day 6	16NOV2009	BILIRUBIN	ND		ND	
				CASTS	NEGATIVE	/LPF	NEGATIVE	/LPF
				CRYSTALS	NEGATIVE	/LPF	NEGATIVE	/LPF
				EPITHELIAL CELLS	1	/HPF	1	/HPF
				GLUCOSE	NORMAL		NORMAL	
				HEMOGLOBIN	NEGATIVE		NEGATIVE	
				KETONES	NEGATIVE		NEGATIVE	MG/DL
				PH	6.0		6.0	
				PROTEIN	NEGATIVE		NEGATIVE	MG/DL
				RED BLOOD CELLS	6	/HPF	6	/UL
				SPECIFIC GRAVITY	1.013		1.013	
				WHITE BLOOD CELLS	3	/HPF	3	/UL

Note: ND=Not Done.

Listing 10.3
Laboratory Data: Urinalysis

Center	Patient	Visit	Assessment Date	Parameter	Recorded Result	Recorded Units	Converted Result	Converted Units
XVO_001	101	Day 14	24NOV2009	BILIRUBIN	ND		ND	
				CASTS	NEGATIVE	/LPF	NEGATIVE	/LPF
				CRYSTALS	NEGATIVE	/LPF	NEGATIVE	/LPF
				EPITHELIAL CELLS	4	/HPF	4	/HPF
				GLUCOSE	NORMAL		NORMAL	
				HEMOGLOBIN	SMALL		SMALL	
				KETONES	NEGATIVE		NEGATIVE	MG/DL
				PH	5.0		5.0	
				PROTEIN	NEGATIVE		NEGATIVE	MG/DL
				RED BLOOD CELLS	9	/HPF	9	/UL
				SPECIFIC GRAVITY	1.016		1.016	
				WHITE BLOOD CELLS	2	/HPF	2	/UL
		Day 30	11DEC2009	BILIRUBIN	ND		ND	
				CASTS	NEGATIVE	/LPF	NEGATIVE	/LPF
				CRYSTALS	NEGATIVE	/LPF	NEGATIVE	/LPF
				EPITHELIAL CELLS	<1	/HPF	<1	/HPF
				GLUCOSE	NORMAL		NORMAL	
				HEMOGLOBIN	NEGATIVE		NEGATIVE	
				KETONES	NEGATIVE		NEGATIVE	MG/DL
				PH	5.5		5.5	
				PROTEIN	NEGATIVE		NEGATIVE	MG/DL
				RED BLOOD CELLS	3	/HPF	3	/UL
				SPECIFIC GRAVITY	1.006		1.006	
				WHITE BLOOD CELLS	<1	/HPF	<1	/UL

Note: ND=Not Done.

Listing 10.3
Laboratory Data: Urinalysis

Center	Patient	Visit	Assessment Date	Parameter	Recorded Result	Recorded Units	Converted Result	Converted Units
XVO_001	101	Day 42	05JAN2010	BILIRUBIN	ND		ND	
				CASTS	ND	/LPF	ND	/LPF
				CRYSTALS	ND	/LPF	ND	/LPF
				EPITHELIAL CELLS	ND	/HPF	ND	/HPF
				GLUCOSE	ND		ND	
				HEMOGLOBIN	ND		ND	
				KETONES	ND		ND	MG/DL
				PROTEIN	7	MG/DL	7	MG/DL
				RED BLOOD CELLS	ND	/HPF	ND	/UL
				SPECIFIC GRAVITY	ND		ND	
				WHITE BLOOD CELLS	ND	/HPF	ND	/UL
	102	Screening	11DEC2009	CASTS	NEGATIVE	/LPF	NEGATIVE	/LPF
				CRYSTALS	NEGATIVE	/LPF	NEGATIVE	/LPF
				EPITHELIAL CELLS	1	/HPF	1	/HPF
				GLUCOSE	NORMAL		NORMAL	
				HEMOGLOBIN	NEGATIVE		NEGATIVE	
				KETONES	TRACE		TRACE	MG/DL
				PH	6.0		6.0	
				PROTEIN	30	MG/DL	30	MG/DL
				RED BLOOD CELLS	4	/HPF	4	/UL
				SPECIFIC GRAVITY	1.032		1.032	
				WHITE BLOOD CELLS	2	/HPF	2	/UL
		Day 0 Pre-dose	15DEC2009	BILIRUBIN	ND		ND	
				CASTS	NEGATIVE	/LPF	NEGATIVE	/LPF

Note: ND=Not Done.

Listing 10.3
Laboratory Data: Urinalysis

Center	Patient	Visit	Assessment Date	Parameter	Recorded Result	Recorded Units	Converted Result	Converted Units
XVO_001	102	Day 0 Pre-dose	15DEC2009	CRYSTALS	NEGATIVE	/LPF	NEGATIVE	/LPF
				EPITHELIAL CELLS	0	/HPF	0	/HPF
				GLUCOSE	NORMAL		NORMAL	
				HEMOGLOBIN	NEGATIVE		NEGATIVE	
				KETONES	NEGATIVE		NEGATIVE	MG/DL
				PH	6.0		6.0	
				PROTEIN	10	MG/DL	10	MG/DL
				RED BLOOD CELLS	1	/HPF	1	/UL
				SPECIFIC GRAVITY	1.013		1.013	
				WHITE BLOOD CELLS	1	/HPF	1	/UL
		Day 6	21DEC2009	BILIRUBIN	ND		ND	
				CASTS	NEGATIVE	/LPF	NEGATIVE	/LPF
				CRYSTALS	NEGATIVE	/LPF	NEGATIVE	/LPF
				EPITHELIAL CELLS	0	/HPF	0	/HPF
				GLUCOSE	NORMAL		NORMAL	
				HEMOGLOBIN	NEGATIVE		NEGATIVE	
				KETONES	10	MG/DL	10	MG/DL
				PH	6.0		6.0	
				PROTEIN	30	MG/DL	30	MG/DL
				RED BLOOD CELLS	4	/HPF	4	/UL
				SPECIFIC GRAVITY	1.029		1.029	
				WHITE BLOOD CELLS	2	/HPF	2	/UL
		Day 14	29DEC2009	BILIRUBIN	ND		ND	
				CASTS	NEGATIVE	/LPF	NEGATIVE	/LPF

Note: ND=Not Done.

Listing 10.3
Laboratory Data: Urinalysis

Center	Patient	Visit	Assessment Date	Parameter	Recorded Result	Recorded Units	Converted Result	Converted Units
XVO_001	102	Day 14	29DEC2009	CRYSTALS	NEGATIVE	/LPF	NEGATIVE	/LPF
				EPITHELIAL CELLS	0	/HPF	0	/HPF
				GLUCOSE	NORMAL		NORMAL	
				HEMOGLOBIN	NEGATIVE		NEGATIVE	
				KETONES	NEGATIVE		NEGATIVE	MG/DL
				PH	7.0		7.0	
				PROTEIN	NEGATIVE		NEGATIVE	MG/DL
				RED BLOOD CELLS	1	/HPF	1	/UL
				SPECIFIC GRAVITY	1.017		1.017	
				WHITE BLOOD CELLS	1	/HPF	1	/UL
		Day 30	15JAN2010	BILIRUBIN	ND		ND	
				CASTS	NEGATIVE	/LPF	NEGATIVE	/LPF
				CRYSTALS	NEGATIVE	/LPF	NEGATIVE	/LPF
				EPITHELIAL CELLS	1	/HPF	1	/HPF
				GLUCOSE	NORMAL		NORMAL	
				HEMOGLOBIN	NEGATIVE		NEGATIVE	
				KETONES	NEGATIVE		NEGATIVE	MG/DL
				PH	6.0		6.0	
				PROTEIN	NEGATIVE		NEGATIVE	MG/DL
				RED BLOOD CELLS	2	/HPF	2	/UL
				SPECIFIC GRAVITY	1.019		1.019	
				WHITE BLOOD CELLS	1	/HPF	1	/UL
		Day 42	29JAN2010	PROTEIN	ND		ND	MG/DL
	103	Screening	11JAN2010	BILIRUBIN	ND		ND	

Note: ND=Not Done.

Listing 10.3
Laboratory Data: Urinalysis

Center	Patient	Visit	Assessment Date	Parameter	Recorded Result	Recorded Units	Converted Result	Converted Units
XVO_001	103	Screening	11JAN2010	CASTS	NEGATIVE	/LPF	NEGATIVE	/LPF
				CRYSTALS	NEGATIVE	/LPF	NEGATIVE	/LPF
				EPITHELIAL CELLS	0	/HPF	0	/HPF
				GLUCOSE	NORMAL		NORMAL	
				HEMOGLOBIN	NEGATIVE		NEGATIVE	
				KETONES	NEGATIVE		NEGATIVE	MG/DL
				PH	5.0		5.0	
				PROTEIN	NEGATIVE		NEGATIVE	MG/DL
				RED BLOOD CELLS	5	/HPF	5	/UL
				SPECIFIC GRAVITY	1.008		1.008	
				WHITE BLOOD CELLS	1	/HPF	1	/UL
		Day 6	19JAN2010	BILIRUBIN	ND		ND	
				CASTS	NEGATIVE	/LPF	NEGATIVE	/LPF
				CRYSTALS	NEGATIVE	/LPF	NEGATIVE	/LPF
				EPITHELIAL CELLS	0	/HPF	0	/HPF
				GLUCOSE	NORMAL		NORMAL	
				HEMOGLOBIN	TRACE		TRACE	
				KETONES	NEGATIVE		NEGATIVE	MG/DL
				PH	6.0		6.0	
				PROTEIN	NEGATIVE		NEGATIVE	MG/DL
				RED BLOOD CELLS	9	/HPF	9	/UL
				SPECIFIC GRAVITY	1.025		1.025	
				WHITE BLOOD CELLS	1	/HPF	1	/UL
		Day 14	26JAN2010	BILIRUBIN	ND		ND	

Note: ND=Not Done.

Listing 10.3
Laboratory Data: Urinalysis

Center	Patient	Visit	Assessment Date	Parameter	Recorded Result	Recorded Units	Converted Result	Converted Units
XVO_001	103	Day 14	26JAN2010	CASTS	NEGATIVE	/LPF	NEGATIVE	/LPF
				CRYSTALS	NEGATIVE	/LPF	NEGATIVE	/LPF
				EPITHELIAL CELLS	0	/HPF	0	/HPF
				GLUCOSE	NORMAL		NORMAL	
				HEMOGLOBIN	NEGATIVE		NEGATIVE	
				KETONES	NEGATIVE		NEGATIVE	MG/DL
				PH	6.0		6.0	
				PROTEIN	NEGATIVE		NEGATIVE	MG/DL
				RED BLOOD CELLS	1	/HPF	1	/UL
				SPECIFIC GRAVITY	1.005		1.005	
				WHITE BLOOD CELLS	0	/HPF	0	/UL
		Day 30	09FEB2010	BILIRUBIN	ND		ND	
				CASTS	NEGATIVE	/LPF	NEGATIVE	/LPF
				CRYSTALS	NEGATIVE	/LPF	NEGATIVE	/LPF
				EPITHELIAL CELLS	0	/HPF	0	/HPF
				GLUCOSE	NORMAL		NORMAL	
				HEMOGLOBIN	NEGATIVE		NEGATIVE	
				KETONES	NEGATIVE		NEGATIVE	MG/DL
				PH	6.0		6.0	
				PROTEIN	NEGATIVE		NEGATIVE	MG/DL
				RED BLOOD CELLS	7	/HPF	7	/UL
				SPECIFIC GRAVITY	1.016		1.016	
				WHITE BLOOD CELLS	1	/HPF	1	/UL
		Day 42	19FEB2010	BILIRUBIN	ND		ND	

Note: ND=Not Done.

Listing 10.3
Laboratory Data: Urinalysis

Center	Patient	Visit	Assessment Date	Parameter	Recorded Result	Recorded Units	Converted Result	Converted Units
XVO_001	103	Day 42	19FEB2010	CASTS	NEGATIVE	/LPF	NEGATIVE	/LPF
				CRYSTALS	NEGATIVE	/LPF	NEGATIVE	/LPF
				EPITHELIAL CELLS	0	/HPF	0	/HPF
				GLUCOSE	NORMAL		NORMAL	
				HEMOGLOBIN	TRACE		TRACE	
				KETONES	NEGATIVE		NEGATIVE	MG/DL
				PH	5.0		5.0	
				PROTEIN	NEGATIVE		NEGATIVE	MG/DL
				RED BLOOD CELLS	4	/HPF	4	/UL
				SPECIFIC GRAVITY	1.021		1.021	
				WHITE BLOOD CELLS	1	/HPF	1	/UL
XVO_003	201	Screening	10NOV2009	BILIRUBIN	NEG		NEG	
				CASTS	11	/LPF	11	/LPF
				CRYSTALS	ND	/LPF	ND	/LPF
				EPITHELIAL CELLS	0	/HPF	0	/HPF
				GLUCOSE	NEG		NEG	
				HEMOGLOBIN	NEG		NEG	
				KETONES	NEG		NEG	MG/DL
				PH	5.0		5.0	
				PROTEIN	NEG		NEG	MG/DL
				RED BLOOD CELLS	8	/UL	8	/UL
				SPECIFIC GRAVITY	1.018		1.018	
				WHITE BLOOD CELLS	2	/UL	2	/UL
		Day 0 Pre-dose	17NOV2009	BILIRUBIN	NEG		NEG	

Note: ND=Not Done.

Listing 10.3
Laboratory Data: Urinalysis

Center	Patient	Visit	Assessment Date	Parameter	Recorded Result	Recorded Units	Converted Result	Converted Units
XVO_003	201	Day 0 Pre-dose	17NOV2009	CASTS	0	/LPF	0	/LPF
				CRYSTALS	NEG	/LPF	NEG	/LPF
				EPITHELIAL CELLS	0	/HPF	0	/HPF
				GLUCOSE	NEG		NEG	
				HEMOGLOBIN	NEG		NEG	
				KETONES	NEG		NEG	MG/DL
				PH	5.5		5.5	
				PROTEIN	NEG		NEG	MG/DL
				RED BLOOD CELLS	22	/UL	22	/UL
				SPECIFIC GRAVITY	1.018		1.018	
				WHITE BLOOD CELLS	9	/UL	9	/UL
		Day 6	23NOV2009	BILIRUBIN	NEG		NEG	
				CASTS	4	/LPF	4	/LPF
				CRYSTALS	NEG	/LPF	NEG	/LPF
				EPITHELIAL CELLS	0	/HPF	0	/HPF
				GLUCOSE	NEG		NEG	
				HEMOGLOBIN	NEG		NEG	
				KETONES	NEG		NEG	MG/DL
				PH	5.5		5.5	
				PROTEIN	TRACES		TRACES	MG/DL
				RED BLOOD CELLS	15	/UL	15	/UL
				SPECIFIC GRAVITY	1.022		1.022	
				WHITE BLOOD CELLS	9	/UL	9	/UL
		Day 14	02DEC2009	BILIRUBIN	NEG		NEG	

Note: ND=Not Done.

Listing 10.3
Laboratory Data: Urinalysis

Center	Patient	Visit	Assessment Date	Parameter	Recorded Result	Recorded Units	Converted Result	Converted Units
XVO_003	201	Day 14	02DEC2009	CASTS	11	/LPF	11	/LPF
				CRYSTALS	NEG	/LPF	NEG	/LPF
				EPITHELIAL CELLS	0	/HPF	0	/HPF
				GLUCOSE	NEG		NEG	
				HEMOGLOBIN	NEG		NEG	
				KETONES	NEG		NEG	MG/DL
				PH	5.5		5.5	
				PROTEIN	NEG		NEG	MG/DL
				RED BLOOD CELLS	0	/UL	0	/UL
				SPECIFIC GRAVITY	1.014		1.014	
				WHITE BLOOD CELLS	0	/UL	0	/UL
		Day 30	22DEC2009	BILIRUBIN	NEGATIVE		NEGATIVE	
				CASTS	0	/LPF	0	/LPF
				CRYSTALS	NEG	/LPF	NEG	/LPF
				EPITHELIAL CELLS	0	/HPF	0	/HPF
				GLUCOSE	NEGATIVE		NEGATIVE	
				HEMOGLOBIN	NEGATIVE		NEGATIVE	
				KETONES	NEGATIVE		NEGATIVE	MG/DL
				PH	5.0		5.0	
				PROTEIN	TRACE		TRACE	MG/DL
				RED BLOOD CELLS	6	/UL	6	/UL
				SPECIFIC GRAVITY	1.016		1.016	
				WHITE BLOOD CELLS	5	/UL	5	/UL
		Day 42	29DEC2009	BILIRUBIN	NEG		NEG	

Note: ND=Not Done.

Listing 10.3
Laboratory Data: Urinalysis

Center	Patient	Visit	Assessment Date	Parameter	Recorded Result	Recorded Units	Converted Result	Converted Units
XVO_003	201	Day 42	29DEC2009	CASTS	3	/LPF	3	/LPF
				CRYSTALS	NEG	/LPF	NEG	/LPF
				EPITHELIAL CELLS	0	/HPF	0	/HPF
				GLUCOSE	NEG		NEG	
				HEMOGLOBIN	NEG		NEG	
				KETONES	NEG		NEG	MG/DL
				PH	5.5		5.5	
				PROTEIN	NEG		NEG	MG/DL
				RED BLOOD CELLS	5	/UL	5	/UL
				SPECIFIC GRAVITY	1.015		1.015	
				WHITE BLOOD CELLS	10	/UL	10	/UL
XVO_005	301	Screening	03AUG2009	BILIRUBIN	NEG		NEG	
				CASTS	3	/LPF	3	/LPF
				CRYSTALS	0	/LPF	0	/LPF
				EPITHELIAL CELLS	0	/HPF	0	/HPF
				GLUCOSE	NEG		NEG	
				HEMOGLOBIN	NONE		NONE	
				KETONES	NEG		NEG	MG/DL
				PH	5.0		5.0	
				PROTEIN	NEG		NEG	MG/DL
				RED BLOOD CELLS	1	/HPF	1	/UL
				SPECIFIC GRAVITY	1.025		1.025	
				WHITE BLOOD CELLS	0	/HPF	0	/UL
		Day 0 Pre-dose	04AUG2009	BILIRUBIN	NEG		NEG	

Note: ND=Not Done.

Listing 10.3
Laboratory Data: Urinalysis

Center	Patient	Visit	Assessment Date	Parameter	Recorded Result	Recorded Units	Converted Result	Converted Units
XVO_005	301	Day 0 Pre-dose	04AUG2009	CASTS	0	/LPF	0	/LPF
				CRYSTALS	NONE	/LPF	NONE	/LPF
				EPITHELIAL CELLS	1	/HPF	1	/HPF
				GLUCOSE	NEG		NEG	
				HEMOGLOBIN	NONE		NONE	
				KETONES	NEG		NEG	MG/DL
				PH	5.0		5.0	
				PROTEIN	NEG		NEG	MG/DL
				RED BLOOD CELLS	0	/HPF	0	/UL
				SPECIFIC GRAVITY	1.020		1.020	
				WHITE BLOOD CELLS	0	/HPF	0	/UL
		Day 6	10AUG2009	BILIRUBIN	NEG		NEG	
				CASTS	5	/LPF	5	/LPF
				CRYSTALS	NONE	/LPF	NONE	/LPF
				EPITHELIAL CELLS	3	/HPF	3	/HPF
				GLUCOSE	NEG		NEG	
				HEMOGLOBIN	NONE		NONE	
				KETONES	NEG		NEG	MG/DL
				PH	5.0		5.0	
				PROTEIN	TRACE		TRACE	MG/DL
				RED BLOOD CELLS	1	/HPF	1	/UL
				SPECIFIC GRAVITY	1.030		1.030	
				WHITE BLOOD CELLS	1	/HPF	1	/UL
		Day 14	18AUG2009	BILIRUBIN	NEG		NEG	

Note: ND=Not Done.

Listing 10.3
Laboratory Data: Urinalysis

Center	Patient	Visit	Assessment Date	Parameter	Recorded Result	Recorded Units	Converted Result	Converted Units
XVO_005	301	Day 14	18AUG2009	CASTS	2	/LPF	2	/LPF
				CRYSTALS	NONE	/LPF	NONE	/LPF
				EPITHELIAL CELLS	0	/HPF	0	/HPF
				GLUCOSE	NEG		NEG	
				HEMOGLOBIN	NONE		NONE	
				KETONES	NEG		NEG	MG/DL
				PH	5.0		5.0	
				PROTEIN	NEG		NEG	MG/DL
				RED BLOOD CELLS	1	/HPF	1	/UL
				SPECIFIC GRAVITY	>1.030		>1.030	
				WHITE BLOOD CELLS	1	/HPF	1	/UL
		Day 30	04SEP2009	BILIRUBIN	NEG		NEG	
				CASTS	0	/LPF	0	/LPF
				CRYSTALS	NONE	/LPF	NONE	/LPF
				EPITHELIAL CELLS	0	/HPF	0	/HPF
				GLUCOSE	TRACE		TRACE	
				HEMOGLOBIN	NONE		NONE	
				KETONES	NEG		NEG	MG/DL
				PH	5.5		5.5	
				PROTEIN	NEG		NEG	MG/DL
				RED BLOOD CELLS	0	/HPF	0	/UL
				SPECIFIC GRAVITY	1.015		1.015	
				WHITE BLOOD CELLS	0	/HPF	0	/UL
		Day 42	15SEP2009	BILIRUBIN	NEG		NEG	

Note: ND=Not Done.

Listing 10.3
Laboratory Data: Urinalysis

Center	Patient	Visit	Assessment Date	Parameter	Recorded Result	Recorded Units	Converted Result	Converted Units
XVO_005	301	Day 42	15SEP2009	CASTS	ND	/LPF	ND	/LPF
				CRYSTALS	NONE	/LPF	NONE	/LPF
				EPITHELIAL CELLS	1	/HPF	1	/HPF
				GLUCOSE	NEG		NEG	
				HEMOGLOBIN	NONE		NONE	
				KETONES	NEG		NEG	MG/DL
				PH	5.5		5.5	
				PROTEIN	NEG		NEG	MG/DL
				RED BLOOD CELLS	1	/HPF	1	/UL
				SPECIFIC GRAVITY	1.025		1.025	
				WHITE BLOOD CELLS	1	/HPF	1	/UL
	302	Screening	14DEC2009	BILIRUBIN	NEG		NEG	
				CASTS	0	/LPF	0	/LPF
				CRYSTALS	0	/LPF	0	/LPF
				EPITHELIAL CELLS	1	/HPF	1	/HPF
				GLUCOSE	NEG		NEG	
				HEMOGLOBIN	NONE		NONE	
				KETONES	NEG		NEG	MG/DL
				PH	6.5		6.5	
				PROTEIN	1+		1+	MG/DL
				RED BLOOD CELLS	5	/HPF	5	/UL
				SPECIFIC GRAVITY	1.017		1.017	
				WHITE BLOOD CELLS	4	/HPF	4	/UL
		Day 0 Pre-dose	15DEC2009	BILIRUBIN	NEG		NEG	

Note: ND=Not Done.

Listing 10.3
Laboratory Data: Urinalysis

Center	Patient	Visit	Assessment Date	Parameter	Recorded Result	Recorded Units	Converted Result	Converted Units
XVO_005	302	Day 0 Pre-dose	15DEC2009	CASTS	1	/LPF	1	/LPF
				CRYSTALS	0	/LPF	0	/LPF
				EPITHELIAL CELLS	1	/HPF	1	/HPF
				GLUCOSE	NEG		NEG	
				HEMOGLOBIN	NONE		NONE	
				KETONES	NEG		NEG	MG/DL
				PH	6.5		6.5	
				PROTEIN	NEG		NEG	MG/DL
				RED BLOOD CELLS	4	/HPF	4	/UL
				SPECIFIC GRAVITY	1.015		1.015	
				WHITE BLOOD CELLS	3	/HPF	3	/UL
		Day 6	21DEC2009	BILIRUBIN	NEG		NEG	
				CASTS	0	/LPF	0	/LPF
				CRYSTALS	0	/LPF	0	/LPF
				EPITHELIAL CELLS	0	/HPF	0	/HPF
				GLUCOSE	NEG		NEG	
				HEMOGLOBIN	NONE		NONE	
				KETONES	NEG		NEG	MG/DL
				PH	6.0		6.0	
				PROTEIN	NEG		NEG	MG/DL
				RED BLOOD CELLS	2	/HPF	2	/UL
				SPECIFIC GRAVITY	1.013		1.013	
				WHITE BLOOD CELLS	3	/HPF	3	/UL
		Day 14	29DEC2009	BILIRUBIN	NEG		NEG	

Note: ND=Not Done.

Listing 10.3
Laboratory Data: Urinalysis

Center	Patient	Visit	Assessment Date	Parameter	Recorded Result	Recorded Units	Converted Result	Converted Units
XVO_005	302	Day 14	29DEC2009	CASTS	0	/LPF	0	/LPF
				CRYSTALS	0	/LPF	0	/LPF
				EPITHELIAL CELLS	0	/HPF	0	/HPF
				GLUCOSE	NEG		NEG	
				HEMOGLOBIN	NONE		NONE	
				KETONES	NEG		NEG	MG/DL
				PH	6.0		6.0	
				PROTEIN	NEG		NEG	MG/DL
				RED BLOOD CELLS	3	/HPF	3	/UL
				SPECIFIC GRAVITY	1.016		1.016	
				WHITE BLOOD CELLS	2	/HPF	2	/UL
		Day 30	15JAN2010	BILIRUBIN	NEG		NEG	
				CASTS	0	/LPF	0	/LPF
				CRYSTALS	0	/LPF	0	/LPF
				EPITHELIAL CELLS	0	/HPF	0	/HPF
				GLUCOSE	NEG		NEG	
				HEMOGLOBIN	NONE		NONE	
				KETONES	NEG		NEG	MG/DL
				PH	6.0		6.0	
				PROTEIN	TRACE		TRACE	MG/DL
				RED BLOOD CELLS	5.0	/HPF	5.0	/UL
				SPECIFIC GRAVITY	1.018		1.018	
				WHITE BLOOD CELLS	2.0	/HPF	2.0	/UL
		Day 42	25JAN2010	BILIRUBIN	NEG		NEG	

Note: ND=Not Done.

Listing 10.3
Laboratory Data: Urinalysis

Center	Patient	Visit	Assessment Date	Parameter	Recorded Result	Recorded Units	Converted Result	Converted Units
XVO_005	302	Day 42	25JAN2010	CASTS	0	/LPF	0	/LPF
				CRYSTALS	0	/LPF	0	/LPF
				EPITHELIAL CELLS	0	/HPF	0	/HPF
				GLUCOSE	NEG		NEG	
				HEMOGLOBIN	NONE		NONE	
				KETONES	NEG		NEG	MG/DL
				PH	6.0		6.0	
				PROTEIN	TRACE		TRACE	MG/DL
				RED BLOOD CELLS	5	/HPF	5	/UL
				SPECIFIC GRAVITY	1.020		1.020	
				WHITE BLOOD CELLS	2	/HPF	2	/UL
XVO_007	401	Screening	04NOV2009	BILIRUBIN	NEGATIVE		NEGATIVE	
				CASTS	NONE SEEN	/LPF	NONE SEEN	/LPF
				CRYSTALS	AMORPHOUS URATES	/LPF	AMORPHOUS URATES	/LPF
				EPITHELIAL CELLS	RARE	/HPF	RARE	/HPF
				GLUCOSE	NEGATIVE		NEGATIVE	
				HEMOGLOBIN	NEGATIVE		NEGATIVE	
				KETONES	NEGATIVE		NEGATIVE	MG/DL
				PH	6.0		6.0	
				PROTEIN	NEGATIVE		NEGATIVE	MG/DL
				RED BLOOD CELLS	<2	/HPF	<2	/UL
				SPECIFIC GRAVITY	1.025		1.025	
				WHITE BLOOD CELLS	<2	/HPF	<2	/UL
		Day 0 Pre-dose	10NOV2009	BILIRUBIN	NEGATIVE		NEGATIVE	

Note: ND=Not Done.

Listing 10.3
Laboratory Data: Urinalysis

Center	Patient	Visit	Assessment Date	Parameter	Recorded Result	Recorded Units	Converted Result	Converted Units
XVO_007	401	Day 0 Pre-dose	10NOV2009	CASTS	NONE SEEN	/LPF	NONE SEEN	/LPF
				CRYSTALS	NONE SEEN	/LPF	NONE SEEN	/LPF
				EPITHELIAL CELLS	NONE SEEN	/HPF	NONE SEEN	/HPF
				GLUCOSE	NEGATIVE		NEGATIVE	
				HEMOGLOBIN	NEGATIVE		NEGATIVE	
				KETONES	NEGATIVE		NEGATIVE	MG/DL
				PH	6.0		6.0	
				PROTEIN	NEGATIVE		NEGATIVE	MG/DL
				RED BLOOD CELLS	<2	/HPF	<2	/UL
				SPECIFIC GRAVITY	1.020		1.020	
				WHITE BLOOD CELLS	<2	/HPF	<2	/UL
		Day 6	16NOV2009	BILIRUBIN	NEGATIVE		NEGATIVE	
				CASTS	NONE	/LPF	NONE	/LPF
				CRYSTALS	NONE	/LPF	NONE	/LPF
				EPITHELIAL CELLS	FEW SQUAMOUS	/HPF	FEW SQUAMOUS	/HPF
				GLUCOSE	NEGATIVE		NEGATIVE	
				HEMOGLOBIN	NEGATIVE		NEGATIVE	
				KETONES	NEGATIVE		NEGATIVE	MG/DL
				PH	5.0		5.0	
				PROTEIN	NEGATIVE		NEGATIVE	MG/DL
				RED BLOOD CELLS	<2	/HPF	<2	/UL
				SPECIFIC GRAVITY	1.020		1.020	
				WHITE BLOOD CELLS	<2	/HPF	<2	/UL
		Day 14	24NOV2009	BILIRUBIN	NEGATIVE		NEGATIVE	

Note: ND=Not Done.

Listing 10.3
Laboratory Data: Urinalysis

Center	Patient	Visit	Assessment Date	Parameter	Recorded Result	Recorded Units	Converted Result	Converted Units
XVO_007	401	Day 14	24NOV2009	CASTS	NONE SEEN	/LPF	NONE SEEN	/LPF
				CRYSTALS	NONE SEEN	/LPF	NONE SEEN	/LPF
				EPITHELIAL CELLS	RARE	/HPF	RARE	/HPF
				GLUCOSE	NEGATIVE		NEGATIVE	
				HEMOGLOBIN	NEGATIVE		NEGATIVE	
				KETONES	NEGATIVE		NEGATIVE	MG/DL
				PH	6.0		6.0	
				PROTEIN	NEGATIVE		NEGATIVE	MG/DL
				RED BLOOD CELLS	<2	/HPF	<2	/UL
				SPECIFIC GRAVITY	1.015		1.015	
				WHITE BLOOD CELLS	<2	/HPF	<2	/UL
		Day 30	08DEC2009	BILIRUBIN	NEGATIVE		NEGATIVE	
				CASTS	NONE SEEN	/LPF	NONE SEEN	/LPF
				CRYSTALS	MANY AMORPHOUS	/LPF	MANY AMORPHOUS	/LPF
				EPITHELIAL CELLS	NONE SEEN	/HPF	NONE SEEN	/HPF
				GLUCOSE	NEGATIVE		NEGATIVE	
				HEMOGLOBIN	NEGATIVE		NEGATIVE	
				KETONES	NEGATIVE		NEGATIVE	MG/DL
				PH	6.0		6.0	
				PROTEIN	NEGATIVE		NEGATIVE	MG/DL
				RED BLOOD CELLS	<2	/HPF	<2	/UL
				SPECIFIC GRAVITY	1.015		1.015	
				WHITE BLOOD CELLS	<2	/HPF	<2	/UL
		Day 42	22DEC2009	BILIRUBIN	NEGATIVE		NEGATIVE	

Note: ND=Not Done.

Listing 10.3
Laboratory Data: Urinalysis

Center	Patient	Visit	Assessment Date	Parameter	Recorded Result	Recorded Units	Converted Result	Converted Units
XVO_007	401	Day 42	22DEC2009	CASTS	NONE SEEN	/LPF	NONE SEEN	/LPF
				CRYSTALS	NONE SEEN	/LPF	NONE SEEN	/LPF
				EPITHELIAL CELLS	RARE SQUAMOUS CELL	/HPF	RARE SQUAMOUS CELL	/HPF
				GLUCOSE	NEGATIVE		NEGATIVE	
				HEMOGLOBIN	NEGATIVE		NEGATIVE	
				KETONES	NEGATIVE		NEGATIVE	MG/DL
				PH	5.0		5.0	
				PROTEIN	NEGATIVE		NEGATIVE	MG/DL
				RED BLOOD CELLS	<2	/HPF	<2	/UL
				SPECIFIC GRAVITY	1.020		1.020	
				WHITE BLOOD CELLS	<2	/HPF	<2	/UL
	402	Screening	03DEC2009	BILIRUBIN	NEGATIVE		NEGATIVE	
				CASTS	NONE SEEN	/LPF	NONE SEEN	/LPF
				CRYSTALS	NONE SEEN	/LPF	NONE SEEN	/LPF
				EPITHELIAL CELLS	NONE SEEN	/HPF	NONE SEEN	/HPF
				GLUCOSE	NEGATIVE		NEGATIVE	
				HEMOGLOBIN	NEGATIVE		NEGATIVE	
				KETONES	NEGATIVE		NEGATIVE	MG/DL
				PH	6.0		6.0	
				PROTEIN	NEGATIVE		NEGATIVE	MG/DL
				RED BLOOD CELLS	<2	/HPF	<2	/UL
				SPECIFIC GRAVITY	1.010		1.010	
				WHITE BLOOD CELLS	<2	/HPF	<2	/UL
		Day 0 Pre-dose	08DEC2009	BILIRUBIN	NEGATIVE		NEGATIVE	

Note: ND=Not Done.

Listing 10.3
Laboratory Data: Urinalysis

Center	Patient	Visit	Assessment Date	Parameter	Recorded Result	Recorded Units	Converted Result	Converted Units
XVO_007	402	Day 0 Pre-dose	08DEC2009	CASTS	NONE SEEN	/LPF	NONE SEEN	/LPF
				CRYSTALS	NONE SEEN	/LPF	NONE SEEN	/LPF
				EPITHELIAL CELLS	RARE	/HPF	RARE	/HPF
				GLUCOSE	NEGATIVE		NEGATIVE	
				HEMOGLOBIN	NEGATIVE		NEGATIVE	
				KETONES	NEGATIVE		NEGATIVE	MG/DL
				PH	6.0		6.0	
				PROTEIN	NEGATIVE		NEGATIVE	MG/DL
				RED BLOOD CELLS	<2	/HPF	<2	/UL
				SPECIFIC GRAVITY	1.015		1.015	
				WHITE BLOOD CELLS	2 TO 5	/HPF	2 TO 5	/UL
		Day 6	14DEC2009	BILIRUBIN	NEG		NEG	
				CASTS	NONE	/LPF	NONE	/LPF
				CRYSTALS	NONE	/LPF	NONE	/LPF
				EPITHELIAL CELLS	NONE	/HPF	NONE	/HPF
				GLUCOSE	NEG		NEG	
				HEMOGLOBIN	NEG		NEG	
				KETONES	NEG		NEG	MG/DL
				PH	8.0		8.0	
				PROTEIN	NEG		NEG	MG/DL
				RED BLOOD CELLS	<2	/HPF	<2	/UL
				SPECIFIC GRAVITY	1.010		1.010	
				WHITE BLOOD CELLS	<2	/HPF	<2	/UL
		Day 14	22DEC2009	BILIRUBIN	NEGATIVE		NEGATIVE	

Note: ND=Not Done.

Listing 10.3
Laboratory Data: Urinalysis

Center	Patient	Visit	Assessment Date	Parameter	Recorded Result	Recorded Units	Converted Result	Converted Units
XVO_007	402	Day 14	22DEC2009	CASTS	NONE SEEN	/LPF	NONE SEEN	/LPF
				CRYSTALS	NONE SEEN	/LPF	NONE SEEN	/LPF
				EPITHELIAL CELLS	NONE SEEN	/HPF	NONE SEEN	/HPF
				GLUCOSE	NEGATIVE		NEGATIVE	
				HEMOGLOBIN	NEGATIVE		NEGATIVE	
				KETONES	NEGATIVE		NEGATIVE	MG/DL
				PH	6.0		6.0	
				PROTEIN	NEGATIVE		NEGATIVE	MG/DL
				RED BLOOD CELLS	<2	/HPF	<2	/UL
				SPECIFIC GRAVITY	1.010		1.010	
				WHITE BLOOD CELLS	<2	/HPF	<2	/UL
		Day 30	05JAN2010	BILIRUBIN	NEGATIVE		NEGATIVE	
				CASTS	NONE SEEN	/LPF	NONE SEEN	/LPF
				CRYSTALS	NONE SEEN	/LPF	NONE SEEN	/LPF
				EPITHELIAL CELLS	NONE SEEN	/HPF	NONE SEEN	/HPF
				GLUCOSE	NEGATIVE		NEGATIVE	
				HEMOGLOBIN	NEGATIVE		NEGATIVE	
				KETONES	NEGATIVE		NEGATIVE	MG/DL
				PH	6.0		6.0	
				PROTEIN	NEGATIVE		NEGATIVE	MG/DL
				RED BLOOD CELLS	<2	/HPF	<2	/UL
				SPECIFIC GRAVITY	1.015		1.015	
				WHITE BLOOD CELLS	<2	/HPF	<2	/UL
		Day 42	19JAN2010	BILIRUBIN	NEGATIVE		NEGATIVE	

Note: ND=Not Done.

Listing 10.3
Laboratory Data: Urinalysis

Center	Patient	Visit	Assessment Date	Parameter	Recorded Result	Recorded Units	Converted Result	Converted Units
XVO_007	402	Day 42	19JAN2010	CASTS	NONE SEEN	/LPF	NONE SEEN	/LPF
				CRYSTALS	NONE SEEN	/LPF	NONE SEEN	/LPF
				EPITHELIAL CELLS	NONE SEEN	/HPF	NONE SEEN	/HPF
				GLUCOSE	NEGATIVE		NEGATIVE	
				HEMOGLOBIN	NEGATIVE		NEGATIVE	
				KETONES	NEGATIVE		NEGATIVE	MG/DL
				PH	7.0		7.0	
				PROTEIN	NEGATIVE		NEGATIVE	MG/DL
				RED BLOOD CELLS	<2	/HPF	<2	/UL
				SPECIFIC GRAVITY	1.005		1.005	
				WHITE BLOOD CELLS	<2	/HPF	<2	/UL

Note: ND=Not Done.

Listing 10.4
Laboratory Data: Lipids

Center	Patient	Visit	Assessment Date	Parameter	Recorded Result	Recorded Units	Converted Result	Converted Units
XVO_001	101	Day 0 Pre-dose	10NOV2009	HDL	43	MG/DL	43	MG/DL
				LDL	133	MG/DL	133	MG/DL
				TOTAL CHOLESTEROL	193	MG/DL	193	MG/DL
				TRIGLYCERIDES	83	MG/DL	83	MG/DL
		Day 3	13NOV2009	HDL	041	MG/DL	041	MG/DL
				LDL	116	MG/DL	116	MG/DL
				TOTAL CHOLESTEROL	172	MG/DL	172	MG/DL
				TRIGLYCERIDES	77	MG/DL	77	MG/DL
	102	Day 0 Pre-dose	15DEC2009	HDL	29	MG/DL	29	MG/DL
				TOTAL CHOLESTEROL	150	MG/DL	150	MG/DL
				TRIGLYCERIDES	110	MG/DL	110	MG/DL
		Day 3	18DEC2009	HDL	32	MG/DL	32	MG/DL
				TOTAL CHOLESTEROL	155	MG/DL	155	MG/DL
				TRIGLYCERIDES	118	MG/DL	118	MG/DL
	103	Day 0 Pre-dose	12JAN2010	HDL	38	MG/DL	38	MG/DL
				TOTAL CHOLESTEROL	210	MG/DL	210	MG/DL
				TRIGLYCERIDES	92	MG/DL	92	MG/DL
		Day 3	15JAN2010	HDL	37	MG/DL	37	MG/DL
				TOTAL CHOLESTEROL	203	MG/DL	203	MG/DL
				TRIGLYCERIDES	101	MG/DL	101	MG/DL
XVO_003	201	Day 0 Pre-dose	17NOV2009	HDL	74	MG/DL	74	MG/DL
				LDL	117	MG/DL	117	MG/DL
				TOTAL CHOLESTEROL	203	MG/DL	203	MG/DL

Listing 10.4
Laboratory Data: Lipids

Center	Patient	Visit	Assessment Date	Parameter	Recorded Result	Recorded Units	Converted Result	Converted Units
XVO_003	201	Day 0 Pre-dose	17NOV2009	TRIGLYCERIDES	59	MG/DL	59	MG/DL
		Day 3	20NOV2009	HDL	75	MG/DL	75	MG/DL
				LDL	113	MG/DL	113	MG/DL
				TOTAL CHOLESTEROL	199	MG/DL	199	MG/DL
				TRIGLYCERIDES	53	MG/DL	53	MG/DL
XVO_005	301	Day 0 Pre-dose	04AUG2009	HDL	34	MG/DL	34	MG/DL
				LDL	76	MG/DL	76	MG/DL
				TOTAL CHOLESTEROL	135	MG/DL	135	MG/DL
				TRIGLYCERIDES	126	MG/DL	126	MG/DL
		Day 3	07AUG2009	HDL	36	MG/DL	36	MG/DL
				LDL	91	MG/DL	91	MG/DL
				TOTAL CHOLESTEROL	157	MG/DL	157	MG/DL
				TRIGLYCERIDES	151	MG/DL	151	MG/DL
	302	Day 0 Pre-dose	15DEC2009	HDL	25	MG/DL	25	MG/DL
				LDL	81	MG/DL	81	MG/DL
				TOTAL CHOLESTEROL	135	MG/DL	135	MG/DL
				TRIGLYCERIDES	143	MG/DL	143	MG/DL
		Day 3	18DEC2009	HDL	25	MG/DL	25	MG/DL
				LDL	72	MG/DL	72	MG/DL
				TOTAL CHOLESTEROL	130	MG/DL	130	MG/DL
				TRIGLYCERIDES	162	MG/DL	162	MG/DL
XVO_007	401	Day 0 Pre-dose	10NOV2009	HDL	40	MG/DL	40	MG/DL
				LDL	147	MG/DL	147	MG/DL

Listing 10.4
Laboratory Data: Lipids

Center	Patient	Visit	Assessment Date	Parameter	Recorded Result	Recorded Units	Converted Result	Converted Units
XVO_007	401	Day 0 Pre-dose	10NOV2009	TOTAL CHOLESTEROL	214	MG/DL	214	MG/DL
				TRIGLYCERIDES	136	MG/DL	136	MG/DL
		Day 3	13NOV2009	HDL	39	MG/DL	39	MG/DL
				LDL	144	MG/DL	144	MG/DL
				TOTAL CHOLESTEROL	202	MG/DL	202	MG/DL
				TRIGLYCERIDES	93	MG/DL	93	MG/DL
	402	Day 0 Pre-dose	08DEC2009	HDL	66	MG/DL	66	MG/DL
				LDL	122	MG/DL	122	MG/DL
				TOTAL CHOLESTEROL	208	MG/DL	208	MG/DL
				TRIGLYCERIDES	102	MG/DL	102	MG/DL
		Day 3	11DEC2009	HDL	63	MG/DL	63	MG/DL
				LDL	119	MG/DL	119	MG/DL
				TOTAL CHOLESTEROL	201	MG/DL	201	MG/DL
				TRIGLYCERIDES	96	MG/DL	96	MG/DL

Listing 11
Concomitant Medications

Center	Patient	Preferred Term / Investigator Text	Dose/Unit	Route [a]	Start Date	Stop Date	AE No [b]	Indication
XVO_001	101	ENALAPRIL MALEATE/ ENALAPRIL MALEATE	5MG	1	PRE-STUDY	CONTINUING		HYPERTENSION
		CALCIUM/ CALCIUM	600MG	1	PRE-STUDY	CONTINUING		GENERAL HEALTH
		MULTIVITAMINS/ MULTIVITAMINS	1 DOSE TABLET	1	PRE-STUDY			GENERAL HEALTH
		ACETYLSALICYLIC ACID/ ASPIRIN	1TABLET	1	01JAN2005	CONTINUING		GENERAL HEALTH
		INFLUENZA VACCINE/ FLUZONE	.50ML	3	30OCT2009	30OCT2009		VACCINATION
		POTASSIUM IODIDE/ IOSAT	130MG	1	09NOV2009	24NOV2009		THYROID BLOCKADE
	102	HYDROCORTISONE/ HYDROCORTISONE	1%	8	PRE-STUDY	CONTINUING		RASH
		LEVOTHYROXINE SODIUM/ LEVOXYL	UNK	1	01JAN2000	CONTINUING		HYPOTHYROIDISM
		LISINOPRIL/ LISINOPRIL	UNK	1	01JAN2004	CONTINUING		HYPERTENSION
		LOVASTATIN/ LOVASTATIN	UNK	1	01DEC2008	CONTINUING		HYPERCHOLESTEROLEMIA
		POTASSIUM IODIDE/ IOSAT	130MG	1	14DEC2009	29DEC2009		THYROID BLOCKADE
		DOCUSATE SODIUM/ COLACE	UNK	1	01JAN2010	CONTINUING	2	CONSTIPATION
	103	METOPROLOL SUCCINATE/ METOPROLOL SUCCINATE	25/MG	1	01JAN2005	CONTINUING		HYPERTENSION
		LORAZEPAM/ ATIVAN	1MG	1	13NOV2007	CONTINUING		SLEEP AID

-- =Date unknown or partially unknown
UNK =Unknown
Note: [a] Route: 1=Oral 2=IV 3=IM 4=Subcutaneous 5=Rectal 6=Transdermal 7=Inhalation 8=Topical 9=Ophthalmic 99=Other
[b] Indicates the AE number if this treatment is being used to treat a recorded AE

Listing 11
Concomitant Medications

Center	Patient	Preferred Term / Investigator Text	Dose/Unit	Route [a]	Start Date	Stop Date	AE No [b]	Indication
XVO_001	103	ACETYLSALICYLIC ACID/ ASPIRIN	81MG	1	01JAN2009	CONTINUING		GENERAL HEALTH
		VITAMIN E /01552201// VITAMIN E	UNK	1	01JAN2009	CONTINUING		GENERAL HEALTH
		COLECALCIFEROL/ VITAMIN D	UNK	1	01JAN2009	CONTINUING		GENERAL HEALTH
		RANIBIZUMAB/ LUCENTIS SOLUTION	UNK	9	01JAN2009	CONTINUING		MACULAR DEGENERATION
		ESCITALOPRAM OXALATE/ LEXAPRO	20MG	1	01JAN2009	CONTINUING		MOOD LIFTER
		POTASSIUM IODIDE/ IOSAT	130/MG	1	11JAN2010	26JAN2010		THYROID BLOCKADE
XVO_003	201	CALCIUM WITH VITAMIN D /01233101// CALCIUM + VITAMIN D	1000MG	1	PRE-STUDY	CONTINUING		OSTEOPOROSIS
		DUTASTERIDE/ AVODART (DUTASTERIDE)	0.5MG	1	PRE-STUDY	CONTINUING		BENIGN PROSTATIC HYPERTROPHY
		ALFUZOSIN HYDROCHLORIDE/ UROXATRAL	10MG	1	PRE-STUDY	CONTINUING		BENIGN PROSTATIC HYPERTROPHY
		MULTIVITAMINS/ MULTIVITAMINS	1TAB	1	PRE-STUDY	CONTINUING		SUPPLEMENT
		POTASSIUM IODIDE/ SSKI	12 DROPS	1	16NOV2009	02DEC2009		THYROID BLOCKADE
		LOMOTIL /00034001// LOMOTIL	2.5MG	1	17NOV2009	17NOV2009	2	INTERMITTENT DIARRHEA
		LOMOTIL /00034001// LOMOTIL	2.5MG	1	21NOV2009	24NOV2009	2	INTERMITTENT DIARRHEA
		IBUPROFEN/ IBUPROFEN	200MG	1	24NOV2009	28NOV2009	3	BACKACHE

-- =Date unknown or partially unknown
UNK =Unknown
Note: [a] Route: 1=Oral 2=IV 3=IM 4=Subcutaneous 5=Rectal 6=Transdermal 7=Inhalation 8=Topical 9=Ophthalmic 99=Other
[b] Indicates the AE number if this treatment is being used to treat a recorded AE

Listing 11
Concomitant Medications

Center	Patient	Preferred Term / Investigator Text	Dose/Unit	Route [a]	Start Date	Stop Date	AE No [b]	Indication
XVO_003	201	BACITRACIN/ BACITRACIN	UK	8	21NOV2009	02DEC2009	5	LEFT ELBOW SORE
		DIPHENHYDRAMINE/ DIPHENHYDRAMINE	25MG	1	23NOV2009	24NOV2009	7	NASAL CONGESTION
		PSEUDOEPHEDRINE/ PSEUDOEPHEDRINE	125MG	1	23NOV2009	24NOV2009	7	NASAL CONGESTION
XVO_005	301	LISINOPRIL/ LISINOPRIL	10MG	1	PRE-STUDY	CONTINUING		HIGH BLOOD PRESSURE
		METFORMIN/ METFORMIN	500MG	1	PRE-STUDY	CONTINUING		DIABETES
		OMEPRAZOLE/ PRILOSEC	20MG	1	-----2007	CONTINUING		GASTROESOPHAGEAL REFLUX
		SIMVASTATIN/ SIMVASTIN	40MG	1	-----2009	CONTINUING		HIGH CHOLESTEROL
		VICODIN/ VICODIN PRN	7.5/750MG	1	-----2007	CONTINUING		SCIATICA PAIN
		CLONALIN/ BENADRYL	25MG	1	-----2005	CONTINUING		HIVES
		ENOXAPARIN SODIUM/ LOVENOX	80MG	4	15FEB2007	CONTINUING		PROPHYLAXIS OF DEEP VEIN THROMBOSIS
		CLONALIN/ BENADRYL	50MG	1	03AUG2009	07AUG2009		NASAL DRAINAGE
		POTASSIUM IODIDE/ SATURATED SOLUTION POTASSIUM IODIDE (SSKI)	300MG	1	03AUG2009	18AUG2009		THYROID PROTECTION
		DEXTROMETHORPHAN HYDROBROMIDE/ DEXTROMETHORPHAN HBR	30MG	1	06AUG2009	08SEP2009		COUGH
	302	FISH OIL/ FISH OIL	1000MG	1	PRE-STUDY	CONTINUING		HYPERCHOLESTEROLEMIA

-- =Date unknown or partially unknown
UNK =Unknown
Note: [a] Route: 1=Oral 2=IV 3=IM 4=Subcutaneous 5=Rectal 6=Transdermal 7=Inhalation 8=Topical 9=Ophthalmic 99=Other
[b] Indicates the AE number if this treatment is being used to treat a recorded AE

Listing 11
Concomitant Medications

Center	Patient	Preferred Term / Investigator Text	Dose/Unit	Route [a]	Start Date	Stop Date	AE No [b]	Indication
XVO_005	302	DOCUSATE SODIUM/ EQUATE STOOL SOFTNER	100MG	1	PRE-STUDY	CONTINUING		PROPHYLAXIS
		COTYLENOL/ TYLENOL	500MG	1	PRE-STUDY	CONTINUING		BACK PAIN
		CENTRUM SILVER /06027401// CENTRUM SILVER	UNK	1	22SEP2008	CONTINUING		PROPHYLAXIS
		CALCIUM/ CALCIUM	600MG	1	04FEB2009	CONTINUING		PROPHYLAXIS
		TAMSULOSIN HYDROCHLORIDE/ FLOMAX	0.4MG	1	04FEB2009	CONTINUING		URINARY OBSTRUCTION
		SIMVASTATIN/ SIMVASTIN	10MG	1	04FEB2009	CONTINUING		HYPERCHOLESTEROLEMIA
		ACETYLSALICYLIC ACID/ ASPIRIN	81MG	1	04FEB2009	CONTINUING		PROPHYLAXIS
		RANITIDINE/ RANITIDINE	150MG	1	16FEB2009	CONTINUING		PROPHYLAXIS
		POTASSIUM IODIDE/ SATURATED SOLUTION POTASSIUM IODIDE	300MG	1	14DEC2009	29DEC2009		THYROID PROTECTION
		ZOLEDRONIC ACID/ ZOMETA	4MG	2	15JAN2010	CONTINUING		BONE METASTASIS
XVO_007	401	POTASSIUM IODIDE/ IOSAT	130MG	1	09NOV2009	24NOV2009		THYROID PROTECTION
		CLONALIN/ BENADRYL	50MG	1	10NOV2009	10NOV2009		PREMED
		PREDNISONE/ PREDNISONE	50MG	1	10NOV2009	10NOV2009		PREMED
	402	GABAPENTIN/ GABAPENTIN	400MG	1	--JAN2009	CONTINUING		PAIN

-- =Date unknown or partially unknown
UNK =Unknown
Note: [a] Route: 1=Oral 2=IV 3=IM 4=Subcutaneous 5=Rectal 6=Transdermal 7=Inhalation 8=Topical 9=Ophthalmic 99=Other
[b] Indicates the AE number if this treatment is being used to treat a recorded AE

Listing 11
Concomitant Medications

Center	Patient	Preferred Term / Investigator Text	Dose/Unit	Route [a]	Start Date	Stop Date	AE No [b]	Indication
XVO_007	402	OXYCOCET/ PERCOCET	10/325MG	1	--JAN2009	CONTINUING		PAIN
		DOCUSATE SODIUM/ SENAKOT	1TAB	1	05NOV2009	CONTINUING		STOOL SOFTENER
		MORPHINE SULFATE/ MORPHINE SULFATE	30MG	1	05NOV2009	CONTINUING		PAIN
		POTASSIUM IODIDE/ IOSAT	130MG	1	07DEC2009	22DEC2009		THYROID PROTECTION
		CEFALEXIN/ KEFLEX	500MG	1	19JAN2010	02FEB2010	3	SUPERFICIAL CELLULITIS

-- =Date unknown or partially unknown
UNK =Unknown
Note: [a] Route: 1=Oral 2=IV 3=IM 4=Subcutaneous 5=Rectal 6=Transdermal 7=Inhalation 8=Topical 9=Ophthalmic 99=Other
[b] Indicates the AE number if this treatment is being used to treat a recorded AE

Listing 12
Vital Signs

Center	Patient	Visit	Assessment Date	Parameter	Recorded Result	Recorded Units	Converted Result	Converted Units
XVO_001	101	Screening	09NOV2009	Supine Systolic Blood Pressure	130	mmHg	130.0	mmHg
				Supine Diastolic Blood Pressure	78	mmHg	78.0	mmHg
				Pulse Rate	79	bpm	79.0	bpm
				Temperature	36.5	oC	36.5	oC
				Respiration Rate	17	bpm	17.0	bpm
				Body Weight	80.8	kg	80.8	kg
				Height	165.7	cm	165.7	cm
		Day 0 Pre-dose	10NOV2009	Supine Systolic Blood Pressure	112	mmHg	112.0	mmHg
				Supine Diastolic Blood Pressure	60	mmHg	60.0	mmHg
				Pulse Rate	70	bpm	70.0	bpm
				Temperature	36.6	oC	36.6	oC
				Respiration Rate	20	bpm	20.0	bpm
				SaO2	95	%	95.0	%
		5 Minutes	10NOV2009	Supine Systolic Blood Pressure	116	mmHg	116.0	mmHg
				Supine Diastolic Blood Pressure	60	mmHg	60.0	mmHg
				Pulse Rate	76	bpm	76.0	bpm
				Temperature	36.7	oC	36.7	oC
				Respiration Rate	20	bpm	20.0	bpm
				SaO2	97	%	97.0	%
		15 Minutes	10NOV2009	Supine Systolic Blood Pressure	116	mmHg	116.0	mmHg
				Supine Diastolic Blood Pressure	60	mmHg	60.0	mmHg
				Pulse Rate	81	bpm	81.0	bpm
				Temperature	36.1	oC	36.1	oC
				Respiration Rate	20	bpm	20.0	bpm
				SaO2	96	%	96.0	%

Listing 12
Vital Signs

Center	Patient	Visit	Assessment Date	Parameter	Recorded Result	Recorded Units	Converted Result	Converted Units
XVO_001	101	60 Minutes	10NOV2009	Supine Systolic Blood Pressure	120	mmHg	120.0	mmHg
				Supine Diastolic Blood Pressure	68	mmHg	68.0	mmHg
				Pulse Rate	74	bpm	74.0	bpm
				Temperature	36.8	oC	36.8	oC
				Respiration Rate	20	bpm	20.0	bpm
				SaO2	97	%	97.0	%
		4 - 6 Hours	10NOV2009	Supine Systolic Blood Pressure	114	mmHg	114.0	mmHg
				Supine Diastolic Blood Pressure	56	mmHg	56.0	mmHg
				Pulse Rate	80	bpm	80.0	bpm
				Respiration Rate	20	bpm	20.0	bpm
				SaO2	96	%	96.0	%
		Day 2	12NOV2009	Supine Systolic Blood Pressure	122	mmHg	122.0	mmHg
				Supine Diastolic Blood Pressure	71	mmHg	71.0	mmHg
				Pulse Rate	70	bpm	70.0	bpm
				Temperature	35.8	oC	35.8	oC
				Respiration Rate	12	bpm	12.0	bpm
				SaO2	95	%	95.0	%
		Day 3	13NOV2009	Supine Systolic Blood Pressure	101	mmHg	101.0	mmHg
				Supine Diastolic Blood Pressure	55	mmHg	55.0	mmHg
				Pulse Rate	71	bpm	71.0	bpm
				Temperature	35.9	oC	35.9	oC
				Respiration Rate	17	bpm	17.0	bpm
		Day 6	16NOV2009	Supine Systolic Blood Pressure	124	mmHg	124.0	mmHg
				Supine Diastolic Blood Pressure	66	mmHg	66.0	mmHg
				Pulse Rate	79	bpm	79.0	bpm

Listing 12
Vital Signs

Center	Patient	Visit	Assessment Date	Parameter	Recorded Result	Recorded Units	Converted Result	Converted Units
XVO_001	101	Day 6	16NOV2009	Temperature	36.8	oC	36.8	oC
				Respiration Rate	17	bpm	17.0	bpm
				SaO2	97	%	97.0	%
		Day 14	24NOV2009	Supine Systolic Blood Pressure	140	mmHg	140.0	mmHg
				Supine Diastolic Blood Pressure	64	mmHg	64.0	mmHg
				Pulse Rate	67	bpm	67.0	bpm
				Temperature	35.7	oC	35.7	oC
				Respiration Rate	17	bpm	17.0	bpm
				SaO2	99	%	99.0	%
				Body Weight	81.3	kg	81.3	kg
		Day 30	11DEC2009	Supine Systolic Blood Pressure	148	mmHg	148.0	mmHg
				Supine Diastolic Blood Pressure	71	mmHg	71.0	mmHg
				Pulse Rate	81	bpm	81.0	bpm
				Temperature	35.6	oC	35.6	oC
				Respiration Rate	17	bpm	17.0	bpm
		Day 42	05JAN2010	Supine Systolic Blood Pressure	124	mmHg	124.0	mmHg
				Supine Diastolic Blood Pressure	70	mmHg	70.0	mmHg
				Pulse Rate	87	bpm	87.0	bpm
				Temperature	36.6	oC	36.6	oC
				Respiration Rate	17	bpm	17.0	bpm
	102	Screening	11DEC2009	Supine Systolic Blood Pressure	143	mmHg	143.0	mmHg
				Supine Diastolic Blood Pressure	84	mmHg	84.0	mmHg
				Pulse Rate	107	bpm	107.0	bpm
				Temperature	36.6	oC	36.6	oC
				Respiration Rate	18	bpm	18.0	bpm

Listing 12
Vital Signs

Center	Patient	Visit	Assessment Date	Parameter	Recorded Result	Recorded Units	Converted Result	Converted Units
XVO_001	102	Screening	11DEC2009	SaO2	98	%	98.0	%
				Body Weight	143.7	kg	143.7	kg
				Height	172.2	cm	172.2	cm
		Day 0 Pre-dose	15DEC2009	Supine Systolic Blood Pressure	125	mmHg	125.0	mmHg
				Supine Diastolic Blood Pressure	73	mmHg	73.0	mmHg
				Pulse Rate	101	bpm	101.0	bpm
				Temperature	36.3	oC	36.3	oC
				Respiration Rate	14	bpm	14.0	bpm
				SaO2	96	%	96.0	%
		5 Minutes	15DEC2009	Supine Systolic Blood Pressure	120	mmHg	120.0	mmHg
				Supine Diastolic Blood Pressure	71	mmHg	71.0	mmHg
				Pulse Rate	91	bpm	91.0	bpm
				Temperature	36.6	oC	36.6	oC
				Respiration Rate	16	bpm	16.0	bpm
				SaO2	95	%	95.0	%
		15 Minutes	15DEC2009	Supine Systolic Blood Pressure	138	mmHg	138.0	mmHg
				Supine Diastolic Blood Pressure	88	mmHg	88.0	mmHg
				Pulse Rate	101	bpm	101.0	bpm
				Temperature	36.7	oC	36.7	oC
				Respiration Rate	16	bpm	16.0	bpm
				SaO2	93	%	93.0	%
		30 Minutes	15DEC2009	Supine Systolic Blood Pressure	130	mmHg	130.0	mmHg
				Supine Diastolic Blood Pressure	79	mmHg	79.0	mmHg
				Pulse Rate	102	bpm	102.0	bpm
				Temperature	36.3	oC	36.3	oC

Listing 12
Vital Signs

Center	Patient	Visit	Assessment Date	Parameter	Recorded Result	Recorded Units	Converted Result	Converted Units
XVO_001	102	30 Minutes	15DEC2009	Respiration Rate	15	bpm	15.0	bpm
				SaO2	93	%	93.0	%
		60 Minutes	15DEC2009	Supine Systolic Blood Pressure	125	mmHg	125.0	mmHg
				Supine Diastolic Blood Pressure	80	mmHg	80.0	mmHg
				Pulse Rate	96	bpm	96.0	bpm
				Temperature	36.4	oC	36.4	oC
				Respiration Rate	16	bpm	16.0	bpm
				SaO2	95	%	95.0	%
		4 - 6 Hours	15DEC2009	Supine Systolic Blood Pressure	129	mmHg	129.0	mmHg
				Supine Diastolic Blood Pressure	89	mmHg	89.0	mmHg
				Pulse Rate	104	bpm	104.0	bpm
				Temperature	35.9	oC	35.9	oC
				Respiration Rate	14	bpm	14.0	bpm
				SaO2	97	%	97.0	%
		Day 1	16DEC2009	Supine Systolic Blood Pressure	128	mmHg	128.0	mmHg
				Supine Diastolic Blood Pressure	84	mmHg	84.0	mmHg
				Pulse Rate	96	bpm	96.0	bpm
				Temperature	35.9	oC	35.9	oC
				Respiration Rate	16	bpm	16.0	bpm
				SaO2	99	%	99.0	%
		Day 2	17DEC2009	Supine Systolic Blood Pressure	128	mmHg	128.0	mmHg
				Supine Diastolic Blood Pressure	70	mmHg	70.0	mmHg
				Pulse Rate	116	bpm	116.0	bpm
				Temperature	36.1	oC	36.1	oC
				Respiration Rate	16	bpm	16.0	bpm

Listing 12
Vital Signs

Center	Patient	Visit	Assessment Date	Parameter	Recorded Result	Recorded Units	Converted Result	Converted Units
XVO_001	102	Day 2	17DEC2009	SaO2	98	%	98.0	%
		Day 3	18DEC2009	Supine Systolic Blood Pressure	129	mmHg	129.0	mmHg
				Supine Diastolic Blood Pressure	76	mmHg	76.0	mmHg
				Pulse Rate	89	bpm	89.0	bpm
				Temperature	35.8	oC	35.8	oC
				Respiration Rate	17	bpm	17.0	bpm
				SaO2	95	%	95.0	%
		Day 6	21DEC2009	Supine Systolic Blood Pressure	131	mmHg	131.0	mmHg
				Supine Diastolic Blood Pressure	88	mmHg	88.0	mmHg
				Pulse Rate	94	bpm	94.0	bpm
				Temperature	36.3	oC	36.3	oC
				Respiration Rate	17	bpm	17.0	bpm
				SaO2	100	%	100.0	%
		Day 14	29DEC2009	Supine Systolic Blood Pressure	121	mmHg	121.0	mmHg
				Supine Diastolic Blood Pressure	70	mmHg	70.0	mmHg
				Pulse Rate	94	bpm	94.0	bpm
				Temperature	36.8	oC	36.8	oC
				Respiration Rate	18	bpm	18.0	bpm
				SaO2	97	%	97.0	%
				Body Weight	172.2	kg	172.2	kg
		Day 30	15JAN2010	Supine Systolic Blood Pressure	127	mmHg	127.0	mmHg
				Supine Diastolic Blood Pressure	75	mmHg	75.0	mmHg
				Pulse Rate	87	bpm	87.0	bpm
				Temperature	35.7	oC	35.7	oC
				Respiration Rate	16	bpm	16.0	bpm

Listing 12
Vital Signs

Center	Patient	Visit	Assessment Date	Parameter	Recorded Result	Recorded Units	Converted Result	Converted Units
XVO_001	102	Day 30	15JAN2010	SaO2	99	%	99.0	%
		Day 42	29JAN2010	Supine Systolic Blood Pressure	139	mmHg	139.0	mmHg
				Supine Diastolic Blood Pressure	79	mmHg	79.0	mmHg
				Pulse Rate	86	bpm	86.0	bpm
				Temperature	35.4	oC	35.4	oC
				Respiration Rate	17	bpm	17.0	bpm
				SaO2	98	%	98.0	%
				Body Weight	140	kg	140.0	kg
	103	Screening	11JAN2010	Supine Systolic Blood Pressure	109	mmHg	109.0	mmHg
				Supine Diastolic Blood Pressure	76	mmHg	76.0	mmHg
				Pulse Rate	56	bpm	56.0	bpm
				Temperature	36.1	oC	36.1	oC
				Respiration Rate	16	bpm	16.0	bpm
				SaO2	97	%	97.0	%
				Body Weight	86.6	kg	86.6	kg
				Height	170	cm	170.0	cm
		Day 0 Pre-dose	12JAN2010	Supine Systolic Blood Pressure	110	mmHg	110.0	mmHg
				Supine Diastolic Blood Pressure	75	mmHg	75.0	mmHg
				Pulse Rate	59	bpm	59.0	bpm
				Temperature	35.7	oC	35.7	oC
				Respiration Rate	18	bpm	18.0	bpm
				SaO2	95	%	95.0	%
				Body Weight	87	kg	87.0	kg
		5 Minutes	12JAN2010	Supine Systolic Blood Pressure	126	mmHg	126.0	mmHg
				Supine Diastolic Blood Pressure	82	mmHg	82.0	mmHg

Listing 12
Vital Signs

Center	Patient	Visit	Assessment Date	Parameter	Recorded Result	Recorded Units	Converted Result	Converted Units
XVO_001	103	5 Minutes	12JAN2010	Pulse Rate	62	bpm	62.0	bpm
				Temperature	36.4	oC	36.4	oC
				Respiration Rate	16	bpm	16.0	bpm
				SaO2	97	%	97.0	%
		15 Minutes	12JAN2010	Supine Systolic Blood Pressure	122	mmHg	122.0	mmHg
				Supine Diastolic Blood Pressure	74	mmHg	74.0	mmHg
				Pulse Rate	63	bpm	63.0	bpm
				Temperature	36.3	oC	36.3	oC
				Respiration Rate	15	bpm	15.0	bpm
				SaO2	99	%	99.0	%
		30 Minutes	12JAN2010	Supine Systolic Blood Pressure	117	mmHg	117.0	mmHg
				Supine Diastolic Blood Pressure	81	mmHg	81.0	mmHg
				Pulse Rate	65	bpm	65.0	bpm
				Temperature	36.3	oC	36.3	oC
				Respiration Rate	16	bpm	16.0	bpm
				SaO2	97	%	97.0	%
		60 Minutes	12JAN2010	Supine Systolic Blood Pressure	125	mmHg	125.0	mmHg
				Supine Diastolic Blood Pressure	78	mmHg	78.0	mmHg
				Pulse Rate	52	bpm	52.0	bpm
				Temperature	36.5	oC	36.5	oC
				Respiration Rate	14	bpm	14.0	bpm
				SaO2	97	%	97.0	%
		4 - 6 Hours	12JAN2010	Supine Systolic Blood Pressure	108	mmHg	108.0	mmHg
				Supine Diastolic Blood Pressure	69	mmHg	69.0	mmHg
				Pulse Rate	67	bpm	67.0	bpm

Listing 12
Vital Signs

Center	Patient	Visit	Assessment Date	Parameter	Recorded Result	Recorded Units	Converted Result	Converted Units
XVO_001	103	4 - 6 Hours	12JAN2010	Temperature	36.3	oC	36.3	oC
				Respiration Rate	14	bpm	14.0	bpm
				SaO2	97	%	97.0	%
		Day 1	13JAN2010	Supine Systolic Blood Pressure	117	mmHg	117.0	mmHg
				Supine Diastolic Blood Pressure	82	mmHg	82.0	mmHg
				Pulse Rate	55	bpm	55.0	bpm
				Temperature	36.3	oC	36.3	oC
				Respiration Rate	15	bpm	15.0	bpm
				SaO2	96	%	96.0	%
		Day 2	14JAN2010	Supine Systolic Blood Pressure	117	mmHg	117.0	mmHg
				Supine Diastolic Blood Pressure	75	mmHg	75.0	mmHg
				Pulse Rate	57	bpm	57.0	bpm
				Temperature	36.3	oC	36.3	oC
				Respiration Rate	14	bpm	14.0	bpm
				SaO2	95	%	95.0	%
		Day 3	15JAN2010	Supine Systolic Blood Pressure	107	mmHg	107.0	mmHg
				Supine Diastolic Blood Pressure	65	mmHg	65.0	mmHg
				Pulse Rate	65	bpm	65.0	bpm
				Temperature	36.2	oC	36.2	oC
				Respiration Rate	16	bpm	16.0	bpm
				SaO2	96	%	96.0	%
		Day 6	19JAN2010	Supine Systolic Blood Pressure	107	mmHg	107.0	mmHg
				Supine Diastolic Blood Pressure	64	mmHg	64.0	mmHg
				Pulse Rate	52	bpm	52.0	bpm
				Temperature	36.5	oC	36.5	oC

Listing 12
Vital Signs

Center	Patient	Visit	Assessment Date	Parameter	Recorded Result	Recorded Units	Converted Result	Converted Units
XVO_001	103	Day 6	19JAN2010	Respiration Rate	16	bpm	16.0	bpm
				SaO2	94	%	94.0	%
		Day 14	26JAN2010	Supine Systolic Blood Pressure	111	mmHg	111.0	mmHg
				Supine Diastolic Blood Pressure	76	mmHg	76.0	mmHg
				Pulse Rate	70	bpm	70.0	bpm
				Respiration Rate	16	bpm	16.0	bpm
				Body Weight	85.8	kg	85.8	kg
		Day 30	09FEB2010	Supine Systolic Blood Pressure	102	mmHg	102.0	mmHg
				Supine Diastolic Blood Pressure	64	mmHg	64.0	mmHg
				Pulse Rate	61	bpm	61.0	bpm
				Temperature	36.5	oC	36.5	oC
				Respiration Rate	18	bpm	18.0	bpm
		Day 42	19FEB2010	Supine Systolic Blood Pressure	121	mmHg	121.0	mmHg
				Supine Diastolic Blood Pressure	78	mmHg	78.0	mmHg
				Pulse Rate	54	bpm	54.0	bpm
				Temperature	35.2	oC	35.2	oC
				Respiration Rate	18	bpm	18.0	bpm
XVO_003	201	Screening	10NOV2009	Supine Systolic Blood Pressure	138	mmHg	138.0	mmHg
				Supine Diastolic Blood Pressure	80	mmHg	80.0	mmHg
				Pulse Rate	70	bpm	70.0	bpm
				Temperature	98.3	oF	36.8	oC
				Respiration Rate	16	bpm	16.0	bpm
				SaO2	97	%	97.0	%
				Body Weight	134.7	lb	61.1	kg
				Height	66	ins	167.6	cm

Listing 12
Vital Signs

Center	Patient	Visit	Assessment Date	Parameter	Recorded Result	Recorded Units	Converted Result	Converted Units
XVO_003	201	Day 0 Pre-dose	17NOV2009	Supine Systolic Blood Pressure	116	mmHg	116.0	mmHg
				Supine Diastolic Blood Pressure	67	mmHg	67.0	mmHg
				Pulse Rate	80	bpm	80.0	bpm
				Temperature	96.8	oF	36.0	oC
				Respiration Rate	22	bpm	22.0	bpm
				SaO2	98	%	98.0	%
				Body Weight	135	lb	61.2	kg
		5 Minutes	17NOV2009	Supine Systolic Blood Pressure	128	mmHg	128.0	mmHg
				Supine Diastolic Blood Pressure	78	mmHg	78.0	mmHg
				Pulse Rate	74	bpm	74.0	bpm
				Temperature	98.6	oF	37.0	oC
				Respiration Rate	20	bpm	20.0	bpm
				SaO2	98	%	98.0	%
		15 Minutes	17NOV2009	Supine Systolic Blood Pressure	133	mmHg	133.0	mmHg
				Supine Diastolic Blood Pressure	78	mmHg	78.0	mmHg
				Pulse Rate	74	bpm	74.0	bpm
				Temperature	97.8	oF	36.6	oC
				Respiration Rate	24	bpm	24.0	bpm
				SaO2	96	%	96.0	%
		30 Minutes	17NOV2009	Supine Systolic Blood Pressure	139	mmHg	139.0	mmHg
				Supine Diastolic Blood Pressure	82	mmHg	82.0	mmHg
				Pulse Rate	84	bpm	84.0	bpm
				Temperature	97.8	oF	36.6	oC
				Respiration Rate	24	bpm	24.0	bpm
				SaO2	97	%	97.0	%

Listing 12
Vital Signs

Center	Patient	Visit	Assessment Date	Parameter	Recorded Result	Recorded Units	Converted Result	Converted Units
XVO_003	201	60 Minutes	17NOV2009	Supine Systolic Blood Pressure	147	mmHg	147.0	mmHg
				Supine Diastolic Blood Pressure	80	mmHg	80.0	mmHg
				Pulse Rate	74	bpm	74.0	bpm
				Temperature	97.8	oF	36.6	oC
				Respiration Rate	20	bpm	20.0	bpm
				SaO2	97	%	97.0	%
		4 - 6 Hours	17NOV2009	Supine Systolic Blood Pressure	107	mmHg	107.0	mmHg
				Supine Diastolic Blood Pressure	62	mmHg	62.0	mmHg
				Pulse Rate	85	bpm	85.0	bpm
				Temperature	97.5	oF	36.4	oC
				Respiration Rate	20	bpm	20.0	bpm
				SaO2	97	%	97.0	%
		Day 1	18NOV2009	Supine Systolic Blood Pressure	134	mmHg	134.0	mmHg
				Supine Diastolic Blood Pressure	79	mmHg	79.0	mmHg
				Pulse Rate	90	bpm	90.0	bpm
				Temperature	36.3	oC	36.3	oC
				Respiration Rate	18	bpm	18.0	bpm
				SaO2	98	%	98.0	%
		Day 2	19NOV2009	Supine Systolic Blood Pressure	129	mmHg	129.0	mmHg
				Supine Diastolic Blood Pressure	76	mmHg	76.0	mmHg
				Pulse Rate	90	bpm	90.0	bpm
				Temperature	98.2	oF	36.8	oC
				Respiration Rate	20	bpm	20.0	bpm
				SaO2	97	%	97.0	%
		Day 3	20NOV2009	Supine Systolic Blood Pressure	105	mmHg	105.0	mmHg

Listing 12
Vital Signs

Center	Patient	Visit	Assessment Date	Parameter	Recorded Result	Recorded Units	Converted Result	Converted Units
XVO_003	201	Day 3	20NOV2009	Supine Diastolic Blood Pressure	69	mmHg	69.0	mmHg
				Pulse Rate	91	bpm	91.0	bpm
				Temperature	97.5	oF	36.4	oC
				SaO2	97	%	97.0	%
		Day 6	23NOV2009	Supine Systolic Blood Pressure	120	mmHg	120.0	mmHg
				Supine Diastolic Blood Pressure	75	mmHg	75.0	mmHg
				Pulse Rate	92	bpm	92.0	bpm
				Temperature	97.5	oF	36.4	oC
				Respiration Rate	18	bpm	18.0	bpm
				SaO2	98	%	98.0	%
		Day 14	02DEC2009	Supine Systolic Blood Pressure	95	mmHg	95.0	mmHg
				Supine Diastolic Blood Pressure	88	mmHg	88.0	mmHg
				Pulse Rate	101	bpm	101.0	bpm
				Temperature	98	oF	36.7	oC
				Respiration Rate	20	bpm	20.0	bpm
				SaO2	97	%	97.0	%
				Body Weight	136.4	lb	61.9	kg
		Day 30	22DEC2009	Supine Systolic Blood Pressure	141	mmHg	141.0	mmHg
				Supine Diastolic Blood Pressure	82	mmHg	82.0	mmHg
				Pulse Rate	100	bpm	100.0	bpm
				Temperature	96.8	oF	36.0	oC
				Respiration Rate	20	bpm	20.0	bpm
				SaO2	97	%	97.0	%
		Day 42	29DEC2009	Supine Systolic Blood Pressure	110	mmHg	110.0	mmHg
				Supine Diastolic Blood Pressure	75	mmHg	75.0	mmHg

Listing 12
Vital Signs

Center	Patient	Visit	Assessment Date	Parameter	Recorded Result	Recorded Units	Converted Result	Converted Units
XVO_003	201	Day 42	29DEC2009	Pulse Rate	108	bpm	108.0	bpm
				Temperature	98.2	oF	36.8	oC
				Respiration Rate	22	bpm	22.0	bpm
				SaO2	96	%	96.0	%
XVO_005	301	Screening	03AUG2009	Supine Systolic Blood Pressure	115	mmHg	115.0	mmHg
				Supine Diastolic Blood Pressure	70	mmHg	70.0	mmHg
				Pulse Rate	67	bpm	67.0	bpm
				Temperature	98.3	oF	36.8	oC
				Respiration Rate	20	bpm	20.0	bpm
				SaO2	97	%	97.0	%
				Body Weight	270.8	lb	122.8	kg
				Height	176	cm	176.0	cm
		Day 0 Pre-dose	04AUG2009	Supine Systolic Blood Pressure	136	mmHg	136.0	mmHg
				Supine Diastolic Blood Pressure	69	mmHg	69.0	mmHg
				Pulse Rate	76	bpm	76.0	bpm
				Temperature	99.1	oF	37.3	oC
				Respiration Rate	20	bpm	20.0	bpm
				SaO2	97	%	97.0	%
				Body Weight	270	lb	122.5	kg
		5 Minutes	04AUG2009	Supine Systolic Blood Pressure	121	mmHg	121.0	mmHg
				Supine Diastolic Blood Pressure	78	mmHg	78.0	mmHg
				Pulse Rate	68	bpm	68.0	bpm
				Temperature	99.1	oF	37.3	oC
				Respiration Rate	24	bpm	24.0	bpm
				SaO2	98	%	98.0	%

Listing 12
Vital Signs

Center	Patient	Visit	Assessment Date	Parameter	Recorded Result	Recorded Units	Converted Result	Converted Units
XVO_005	301	15 Minutes	04AUG2009	Supine Systolic Blood Pressure	126	mmHg	126.0	mmHg
				Supine Diastolic Blood Pressure	87	mmHg	87.0	mmHg
				Pulse Rate	69	bpm	69.0	bpm
				Temperature	99.5	oF	37.5	oC
				Respiration Rate	20	bpm	20.0	bpm
				SaO2	97	%	97.0	%
		60 Minutes	04AUG2009	Supine Systolic Blood Pressure	128	mmHg	128.0	mmHg
				Supine Diastolic Blood Pressure	82	mmHg	82.0	mmHg
				Pulse Rate	65	bpm	65.0	bpm
				Temperature	99.1	oF	37.3	oC
				Respiration Rate	20	bpm	20.0	bpm
				SaO2	97	%	97.0	%
		4 - 6 Hours	04AUG2009	Supine Systolic Blood Pressure	122	mmHg	122.0	mmHg
				Supine Diastolic Blood Pressure	80	mmHg	80.0	mmHg
				Pulse Rate	79	bpm	79.0	bpm
				Temperature	98.9	oF	37.2	oC
				Respiration Rate	20	bpm	20.0	bpm
				SaO2	98	%	98.0	%
		Day 1	05AUG2009	Supine Systolic Blood Pressure	118	mmHg	118.0	mmHg
				Supine Diastolic Blood Pressure	81	mmHg	81.0	mmHg
				Pulse Rate	77	bpm	77.0	bpm
				Temperature	98.8	oF	37.1	oC
				SaO2	98	%	98.0	%
		Day 2	06AUG2009	Supine Systolic Blood Pressure	120	mmHg	120.0	mmHg
				Supine Diastolic Blood Pressure	73	mmHg	73.0	mmHg

Listing 12
Vital Signs

Center	Patient	Visit	Assessment Date	Parameter	Recorded Result	Recorded Units	Converted Result	Converted Units
XVO_005	301	Day 2	06AUG2009	Pulse Rate	75	bpm	75.0	bpm
				Temperature	97.8	oF	36.6	oC
				Respiration Rate	24	bpm	24.0	bpm
				SaO2	97	%	97.0	%
		Day 3	07AUG2009	Supine Systolic Blood Pressure	122	mmHg	122.0	mmHg
				Supine Diastolic Blood Pressure	78	mmHg	78.0	mmHg
				Pulse Rate	78	bpm	78.0	bpm
				Temperature	97.9	oF	36.6	oC
				Respiration Rate	20	bpm	20.0	bpm
				SaO2	97	%	97.0	%
		Day 6	10AUG2009	Supine Systolic Blood Pressure	116	mmHg	116.0	mmHg
				Supine Diastolic Blood Pressure	84	mmHg	84.0	mmHg
				Pulse Rate	81	bpm	81.0	bpm
				Temperature	99.1	oF	37.3	oC
				Respiration Rate	20	bpm	20.0	bpm
				SaO2	96	%	96.0	%
		Day 14	18AUG2009	Supine Systolic Blood Pressure	119	mmHg	119.0	mmHg
				Supine Diastolic Blood Pressure	79	mmHg	79.0	mmHg
				Pulse Rate	70	bpm	70.0	bpm
				Temperature	97.9	oF	36.6	oC
				Respiration Rate	20	bpm	20.0	bpm
				SaO2	96	%	96.0	%
				Body Weight	272	lb	123.4	kg
		Day 30	04SEP2009	Supine Systolic Blood Pressure	102	mmHg	102.0	mmHg
				Supine Diastolic Blood Pressure	59	mmHg	59.0	mmHg

Listing 12
Vital Signs

Center	Patient	Visit	Assessment Date	Parameter	Recorded Result	Recorded Units	Converted Result	Converted Units
XVO_005	301	Day 30	04SEP2009	Pulse Rate	84	bpm	84.0	bpm
				Temperature	98.6	oF	37.0	oC
				Respiration Rate	24	bpm	24.0	bpm
				SaO2	96	%	96.0	%
		Day 42	15SEP2009	Supine Systolic Blood Pressure	129	mmHg	129.0	mmHg
				Supine Diastolic Blood Pressure	75	mmHg	75.0	mmHg
				Pulse Rate	75	bpm	75.0	bpm
				Temperature	98.6	oF	37.0	oC
				Respiration Rate	18	bpm	18.0	bpm
				SaO2	97	%	97.0	%
	302	Screening	14DEC2009	Supine Systolic Blood Pressure	135	mmHg	135.0	mmHg
				Supine Diastolic Blood Pressure	70	mmHg	70.0	mmHg
				Pulse Rate	64	bpm	64.0	bpm
				Temperature	97.9	oF	36.6	oC
				Respiration Rate	15	bpm	15.0	bpm
				SaO2	95	%	95.0	%
				Body Weight	81.3	kg	81.3	kg
				Height	170.4	cm	170.4	cm
		Day 0 Pre-dose	15DEC2009	Supine Systolic Blood Pressure	137	mmHg	137.0	mmHg
				Supine Diastolic Blood Pressure	66	mmHg	66.0	mmHg
				Pulse Rate	65	bpm	65.0	bpm
				Temperature	98.8	oF	37.1	oC
				Respiration Rate	20	bpm	20.0	bpm
				SaO2	94	%	94.0	%
				Body Weight	180	lb	81.6	kg

Listing 12
Vital Signs

Center	Patient	Visit	Assessment Date	Parameter	Recorded Result	Recorded Units	Converted Result	Converted Units
XVO_005	302	5 Minutes	15DEC2009	Supine Systolic Blood Pressure	123	mmHg	123.0	mmHg
				Supine Diastolic Blood Pressure	67	mmHg	67.0	mmHg
				Pulse Rate	61	bpm	61.0	bpm
				Temperature	98.1	oF	36.7	oC
				Respiration Rate	20	bpm	20.0	bpm
				SaO2	92	%	92.0	%
		15 Minutes	15DEC2009	Supine Systolic Blood Pressure	107	mmHg	107.0	mmHg
				Supine Diastolic Blood Pressure	67	mmHg	67.0	mmHg
				Pulse Rate	61	bpm	61.0	bpm
				Temperature	97.8	oF	36.6	oC
				Respiration Rate	18	bpm	18.0	bpm
				SaO2	93	%	93.0	%
		60 Minutes	15DEC2009	Supine Systolic Blood Pressure	125	mmHg	125.0	mmHg
				Supine Diastolic Blood Pressure	71	mmHg	71.0	mmHg
				Pulse Rate	61	bpm	61.0	bpm
				Temperature	98.1	oF	36.7	oC
				Respiration Rate	18	bpm	18.0	bpm
				SaO2	96	%	96.0	%
		4 - 6 Hours	15DEC2009	Supine Systolic Blood Pressure	125	mmHg	125.0	mmHg
				Supine Diastolic Blood Pressure	64	mmHg	64.0	mmHg
				Pulse Rate	71	bpm	71.0	bpm
				Temperature	98	oF	36.7	oC
				Respiration Rate	16	bpm	16.0	bpm
				SaO2	95	%	95.0	%
		Day 1	16DEC2009	Supine Systolic Blood Pressure	134	mmHg	134.0	mmHg

Listing 12
Vital Signs

Center	Patient	Visit	Assessment Date	Parameter	Recorded Result	Recorded Units	Converted Result	Converted Units
XVO_005	302	Day 1	16DEC2009	Supine Diastolic Blood Pressure	71	mmHg	71.0	mmHg
				Pulse Rate	72	bpm	72.0	bpm
				Temperature	99.3	oF	37.4	oC
				Respiration Rate	20	bpm	20.0	bpm
				SaO2	95	%	95.0	%
		Day 2	17DEC2009	Supine Systolic Blood Pressure	139	mmHg	139.0	mmHg
				Supine Diastolic Blood Pressure	72	mmHg	72.0	mmHg
				Pulse Rate	71	bpm	71.0	bpm
				Temperature	98.9	oF	37.2	oC
				Respiration Rate	20	bpm	20.0	bpm
				SaO2	96	%	96.0	%
		Day 3	18DEC2009	Supine Systolic Blood Pressure	153	mmHg	153.0	mmHg
				Supine Diastolic Blood Pressure	78	mmHg	78.0	mmHg
				Pulse Rate	67	bpm	67.0	bpm
				Temperature	97.8	oF	36.6	oC
				Respiration Rate	18	bpm	18.0	bpm
				SaO2	96	%	96.0	%
		Day 6	21DEC2009	Supine Systolic Blood Pressure	138	mmHg	138.0	mmHg
				Supine Diastolic Blood Pressure	73	mmHg	73.0	mmHg
				Pulse Rate	67	bpm	67.0	bpm
				Temperature	98.5	oF	36.9	oC
				Respiration Rate	18	bpm	18.0	bpm
				SaO2	96	%	96.0	%
		Day 14	29DEC2009	Supine Systolic Blood Pressure	141	mmHg	141.0	mmHg
				Supine Diastolic Blood Pressure	76	mmHg	76.0	mmHg

Listing 12
Vital Signs

Center	Patient	Visit	Assessment Date	Parameter	Recorded Result	Recorded Units	Converted Result	Converted Units
XVO_005	302	Day 14	29DEC2009	Pulse Rate	66	bpm	66.0	bpm
				Temperature	98.2	oF	36.8	oC
				Respiration Rate	18	bpm	18.0	bpm
				SaO2	95	%	95.0	%
				Body Weight	178	lb	80.7	kg
		Day 30	15JAN2010	Supine Systolic Blood Pressure	138	mmHg	138.0	mmHg
				Supine Diastolic Blood Pressure	75	mmHg	75.0	mmHg
				Pulse Rate	65	bpm	65.0	bpm
				Temperature	98.3	oF	36.8	oC
				Respiration Rate	20	bpm	20.0	bpm
				SaO2	95	%	95.0	%
		Day 42	25JAN2010	Supine Systolic Blood Pressure	147	mmHg	147.0	mmHg
				Supine Diastolic Blood Pressure	72	mmHg	72.0	mmHg
				Pulse Rate	65	bpm	65.0	bpm
				Temperature	97.8	oF	36.6	oC
				Respiration Rate	18	bpm	18.0	bpm
				SaO2	97	%	97.0	%
XVO_007	401	Screening	04NOV2009	Supine Systolic Blood Pressure	148	mmHg	148.0	mmHg
				Supine Diastolic Blood Pressure	76	mmHg	76.0	mmHg
				Pulse Rate	74	bpm	74.0	bpm
				Temperature	36.8	oC	36.8	oC
				Respiration Rate	20	bpm	20.0	bpm
				SaO2	95	%	95.0	%
				Body Weight	209	lb	94.8	kg
				Height	152.4	cm	152.4	cm

Listing 12
Vital Signs

Center	Patient	Visit	Assessment Date	Parameter	Recorded Result	Recorded Units	Converted Result	Converted Units
XVO_007	401	Day 0 Pre-dose	10NOV2009	Supine Systolic Blood Pressure	141	mmHg	141.0	mmHg
				Supine Diastolic Blood Pressure	77	mmHg	77.0	mmHg
				Pulse Rate	81	bpm	81.0	bpm
				Temperature	36.8	oC	36.8	oC
				Respiration Rate	24	bpm	24.0	bpm
				SaO2	98	%	98.0	%
				Body Weight	94.5	kg	94.5	kg
		5 Minutes	10NOV2009	Supine Systolic Blood Pressure	156	mmHg	156.0	mmHg
				Supine Diastolic Blood Pressure	85	mmHg	85.0	mmHg
				Pulse Rate	83	bpm	83.0	bpm
				Temperature	36.7	oC	36.7	oC
				SaO2	97	%	97.0	%
		15 Minutes	10NOV2009	Supine Systolic Blood Pressure	157	mmHg	157.0	mmHg
				Supine Diastolic Blood Pressure	81	mmHg	81.0	mmHg
				Pulse Rate	84	bpm	84.0	bpm
				Temperature	36.7	oC	36.7	oC
				SaO2	97	%	97.0	%
		30 Minutes	10NOV2009	Supine Systolic Blood Pressure	148	mmHg	148.0	mmHg
				Supine Diastolic Blood Pressure	75	mmHg	75.0	mmHg
				Pulse Rate	71	bpm	71.0	bpm
				Temperature	36.6	oC	36.6	oC
				Respiration Rate	26	bpm	26.0	bpm
				SaO2	97	%	97.0	%
		60 Minutes	10NOV2009	Supine Systolic Blood Pressure	141	mmHg	141.0	mmHg
				Supine Diastolic Blood Pressure	68	mmHg	68.0	mmHg

Listing 12
Vital Signs

Center	Patient	Visit	Assessment Date	Parameter	Recorded Result	Recorded Units	Converted Result	Converted Units
XVO_007	401	60 Minutes	10NOV2009	Pulse Rate	76	bpm	76.0	bpm
				Temperature	36.6	oC	36.6	oC
				Respiration Rate	22	bpm	22.0	bpm
				SaO2	98	%	98.0	%
		4 - 6 Hours	10NOV2009	Supine Systolic Blood Pressure	140	mmHg	140.0	mmHg
				Supine Diastolic Blood Pressure	72	mmHg	72.0	mmHg
				Pulse Rate	77	bpm	77.0	bpm
				Temperature	36.6	oC	36.6	oC
				Respiration Rate	22	bpm	22.0	bpm
				SaO2	97	%	97.0	%
		Day 1	11NOV2009	Supine Systolic Blood Pressure	141	mmHg	141.0	mmHg
				Supine Diastolic Blood Pressure	67	mmHg	67.0	mmHg
				Pulse Rate	62	bpm	62.0	bpm
				Temperature	36.3	oC	36.3	oC
				Respiration Rate	20	bpm	20.0	bpm
				SaO2	97	%	97.0	%
		Day 2	12NOV2009	Supine Systolic Blood Pressure	129	mmHg	129.0	mmHg
				Supine Diastolic Blood Pressure	71	mmHg	71.0	mmHg
				Pulse Rate	73	bpm	73.0	bpm
				Temperature	36.7	oC	36.7	oC
				Respiration Rate	22	bpm	22.0	bpm
				SaO2	97	%	97.0	%
		Day 3	13NOV2009	Supine Systolic Blood Pressure	133	mmHg	133.0	mmHg
				Supine Diastolic Blood Pressure	77	mmHg	77.0	mmHg
				Pulse Rate	72	bpm	72.0	bpm

Listing 12
Vital Signs

Center	Patient	Visit	Assessment Date	Parameter	Recorded Result	Recorded Units	Converted Result	Converted Units
XVO_007	401	Day 3	13NOV2009	Temperature	36.5	oC	36.5	oC
				Respiration Rate	24	bpm	24.0	bpm
				SaO2	97	%	97.0	%
		Day 6	16NOV2009	Supine Systolic Blood Pressure	131	mmHg	131.0	mmHg
				Supine Diastolic Blood Pressure	69	mmHg	69.0	mmHg
				Pulse Rate	65	bpm	65.0	bpm
				Temperature	36.6	oC	36.6	oC
				Respiration Rate	22	bpm	22.0	bpm
				SaO2	96	%	96.0	%
		Day 14	24NOV2009	Supine Systolic Blood Pressure	147	mmHg	147.0	mmHg
				Supine Diastolic Blood Pressure	67	mmHg	67.0	mmHg
				Pulse Rate	61	bpm	61.0	bpm
				Temperature	36.6	oC	36.6	oC
				Respiration Rate	22	bpm	22.0	bpm
				SaO2	97	%	97.0	%
				Body Weight	95	kg	95.0	kg
		Day 30	08DEC2009	Supine Systolic Blood Pressure	150	mmHg	150.0	mmHg
				Supine Diastolic Blood Pressure	76	mmHg	76.0	mmHg
				Pulse Rate	72	bpm	72.0	bpm
				Temperature	36.7	oC	36.7	oC
				Respiration Rate	20	bpm	20.0	bpm
				SaO2	97	%	97.0	%
		Day 42	22DEC2009	Supine Systolic Blood Pressure	156	mmHg	156.0	mmHg
				Supine Diastolic Blood Pressure	87	mmHg	87.0	mmHg
				Pulse Rate	90	bpm	90.0	bpm

Listing 12
Vital Signs

Center	Patient	Visit	Assessment Date	Parameter	Recorded Result	Recorded Units	Converted Result	Converted Units
XVO_007	401	Day 42	22DEC2009	Temperature	36.6	oC	36.6	oC
				Respiration Rate	18	bpm	18.0	bpm
				SaO2	97	%	97.0	%
	402	Screening	03DEC2009	Supine Systolic Blood Pressure	124	mmHg	124.0	mmHg
				Supine Diastolic Blood Pressure	84	mmHg	84.0	mmHg
				Pulse Rate	60	bpm	60.0	bpm
				Temperature	36.7	oC	36.7	oC
				Respiration Rate	20	bpm	20.0	bpm
				SaO2	99	%	99.0	%
				Body Weight	71.3	kg	71.3	kg
				Height	177.8	cm	177.8	cm
		Day 0 Pre-dose	08DEC2009	Supine Systolic Blood Pressure	117	mmHg	117.0	mmHg
				Supine Diastolic Blood Pressure	83	mmHg	83.0	mmHg
				Pulse Rate	73	bpm	73.0	bpm
				Temperature	36.8	oC	36.8	oC
				Respiration Rate	18	bpm	18.0	bpm
				SaO2	98	%	98.0	%
				Body Weight	70.7	kg	70.7	kg
		5 Minutes	08DEC2009	Supine Systolic Blood Pressure	124	mmHg	124.0	mmHg
				Supine Diastolic Blood Pressure	87	mmHg	87.0	mmHg
				Pulse Rate	58	bpm	58.0	bpm
				Temperature	36.6	oC	36.6	oC
				Respiration Rate	20	bpm	20.0	bpm
				SaO2	98	%	98.0	%
		15 Minutes	08DEC2009	Supine Systolic Blood Pressure	104	mmHg	104.0	mmHg

Listing 12
Vital Signs

Center	Patient	Visit	Assessment Date	Parameter	Recorded Result	Recorded Units	Converted Result	Converted Units
XVO_007	402	15 Minutes	08DEC2009	Supine Diastolic Blood Pressure	68	mmHg	68.0	mmHg
				Pulse Rate	52	bpm	52.0	bpm
				Temperature	36.7	oC	36.7	oC
				Respiration Rate	16	bpm	16.0	bpm
				SaO2	99	%	99.0	%
		30 Minutes	08DEC2009	Supine Systolic Blood Pressure	124	mmHg	124.0	mmHg
				Supine Diastolic Blood Pressure	81	mmHg	81.0	mmHg
				Pulse Rate	53	bpm	53.0	bpm
				Temperature	36.5	oC	36.5	oC
				Respiration Rate	16	bpm	16.0	bpm
				SaO2	97	%	97.0	%
		60 Minutes	08DEC2009	Supine Systolic Blood Pressure	135	mmHg	135.0	mmHg
				Supine Diastolic Blood Pressure	92	mmHg	92.0	mmHg
				Pulse Rate	55	bpm	55.0	bpm
				Temperature	36.3	oC	36.3	oC
				Respiration Rate	16	bpm	16.0	bpm
				SaO2	100	%	100.0	%
		4 - 6 Hours	08DEC2009	Supine Systolic Blood Pressure	125	mmHg	125.0	mmHg
				Supine Diastolic Blood Pressure	85	mmHg	85.0	mmHg
				Pulse Rate	70	bpm	70.0	bpm
				Temperature	36.5	oC	36.5	oC
				Respiration Rate	20	bpm	20.0	bpm
				SaO2	100	%	100.0	%
		Day 1	09DEC2009	Supine Systolic Blood Pressure	135	mmHg	135.0	mmHg
				Supine Diastolic Blood Pressure	86	mmHg	86.0	mmHg

Listing 12
Vital Signs

Center	Patient	Visit	Assessment Date	Parameter	Recorded Result	Recorded Units	Converted Result	Converted Units
XVO_007	402	Day 1	09DEC2009	Pulse Rate	76	bpm	76.0	bpm
				Temperature	36.7	oC	36.7	oC
				Respiration Rate	20	bpm	20.0	bpm
				SaO2	100	%	100.0	%
		Day 2	10DEC2009	Supine Systolic Blood Pressure	123	mmHg	123.0	mmHg
				Supine Diastolic Blood Pressure	86	mmHg	86.0	mmHg
				Pulse Rate	78	bpm	78.0	bpm
				Temperature	36.6	oC	36.6	oC
				Respiration Rate	20	bpm	20.0	bpm
				SaO2	100	%	100.0	%
		Day 3	11DEC2009	Supine Systolic Blood Pressure	121	mmHg	121.0	mmHg
				Supine Diastolic Blood Pressure	81	mmHg	81.0	mmHg
				Pulse Rate	79	bpm	79.0	bpm
				Temperature	36.7	oC	36.7	oC
				Respiration Rate	20	bpm	20.0	bpm
				SaO2	98	%	98.0	%
		Day 6	14DEC2009	Supine Systolic Blood Pressure	126	mmHg	126.0	mmHg
				Supine Diastolic Blood Pressure	84	mmHg	84.0	mmHg
				Pulse Rate	72	bpm	72.0	bpm
				Temperature	36.7	oC	36.7	oC
				Respiration Rate	20	bpm	20.0	bpm
				SaO2	99	%	99.0	%
		Day 14	22DEC2009	Supine Systolic Blood Pressure	117	mmHg	117.0	mmHg
				Supine Diastolic Blood Pressure	74	mmHg	74.0	mmHg
				Pulse Rate	66	bpm	66.0	bpm

Listing 12
Vital Signs

Center	Patient	Visit	Assessment Date	Parameter	Recorded Result	Recorded Units	Converted Result	Converted Units
XVO_007	402	Day 14	22DEC2009	Temperature	36.8	oC	36.8	oC
				Respiration Rate	18	bpm	18.0	bpm
				SaO2	98	%	98.0	%
		Day 30	05JAN2010	Supine Systolic Blood Pressure	125	mmHg	125.0	mmHg
				Supine Diastolic Blood Pressure	89	mmHg	89.0	mmHg
				Pulse Rate	82	bpm	82.0	bpm
				Temperature	36.4	oC	36.4	oC
				Respiration Rate	20	bpm	20.0	bpm
				SaO2	100	%	100.0	%
		Day 42	19JAN2010	Supine Systolic Blood Pressure	125	mmHg	125.0	mmHg
				Supine Diastolic Blood Pressure	86	mmHg	86.0	mmHg
				Pulse Rate	70	bpm	70.0	bpm
				Temperature	36.5	oC	36.5	oC
				Respiration Rate	20	bpm	20.0	bpm
				SaO2	98	%	98.0	%
				Body Weight	78.4	kg	78.4	kg

Listing 13
Electrocardiogram

Center	Patient	Visit	Date Performed	Was Aquired Per Protocol	Interpretation [a]	Clinically Significant Abnormalities
XVO_001	101	Screening	09NOV2009		Normal/within limits	
		Day 0 Pre-dose		Yes		
		5 Minutes		Yes		
		Day 1		Yes		
		Day 6		Yes		
		Day 14		Yes		
	102	Screening	11DEC2009		Abnormal, clinically significant	INFERIOR WALL MYOCARDIAL INFARCTION ; AGE UNDETERMINED
		Day 0 Pre-dose		Yes		
		5 Minutes		Yes		
		Day 1		Yes		
		Day 6		Yes		
		Day 14		Yes		
	103	Screening	11JAN2010		Normal/within limits	
		Day 0 Pre-dose		Yes		
		5 Minutes		Yes		
		Day 1		Yes		
		Day 6		Yes		
		Day 14		Yes		
XVO_003	201	Screening	10NOV2009		Normal/within limits	
		Day 0 Pre-dose		Yes		
		5 Minutes		Yes		
		Day 1		Yes		
		Day 6		Yes		
		Day 14		Yes		

Note: [a] Interpretation is only recorded at screening.

Listing 13
Electrocardiogram

Center	Patient	Visit	Date Performed	Was Aquired Per Protocol	Interpretation [a]	Clinically Significant Abnormalities
XVO_005	301	Screening	03AUG2009		Abnormal, not clinically significant	
		Day 0 Pre-dose		Yes		
		5 Minutes		Yes		
		Day 1		Yes		
		Day 6		Yes		
		Day 14		Yes		
	302	Screening	14DEC2009		Abnormal, not clinically significant	
		Day 0 Pre-dose		Yes		
		5 Minutes		Yes		
		Day 1		Yes		
		Day 6		Yes		
		Day 14		Yes		
XVO_007	401	Screening	04NOV2009		Normal/within limits	
		Day 0 Pre-dose		Yes		
		5 Minutes		Yes		
		Day 1		Yes		
		Day 6		Yes		
		Day 14		Yes		
	402	Screening	03DEC2009		Abnormal, not clinically significant	
		Day 0 Pre-dose		Yes		
		5 Minutes		Yes		
		Day 1		Yes		
		Day 6		Yes		
		Day 14		Yes		

Note: [a] Interpretation is only recorded at screening.

Listing 14
ECOG Performance Status

Center	Patient	Visit	Assessment Date	ECOG Performance Status
XVO_001	101	Screening	09NOV2009	0
		Day 14		0
		Day 30		0
		Day 42		1
	102	Day 0		1
		Day 14		1
		Day 30		0
		Day 42		0
	103	Screening	12JAN2010	0
		Day 0		0
		Day 14		0
		Day 30		0
		Day 42		0
XVO_003	201	Screening	10NOV2009	1
		Day 0		1
		Day 14		1
		Day 30		1
		Day 42		1
XVO_005	301	Screening	03AUG2009	1
		Day 0		1
		Day 14		1
		Day 30		1
		Day 42		1

Listing 14
ECOG Performance Status

Center	Patient	Visit	Assessment Date	ECOG Performance Status
XVO_005	302	Screening	14DEC2009	1
		Day 0		1
		Day 14		1
		Day 30		1
		Day 42		1
XVO_007	401	Screening	10NOV2009	0
		Day 0		0
		Day 14		0
		Day 30		0
		Day 42		0
	402	Screening	03DEC2009	0
		Day 0		0
		Day 14		0
		Day 30		0
		Day 42		0

Listing 15
Pregnancy Test Results

Center	Patient	Visit	Date of Test	Result
XVO_001	101	Day 0		N/A
		Day 42		N/A
	102	Day 0		N/A
		Day 42		N/A
	103	Day 0		N/A
		Day 42		N/A
XVO_003	201	Day 0		N/A
		Day 42		N/A
XVO_005	301	Day 0		N/A
		Day 42		N/A
	302	Day 0		N/A
		Day 42		N/A
XVO_007	401	Day 0		N/A
		Day 42		N/A
	402	Day 0		N/A
		Day 42		N/A

Listing 16
Physical Examination

Center	Patient	Visit	Date Performed	Body System	Result	Specify
XVO_001	101	Screening	09NOV2009	General Appearance	Normal	
				EENT	Normal	
				Head and Neck	Normal	
				Heart	Normal	
				Lungs	Normal	
				Abdomen	Normal	
				Genitourinary	Not Examined	
				Musculoskeletal	Normal	
				Neurological	Normal	
				Dermatological	Normal	
		Day 6		General Appearance	Normal	
				EENT	Normal	
				Head and Neck	Normal	
				Heart	Normal	
				Lungs	Normal	
				Abdomen	Normal	
				Genitourinary	Not Examined	
				Musculoskeletal	Normal	
				Neurological	Normal	
				Dermatological	Normal	
		Day 14		General Appearance	Normal	
				EENT	Normal	
				Head and Neck	Normal	
				Heart	Normal	
				Lungs	Normal	
				Abdomen	Normal	
				Genitourinary	Not Examined	
				Musculoskeletal	Normal	
				Neurological	Normal	
				Dermatological	Normal	

Listing 16
Physical Examination

Center	Patient	Visit	Date Performed	Body System	Result	Specify
XVO_001	101	Day 30		General Appearance	Normal	
				EENT	Normal	
				Head and Neck	Normal	
				Heart	Normal	
				Lungs	Normal	
				Abdomen	Normal	
				Genitourinary	Not Examined	
				Musculoskeletal	Normal	
				Neurological	Normal	
				Dermatological	Normal	
		Day 42		General Appearance	Normal	
				EENT	Normal	
				Head and Neck	Normal	
				Heart	Normal	
				Lungs	Normal	
				Abdomen	Normal	
				Genitourinary	Not Examined	
				Musculoskeletal	Normal	
				Neurological	Normal	
				Dermatological	Normal	
	102	Screening	11DEC2009	General Appearance	Normal	
				EENT	Normal	
				Head and Neck	Normal	
				Heart	Normal	
				Lungs	Normal	
				Abdomen	Normal	
				Genitourinary	Not Examined	
				Musculoskeletal	Normal	
				Neurological	Normal	
				Dermatological	Abnormal	MILD ACNEIFORM RASH 5% CONFLUENCE ANTERIORLY AND POSTERIORLY AT THORAX

Listing 16
Physical Examination

Center	Patient	Visit	Date Performed	Body System	Result	Specify
XVO_001	102	Day 6		General Appearance	Normal	
				EENT	Normal	
				Head and Neck	Normal	
				Heart	Normal	
				Lungs	Normal	
				Abdomen	Normal	
				Genitourinary	Not Examined	
				Musculoskeletal	Normal	
				Neurological	Normal	
				Dermatological	Abnormal	RESOLVED ACNEFORM RASH AT THORAX
		Day 14		General Appearance	Normal	
				EENT	Normal	
				Head and Neck	Normal	
				Heart	Normal	
				Lungs	Normal	
				Abdomen	Normal	
				Genitourinary	Not Examined	
				Musculoskeletal	Normal	
				Neurological	Normal	
				Dermatological	Abnormal	INTEGUMENTARY: HYPERPIGMENTATION SECONDARY TO RESOLVED ACNEIFORM AND CETUXIMAB INDUCED RASH PARONYCHIA ON LEFT BIG TOES
		Day 30		General Appearance	Normal	
				EENT	Normal	
				Head and Neck	Normal	
				Heart	Normal	
				Lungs	Normal	
				Abdomen	Normal	
				Genitourinary	Not Examined	
				Musculoskeletal	Normal	
				Neurological	Normal	

Listing 16
Physical Examination

Center	Patient	Visit	Date Performed	Body System	Result	Specify
XVO_001	102	Day 30		Dermatological	Abnormal	PARONYCHIA ON LEFT BIG TOE RESOLVING
		Day 42		General Appearance	Normal	
				EENT	Normal	
				Head and Neck	Normal	
				Heart	Normal	
				Lungs	Normal	
				Abdomen	Normal	
				Genitourinary	Not Examined	
				Musculoskeletal	Normal	
				Neurological	Normal	
				Dermatological	Abnormal	PARONYCHIA ON LEFT BIG TOE RESOLVING
	103	Screening	12JAN2010	General Appearance	Normal	
				EENT	Normal	
				Head and Neck	Normal	
				Heart	Normal	
				Lungs	Normal	
				Abdomen	Normal	
				Genitourinary	Not Examined	
				Musculoskeletal	Normal	
				Neurological	Normal	
				Dermatological	Normal	
		Day 6		General Appearance	Normal	
				EENT	Normal	
				Head and Neck	Normal	
				Heart	Normal	
				Lungs	Normal	
				Abdomen	Normal	
				Genitourinary	Not Examined	
				Musculoskeletal	Normal	
				Neurological	Normal	
				Dermatological	Normal	

Listing 16
Physical Examination

Center	Patient	Visit	Date Performed	Body System	Result	Specify
XVO_001	103	Day 14		General Appearance	Normal	
				EENT	Normal	
				Head and Neck	Normal	
				Heart	Normal	
				Lungs	Normal	
				Abdomen	Normal	
				Genitourinary	Not Examined	
				Musculoskeletal	Normal	
				Neurological	Normal	
				Dermatological	Normal	
		Day 30		General Appearance	Normal	
				EENT	Normal	
				Head and Neck	Normal	
				Heart	Normal	
				Lungs	Normal	
				Abdomen	Normal	
				Genitourinary	Not Examined	
				Musculoskeletal	Normal	
				Neurological	Normal	
				Dermatological	Normal	
		Day 42		General Appearance	Normal	
				EENT	Normal	
				Head and Neck	Normal	
				Heart	Normal	
				Lungs	Normal	
				Abdomen	Normal	
				Genitourinary	Not Examined	
				Musculoskeletal	Normal	
				Neurological	Normal	
				Dermatological	Normal	
XVO_003	201	Screening	10NOV2009	General Appearance	Normal	

Listing 16
Physical Examination

Center	Patient	Visit	Date Performed	Body System	Result	Specify
XVO_003	201	Screening	10NOV2009	EENT	Abnormal	MAXILLARY DENTURES
				Head and Neck	Normal	
				Heart	Normal	
				Lungs	Normal	
				Abdomen	Normal	
				Genitourinary	Not Examined	
				Musculoskeletal	Normal	
				Neurological	Normal	
				Dermatological	Abnormal	-PURPURIC LESIONS OF BILATERAL ELBOWS - LEFT UPPER QUADRANT ABDOMEN - POST-SURGICAL SCAR - RIGHT POSTEROLATERAL THORAX - POST-SURGICAL SCAR
		Day 0		General Appearance	Normal	
				EENT	Abnormal	MAXILLARY DENTURES
				Head and Neck	Normal	
				Heart	Normal	
				Lungs	Normal	
				Abdomen	Normal	
				Genitourinary	Normal	
				Musculoskeletal	Normal	
				Neurological	Normal	
				Dermatological	Abnormal	-PURPURIC LESIONS -BILATERAL ELBOWS -POST SURGICAL CHANGES ON ABDOMEN AND THORAX
		Day 6		General Appearance	Normal	
				EENT	Abnormal	MAXILLARY DENTURES
				Head and Neck	Abnormal	MILD BILATERAL SALIVARY GLAND TENDERNESS
				Heart	Normal	
				Lungs	Normal	
				Abdomen	Normal	

Listing 16
Physical Examination

Center	Patient	Visit	Date Performed	Body System	Result	Specify
XVO_003	201	Day 6		Genitourinary	Normal	
				Musculoskeletal	Normal	
				Neurological	Normal	
				Dermatological	Abnormal	-PURPURIC LESIONS OF BILATERAL ELBOWS -LEFT ELBOW WITH HEALING SORE WITH OVERLYING BANDAGE-NO INDURATION/EDEMA/MILD ERYTHEMA -HEALED POST-SURGICAL SCARS ON ABDOMEN/THORAX
		Day 14		General Appearance	Normal	
				EENT	Abnormal	MAXILLARY DENTURES
				Head and Neck	Abnormal	MILD BILATERAL SUBMANDIBULAR GLAND TENDERNESS
				Heart	Normal	
				Lungs	Normal	
				Abdomen	Normal	
				Genitourinary	Normal	
				Musculoskeletal	Normal	
				Neurological	Normal	
				Dermatological	Abnormal	-LEFT ELBOW SORE HEALED -NEW RIGHT ELBOW SORE WITH OVERLYING BANDAGE -NO INDURATION/EDEMA BUT MILD ERYTHEMA -BILATERAL ELBOW PURPURIC LESIONS -HEALED POSTSURGICAL SCARS ON ABDOMEN AND THORAX
		Day 30		General Appearance	Normal	
				EENT	Abnormal	MAXILLARY DENTURES
				Head and Neck	Normal	
				Heart	Normal	
				Lungs	Normal	
				Abdomen	Normal	
				Genitourinary	Normal	
				Musculoskeletal	Normal	

Listing 16
Physical Examination

Center	Patient	Visit	Date Performed	Body System	Result	Specify
XVO_003	201	Day 30		Neurological	Normal	
				Dermatological	Abnormal	-LEFT AND RIGHT ELBOW SORES HEALED -SMALL BILATERAL ELBOW PURPURIC LESIONS -HEALED POST-SURGICAL SCARS ON ABDOMEN AND THORAX
		Day 42		General Appearance	Normal	
				EENT	Abnormal	MAXILLARY DENTURES
				Head and Neck	Normal	
				Heart	Normal	
				Lungs	Normal	
				Abdomen	Normal	
				Genitourinary	Normal	
				Musculoskeletal	Normal	
				Neurological	Normal	
				Dermatological	Abnormal	-SMALL BILATERAL ELBOW PURPURIC LESIONS,-HEALED POST-SURGICAL SCAR ON ABDOMEN AND THORAX
XVO_005	301	Screening	03AUG2009	General Appearance	Normal	
				EENT	Normal	
				Head and Neck	Normal	
				Heart	Normal	
				Lungs	Abnormal	DECREASED BREATH SOUNDS LEFT LUNG BASE RELATED TO PRIOR SURGERY
				Abdomen	Abnormal	PALPIABLE HERNIAS
				Genitourinary	Not Examined	
				Musculoskeletal	Normal	
				Neurological	Normal	
				Dermatological	Abnormal	LOVENOX INJECTION SITE BRUISING TO DERMATOLOGICAL,ABDOMINAL MIDLINE SCAR,UPPER LEFT AND RIGHT THORACIC SCARS
		Day 6		General Appearance	Normal	

Listing 16
Physical Examination

Center	Patient	Visit	Date Performed	Body System	Result	Specify
XVO_005	301	Day 6		EENT	Normal	
				Head and Neck	Normal	
				Heart	Normal	
				Lungs	Abnormal	DECREASED BREATH SOUNDS LEFT LUNG BASE RELATED TO PRIOR SURGERY
				Abdomen	Abnormal	PALPABLE HERNIAS
				Genitourinary	Not Examined	
				Musculoskeletal	Normal	
				Neurological	Normal	
				Dermatological	Abnormal	MIDLINE ABNORMAL SCAR,LOVENOX INJECTION SITE BRUISING,UPPER THORACIC LEFT AND RIGHT SCARS
		Day 14		General Appearance	Normal	
				EENT	Normal	
				Head and Neck	Normal	
				Heart	Normal	
				Lungs	Abnormal	DECREASED BREATH SOUNDS LEFT LUNG BASE RELATED TO PRIOR SURGERY
				Abdomen	Abnormal	PALPABLE HERNIAS
				Genitourinary	Not Examined	
				Musculoskeletal	Normal	
				Neurological	Normal	
				Dermatological	Abnormal	LOVENOX INJECTION SITE BRUSING MIDLINE ABDOMINAL SCAR,UPPER THORACIC LEFT AND RIGHT SCARS
		Day 30		General Appearance	Normal	
				EENT	Normal	
				Head and Neck	Normal	
				Heart	Normal	
				Lungs	Abnormal	DECREASED BREATH SOUNDS LEFT LUNG BASE
				Abdomen	Abnormal	PALPABLE HERNIAS

Listing 16
Physical Examination

Center	Patient	Visit	Date Performed	Body System	Result	Specify
XVO_005	301	Day 30		Genitourinary	Not Examined	
				Musculoskeletal	Normal	
				Neurological	Normal	
				Dermatological	Abnormal	LOVENOX INJECTION SITES(BRUISING) MIDLINE ABDOMINAL SCAR UPPER THORACIC LEFT AND RIGHT SCARS
		Day 42		General Appearance	Normal	
				EENT	Normal	
				Head and Neck	Normal	
				Heart	Normal	
				Lungs	Abnormal	DECREASED BREATH SOUNDS LEFT LUNG BASE MILD WHEEZING
				Abdomen	Abnormal	PALPABLE HERNIAS
				Genitourinary	Not Examined	
				Musculoskeletal	Normal	
				Neurological	Normal	
				Dermatological	Abnormal	LOVENOX SITES (BRUISING) MIDLINE ABDOMINAL SCAR, UPPER THORACIC LEFT AND RIGHT SCARS
	302	Screening	14DEC2009	General Appearance	Normal	
				EENT	Normal	
				Head and Neck	Normal	
				Heart	Normal	
				Lungs	Normal	
				Abdomen	Normal	
				Genitourinary	Not Examined	
				Musculoskeletal	Normal	
				Neurological	Normal	
				Dermatological	Normal	
		Day 14		General Appearance	Normal	
				EENT	Abnormal	SMALL STYE BELOW RIGHT EYE

Listing 16
Physical Examination

Center	Patient	Visit	Date Performed	Body System	Result	Specify
XVO_005	302	Day 14		Head and Neck	Normal	
				Heart	Normal	
				Lungs	Normal	
				Abdomen	Normal	
				Genitourinary	Not Examined	
				Musculoskeletal	Normal	
				Neurological	Normal	
				Dermatological	Normal	
		Day 30		General Appearance	Normal	
				EENT	Normal	
				Head and Neck	Normal	
				Heart	Normal	
				Lungs	Normal	
				Abdomen	Normal	
				Genitourinary	Not Examined	
				Musculoskeletal	Normal	
				Neurological	Normal	
				Dermatological	Normal	
		Day 42		General Appearance	Normal	
				EENT	Normal	
				Head and Neck	Normal	
				Heart	Normal	
				Lungs	Normal	
				Abdomen	Normal	
				Genitourinary	Not Examined	
				Musculoskeletal	Normal	
				Neurological	Normal	
				Dermatological	Normal	
XVO_007	401	Screening	10NOV2009	General Appearance	Normal	
				EENT	Normal	
				Head and Neck	Normal	

Listing 16
Physical Examination

Center	Patient	Visit	Date Performed	Body System	Result	Specify
XVO_007	401	Screening	10NOV2009	Heart	Normal	
				Lungs	Normal	
				Abdomen	Normal	
				Genitourinary	Not Examined	
				Musculoskeletal	Normal	
				Neurological	Not Examined	
				Dermatological	Normal	
		Day 0		General Appearance	Normal	
				EENT	Normal	
				Head and Neck	Normal	
				Heart	Normal	
				Lungs	Normal	
				Abdomen	Normal	
				Genitourinary	Not Examined	
				Musculoskeletal	Normal	
				Neurological	Not Examined	
				Dermatological	Normal	
		Day 6		General Appearance	Normal	
				EENT	Normal	
				Head and Neck	Normal	
				Heart	Normal	
				Lungs	Normal	
				Abdomen	Normal	
				Genitourinary	Not Examined	
				Musculoskeletal	Normal	
				Neurological	Not Examined	
				Dermatological	Normal	
		Day 14		General Appearance	Normal	
				EENT	Normal	
				Head and Neck	Normal	
				Heart	Normal	

Listing 16
Physical Examination

Center	Patient	Visit	Date Performed	Body System	Result	Specify
XVO_007	401	Day 14		Lungs	Normal	
				Abdomen	Normal	
				Genitourinary	Not Examined	
				Musculoskeletal	Normal	
				Neurological	Not Examined	
				Dermatological	Normal	
		Day 30		General Appearance	Normal	
				EENT	Normal	
				Head and Neck	Normal	
				Heart	Normal	
				Lungs	Normal	
				Abdomen	Normal	
				Genitourinary	Not Examined	
				Musculoskeletal	Normal	
				Neurological	Not Examined	
				Dermatological	Abnormal	ERYTHEMATOUS ON CHEST
		Day 42		General Appearance	Normal	
				EENT	Normal	
				Head and Neck	Normal	
				Heart	Normal	
				Lungs	Normal	
				Abdomen	Normal	
				Genitourinary	Not Examined	
				Musculoskeletal	Normal	
				Neurological	Not Examined	
				Dermatological	Normal	
	402	Screening	03DEC2009	General Appearance	Normal	
				EENT	Normal	
				Head and Neck	Normal	
				Heart	Normal	
				Lungs	Normal	

Listing 16
Physical Examination

Center	Patient	Visit	Date Performed	Body System	Result	Specify
XVO_007	402	Screening	03DEC2009	Abdomen	Normal	
				Genitourinary	Normal	
				Musculoskeletal	Normal	
				Neurological	Normal	
				Dermatological	Normal	
		Day 0		General Appearance	Normal	
				EENT	Normal	
				Head and Neck	Normal	
				Heart	Normal	
				Lungs	Normal	
				Abdomen	Normal	
				Genitourinary	Normal	
				Musculoskeletal	Normal	
				Neurological	Normal	
				Dermatological	Normal	
		Day 6		General Appearance	Normal	
				EENT	Normal	
				Head and Neck	Normal	
				Heart	Normal	
				Lungs	Normal	
				Abdomen	Normal	
				Genitourinary	Normal	
				Musculoskeletal	Normal	
				Neurological	Normal	
				Dermatological	Normal	
		Day 14		General Appearance	Normal	
				EENT	Normal	
				Head and Neck	Normal	
				Heart	Normal	
				Lungs	Normal	
				Abdomen	Normal	

Listing 16
Physical Examination

Center	Patient	Visit	Date Performed	Body System	Result	Specify
XVO_007	402	Day 14		Genitourinary	Normal	
				Musculoskeletal	Normal	
				Neurological	Normal	
				Dermatological	Normal	
		Day 30		General Appearance	Normal	
				EENT	Normal	
				Head and Neck	Normal	
				Heart	Normal	
				Lungs	Normal	
				Abdomen	Normal	
				Genitourinary	Normal	
				Musculoskeletal	Normal	
				Neurological	Normal	
				Dermatological	Normal	
		Day 42		General Appearance	Normal	
				EENT	Normal	
				Head and Neck	Normal	
				Heart	Normal	
				Lungs	Normal	
				Abdomen	Normal	
				Genitourinary	Normal	
				Musculoskeletal	Normal	
				Neurological	Normal	
				Dermatological	Normal	

Listing 17
Camera and Whole Body Imaging (Part I)

Center	Patient	Scan	Camera Manufacturer	Camera Model	Computer Vendor	Computer Software	Camera Type	Collimator	Same as Initial	Void Prior?	CO-57 Peak
XVO_001	101	Blank	SIEMENS	ECAM SIGNATURE	DELL	ESOFT (SYNGO)	DUAL DETECTOR	HI ENERGY			122±10%
		Attenuation	SIEMENS	ECAM SIGNATURE	DELL	ESOFT (SYNGO)	DUAL DETECTOR	HI ENERGY			122±10%
		15 Minutes	SIEMENS	ECAM SIGNATURE	DELL	ESOFT (SYNGO)	DUAL DETECTOR	HI ENERGY		NO	
		4-6 Hours	SIEMENS	ECAM SIGNATURE	DELL	ESOFT (SYNGO)	DUAL DETECTOR	HI ENERGY	YES		
		Day 1	SIEMENS	ECAM (SIGNATURE)	DELL	E SOFT (SYNGO)	DUAL DETECTOR	HI ENERGY	YES		
		Day 2	SIEMANS	E-CAM SIGNATURE	DELL	E-SOFT SYNGO	DUAL DETECTOR	HI ENERGY	YES		
		Day 3	SIEMANS	ECAM SIGNATURE	DELL	ESOFT SYNGO	DUAL DETECTOR	HI ENERGY	YES		
		Day 6	SIEMANS	ECAM SIGNATURE	DELL	ESOFT SYNGO	DUAL DETECTOR	HI ENERGY	YES		
		Day 14	SIEMENS	ECAM SIGNATURE	DELL	E-SOFT SYNGO	DUAL DETECTOR	HI ENERGY	YES		
	102	Blank	SIEMENS	ECAM SIGNATURE	DELL	SYNGO ESOFT	DUAL DETECTOR	HI ENERGY			122±10%
		Attenuation	SIEMENS	ECAM SIGNATURE	DELL	SYNGO ESOFT	DUAL DETECTOR	HI ENERGY			122±10%
		15 Minutes	SIEMENS	ECAM SIGNATURE	DELL	SYNGO ESOFT	DUAL DETECTOR	HI ENERGY		NO	
		4-6 Hours	SIEMANS	ECAM SIGNATURE	DELL	ESOFT (SYNGO)	DUAL DETECTOR	HI ENERGY	YES		
		Day 1	SIEMANS	ECAM SIGNATURE	DELL	ESOFT (SYNGO)	DUAL DETECTOR	HI ENERGY	YES		
		Day 2	SIEMENS	ECAM SIGNATURE	DELL	ESOFT (SYNGO)	DUAL DETECTOR	HI ENERGY	YES		
		Day 3	SIEMANS	ECAM SIGNATURE	DELL	ESOFT (SYNGO)	DUAL DETECTOR	HI ENERGY	YES		
		Day 6	SIEMENS	ECAM (SIGNATURE)	DELL	ESOFT (SYNGO)	DUAL DETECTOR	HI ENERGY	YES		
		Day 14	SIEMANS	ECAM SIGNATURE	DELL	ESOFT (SYNGO)	DUAL DETECTOR	HI ENERGY	YES		
	103	Blank	SIEMENS	ECAM SIGNATURE	DELL	SYNGO ESOFT	DUAL DETECTOR	HI ENERGY			122±10%
		Attenuation	SIEMENS	ECAM SIGNATURE	DELL	SYNGO ESOFT	DUAL DETECTOR	HI ENERGY			122±10%
		15 Minutes	SIEMENS	ECAM SIGNATURE	DELL	SYNGO ESOFT	DUAL DETECTOR	HI ENERGY		NO	
		4-6 Hours	SIEMENS	ECAM SIGNATURE	DELL	SYNGO ESOFT	DUAL DETECTOR	HI ENERGY	YES		
		Day 1	SIEMENS	ECAM SIGNATURE	DELL	SYNGO ESOFT	DUAL DETECTOR	HI ENERGY	YES		
		Day 2	SIEMENS	ECAM SIGNATURE	DELL	SYNGO ESOFT	DUAL DETECTOR	HI ENERGY	YES		
		Day 3	SIEMENS	ECAM SIGNATURE	DELL	SYNGO ESOFT	DUAL DETECTOR	HI ENERGY	YES		
		Day 6	SIEMENS	ECAM SIGNATURE	DELL	SYNGO ESOFT	DUAL DETECTOR	HI ENERGY	YES		
		Day 14	SIEMENS	ECAM SIGNATURE	DELL	ESOFT SYNGO	DUAL DETECTOR	HI ENERGY	YES		

Listing 17
Camera and Whole Body Imaging (Part I)

			Camera		Computer						
Center	Patient	Scan	Manufacturer	Model	Vendor	Software	Camera Type	Collimator	Same as Initial	Void Prior?	CO-57 Peak
XVO_003	201	Blank	GE	MILLENNIUM VG	GE	XELERIS	DUAL DETECTOR	HI ENERGY			122±10%
		Attenuation	GE	MILLENNIUM VG	GE	XELERIS	DUAL DETECTOR	HI ENERGY			122±10%
		15 Minutes	GE	MILLENNIUM VG	GE	XELERIS	DUAL DETECTOR	HI ENERGY		NO	
		4-6 Hours	GE	MILLENNIUM VG	GE	XELERIS	DUAL DETECTOR	HI ENERGY	YES		
		Day 1	GE	MILLENNIUM VG	GE	XELERIS	DUAL DETECTOR	HI ENERGY	YES		
		Day 2	GE	MILLENNIUM VG	GE	XELERIS	DUAL DETECTOR	HI ENERGY	YES		
		Day 3	GE	MILLENNIUM VG	GE	XELERIS	DUAL DETECTOR	HI ENERGY	YES		
		Day 6	GE	MILLENIUM VG	GE	XELERIS	DUAL DETECTOR	HI ENERGY	YES		
		Day 14	GE	MILLENIUM VG	GE	XELERIS	DUAL DETECTOR	HI ENERGY	YES		
XVO_005	301	Blank	GE	INFINIA	GE	XELERIS	DUAL DETECTOR	HI ENERGY			122±10%
		Attenuation	GE	INFINIA	GE	XELERIS	DUAL DETECTOR	HI ENERGY			122±10%
		15 Minutes	GE	INFINIA	GE	XELERIS	DUAL DETECTOR	HI ENERGY		NO	
		4-6 Hours	GE	INFINIA	GE	XELERIS	DUAL DETECTOR	HI ENERGY	YES		
		Day 1	GE	INFINIA	GE	XELERIS	DUAL DETECTOR	HI ENERGY	YES		
		Day 2	GE	INFINIA	GE	XELERIS	DUAL DETECTOR	HI ENERGY	YES		
		Day 3	GE	INFINIA	GE	XELERIS	DUAL DETECTOR	HI ENERGY	YES		
		Day 6	GE	INFINIA	GE	XELERIS	DUAL DETECTOR	HI ENERGY	YES		
		Day 14	GE	INFINIA	GE	XELERIS	DUAL DETECTOR	HI ENERGY	YES		
	302	Blank	GE	INFINIA	GE	XELERIS	DUAL DETECTOR	HI ENERGY			122±10%
		Attenuation	GE	INFINIA	GE	XELERIS	DUAL DETECTOR	HI ENERGY			122±10%
		15 Minutes	GE	INFINIA	GE	XELERIS	DUAL DETECTOR	HI ENERGY		NO	
		4-6 Hours	GE	INFINIA	GE	XELERIS	DUAL DETECTOR	HI ENERGY	YES		
		Day 1	GE	INFINIA	GE	XELERIS	DUAL DETECTOR	HI ENERGY	YES		
		Day 2	GE	INFINIA	GE	XELERIS	DUAL DETECTOR	HI ENERGY	YES		
		Day 3	GE	INFINIA	GE	XELERIS	DUAL DETECTOR	HI ENERGY	YES		
		Day 6	GE	INFINIA	GE	XELERIS	DUAL DETECTOR	HI ENERGY	YES		
		Day 14	GE	INFINIA	GE	XELERIS	DUAL DETECTOR	HI ENERGY	YES		

Listing 17
Camera and Whole Body Imaging (Part I)

Center	Patient	Scan	Camera Manufacturer	Camera Model	Computer Vendor	Computer Software	Camera Type	Collimator	Same as Initial	Void Prior?	CO-57 Peak
XVO_007	401	Blank	GE	INFINIA/HAWKEYE	GE	XELERIS 1.1	DUAL DETECTOR	HI ENERGY			122±10%
		Attenuation	GE	INFINIA/HAWKEYE	GE	XELERIS 1.1	DUAL DETECTOR	HI ENERGY			122±10%
		15 Minutes	GE	INFINIA/HAWKEYE	GE	XELERIS 1.1	DUAL DETECTOR	HI ENERGY		YES: PATIENT'S BLADDER FULL COULDNOT HOLD IT	
		4-6 Hours	GE	INFINIA/HAWKEYE	GE	XELERIS 1.1	DUAL DETECTOR	HI ENERGY	YES		
		Day 1	GE	INFINIA/HAWKEYE	GE	XELERIS 1.1	DUAL DETECTOR	HI ENERGY	YES		
		Day 2	GE	INFINIA/HAWKEYE	GE	XELERIS 1.1	DUAL DETECTOR	HI ENERGY	YES		
		Day 3	GE	INFINIA/HAWKEYE	GE	XELERIS 1.1	DUAL DETECTOR	HI ENERGY	YES		
		Day 6	GE	INFINIA/HAWKEYE	GE	XELERIS 1.1	DUAL DETECTOR	HI ENERGY	YES		
		Day 14	GE	INFINIA/HAWKEYE	GE	XELERIS 1.1	DUAL DETECTOR	HI ENERGY	YES		
	402	Blank	GE	INFINIA/HAWKEYE	GE	XELERIS 1.1.	DUAL DETECTOR	HI ENERGY			122±10%
		Attenuation	GE	INFINIA/HAWKEYE	GE	XELERIS 1.1.	DUAL DETECTOR	HI ENERGY			122±10%
		15 Minutes	GE	INFINIA/HAWKEYE	GE	XELERIS 1.1.	DUAL DETECTOR	HI ENERGY		YES: CONDOM CATHETER PATIENT IS INCONTINENT	
		4-6 Hours	GE	INFINIA/HAWKEYE	GE	XELERIS 1.1	DUAL DETECTOR	HI ENERGY	YES		
		Day 1	GE	INFINIA/HAWKEYE	GE	XELERIS 1.1	DUAL DETECTOR	HI ENERGY	YES		
		Day 2	GE	INFINIA/HAWKEYE	GE	XELERIS 1.1	DUAL DETECTOR	HI ENERGY	YES		
		Day 3	GE	INFINIA/HAWKEYE	GE	XELERIS 1.1	DUAL DETECTOR	HI ENERGY	YES		
		Day 6	GE	INFINIA/HAWKEYE	GE	XELERIS 1.1	DUAL DETECTOR	HI ENERGY	YES		
		Day 14	GE	INFINIA/HAWKEYE	GE	XELERIS 1.1	DUAL DETECTOR	HI ENERGY	YES		

Listing 17
Camera and Whole Body Imaging (Part II)

Center	Patient	Scan	Acquisition Times Start/End	Acquisition Times Start/End [a]	Scan Speed (cm/min)	Matrix	I-131 Peaks	Reference Standard Activity	Date/Time Assayed	Urine Collection Completed As Per Protocol
XVO_001	101	Blank	11:14/ 11:26		20	256 X 1024				
		Attenuation	13:10/ 13:22		20	256 X 1024				
		15 Minutes	15:46/ 16:11		10	256 X 1024	298± 7.5%; 364± 10%; 436± 7.5%	51 UCI	10NOV2009 15:44	
		4-6 Hours	19:54/ 20:17		10	256 X 1024	298± 7.5%; 364± 10%; 436± 7.5%	48 UCI	10NOV2009 19:52	Yes
		Day 1	14:19/ 14:43	14:54/ 15:18	10	256 X 1024	298± 7.5%; 364± 10%; 436± 7.5%	47 UCI	11NOV2009 14:19	Yes
		Day 2	11:57/ 12:23		10	256 X 1024	298± 7.5%; 364± 10%; 436± 7.5%	43 UCI	12NOV2009 11:54	Yes
		Day 3	12:00/ 12:24		10	256 X 1024	298± 7.5%; 364± 10%; 436± 7.5%	38 UCI	13NOV2009 12:43	Yes
		Day 6	13:51/ 14:14		10	256 X 1024	298± 7.5%; 364± 10%; 436± 7.5%	27 UCI	16NOV2009 13:48	Yes
		Day 14	10:12/ 10:36		10	256 X 1024	298± 7.5%; 364± 10%; 436± 7.5%	20 UCI	24NOV2009 10:09	Yes
	102	Blank	09:31/ 09:43		20	256 X 1024				
		Attenuation	11:46/ 11:57		20	256 X 1024				
		15 Minutes	13:35/ 13:59		10	256 X 1024	298± 7.5%; 364± 10%; 436± 7.5%	150 UCI	15DEC2009 13:33	

Note: [a] second acquisition, if single detector
ND =Not Done

Listing 17
Camera and Whole Body Imaging (Part II)

Center	Patient	Scan	Acquisition Times Start/End	Start/End [a]	Scan Speed (cm/min)	Matrix	I-131 Peaks	Reference Standard Activity	Date/Time Assayed	Urine Collection Completed As Per Protocol
XVO_001	102	4-6 Hours	17:15/ 17:40		10	256 X 1024	298± 7.5%; 364± 10%; 436± 7.5%	149 UCI	15DEC2009 17:01	Yes
		Day 1	12:37/ 13:01		10	256 X 1024	298± 7.5%; 364± 10%; 436± 7.5%	138 UCI	16DEC2009 12:35	Yes
		Day 2	16:45/ 17:09		10	256 X 1024	298± 7.5%; 364± 10%; 436± 7.5%	125 UCI	17DEC2009 16:38	Yes
		Day 3	10:52/ 11:16		10	256 X 1024	298± 7.5%; 364± 10%; 436± 7.5%	120 UCI	18DEC2009 10:50	Yes
		Day 6	10:49/ 11:13		10	256 X 1024	298± 7.5%; 364± 10%; 436± 7.5%	ND		Yes
		Day 14	11:20/ 11:44		10	256 X 1024	298± 7.5%; 364± 10%; 436± 7.5%	50 UCI	29DEC2009 11:15	Yes
	103	Blank	08:13/ 08:25		20	256 X 1024				
		Attenuation	12:03/ 12:15		20	256 X 1024				
		15 Minutes	13:21/ 13:46		10	256 X 1024	298± 7.5%; 364± 10%; 436± 7.5%	123 UCI	12JAN2010 13:18	
		4-6 Hours	17:19/ 17:43		10	256 X 1024	298± 7.5%; 364± 10%; 436± 7.5%	123 UCI	12JAN2010 17:16	Yes
		Day 1	09:32/ 09:56		10	256 X 1024	298± 7.5%; 364± 10%; 436± 7.5%	115 UCI	13JAN2010 09:28	Yes
		Day 2	09:13/ 09:37		10	256 X 1024	298± 7.5%; 364± 10%; 436± 7.5%	104 UCI	14JAN2010 09:06	Yes
		Day 3	13:28/ 13:52		10	256 X 1024	298± 7.5%; 364± 10%; 436± 7.5%	96 UCI	15JAN2010 13:24	Yes

Note: [a] second acquisition, if single detector
ND =Not Done

Listing 17
Camera and Whole Body Imaging (Part II)

Center	Patient	Scan	Acquisition Times Start/End	Acquisition Times Start/End [a]	Scan Speed (cm/min)	Matrix	I-131 Peaks	Reference Standard Activity	Date/Time Assayed	Urine Collection Completed As Per Protocol
XVO_001	103	Day 6	12:18/ 12:46		10	256 X 1024	298± 7.5%; 364± 10%; 436± 7.5%	68 UCI	19JAN2010 12:17	Yes
		Day 14	10:18/ 10:42		10	256 X 1024	298± 7.5%; 364± 10%; 436± 7.5%	38 UCI	26JAN2010 10:10	Yes
XVO_003	201	Blank	10:41/ 10:53		20	256 X 1024				
		Attenuation	10:19/ 10:31		20	256 X 1024				
		15 Minutes	14:10/ 14:34		10	256 X 1024	298± 7.5%; 364± 10%; 436± 7.5%	0.156 MCI	17NOV2009 12:50	
		4-6 Hours	17:36/ 18:00		10	256 X 1024	298± 7.5%; 364± 10%; 436± 7.5%	0.156 MCI	17NOV2009 12:50	Yes
		Day 1	10:43/ 11:07		10	256 X 1024	298± 7.5%; 364± 10%; 436± 7.5%	0.156 MCI	17NOV2009 12:50	Yes
		Day 2	09:22/ 09:45		10	256 X 1024	298± 7.5%; 364± 10%; 436± 7.5%	0.156 MCI	17NOV2009 12:50	Yes
		Day 3	09:50/ 10:14		10	256 X 1024	298± 7.5%; 364± 10%; 436± 7.5%	0.156 MCI	17NOV2009 12:50	Yes
		Day 6	09:49/ 10:12		10	256 X 1024	298± 7.5%; 364± 10%; 436± 7.5%	0.156 MCI	17NOV2009 12:50	Yes
		Day 14	09:50/ 10:13		10	256 X 1024	298± 7.5%; 364± 10%; 436± 7.5%	0.156 MCI	17NOV2009 12:50	Yes
XVO_005	301	Blank	16:14/ 16:26		20	256 X 1024				
		Attenuation	11:37/ 11:49		20	256 X 1024				

Note: [a] second acquisition, if single detector
ND =Not Done

Listing 17
Camera and Whole Body Imaging (Part II)

			Acquisition Times							
Center	Patient	Scan	Start/End	Start/End [a]	Scan Speed (cm/min)	Matrix	I-131 Peaks	Reference Standard Activity	Date/Time Assayed	Urine Collection Completed As Per Protocol
XVO_005	301	15 Minutes	12:45/ 13:07		10	256 X 1024	OTHER: 298 ± 7.5%; 364 ± 7.5% 436 ± 10%	145.7 UCI	04AUG2009 12:43	
		4-6 Hours	17:05/ 17:26		10	256 X 1024	OTHER: 298±7.5%; 364±7.5% 436±10%	143.4 UCI	04AUG2009 17:00	Yes
		Day 1	11:57/ 12:18		20	256 X 1024	OTHER: 298+-7.5%; 364+-7.5% 436+-10%	134 UCI	05AUG2009 11:45	Yes
		Day 2	09:51/ 10:12		20	256 X 1024	OTHER: 298+-7.5%; 364+-7.5% 436+-10%	123.8 UCI	06AUG2009 09:45	Yes
		Day 3	09:55/ 10:18		10	256 X 1024	OTHER: 298+-7.5% 364+-7.5% 436+-10%	113.2 UCI	07AUG2009 09:45	Yes
		Day 6	11:40/ 12:03		10	256 X 1024	OTHER: 298+-7.5% 364+-7.5% 436+-10%	87.25 UCI	10AUG2009 11:30	Yes
		Day 14	11:26/ 11:47		10	256 X 1024	OTHER: 298+-7.5% 364+-7.5% 436+-10%	43.8 UCI	18AUG2009 11:05	No: SUBJECT MISSED ONE URINE COLLECTION ON 11/AUG/2009, DAY 7 OF PROTOCOL. URINE COLLECTION MISSED WAS EARLY MORNING OF 11/AUG/2009. URINE WOULD HAVE BEEN COLLECTED IN DAY 6-7 JUG.
	302	Blank	11:11/ 11:24		20	256 X 1024				
		Attenuation	11:44/ 11:57		20	256 X 1024				
		15 Minutes	12:37/ 12:58		10	256 X 1024	298± 7.5%; 364± 10%; 436± 7.5%	136.4 UCI	15DEC2009 11:36	

Note: [a] second acquisition, if single detector
ND =Not Done

Listing 17
Camera and Whole Body Imaging (Part II)

Center	Patient	Scan	Acquisition Times Start/End	Acquisition Times Start/End [a]	Scan Speed (cm/min)	Matrix	I-131 Peaks	Reference Standard Activity	Date/Time Assayed	Urine Collection Completed As Per Protocol
XVO_005	302	4-6 Hours	16:33/ 16:57		10	256 X 1024	298± 7.5%; 364± 10%; 436± 7.5%	147.8 UCI	15DEC2009 16:33	Yes
		Day 1	10:45/ 11:09		10	256 X 1024	298± 7.5%; 364± 10%; 436± 7.5%	116.4 UCI	16DEC2009 10:35	Yes
		Day 2	10:43/ 11:05		10	256 X 1024	298± 7.5%; 364± 10%; 436± 7.5%	112.5 UCI	17DEC2009 10:36	Yes
		Day 3	09:50/ 10:12		10	256 X 1024	298± 7.5%; 364± 10%; 436± 7.5%	104.2 UCI	18DEC2009 10:16	Yes
		Day 6	10:08/ 10:31		10	256 X 1024	298± 7.5%; 364± 10%; 436± 7.5%	81.0 UCI	21DEC2009 09:45	Yes
		Day 14	10:23/ 10:44		10	256 X 1024	298± 7.5%; 364± 10%; 436± 7.5%	38.1 UCI	29DEC2009 09:35	Yes
XVO_007	401	Blank	08:35/ 08:45		20	256 X 1024				
		Attenuation	11:59/ 12:10		20	256 X 1024				
		15 Minutes	13:10/ 13:31		10	256 X 1024	298± 7.5%; 364± 10%; 436± 7.5%	143 UCI	10NOV2009 11:28	
		4-6 Hours	17:00/ 17:22		10	256 X 1024	298± 7.5%; 364± 10%; 436± 7.5%	143 UCI	10NOV2009 11:28	Yes
		Day 1	08:10/ 08:33		10	256 X 1024	298± 7.5%; 364± 10%; 436± 7.5%	143 UCI	10NOV2009 11:28	Yes
		Day 2	10:05/ 10:27		10	256 X 1024	298± 7.5%; 364± 10%; 436± 7.5%	143 UCI	10NOV2009 11:28	Yes
		Day 3	08:44/ 09:06		10	256 X 1024	298± 7.5%; 364± 10%; 436± 7.5%	143 UCI	10NOV2009 11:20	Yes

Note: [a] second acquisition, if single detector
ND =Not Done

Listing 17
Camera and Whole Body Imaging (Part II)

Center	Patient	Scan	Acquisition Times Start/End	Acquisition Times Start/End [a]	Scan Speed (cm/min)	Matrix	I-131 Peaks	Reference Standard Activity	Date/Time Assayed	Urine Collection Completed As Per Protocol
XVO_007	401	Day 6	09:24/ 09:46		10	256 X 1024	298± 7.5%; 364± 10%; 436± 7.5%	143 UCI	10NOV2009 11:28	Yes
		Day 14	08:23/ 08:47		10	256 X 1024	298± 7.5%; 364± 10%; 436± 7.5%	143 UCI	10NOV2009 11:28	Yes
	402	Blank	11:00/ 11:11		20	256 X 1024				
		Attenuation	11:42/ 11:53		20	256 X 1024				
		15 Minutes	12:49/ 13:13		10	256 X 1024	298± 7.5%; 364± 10%; 436± 7.5%	136 UCI	08DEC2009 11:22	
		4-6 Hours	16:56/ 17:19		10	256 X 1024	298± 7.5%; 364± 10%; 436± 7.5%	136 UCI	08DEC2009 11:22	Yes
		Day 1	08:28/ 08:52		10	256 X 1024	298± 7.5%; 364± 10%; 436± 7.5%	136 UCI	08DEC2009 11:22	Yes
		Day 2	08:25/ 08:49		10	256 X 1024	298± 7.5%; 364± 10%; 436± 7.5%	136 UCI	08DEC2009 11:22	Yes
		Day 3	08:17/ 08:40		10	256 X 1024	298± 7.5%; 364± 10%; 436± 7.5%	136 UCI	08DEC2009 11:22	Yes
		Day 6	12:54/ 13:18		10	256 X 1024	298± 7.5%; 364± 10%; 436± 7.5%	82.8 UCI	14DEC2009 13:23	Yes
		Day 14	08:28/ 08:51		10	256 X 1024	298± 7.5%; 364± 10%; 436± 7.5%	136 UCI	08DEC2009 11:22	Yes

Note: [a] second acquisition, if single detector
ND =Not Done

Listing 18.1
Blood Sampling Times - Biodistribution

Center	Patient	Time Point	Date	Actual Time	Actual Time Post Dose
XVO_001	101	Day 0 Pre-dose	10NOV2009	14:11	-01:03
		Infusion Stop Time		15:24	00:10
		15 Minutes		15:39	00:25
		60 Minutes		16:36	01:22
		4-6 Hours		19:41	04:27
		18-24 Hours	11NOV2009	15:36	24:22
		Day 2	12NOV2009	11:36	44:22
		Day 3	13NOV2009	11:54	68:40
		Day 6	16NOV2009	13:45	142:31
		Day 14	24NOV2009	10:50	331:36
		Day 30	11DEC2009	12:00	740:46
		Day 42	05JAN2010	14:30	1343:16
	102	Day 0 Pre-dose	15DEC2009	12:32	-00:13
		Infusion Stop Time		12:55	00:10
		15 Minutes		13:13	00:28
		60 Minutes		14:07	01:22
		4-6 Hours		17:40	04:55
		18-24 Hours	16DEC2009	13:20	24:35
		Day 2	17DEC2009	16:22	51:37
		Day 3	18DEC2009	18:17	77:32
		Day 6	21DEC2009	08:40	139:55
		Day 14	29DEC2009	08:41	331:56
		Day 30	15JAN2010	08:20	739:35
		Day 42	28JAN2010	09:50	1053:05

Listing 18.1
Blood Sampling Times - Biodistribution

Center	Patient	Time Point	Date	Actual Time	Actual Time Post Dose
XVO_001	103	Day 0 Pre-dose	12JAN2010	12:33	-00:18
		Infusion Stop Time		13:01	00:10
		15 Minutes		13:16	00:25
		60 Minutes		14:09	01:18
		4-6 Hours		17:06	04:15
		18-24 Hours	13JAN2010	09:10	20:19
		Day 2	14JAN2010	09:52	45:01
		Day 3	15JAN2010	15:03	74:12
		Day 6	19JAN2010	12:12	167:21
		Day 14	26JAN2010	10:15	333:24
		Day 30	09FEB2010	12:05	671:14
		Day 42	19FEB2010	08:15	907:24
XVO_003	201	Day 0 Pre-dose	17NOV2009	08:50	-04:25
		Infusion Stop Time		13:25	00:10
		15 Minutes		13:41	00:26
		60 Minutes		14:25	01:10
		4-6 Hours		17:15	04:00
		18-24 Hours	18NOV2009	09:13	19:58
		Day 2	19NOV2009	08:45	43:30
		Day 3	20NOV2009	08:47	67:32
		Day 6	23NOV2009	09:05	139:50
		Day 14	02DEC2009	09:00	355:45
		Day 30	22DEC2009	09:02	835:47
		Day 42	29DEC2009	08:54	1003:39

Listing 18.1
Blood Sampling Times - Biodistribution

Center	Patient	Time Point	Date	Actual Time	Actual Time Post Dose
XVO_005	301	Day 0 Pre-dose	04AUG2009	11:57	-00:12
		Infusion Stop Time		12:20	00:11
		15 Minutes		12:40	00:31
		60 Minutes		13:25	01:16
		4-6 Hours		16:38	04:29
		18-24 Hours	05AUG2009	11:20	23:11
		Day 2	06AUG2009	09:45	45:36
		Day 3	07AUG2009	09:44	69:35
		Day 6	10AUG2009	10:45	142:36
		Day 14	18AUG2009	10:28	334:19
		Day 30	04SEP2009	10:03	741:54
		Day 42	15SEP2009	10:20	1006:11
	302	Day 0 Pre-dose	15DEC2009	09:07	-02:56
		Infusion Stop Time		12:13	00:10
		15 Minutes		12:28	00:25
		60 Minutes		13:13	01:10
		4-6 Hours		16:20	04:17
		18-24 Hours	16DEC2009	10:30	22:27
		Day 2	17DEC2009	10:35	46:32
		Day 3	18DEC2009	08:30	68:27
		Day 6	21DEC2009	08:40	140:37
		Day 14	29DEC2009	08:40	332:37
		Day 30	15JAN2010	10:40	742:37
		Day 42	25JAN2010	11:18	983:15

Listing 18.1
Blood Sampling Times - Biodistribution

Center	Patient	Time Point	Date	Actual Time	Actual Time Post Dose
XVO_007	401	Day 0 Pre-dose	10NOV2009	10:38	-01:47
		Infusion Stop Time		12:36	00:11
		15 Minutes		12:51	00:26
		60 Minutes		13:36	01:11
		4-6 Hours		16:40	04:15
		18-24 Hours	11NOV2009	08:50	20:25
		Day 2	12NOV2009	11:10	46:45
		Day 3	13NOV2009	08:15	67:50
		Day 6	16NOV2009	08:45	140:20
		Day 14	24NOV2009	09:45	333:20
		Day 30	08DEC2009	12:15	671:50
		Day 42	22DEC2009	14:30	1010:05
	402	Day 0 Pre-dose	08DEC2009	11:30	-00:54
		Infusion Stop Time		12:34	00:10
		15 Minutes		13:05	00:41
		60 Minutes		13:37	01:13
		4-6 Hours		16:45	04:21
		18-24 Hours	09DEC2009	08:00	19:36
		Day 2	10DEC2009	08:20	43:56
		Day 3	11DEC2009	08:00	67:36
		Day 6	14DEC2009	11:45	143:21
		Day 14	22DEC2009	09:50	333:26
		Day 30	05JAN2010	10:00	669:36
		Day 42	19JAN2010	11:50	1007:26

Listing 18.2
Blood Sampling Times - Pharmacokinetics

Center	Patient	Time Point	Date	Actual Time	Actual Time Post Dose
XVO_001	101	Day 0 Pre-dose	10NOV2009	14:11	-01:03
		Infusion Stop Time		15:24	00:10
		5 Minutes		15:19	00:05
		15 Minutes		15:39	00:25
		30 Minutes		16:09	00:55
		60 Minutes		16:36	01:22
		4-6 Hours		19:41	04:27
		18-24 Hours	11NOV2009	15:36	24:22
		Day 2	12NOV2009	11:35	44:21
		Day 3	13NOV2009	11:54	68:40
		Day 6	16NOV2009	13:45	142:31
		Day 14	24NOV2009	10:50	331:36
		Day 30	11DEC2009	12:00	740:46
		Day 42	05JAN2010	14:30	1343:16
	102	Day 0 Pre-dose	15DEC2009	12:31	-00:14
		Infusion Stop Time		12:55	00:10
		5 Minutes		13:08	00:23
		15 Minutes		13:12	00:27
		30 Minutes		13:25	00:40
		60 Minutes		14:07	01:22
		4-6 Hours		17:40	04:55
		18-24 Hours	16DEC2009	13:25	24:40
		Day 2	17DEC2009	16:21	51:36
		Day 3	18DEC2009	18:17	77:32
		Day 6	21DEC2009	08:40	139:55
		Day 14	29DEC2009	08:41	331:56
		Day 30	15JAN2010	08:20	739:35
		Day 42	28JAN2010	09:50	1053:05

Listing 18.2
Blood Sampling Times - Pharmacokinetics

Center	Patient	Time Point	Date	Actual Time	Actual Time Post Dose
XVO_001	103	Day 0 Pre-dose	12JAN2010	12:33	-00:18
		Infusion Stop Time		13:01	00:10
		5 Minutes		13:06	00:15
		15 Minutes		13:16	00:25
		30 Minutes		13:33	00:42
		60 Minutes		14:09	01:18
		4-6 Hours		17:06	04:15
		18-24 Hours	13JAN2010	09:11	20:20
		Day 2	14JAN2010	09:52	45:01
		Day 3	15JAN2010	15:03	74:12
		Day 6	19JAN2010	12:12	167:21
		Day 14	26JAN2010	10:15	333:24
		Day 30	09FEB2010	12:05	671:14
		Day 42	19FEB2010	08:15	907:24
XVO_003	201	Day 0 Pre-dose	17NOV2009	08:50	-04:25
		Infusion Stop Time		13:25	00:10
		5 Minutes		13:30	00:15
		15 Minutes		13:41	00:26
		30 Minutes		13:55	00:40
		60 Minutes		14:25	01:10
		4-6 Hours		17:15	04:00
		18-24 Hours	18NOV2009	09:13	19:58
		Day 2	19NOV2009	08:45	43:30
		Day 3	20NOV2009	08:47	67:32
		Day 6	23NOV2009	09:05	139:50
		Day 14	02DEC2009	09:00	355:45
		Day 30	22DEC2009	09:02	835:47
		Day 42	29DEC2009	08:54	1003:39

Listing 18.2
Blood Sampling Times - Pharmacokinetics

Center	Patient	Time Point	Date	Actual Time	Actual Time Post Dose
XVO_005	301	Day 0 Pre-dose	04AUG2009	11:57	-00:12
		Infusion Stop Time		12:20	00:11
		5 Minutes		12:30	00:21
		15 Minutes		12:40	00:31
		30 Minutes			
		60 Minutes		13:25	01:16
		4-6 Hours		16:38	04:29
		18-24 Hours	05AUG2009	11:20	23:11
		Day 2	06AUG2009	09:45	45:36
		Day 3	07AUG2009	09:44	69:35
		Day 6	10AUG2009	10:45	142:36
		Day 14	18AUG2009	10:28	334:19
		Day 30	04SEP2009	10:05	741:56
		Day 42	15SEP2009	10:20	1006:11
	302	Day 0 Pre-dose	15DEC2009	09:07	-02:56
		Infusion Stop Time		12:13	00:10
		5 Minutes		12:18	00:15
		15 Minutes		12:28	00:25
		30 Minutes			
		60 Minutes		13:13	01:10
		4-6 Hours		16:20	04:17
		18-24 Hours	16DEC2009	10:30	22:27
		Day 2	17DEC2009	10:35	46:32
		Day 3	18DEC2009	08:30	68:27
		Day 6	21DEC2009	08:40	140:37
		Day 14	29DEC2009	08:40	332:37
		Day 30	15JAN2010	10:40	742:37
		Day 42	25JAN2010	11:18	983:15

Listing 18.2
Blood Sampling Times - Pharmacokinetics

Center	Patient	Time Point	Date	Actual Time	Actual Time Post Dose
XVO_007	401	Day 0 Pre-dose	10NOV2009	10:38	-01:47
		Infusion Stop Time		12:36	00:11
		5 Minutes		12:41	00:16
		15 Minutes		12:51	00:26
		30 Minutes		13:06	00:41
		60 Minutes		13:36	01:11
		4-6 Hours		16:40	04:15
		18-24 Hours	11NOV2009	08:50	20:25
		Day 2	12NOV2009	11:10	46:45
		Day 3	13NOV2009	08:15	67:50
		Day 6	16NOV2009	08:45	140:20
		Day 14	24NOV2009	09:45	333:20
		Day 30	08DEC2009	12:15	671:50
		Day 42	22DEC2009	14:30	1010:05
	402	Day 0 Pre-dose	08DEC2009	11:30	-00:54
		Infusion Stop Time		12:34	00:10
		5 Minutes		12:39	00:15
		15 Minutes		13:05	00:41
		30 Minutes		13:12	00:48
		60 Minutes		13:37	01:13
		4-6 Hours		16:45	04:21
		18-24 Hours	09DEC2009	08:00	19:36
		Day 2	10DEC2009	08:20	43:56
		Day 3	11DEC2009	08:00	67:36
		Day 6	14DEC2009	11:45	143:21
		Day 14	22DEC2009	09:50	333:26
		Day 30	05JAN2010	10:00	669:36
		Day 42	19JAN2010	11:50	1007:26

Listing 19
Study Termination

Center	Patient	Date of Visit	Reason for Ending Study Participation
XVO_001	101	05JAN2010	Study Completed
	102	29JAN2010	Study Completed
	103	19FEB2010	Study Completed
XVO_003	201	29DEC2009	Study Completed
XVO_005	301	15SEP2009	Study Completed
	302	25JAN2010	Study Completed
XVO_007	401	22DEC2009	Study Completed
	402	19JAN2010	Study Completed

Listing 20
Study Comments

Center	Patient	Date	CRF Page	Comment
XVO_001	101	12MAR2009	018	TEMP AT 46HR INTERVAL NOT RECORDED DUE TO SITE OVERSIGHT. VITALS NOT TAKEN AT 30MIN INTERVAL DUE TO PATIENT BEING UNDER SCANNER.
			025	13NOV2009 VITAL SIGNS: CRF IS SOURCE DOUCUMENT. SAO2% NOTDONE DUE TO SITE OVERSIGHT
			028	VITALS COMPLETED IN UPRIGHT POSITION
			032	VITALS COMPLETED IN UPRIGHT POSITION.
			OPK	BLOOD DRAWN 5MIN AFTER INFUSION INSTEAD OF 5MIN AFTER INFUSION ENDED
		13JAN2010	39	DAY 56 05-JAN2010 BLOOD PRESSURE WAS OBTAINED UPRIGHT INSTEAD OF SUPINE
		10FEB2010	37	PHYSICAL EXAMINATION PAGE FOR DAY 31 SHOULD HAVE BEEN NUMBERED PAGE 37AND WAS MISTAKENLY NUMBERED 33. DAY 31VISIT IS NOT OUTSIDE OF PROTOCOL.
			42	PAGES 39,40,41,42;ALL PATIENT ASSESSMENT FOR DAY 42 PER PROTOCOL WERE PERFORMED ON DAY 56 DUE TO PATIENT BEING ON VACATION AND UNAVAILABLE FOR FOLLOW-UP
		22FEB2010	PG.039	VITALS UPRIGHT AT DAY 30 VISIT
			PG.041	GGT NOT DONE : URINALYSIS NOT DONE DUE TO OVERSIGHT
	102	22FEB2010	PG 06	SCREENING VITALS PERFORMED IN UPRIGHT POSITION
			PG 10	PHASE I TRAIL OF ERBITUX & IRINOTECAN & RAD001 JUN2008-JUL2009
	103	03MAR2010	PG28	DAY 6 ASSESSMENTS PERFORMED ON DAY 7 DS 03/MAR/2010.
XVO_003	201	20JAN2010	002	INR DRAWN 22DEC2009 AND WAS WNL
			010	PATIENT WAS ON OCTREOTIDE THERAPY FROM 28MAY2009 TO 30JUL2009. THIS WAS FOR SYMPTOM RELIEF OF CHRONIC DIARRHEA AND NOT ANTI-TUMOR THERAPY.
			018	PATIENT HAS HISTORY OF LABILE BP NOTED THROUGHOUT THE STUDY; HOWEVER THE SIGNIFICANT CHANGE IN SBP AND DBP BETWEEN 60MIN AND 4-6 HRS IS RECORDED AS AN AE.
			019	15MIN IMAGE START TIME WAS 35MIN POST INJECTION. PROTOCOL STATES "15MIN +- 5MIN". DELAY WAS DUE TO LOGISTICS OF GETTING 15 AND 30MIN BLOOD SAMPLES.
			036	DAY 35 VISIT WAS OUTSIDE THE PROTOCOL-DEFINED RANGE OF 30 +- 2 DAYS DUE TO PATIENT VACATION.
XVO_005	301	04AUG2009	OBD	15 MINUTE BIODISTRIBUTION WAS DELAYED DUE TO DIFFICULTY WITH BLOOD DRAW
			OPK	15 MINUTE PK WAS DELAYED DUE TO DIFFICULTY WITH BLOOD DRAW.

Listing 20
Study Comments

Center	Patient	Date	CRF Page	Comment
XVO_005	301	10AUG2009	030	SPECIFIC GRAVITY WAS DONE VIA DIPSTICK WHICH ONLY MEASURES UPTO 1.0303,SPECIFIC GRAVOTY WAS NOT PERFORMED ON A REFRACTOMETER.
		24SEP2009	019	15 MINUTE SCAN WAS DELAYED DUE TO DIFFICULTY WITH BLOOD DRAW.
	302	07JAN2010	018	30 MINUTE PROCEDURES WERE NOT DONE DUE TO SUBJECT BEING IN SCANNER DURING THE TIME POINT.
			029	DAY 06 PHYSICAL EXAMINATION NOT DONE DUE TO PI BEING AWAY FROM DUKE AT THIS TIME POINT.

Listing 21
Death Details

Center	Patient	Date of Death	Time of Death	Primary Cause of Death	Associated Morbidity/Condition	Reported by	Was an Autopsy Performed?

There were no deaths reported

Table 1
Summary of Eligibility and Treatment Status

All Patients (N = 8)

	I-131-CLR1404 N (%)
Number Enrolled	8
Number Treated	8 (100.0%)
Number Meeting all Study Entry Criteria	3 (37.5%)
Number Included in Safety Population	8 (100.0%)
Number Completing Study	8 (100.0%)
Early Termination	0 (0.0%)
Patient Withdrawal of Consent	0 (0.0%)
Disease Progression	0 (0.0%)
Adverse Event/Unacceptable Toxicity	0 (0.0%)
Lost to Follow-up	0 (0.0%)
Patient Pregnant or Breastfeeding	0 (0.0%)
Investigator Decision	0 (0.0%)
Other Reason	0 (0.0%)

Source Listings: 1, 2, 3, 8, 19
Note: Percentages are determined from a denominator of all patients enrolled for the number treated, meeting all study entry criteria and included in the safety population, and determined from a denominator of the number of patients in the safety population for the remaining categories.
Note: Three patients did not satisfy all criteria as samples for INR were not drawn at screening.

Table 2
Summary of Demographics and Baseline Characteristics

Safety Population (N = 8)

Characteristic		I-131-CLR1404 N (%)
Gender N (%)	Male	6 (75.0%)
	Female	2 (25.0%)
Ethnicity N (%) [a]	Hispanic or Latino	1 (12.5%)
	Multi-Racial	0 (0.0%)
	Other	0 (0.0%)
Race N (%) [a]	American Indian or Alaska Native	0 (0.0%)
	Asian	0 (0.0%)
	Black or African American	1 (12.5%)
	Native Hawaiin or Other Pacific Islander	0 (0.0%)
	White	6 (75.0%)
	Other	0 (0.0%)
Age (Years)	N	8
	Mean	59.5
	Std Dev	8.75
	Median	59.0
	Range	(46, 71)

Source Listing: 4
Note: All percentages are determined from a denominator of all patients in the safety population.
[a] For each patient, either an ethnicity category or a race category was recorded.

Table 3
Summary of Cancer Therapy History and Medical History

Safety Population (N = 8)

		I-131-CLR1404 N (%)
Patients with Medical/Surgical History [a]		8 (100.0%)
Prior Medical/Surgical History		8 (100.0%)
Ongoing Medical/Surgical History		8 (100.0%)
Patients with Prior Radiation Therapy		2 (25.0%)
Number of Radiation Therapy Cycles	N	8
	Mean	0.3
	Std Dev	0.46
	Median	0.0
	Range	(0, 1)

Source Listings: 5, 6.1
Note: All percentages are determined from a denominator of all patients in the safety population.
[a] Patients can be included in more than one category.
Note: Patients with no prior radiation therapy are included as zero cycles in the summary.

Table 4
Summary of Total Injected Dose Activity

Safety Population (N = 8)

		I-131-CLR1404 N (%)
Total Injected Dose Activity (mCi)	N	8
	Mean	10.2
	Std Dev	0.45
	Median	10.2
	Range	(9, 11)
Dose Delivered as Per Protocol		8 (100.0%)
Dose not Delivered as Per Protocol		0 (0.0%)
Adverse Event		0 (0.0%)
Patient Non-Compliance		0 (0.0%)
Withdrawal of Consent		0 (0.0%)
Technical Complications		0 (0.0%)
Other Reason		0 (0.0%)

Source Listing: 8
Note: Percentage of patients with dose delivered as per protocol is determined from a denominator of the safety population. All other percentages are determined from a denominator of all patients with dose not delivered as per protocol.
[a] Patients can be included in more than one category.

Table 5
Summary of Treatment Emergent Adverse Events

Safety Population (N= 8)

System Organ Class	Preferred Term	I-131-CLR1404 N (%)	Events
NUMBER OF PATIENTS WITH ADVERSE EVENTS		7 (87.5%)	19
GENERAL DISORDERS AND ADMINISTRATION SITE CONDITIONS	ALL	5 (62.5%)	5
	FATIGUE	4 (50.0%)	4
	OEDEMA PERIPHERAL	1 (12.5%)	1
SKIN AND SUBCUTANEOUS TISSUE DISORDERS	ALL	3 (37.5%)	4
	ERYTHEMA	2 (25.0%)	2
	SKIN ULCER	1 (12.5%)	2
INFECTIONS AND INFESTATIONS	ALL	3 (37.5%)	3
	ERYSIPELAS	1 (12.5%)	1
	HORDEOLUM	1 (12.5%)	1
	SIALOADENITIS	1 (12.5%)	1
RESPIRATORY, THORACIC AND MEDIASTINAL DISORDERS	ALL	2 (25.0%)	2
	NASAL CONGESTION	1 (12.5%)	1
	WHEEZING	1 (12.5%)	1

Source Listing: 9
Note: All percentages are determined from a denominator of all patients in the safety population.
If a patient has more than one event with a given preferred term, it is counted for each event in the count of events for that preferred term, and counted once for that patient in the count of patients for that preferred term. Similarly if a patient has more than one event with a given system organ class, it is counted for each event in the count of events for that system organ class, and counted once for that patient in the count of patients for that preferred term.

Table 5
Summary of Treatment Emergent Adverse Events

Safety Population (N= 8)

System Organ Class	Preferred Term	I-131-CLR1404 N (%)	Events
GASTROINTESTINAL DISORDERS	ALL	2 (25.0%)	2
	CONSTIPATION	1 (12.5%)	1
	DIARRHOEA	1 (12.5%)	1
INVESTIGATIONS	ALL	1 (12.5%)	2
	BLOOD PRESSURE DECREASED	1 (12.5%)	1
	GAMMA-GLUTAMYLTRANSFERASE INCREASED	1 (12.5%)	1
MUSCULOSKELETAL AND CONNECTIVE TISSUE DISORDERS	ALL	1 (12.5%)	1
	BACK PAIN	1 (12.5%)	1

Source Listing: 9
Note: All percentages are determined from a denominator of all patients in the safety population.
If a patient has more than one event with a given preferred term, it is counted for each event in the count of events for that preferred term, and counted once for that patient in the count of patients for that preferred term. Similarly if a patient has more than one event with a given system organ class, it is counted for each event in the count of events for that system organ class, and counted once for that patient in the count of patients for that preferred term.

Table 6
Summary of Treatment Emergent Adverse Events by Worst Severity

Safety Population (N= 8)

System Organ Class	Preferred Term	CTCAE Grade	I-131-CLR1404 N (%)	Events
GENERAL DISORDERS AND ADMINISTRATION SITE CONDITIONS	ALL	1	5 (62.5%)	5
		2	0 (0.0%)	0
		3	0 (0.0%)	0
		4	0 (0.0%)	0
		5	0 (0.0%)	0
	FATIGUE	1	4 (50.0%)	4
		2	0 (0.0%)	0
		3	0 (0.0%)	0
		4	0 (0.0%)	0
		5	0 (0.0%)	0
	OEDEMA PERIPHERAL	1	1 (12.5%)	1
		2	0 (0.0%)	0
		3	0 (0.0%)	0
		4	0 (0.0%)	0
		5	0 (0.0%)	0

Source Listing: 9
Note: All percentages are determined from a denominator of all patients in the safety population.
If a patient has more than one event with a given preferred term, it is counted for each event in the count of events for that preferred Term/CTCAE grade, and counted once for that patient in the count of patients for that preferred term/CTCAE grade. Similarly if a patient has more than one event with a given system organ class/CTCAE grade, it is counted for each event in the count of events for that system organ class/CTCAE grade, and counted once for that patient in the count of patients for that preferred term/CTCAE grade. Only the worst CTCAE grade for each term is summarized.

Table 6
Summary of Treatment Emergent Adverse Events by Worst Severity

Safety Population (N= 8)

System Organ Class	Preferred Term	CTCAE Grade	I-131-CLR1404 N (%)	Events
SKIN AND SUBCUTANEOUS TISSUE DISORDERS	ALL	1	3 (37.5%)	4
		2	0 (0.0%)	0
		3	0 (0.0%)	0
		4	0 (0.0%)	0
		5	0 (0.0%)	0
	ERYTHEMA	1	2 (25.0%)	2
		2	0 (0.0%)	0
		3	0 (0.0%)	0
		4	0 (0.0%)	0
		5	0 (0.0%)	0
	SKIN ULCER	1	1 (12.5%)	2
		2	0 (0.0%)	0
		3	0 (0.0%)	0
		4	0 (0.0%)	0
		5	0 (0.0%)	0

Source Listing: 9
Note: All percentages are determined from a denominator of all patients in the safety population.
If a patient has more than one event with a given preferred term, it is counted for each event in the count of events for that preferred Term/CTCAE grade, and counted once for that patient in the count of patients for that preferred term/CTCAE grade. Similarly if a patient has more than one event with a given system organ class/CTCAE grade, it is counted for each event in the count of events for that system organ class/CTCAE grade, and counted once for that patient in the count of patients for that preferred term/CTCAE grade. Only the worst CTCAE grade for each term is summarized.

Table 6
Summary of Treatment Emergent Adverse Events by Worst Severity

Safety Population (N= 8)

System Organ Class	Preferred Term	CTCAE Grade	I-131-CLR1404 N (%)	Events
INFECTIONS AND INFESTATIONS	ALL	1	3 (37.5%)	3
		2	0 (0.0%)	0
		3	0 (0.0%)	0
		4	0 (0.0%)	0
		5	0 (0.0%)	0
	ERYSIPELAS	1	1 (12.5%)	1
		2	0 (0.0%)	0
		3	0 (0.0%)	0
		4	0 (0.0%)	0
		5	0 (0.0%)	0
	HORDEOLUM	1	1 (12.5%)	1
		2	0 (0.0%)	0
		3	0 (0.0%)	0
		4	0 (0.0%)	0
		5	0 (0.0%)	0
	SIALOADENITIS	1	1 (12.5%)	1
		2	0 (0.0%)	0
		3	0 (0.0%)	0
		4	0 (0.0%)	0
		5	0 (0.0%)	0

Source Listing: 9
Note: All percentages are determined from a denominator of all patients in the safety population.
If a patient has more than one event with a given preferred term, it is counted for each event in the count of events for that preferred Term/CTCAE grade, and counted once for that patient in the count of patients for that preferred term/CTCAE grade. Similarly if a patient has more than one event with a given system organ class/CTCAE grade, it is counted for each event in the count of events for that system organ class/CTCAE grade, and counted once for that patient in the count of patients for that preferred term/CTCAE grade. Only the worst CTCAE grade for each term is summarized.

Table 6
Summary of Treatment Emergent Adverse Events by Worst Severity

Safety Population (N= 8)

System Organ Class	Preferred Term	CTCAE Grade	I-131-CLR1404 N (%)	Events
RESPIRATORY, THORACIC AND MEDIASTINAL DISORDERS	ALL	1	2 (25.0%)	2
		2	0 (0.0%)	0
		3	0 (0.0%)	0
		4	0 (0.0%)	0
		5	0 (0.0%)	0
	NASAL CONGESTION	1	1 (12.5%)	1
		2	0 (0.0%)	0
		3	0 (0.0%)	0
		4	0 (0.0%)	0
		5	0 (0.0%)	0
	WHEEZING	1	1 (12.5%)	1
		2	0 (0.0%)	0
		3	0 (0.0%)	0
		4	0 (0.0%)	0
		5	0 (0.0%)	0

Source Listing: 9
Note: All percentages are determined from a denominator of all patients in the safety population.
If a patient has more than one event with a given preferred term, it is counted for each event in the count of events for that preferred Term/CTCAE grade, and counted once for that patient in the count of patients for that preferred term/CTCAE grade. Similarly if a patient has more than one event with a given system organ class/CTCAE grade, it is counted for each event in the count of events for that system organ class/CTCAE grade, and counted once for that patient in the count of patients for that preferred term/CTCAE grade. Only the worst CTCAE grade for each term is summarized.

Table 6
Summary of Treatment Emergent Adverse Events by Worst Severity

Safety Population (N= 8)

System Organ Class	Preferred Term	CTCAE Grade	I-131-CLR1404 N (%)	Events
GASTROINTESTINAL DISORDERS	ALL	1	2 (25.0%)	2
		2	0 (0.0%)	0
		3	0 (0.0%)	0
		4	0 (0.0%)	0
		5	0 (0.0%)	0
	CONSTIPATION	1	1 (12.5%)	1
		2	0 (0.0%)	0
		3	0 (0.0%)	0
		4	0 (0.0%)	0
		5	0 (0.0%)	0
	DIARRHOEA	1	1 (12.5%)	1
		2	0 (0.0%)	0
		3	0 (0.0%)	0
		4	0 (0.0%)	0
		5	0 (0.0%)	0

Source Listing: 9
Note: All percentages are determined from a denominator of all patients in the safety population.
If a patient has more than one event with a given preferred term, it is counted for each event in the count of events for that preferred Term/CTCAE grade, and counted once for that patient in the count of patients for that preferred term/CTCAE grade. Similarly if a patient has more than one event with a given system organ class/CTCAE grade, it is counted for each event in the count of events for that system organ class/CTCAE grade, and counted once for that patient in the count of patients for that preferred term/CTCAE grade. Only the worst CTCAE grade for each term is summarized.

Table 6
Summary of Treatment Emergent Adverse Events by Worst Severity

Safety Population (N= 8)

System Organ Class	Preferred Term	CTCAE Grade	I-131-CLR1404 N (%)	Events
INVESTIGATIONS	ALL	1	1 (12.5%)	1
		2	1 (12.5%)	1
		3	0 (0.0%)	0
		4	0 (0.0%)	0
		5	0 (0.0%)	0
	BLOOD PRESSURE DECREASED	1	1 (12.5%)	1
		2	0 (0.0%)	0
		3	0 (0.0%)	0
		4	0 (0.0%)	0
		5	0 (0.0%)	0
	GAMMA-GLUTAMYLTRANSFERASE INCREASED	1	0 (0.0%)	0
		2	1 (12.5%)	1
		3	0 (0.0%)	0
		4	0 (0.0%)	0
		5	0 (0.0%)	0

Source Listing: 9
Note: All percentages are determined from a denominator of all patients in the safety population.
If a patient has more than one event with a given preferred term, it is counted for each event in the count of events for that preferred Term/CTCAE grade, and counted once for that patient in the count of patients for that preferred term/CTCAE grade. Similarly if a patient has more than one event with a given system organ class/CTCAE grade, it is counted for each event in the count of events for that system organ class/CTCAE grade, and counted once for that patient in the count of patients for that preferred term/CTCAE grade. Only the worst CTCAE grade for each term is summarized.

Table 6
Summary of Treatment Emergent Adverse Events by Worst Severity

Safety Population (N= 8)

System Organ Class	Preferred Term	CTCAE Grade	I-131-CLR1404 N (%)	Events
MUSCULOSKELETAL AND CONNECTIVE TISSUE DISORDERS	ALL	1	0 (0.0%)	0
		2	1 (12.5%)	1
		3	0 (0.0%)	0
		4	0 (0.0%)	0
		5	0 (0.0%)	0
	BACK PAIN	1	0 (0.0%)	0
		2	1 (12.5%)	1
		3	0 (0.0%)	0
		4	0 (0.0%)	0
		5	0 (0.0%)	0

Source Listing: 9
Note: All percentages are determined from a denominator of all patients in the safety population.
If a patient has more than one event with a given preferred term, it is counted for each event in the count of events for that preferred Term/CTCAE grade, and counted once for that patient in the count of patients for that preferred term/CTCAE grade. Similarly if a patient has more than one event with a given system organ class/CTCAE grade, it is counted for each event in the count of events for that system organ class/CTCAE grade, and counted once for that patient in the count of patients for that preferred term/CTCAE grade. Only the worst CTCAE grade for each term is summarized.

Table 7
Summary of Treatment Emergent Adverse Events by Relationship to Study Drug

Safety Population (N= 8)

System Organ Class	Preferred Term	Relationship	I-131-CLR1404 N (%)	Events
GENERAL DISORDERS AND ADMINISTRATION SITE CONDITIONS	ALL	Related	3 (37.5%)	3
		Not Related	2 (25.0%)	2
	FATIGUE	Related	3 (37.5%)	3
		Not Related	1 (12.5%)	1
	OEDEMA PERIPHERAL	Related	0 (0.0%)	0
		Not Related	1 (12.5%)	1
SKIN AND SUBCUTANEOUS TISSUE DISORDERS	ALL	Related	0 (0.0%)	0
		Not Related	3 (37.5%)	4
	ERYTHEMA	Related	0 (0.0%)	0
		Not Related	2 (25.0%)	2
	SKIN ULCER	Related	0 (0.0%)	0
		Not Related	1 (12.5%)	2
INFECTIONS AND INFESTATIONS	ALL	Related	1 (12.5%)	1
		Not Related	2 (25.0%)	2
	ERYSIPELAS	Related	0 (0.0%)	0
		Not Related	1 (12.5%)	1
	HORDEOLUM	Related	0 (0.0%)	0
		Not Related	1 (12.5%)	1
	SIALOADENITIS	Related	1 (12.5%)	1
		Not Related	0 (0.0%)	0
RESPIRATORY, THORACIC AND MEDIASTINAL DISORDERS	ALL	Related	0 (0.0%)	0
		Not Related	2 (25.0%)	2
	NASAL CONGESTION	Related	0 (0.0%)	0
		Not Related	1 (12.5%)	1

Source Listing: 9
Note: All percentages are determined from a denominator of all patients in the safety population.
If a patient has more than one event with a given preferred term, it is counted for each event in the count of events for that preferred term, and counted once for that patient in the count of patients for that preferred term. Similarly if a patient has more than one event with a given system organ class, it is counted for each event in the count of events for that system organ class, and counted once for that patient in the count of patients for that preferred term.

Table 7
Summary of Treatment Emergent Adverse Events by Relationship to Study Drug

Safety Population (N= 8)

System Organ Class	Preferred Term	Relationship	I-131-CLR1404 N (%)	Events
RESPIRATORY, THORACIC AND MEDIASTINAL DISORDERS	WHEEZING	Related	0 (0.0%)	0
		Not Related	1 (12.5%)	1
GASTROINTESTINAL DISORDERS	ALL	Related	1 (12.5%)	1
		Not Related	1 (12.5%)	1
	CONSTIPATION	Related	1 (12.5%)	1
		Not Related	0 (0.0%)	0
	DIARRHOEA	Related	0 (0.0%)	0
		Not Related	1 (12.5%)	1
INVESTIGATIONS	ALL	Related	0 (0.0%)	0
		Not Related	1 (12.5%)	2
	BLOOD PRESSURE DECREASED	Related	0 (0.0%)	0
		Not Related	1 (12.5%)	1
	GAMMA-GLUTAMYLTRANSFERASE INCREASED	Related	0 (0.0%)	0
		Not Related	1 (12.5%)	1
MUSCULOSKELETAL AND CONNECTIVE TISSUE DISORDERS	ALL	Related	0 (0.0%)	0
		Not Related	1 (12.5%)	1
	BACK PAIN	Related	0 (0.0%)	0
		Not Related	1 (12.5%)	1

Source Listing: 9
Note: All percentages are determined from a denominator of all patients in the safety population.
If a patient has more than one event with a given preferred term, it is counted for each event in the count of events for that preferred term, and counted once for that patient in the count of patients for that preferred term. Similarly if a patient has more than one event with a given system organ class, it is counted for each event in the count of events for that system organ class, and counted once for that patient in the count of patients for that preferred term.

Table 8
Summary of Treatment Emergent Serious Adverse Events

Safety Population (N= 8)

System Organ Class	Preferred Term	I-131-CLR1404 N (%)	Events

There were no Treatment Emergent Serious Adverse Events reported

Source Listing: 9
Note: All percentages are determined from a denominator of all patients in the safety population.
If a patient has more than one event with a given preferred term, it is counted for each event in the count of events for that preferred term, and counted once for that patient in the count of patients for that preferred term. Similarly if a patient has more than one event with a given system organ class, it is counted for each event in the count of events for that system organ class, and counted once for that patient in the count of patients for that preferred term.

Table 9
Summary of Treatment Emergent Adverse Events Leading to Termination of Study Drug

Safety Population (N= 8)

System Organ Class	Preferred Term	I-131-CLR1404 N (%)	Events

There were no Treatment Emergent Adverse Events Leading to Termination of Study Drug

Source Listing: 9
Note: All percentages are determined from a denominator of all patients in the safety population.
If a patient has more than one event with a given preferred term, it is counted for each event in the count of events for that preferred term, and counted once for that patient in the count of patients for that preferred term. Similarly if a patient has more than one event with a given system organ class, it is counted for each event in the count of events for that system organ class, and counted once for that patient in the count of patients for that preferred term.

Table 10
Summary of Dose Limiting Toxicities [a]

Safety Population (N= 8)

System Organ Class	Preferred Term	I-131-CLR1404 N (%)	Events

There were no Dose Limiting Toxicities

Source Listing: 9
Note: All percentages are determined from a denominator of all patients in the safety population.
If a patient has more than one event with a given preferred term, it is counted for each event in the count of events for that preferred term, and counted once for that patient in the count of patients for that preferred term. Similarly if a patient has more than one event with a given system organ class, it is counted for each event in the count of events for that system organ class, and counted once for that patient in the count of patients for that preferred term.
[a] Dose limiting toxicities are defined for hematological events as any CTCAE grade 4 toxicity (except neutropenia without concurrent fever which had to be present for at least 7 days), CTCAE grade 3 neutropenia with fever or CTCAE grade 3 thrombocytopenia associated with bleeding. Dose limiting toxicities are defined for non-hematological events as any CTCAE grade 3 toxicity despite adequate supportive care (supportive care limited to nausea, vomiting and diarrhea only) or any CTCAE grade 4 toxicity

Table 11.1
Summary of Laboratory Data - Quantitative

Safety Population (N = 8)

Laboratory Type	Parameter	Visit	I-131-CLR1404 N	Mean	Std Dev	Median	Range
Clinical Chemistry	Sodium (mEq/L)	Screening	8	138.875	1.1260	139.000	(137.00, 140.00)
		Baseline	6	138.500	1.6432	138.500	(136.00, 141.00)
		Day 6	8	137.250	2.7124	137.000	(133.00, 140.00)
		Day 14	7	138.000	2.1602	138.000	(134.00, 141.00)
		Day 30	7	136.857	2.0354	137.000	(134.00, 140.00)
		Day 42	7	137.143	1.5736	137.000	(135.00, 139.00)
	Sodium (mEq/L) Change from Baseline	Day 6	8	-1.250	2.3755	-1.500	(-4.00, 2.00)
		Day 14	7	-0.143	2.1157	-1.000	(-2.00, 4.00)
		Day 30	7	-1.286	1.7995	-1.000	(-4.00, 1.00)
		Day 42	7	-1.000	1.6330	-1.000	(-4.00, 1.00)
	Potassium (mEq/L)	Screening	8	4.213	0.3603	4.000	(3.90, 4.80)
		Baseline	6	4.383	0.6706	4.250	(3.40, 5.40)
		Day 6	8	4.163	0.4749	4.200	(3.50, 4.90)
		Day 14	7	4.186	0.2545	4.100	(3.80, 4.50)
		Day 30	7	4.171	0.3402	4.100	(3.90, 4.90)
		Day 42	7	4.314	0.5178	4.100	(3.70, 5.10)
	Potassium (mEq/L) Change from Baseline	Day 6	8	-0.175	0.3808	-0.050	(-0.80, 0.30)
		Day 14	7	0.000	0.3317	-0.100	(-0.40, 0.50)
		Day 30	7	-0.014	0.2968	-0.100	(-0.30, 0.50)
		Day 42	7	0.129	0.4990	0.000	(-0.50, 0.90)

Source Listing: 10.1, 10.2, 10.3, 10.4

Table 11.1
Summary of Laboratory Data - Quantitative

Safety Population (N = 8)

			I-131-CLR1404				
Laboratory Type	Parameter	Visit	N	Mean	Std Dev	Median	Range
Clinical Chemistry	Total Protein(G/DL)	Screening	8	6.925	0.5175	6.750	(6.20, 7.90)
		Baseline	6	6.933	0.5820	6.950	(6.10, 7.70)
		Day 6	8	6.813	0.2100	6.800	(6.50, 7.10)
		Day 14	7	6.614	0.4059	6.500	(6.20, 7.30)
		Day 30	7	6.900	0.3606	7.000	(6.50, 7.50)
		Day 42	7	6.829	0.3302	6.700	(6.40, 7.20)
	Total Protein(G/DL) Change from Baseline	Day 6	8	-0.100	0.3546	-0.100	(-0.70, 0.40)
		Day 14	7	-0.229	0.2059	-0.300	(-0.40, 0.10)
		Day 30	7	0.057	0.3101	0.100	(-0.50, 0.40)
		Day 42	7	-0.014	0.3934	0.100	(-0.50, 0.60)
	Bicarbionate (mEq/L)	Screening	8	26.000	2.8284	27.000	(21.00, 29.00)
		Baseline	6	25.500	3.5637	25.500	(21.00, 31.00)
		Day 6	8	25.250	2.7124	25.000	(22.00, 30.00)
		Day 14	7	26.000	3.0000	27.000	(22.00, 30.00)
		Day 30	7	26.286	3.2514	26.000	(21.00, 30.00)
		Day 42	7	25.429	3.5051	26.000	(21.00, 29.00)
	Bicarbionate (mEq/L) Change from Baseline	Day 6	8	-0.250	2.0529	-1.000	(-3.00, 3.00)
		Day 14	7	0.714	2.5635	2.000	(-4.00, 3.00)
		Day 30	7	1.000	2.5820	1.000	(-2.00, 6.00)
		Day 42	7	0.143	2.3401	0.000	(-3.00, 4.00)

Source Listing: 10.1, 10.2, 10.3, 10.4

Table 11.1
Summary of Laboratory Data - Quantitative

Safety Population (N = 8)

Laboratory Type	Parameter	Visit	I-131-CLR1404 N	Mean	Std Dev	Median	Range
Clinical Chemistry	BUN (mg/dL)	Screening	8	16.750	6.8191	16.500	(5.00, 27.00)
		Baseline	6	19.333	8.2865	19.500	(7.00, 28.00)
		Day 6	8	16.000	6.9693	16.500	(5.00, 27.00)
		Day 14	7	15.286	6.1023	14.000	(5.00, 24.00)
		Day 30	7	16.000	5.5976	17.000	(5.00, 22.00)
		Day 42	7	15.571	6.6045	14.000	(7.00, 27.00)
	BUN (mg/dL) Change from Baseline	Day 6	8	-2.375	2.8253	-1.500	(-9.00, 0.00)
		Day 14	7	-1.714	3.0394	-1.000	(-7.00, 2.00)
		Day 30	7	-1.000	4.2817	-1.000	(-6.00, 7.00)
		Day 42	7	-1.429	2.5071	-1.000	(-6.00, 2.00)
	Glucose (mg/dL)	Screening	8	105.875	28.6727	93.000	(81.00, 161.00)
		Baseline	6	112.000	22.6892	105.000	(94.00, 157.00)
		Day 6	8	115.250	37.3698	105.000	(77.00, 200.00)
		Day 14	7	103.143	39.8641	93.000	(72.00, 190.00)
		Day 30	7	100.143	20.2355	91.000	(85.00, 144.00)
		Day 42	7	125.143	53.1960	99.000	(86.00, 205.00)
	Glucose (mg/dL) Change from Baseline	Day 6	8	9.750	21.7239	5.500	(-25.00, 43.00)
		Day 14	7	-2.000	20.3142	-1.000	(-24.00, 33.00)
		Day 30	7	-5.000	8.9443	-5.000	(-17.00, 5.00)
		Day 42	7	20.000	45.7530	13.000	(-22.00, 111.00)

Source Listing: 10.1, 10.2, 10.3, 10.4

Table 11.1
Summary of Laboratory Data - Quantitative

Safety Population (N = 8)

			I-131-CLR1404				
Laboratory Type	Parameter	Visit	N	Mean	Std Dev	Median	Range
Clinical Chemistry	GGT (U/L)	Screening	8	41.125	22.6743	44.000	(11.00, 68.00)
		Baseline	7	44.143	17.9018	49.000	(22.00, 68.00)
		Day 6	8	49.250	48.5673	39.500	(8.00, 163.00)
		Day 14	8	37.375	20.5631	33.000	(12.00, 74.00)
		Day 30	8	39.000	21.4609	36.000	(12.00, 74.00)
		Day 42	7	37.000	20.3388	32.000	(18.00, 74.00)
	GGT (U/L) Change from Baseline	Day 6	8	9.250	38.0517	-2.000	(-10.00, 103.00)
		Day 14	8	-2.625	10.4326	-0.500	(-17.00, 14.00)
		Day 30	8	-1.000	9.1807	0.000	(-19.00, 14.00)
		Day 42	7	-7.143	13.2342	-4.000	(-27.00, 14.00)
	Calcium (mg/dL)	Screening	8	9.275	0.2493	9.300	(8.80, 9.60)
		Baseline	6	9.400	0.4243	9.350	(9.00, 10.20)
		Day 6	8	9.163	0.2722	9.200	(8.80, 9.50)
		Day 14	7	9.071	0.2812	9.000	(8.70, 9.50)
		Day 30	7	8.957	0.2699	8.900	(8.50, 9.30)
		Day 42	7	9.014	0.2795	8.900	(8.70, 9.40)
	Calcium (mg/dL) Change from Baseline	Day 6	8	-0.188	0.3271	-0.050	(-0.80, 0.20)
		Day 14	7	-0.157	0.2070	-0.200	(-0.40, 0.20)
		Day 30	7	-0.271	0.3450	-0.100	(-0.90, 0.00)
		Day 42	7	-0.214	0.2911	-0.200	(-0.60, 0.10)

Source Listing: 10.1, 10.2, 10.3, 10.4

Table 11.1
Summary of Laboratory Data - Quantitative

Safety Population (N = 8)

Laboratory Type	Parameter	Visit	I-131-CLR1404 N	Mean	Std Dev	Median	Range
Clinical Chemistry	Chloride (MEQ/L)	Screening	8	104.500	2.9761	103.500	(102.00, 111.00)
		Baseline	6	104.000	3.7947	103.000	(100.00, 111.00)
		Day 6	8	103.750	3.2404	103.000	(100.00, 111.00)
		Day 14	7	104.000	3.5590	105.000	(100.00, 110.00)
		Day 30	7	102.286	2.7516	102.000	(99.00, 108.00)
		Day 42	7	103.286	4.4987	102.000	(97.00, 112.00)
	Chloride (MEQ/L) Change from Baseline	Day 6	8	-0.375	1.9955	0.000	(-4.00, 3.00)
		Day 14	7	0.000	2.1602	-1.000	(-2.00, 3.00)
		Day 30	7	-1.714	2.4300	-1.000	(-5.00, 2.00)
		Day 42	7	-0.714	3.1472	1.000	(-6.00, 2.00)
	Albumin (mg/dL)	Screening	8	3850.000	226.7787	3850.000	(3500.00, 4200.00)
		Baseline	6	3816.667	248.3277	3750.000	(3500.00, 4200.00)
		Day 6	8	3775.000	183.2251	3850.000	(3500.00, 4000.00)
		Day 14	7	3628.571	228.8689	3700.000	(3300.00, 3800.00)
		Day 30	7	3871.429	197.6047	3900.000	(3500.00, 4100.00)
		Day 42	7	3771.429	188.9822	3700.000	(3500.00, 4100.00)
	Albumin (mg/dL) Change from Baseline	Day 6	8	-37.500	176.7767	-50.000	(-300.00, 200.00)
		Day 14	7	-128.571	179.9471	-200.000	(-400.00, 100.00)
		Day 30	7	114.286	157.3592	100.000	(-100.00, 300.00)
		Day 42	7	14.286	157.3592	0.000	(-200.00, 200.00)

Source Listing: 10.1, 10.2, 10.3, 10.4

Table 11.1
Summary of Laboratory Data - Quantitative

Safety Population (N = 8)

			I-131-CLR1404				
Laboratory Type	Parameter	Visit	N	Mean	Std Dev	Median	Range
Clinical Chemistry	Total Bilirubin (mg/dL)	Screening	8	0.563	0.2560	0.550	(0.20, 0.90)
		Baseline	6	0.633	0.2066	0.500	(0.50, 0.90)
		Day 6	8	0.588	0.2588	0.600	(0.20, 1.00)
		Day 14	7	0.571	0.1604	0.500	(0.40, 0.90)
		Day 30	7	0.643	0.2149	0.700	(0.30, 1.00)
		Day 42	7	0.629	0.1704	0.600	(0.40, 0.80)
	Total Bilirubin (mg/dL) Change from Baseline	Day 6	8	-0.100	0.2204	-0.100	(-0.50, 0.20)
		Day 14	7	-0.143	0.1902	-0.100	(-0.40, 0.10)
		Day 30	7	-0.071	0.1380	-0.100	(-0.20, 0.10)
		Day 42	7	-0.086	0.1069	-0.100	(-0.30, 0.00)
	Alkaline Phosphatase (U/L)	Screening	8	116.500	82.5919	81.500	(50.00, 297.00)
		Baseline	6	128.167	76.9868	101.000	(52.00, 256.00)
		Day 6	8	106.875	58.7305	84.000	(48.00, 199.00)
		Day 14	7	94.000	58.5320	75.000	(51.00, 220.00)
		Day 30	7	105.429	80.8926	77.000	(61.00, 285.00)
		Day 42	7	106.857	85.6085	76.000	(53.00, 298.00)
	Alkaline Phosphatase (U/L) Change from Baseline	Day 6	8	-6.875	36.0176	-3.000	(-85.00, 40.00)
		Day 14	7	0.571	16.7118	-3.000	(-15.00, 37.00)
		Day 30	7	12.000	40.2823	-1.000	(-14.00, 102.00)
		Day 42	7	13.429	45.1621	-2.000	(-11.00, 115.00)

Source Listing: 10.1, 10.2, 10.3, 10.4

Table 11.1
Summary of Laboratory Data - Quantitative

Safety Population (N = 8)

			I-131-CLR1404				
Laboratory Type	Parameter	Visit	N	Mean	Std Dev	Median	Range
Clinical Chemistry	AST (U/L)	Screening	8	25.875	11.0768	22.500	(17.00, 52.00)
		Baseline	6	26.667	7.3394	26.000	(18.00, 40.00)
		Day 6	8	27.375	8.3313	23.500	(17.00, 43.00)
		Day 14	7	22.571	4.3150	24.000	(14.00, 28.00)
		Day 30	7	23.429	4.5774	24.000	(17.00, 29.00)
		Day 42	7	24.429	5.5032	23.000	(16.00, 32.00)
	AST (U/L) Change from Baseline	Day 6	8	2.000	4.5670	1.500	(-3.00, 11.00)
		Day 14	7	-0.714	2.6904	-1.000	(-4.00, 4.00)
		Day 30	7	0.143	4.9135	-1.000	(-6.00, 9.00)
		Day 42	7	1.143	5.1455	1.000	(-5.00, 8.00)
	ALT (U/L)	Screening	8	18.750	9.1456	16.500	(9.00, 37.00)
		Baseline	6	18.667	6.3456	18.000	(11.00, 29.00)
		Day 6	8	20.625	7.0089	18.500	(14.00, 36.00)
		Day 14	7	16.714	8.0976	15.000	(10.00, 34.00)
		Day 30	7	18.143	13.8856	15.000	(8.00, 49.00)
		Day 42	7	18.000	10.4243	15.000	(10.00, 40.00)
	ALT (U/L) Change from Baseline	Day 6	8	-0.375	3.5026	-1.500	(-4.00, 7.00)
		Day 14	7	-3.143	1.7728	-3.000	(-6.00, -1.00)
		Day 30	7	-1.714	6.9693	-3.000	(-9.00, 12.00)
		Day 42	7	-1.857	3.7161	-2.000	(-7.00, 3.00)

Source Listing: 10.1, 10.2, 10.3, 10.4

Table 11.1
Summary of Laboratory Data - Quantitative

Safety Population (N = 8)

Laboratory Type	Parameter	Visit	I-131-CLR1404 N	Mean	Std Dev	Median	Range
Clinical Chemistry	Creatinine (mg/dL)	Screening	8	0.998	0.3267	0.915	(0.60, 1.60)
		Baseline	6	0.977	0.1973	1.000	(0.62, 1.20)
		Day 6	8	1.026	0.2855	1.100	(0.60, 1.30)
		Day 14	7	0.983	0.2899	1.000	(0.58, 1.30)
		Day 30	7	0.996	0.2997	1.100	(0.60, 1.30)
		Day 42	7	0.943	0.2440	1.000	(0.60, 1.20)
	Creatinine (mg/dL) Change from Baseline	Day 6	8	0.069	0.0996	0.100	(-0.13, 0.20)
		Day 14	7	0.023	0.0547	0.000	(-0.04, 0.10)
		Day 30	7	0.036	0.1107	0.100	(-0.20, 0.10)
		Day 42	7	-0.017	0.0373	0.000	(-0.10, 0.00)
Hematology	Hemoglobin (g/dL)	Screening	8	13.400	0.8896	13.500	(12.00, 15.00)
		Baseline	6	13.483	0.8954	13.700	(12.10, 14.60)
		Day 6	8	13.075	0.9982	13.050	(11.50, 14.80)
		Day 14	8	12.975	0.9750	13.200	(11.40, 14.20)
		Day 30	7	13.214	1.1261	13.600	(11.10, 14.60)
		Day 42	8	13.238	1.1426	13.600	(10.80, 14.60)
	Hemoglobin (g/dL) Change from Baseline	Day 6	8	-0.588	0.6664	-0.400	(-1.80, 0.10)
		Day 14	8	-0.688	0.3871	-0.650	(-1.40, -0.30)
		Day 30	7	-0.429	0.7111	-0.400	(-1.70, 0.60)
		Day 42	8	-0.425	0.7459	-0.250	(-2.00, 0.40)

Source Listing: 10.1, 10.2, 10.3, 10.4

Table 11.1
Summary of Laboratory Data - Quantitative

Safety Population (N = 8)

Laboratory Type	Parameter	Visit	I-131-CLR1404 N	Mean	Std Dev	Median	Range
Hematology	Hematocrit (%)	Screening	8	39.913	2.3877	40.050	(36.00, 42.90)
		Baseline	6	40.283	2.6339	40.000	(36.30, 43.20)
		Day 6	8	39.000	3.3840	38.350	(34.00, 44.50)
		Day 14	8	38.688	2.9367	38.850	(34.60, 42.50)
		Day 30	7	39.629	3.8595	40.000	(33.00, 44.90)
		Day 42	8	39.700	3.5557	40.100	(33.00, 44.00)
	Hematocrit (%) Change from Baseline	Day 6	8	-1.575	2.6847	-1.750	(-5.20, 2.00)
		Day 14	8	-1.888	1.3943	-1.950	(-4.00, 0.00)
		Day 30	7	-1.029	2.8646	-0.200	(-6.00, 2.10)
		Day 42	8	-0.875	2.5070	0.300	(-6.00, 1.20)
	Red Blood Count (10x6/uL)	Screening	8	4.474	0.3977	4.550	(3.97, 5.19)
		Baseline	6	4.605	0.3826	4.525	(4.27, 5.18)
		Day 6	8	4.379	0.4069	4.285	(3.83, 4.93)
		Day 14	8	4.348	0.4446	4.300	(3.84, 5.14)
		Day 30	7	4.420	0.5189	4.490	(3.72, 5.24)
		Day 42	8	4.449	0.4817	4.500	(3.67, 5.33)
	Red Blood Count (10x6/uL) Change from Baseline	Day 6	8	-0.165	0.2326	-0.125	(-0.52, 0.15)
		Day 14	8	-0.196	0.1278	-0.180	(-0.44, -0.04)
		Day 30	7	-0.093	0.2646	-0.020	(-0.56, 0.20)
		Day 42	8	-0.095	0.2637	-0.065	(-0.61, 0.15)

Source Listing: 10.1, 10.2, 10.3, 10.4

Table 11.1
Summary of Laboratory Data - Quantitative

Safety Population (N = 8)

Laboratory Type	Parameter	Visit	I-131-CLR1404 N	Mean	Std Dev	Median	Range
Hematology	MCV (FL)	Screening	8	89.725	5.0273	90.450	(82.60, 97.70)
		Baseline	6	87.583	4.2692	86.850	(83.20, 93.60)
		Day 6	8	89.313	4.4585	88.850	(83.30, 96.30)
		Day 14	8	89.125	4.7972	88.600	(81.80, 95.90)
		Day 30	7	89.900	5.4693	89.000	(82.00, 97.00)
		Day 42	8	89.413	5.1285	88.850	(82.30, 95.50)
	MCV (FL) Change from Baseline	Day 6	8	-0.187	1.5245	-0.450	(-1.50, 3.00)
		Day 14	8	-0.375	1.5069	-1.000	(-1.90, 2.00)
		Day 30	7	-0.386	1.3005	-0.700	(-2.00, 1.70)
		Day 42	8	-0.088	1.6348	-0.550	(-2.20, 2.40)
	MCH (PG)	Screening	8	30.088	2.1970	29.750	(26.60, 32.70)
		Baseline	6	29.317	1.6339	29.450	(27.00, 31.70)
		Day 6	8	29.963	1.8205	29.850	(27.10, 32.90)
		Day 14	8	30.025	2.3566	29.750	(25.80, 33.30)
		Day 30	7	30.043	2.4027	30.000	(26.00, 33.20)
		Day 42	8	29.888	2.1404	29.700	(26.00, 32.60)
	MCH (PG) Change from Baseline	Day 6	8	-0.188	0.6244	-0.100	(-1.50, 0.60)
		Day 14	8	-0.125	0.7555	-0.050	(-1.20, 0.70)
		Day 30	7	-0.271	0.8420	-0.100	(-1.80, 0.50)
		Day 42	8	-0.263	0.7070	-0.300	(-1.00, 0.90)

Source Listing: 10.1, 10.2, 10.3, 10.4

Table 11.1
Summary of Laboratory Data - Quantitative

Safety Population (N = 8)

Laboratory Type	Parameter	Visit	I-131-CLR1404 N	Mean	Std Dev	Median	Range
Hematology	MCHC (PG)	Screening	8	33.513	0.9031	33.300	(32.20, 35.10)
		Baseline	6	30.367	7.9482	33.550	(14.20, 34.50)
		Day 6	8	33.550	0.5425	33.600	(32.60, 34.10)
		Day 14	8	33.613	1.0656	33.600	(31.50, 34.90)
		Day 30	7	33.400	0.9747	33.600	(31.70, 34.30)
		Day 42	8	33.400	0.9754	33.400	(31.60, 34.60)
	MCHC (PG) Change from Baseline	Day 6	8	2.200	6.9284	0.150	(-1.80, 19.20)
		Day 14	8	2.263	6.7534	0.250	(-1.60, 18.80)
		Day 30	7	2.500	7.5313	0.400	(-2.50, 19.40)
		Day 42	8	2.050	6.6567	0.000	(-1.90, 18.40)
	White Blood Count (10x3/uL)	Screening	8	6.908	1.9083	6.730	(4.30, 10.70)
		Baseline	6	7.915	3.0760	6.850	(4.60, 12.60)
		Day 6	8	7.199	1.9174	7.700	(4.30, 9.70)
		Day 14	8	6.395	1.6943	6.550	(4.10, 9.20)
		Day 30	7	6.699	2.3178	6.100	(3.70, 10.00)
		Day 42	8	6.568	1.6264	6.570	(4.40, 9.10)
	White Blood Count (10x3/uL) Change from Baseline	Day 6	8	-0.513	2.2306	-0.450	(-3.90, 2.50)
		Day 14	8	-1.316	1.6355	-1.000	(-3.80, 0.90)
		Day 30	7	-1.186	0.9155	-1.000	(-2.60, 0.20)
		Day 42	8	-1.144	1.8286	-1.350	(-3.50, 1.40)

Source Listing: 10.1, 10.2, 10.3, 10.4

Table 11.1
Summary of Laboratory Data - Quantitative

Safety Population (N = 8)

Laboratory Type	Parameter	Visit	N	Mean	Std Dev	Median	Range
			I-131-CLR1404				
Hematology	Lymphocytes (%)	Screening	8	27.925	7.1159	31.100	(19.00, 37.60)
		Baseline	6	22.467	8.7995	19.950	(15.50, 39.20)
		Day 6	8	21.713	8.5362	22.300	(7.30, 30.90)
		Day 14	8	21.688	5.6819	21.650	(14.80, 28.10)
		Day 30	7	23.443	7.2863	23.900	(15.10, 32.60)
		Day 42	8	21.838	6.1176	24.150	(10.80, 28.50)
	Lymphocytes (%) Change from Baseline	Day 6	8	-3.138	5.4450	-2.200	(-13.40, 3.00)
		Day 14	8	-3.163	5.5017	-2.950	(-11.10, 3.50)
		Day 30	7	-2.743	4.0505	-4.100	(-7.00, 3.00)
		Day 42	8	-3.013	7.1941	-2.600	(-14.70, 4.60)
	Monocytes (%)	Screening	8	9.075	3.0556	8.000	(6.20, 15.70)
		Baseline	6	9.217	3.3725	8.250	(5.80, 15.60)
		Day 6	8	8.825	2.1238	8.000	(7.30, 13.60)
		Day 14	8	10.500	5.7309	8.800	(7.30, 24.50)
		Day 30	7	10.543	3.8621	8.300	(8.00, 18.50)
		Day 42	8	8.288	1.2206	8.350	(6.70, 10.70)
	Monocytes (%) Change from Baseline	Day 6	8	-0.313	1.3726	-0.150	(-2.50, 1.70)
		Day 14	8	1.363	3.2544	0.400	(-1.10, 8.90)
		Day 30	7	1.200	1.9570	0.700	(-1.40, 4.10)
		Day 42	8	-0.850	2.2791	-0.700	(-4.90, 3.10)

Source Listing: 10.1, 10.2, 10.3, 10.4

Table 11.1
Summary of Laboratory Data - Quantitative

Safety Population (N = 8)

Laboratory Type	Parameter	Visit	I-131-CLR1404 N	Mean	Std Dev	Median	Range
Hematology	Eosinophils (%)	Screening	8	3.725	2.3169	3.800	(0.90, 6.90)
		Baseline	6	4.500	1.9759	4.200	(1.50, 6.80)
		Day 6	8	3.775	2.4283	4.250	(0.90, 7.30)
		Day 14	8	4.100	2.4071	3.600	(1.20, 7.90)
		Day 30	7	3.357	2.1031	3.500	(0.80, 6.20)
		Day 42	8	3.725	2.6671	2.650	(1.30, 7.30)
	Eosinophils (%) Change from Baseline	Day 6	8	-0.375	1.2464	-0.250	(-3.10, 1.00)
		Day 14	8	-0.050	1.0836	-0.350	(-1.30, 1.80)
		Day 30	7	-0.414	0.7267	-0.600	(-1.40, 1.00)
		Day 42	8	-0.425	1.6193	-0.350	(-2.90, 2.20)
	Basophils (%)	Screening	8	0.650	0.3854	0.500	(0.30, 1.50)
		Baseline	6	0.517	0.2041	0.500	(0.30, 0.80)
		Day 6	8	0.663	0.3114	0.600	(0.40, 1.40)
		Day 14	8	0.600	0.2449	0.600	(0.20, 1.00)
		Day 30	7	0.729	0.4716	0.400	(0.40, 1.40)
		Day 42	8	0.650	0.3505	0.600	(0.30, 1.40)
	Basophils (%) Change from Baseline	Day 6	8	0.025	0.2375	0.000	(-0.40, 0.30)
		Day 14	8	-0.038	0.4470	0.100	(-0.90, 0.50)
		Day 30	7	0.071	0.2498	0.000	(-0.10, 0.60)
		Day 42	8	0.012	0.2416	-0.100	(-0.20, 0.50)

Source Listing: 10.1, 10.2, 10.3, 10.4

Table 11.1
Summary of Laboratory Data - Quantitative

Safety Population (N = 8)

Laboratory Type	Parameter	Visit	I-131-CLR1404 N	Mean	Std Dev	Median	Range
Hematology	ANC (10x3/uL)	Screening	8	4.081	1.4071	3.850	(2.20, 7.00)
		Baseline	6	5.173	2.5390	4.450	(2.20, 8.70)
		Day 6	8	4.736	1.5849	4.800	(2.40, 6.49)
		Day 14	8	4.045	1.2032	3.800	(2.60, 6.00)
		Day 30	7	4.174	1.6719	3.700	(2.00, 6.50)
		Day 42	8	4.335	1.2715	4.200	(2.80, 6.00)
	ANC (10x3/uL) Change from Baseline	Day 6	8	-0.106	2.0207	0.150	(-3.10, 3.05)
		Day 14	8	-0.798	1.3788	-0.440	(-3.00, 0.70)
		Day 30	7	-0.717	0.8768	-0.320	(-2.40, 0.20)
		Day 42	8	-0.508	1.7114	-0.600	(-2.90, 1.94)
	Platelets (10x3/uL)	Screening	8	264.500	63.1664	271.500	(185.00, 356.00)
		Baseline	6	267.667	73.1182	262.000	(169.00, 375.00)
		Day 6	8	270.875	64.9977	278.000	(197.00, 375.00)
		Day 14	8	274.875	69.5443	299.000	(186.00, 361.00)
		Day 30	7	239.000	41.8171	232.000	(184.00, 303.00)
		Day 42	8	234.250	36.8191	227.000	(192.00, 289.00)
	Platelets (10x3/uL) Change from Baseline	Day 6	8	9.375	37.9245	-1.500	(-33.00, 90.00)
		Day 14	8	13.375	39.1332	2.000	(-23.00, 96.00)
		Day 30	7	-35.714	21.0690	-25.000	(-72.00, -16.00)
		Day 42	8	-27.250	40.5806	-33.500	(-86.00, 41.00)

Source Listing: 10.1, 10.2, 10.3, 10.4

Table 11.1
Summary of Laboratory Data - Quantitative

Safety Population (N = 8)

			I-131-CLR1404				
Laboratory Type	Parameter	Visit	N	Mean	Std Dev	Median	Range
Urinalysis	Epithelial cells (/hpf)	Screening	5	0.400	0.5477	0.000	(0.00, 1.00)
		Baseline	4	0.500	0.5774	0.500	(0.00, 1.00)
		Day 6	6	0.667	1.2111	0.000	(0.00, 3.00)
		Day 14	6	0.667	1.6330	0.000	(0.00, 4.00)
		Day 30	5	0.200	0.4472	0.000	(0.00, 1.00)
		Day 42	4	0.250	0.5000	0.000	(0.00, 1.00)
	Epithelial cells (/hpf) Change from Baseline	Day 6	5	0.200	1.0954	0.000	(-1.00, 2.00)
		Day 14	5	-0.400	0.5477	0.000	(-1.00, 0.00)
		Day 30	5	-0.200	0.8367	0.000	(-1.00, 1.00)
		Day 42	4	-0.250	0.5000	0.000	(-1.00, 0.00)
	White Blood Cells (/UL)	Screening	6	3.000	3.2249	2.000	(0.00, 9.00)
		Baseline	4	3.250	4.0311	2.000	(0.00, 9.00)
		Day 6	6	3.167	2.9944	2.500	(1.00, 9.00)
		Day 14	6	1.000	0.8944	1.000	(0.00, 2.00)
		Day 30	5	1.800	1.9235	1.000	(0.00, 5.00)
		Day 42	4	3.500	4.3589	1.500	(1.00, 10.00)
	white blood cells (/UL) Change from Baseline	Day 6	6	-0.667	2.6583	0.000	(-6.00, 1.00)
		Day 14	6	-2.833	4.1191	-1.000	(-9.00, 1.00)
		Day 30	5	-1.000	1.7321	0.000	(-4.00, 0.00)
		Day 42	4	0.250	0.9574	0.500	(-1.00, 1.00)

Source Listing: 10.1, 10.2, 10.3, 10.4

Table 11.1
Summary of Laboratory Data - Quantitative

Safety Population (N = 8)

Laboratory Type	Parameter	Visit	I-131-CLR1404 N	Mean	Std Dev	Median	Range
Urinalysis	Red Blood Cells (/UL)	Screening	6	4.000	2.6833	4.500	(1.00, 8.00)
		Baseline	4	6.750	10.3078	2.500	(0.00, 22.00)
		Day 6	6	6.167	5.1929	5.000	(1.00, 15.00)
		Day 14	6	2.500	3.3317	1.000	(0.00, 9.00)
		Day 30	6	3.833	2.6394	4.000	(0.00, 7.00)
		Day 42	4	3.750	1.8930	4.500	(1.00, 5.00)
	Red Blood Cells (/UL) Change from Baseline	Day 6	6	0.667	4.5019	2.000	(-7.00, 5.00)
		Day 14	6	-3.000	10.1193	-0.500	(-22.00, 8.00)
		Day 30	6	-1.667	7.0616	1.000	(-16.00, 2.00)
		Day 42	4	-4.000	8.7178	0.000	(-17.00, 1.00)
	Casts (/lpf)	Screening	3	4.667	5.6862	3.000	(0.00, 11.00)
		Baseline	3	0.333	0.5774	0.000	(0.00, 1.00)
		Day 6	3	3.000	2.6458	4.000	(0.00, 5.00)
		Day 14	3	4.333	5.8595	2.000	(0.00, 11.00)
		Day 30	3	0.000	0.0000	0.000	(0.00, 0.00)
		Day 42	2	1.500	2.1213	1.500	(0.00, 3.00)
	Casts (/lpf) Change from Baseline	Day 6	3	2.667	3.2146	4.000	(-1.00, 5.00)
		Day 14	3	4.000	6.2450	2.000	(-1.00, 11.00)
		Day 30	3	-0.333	0.5774	0.000	(-1.00, 0.00)
		Day 42	2	1.000	2.8284	1.000	(-1.00, 3.00)

Source Listing: 10.1, 10.2, 10.3, 10.4

Table 11.1
Summary of Laboratory Data - Quantitative

Safety Population (N = 8)

Laboratory Type	Parameter	Visit	I-131-CLR1404 N	Mean	Std Dev	Median	Range
Urinalysis	Crystals (/lpf)	Screening	2	0.000	0.0000	0.000	(0.00, 0.00)
		Baseline	1	0.000	0.0000	0.000	(0.00, 0.00)
		Day 6	1	0.000	0.0000	0.000	(0.00, 0.00)
		Day 14	1	0.000	0.0000	0.000	(0.00, 0.00)
		Day 30	1	0.000	0.0000	0.000	(0.00, 0.00)
		Day 42	1	0.000	0.0000	0.000	(0.00, 0.00)
	Crystals (/lpf) Change from Baseline	Day 6	1	0.000	0.0000	0.000	(0.00, 0.00)
		Day 14	1	0.000	0.0000	0.000	(0.00, 0.00)
		Day 30	1	0.000	0.0000	0.000	(0.00, 0.00)
		Day 42	1	0.000	0.0000	0.000	(0.00, 0.00)
	pH	Screening	8	5.625	0.5825	5.750	(5.00, 6.50)
		Baseline	6	5.833	0.5164	6.000	(5.00, 6.50)
		Day 6	8	5.938	0.9425	6.000	(5.00, 8.00)
		Day 14	8	5.813	0.6512	6.000	(5.00, 7.00)
		Day 30	8	5.750	0.3780	6.000	(5.00, 6.00)
		Day 42	6	5.667	0.7528	5.500	(5.00, 7.00)
	pH Change from Baseline	Day 6	8	0.250	0.9258	0.000	(-1.00, 2.00)
		Day 14	8	0.125	0.5825	0.000	(-0.50, 1.00)
		Day 30	8	0.063	0.4955	0.000	(-0.50, 1.00)
		Day 42	6	0.000	0.7071	0.000	(-1.00, 1.00)

Source Listing: 10.1, 10.2, 10.3, 10.4

Table 11.1
Summary of Laboratory Data - Quantitative

Safety Population (N = 8)

			I-131-CLR1404				
Laboratory Type	Parameter	Visit	N	Mean	Std Dev	Median	Range
Urinalysis	Specific Gravity	Screening	8	1.020	0.0093	1.020	(1.01, 1.03)
		Baseline	6	1.018	0.0041	1.020	(1.01, 1.02)
		Day 6	8	1.020	0.0093	1.020	(1.01, 1.03)
		Day 14	7	1.016	0.0053	1.020	(1.01, 1.02)
		Day 30	8	1.019	0.0035	1.020	(1.01, 1.02)
		Day 42	6	1.020	0.0063	1.020	(1.01, 1.03)
	Specific Gravity Change from Baseline	Day 6	8	0.004	0.0119	0.000	(-0.01, 0.02)
		Day 14	7	-0.003	0.0095	-0.010	(-0.01, 0.01)
		Day 30	8	-0.001	0.0083	0.000	(-0.01, 0.01)
		Day 42	6	-0.002	0.0117	0.000	(-0.02, 0.01)
Lipids	Total Cholesterol (mg/dL)	Baseline	8	181.000	34.8056	198.000	(135.00, 214.00)
		Day 3	8	177.375	27.9742	185.500	(130.00, 203.00)
	Total Cholesterol (mg/dL) Change from Baseline	Day 3	8	-3.625	12.6935	-6.000	(-21.00, 22.00)
	HDL (mg/dL)	Baseline	8	43.625	17.4105	39.000	(25.00, 74.00)
		Day 3	8	43.500	16.7843	38.000	(25.00, 75.00)
	HDL (mg/dL) Change from Baseline	Day 3	8	-0.125	2.0310	-0.500	(-3.00, 3.00)

Source Listing: 10.1, 10.2, 10.3, 10.4

Table 11.1
Summary of Laboratory Data - Quantitative

Safety Population (N = 8)

Laboratory Type	Parameter	Visit	I-131-CLR1404 N	Mean	Std Dev	Median	Range
Lipids	LDL (mg/dL)	Baseline	6	112.667	28.4441	119.500	(76.00, 147.00)
		Day 3	6	109.167	24.8308	114.500	(72.00, 144.00)
	LDL (mg/dL) Change from Baseline	Day 3	6	-3.500	10.5404	-3.500	(-17.00, 15.00)
	Triglycerides (mg/dL)	Baseline	8	106.375	28.3697	106.000	(59.00, 143.00)
		Day 3	8	106.375	36.3473	98.500	(53.00, 162.00)
	Triglycerides (mg/dL) Change from Baseline	Day 3	8	0.000	21.0034	1.000	(-43.00, 25.00)

Source Listing: 10.1, 10.2, 10.3, 10.4

Table 11.2
Summary of Laboratory Data - Qualitative

Safety Population (N = 8)

			I-131-CLR1404 N (%)				
Laboratory Type	Parameter	Visit	None	Trace	+	++	+++
Urinalysis	Protien	Screening	6 (75.0%)	0 (0.0%)	1 (12.5%)	0 (0.0%)	0 (0.0%)
		Baseline	5 (62.5%)	0 (0.0%)	0 (0.0%)	0 (0.0%)	0 (0.0%)
		Day 6	5 (62.5%)	2 (25.0%)	0 (0.0%)	0 (0.0%)	0 (0.0%)
		Day 14	8 (100.0%)	0 (0.0%)	0 (0.0%)	0 (0.0%)	0 (0.0%)
		Day 30	6 (75.0%)	2 (25.0%)	0 (0.0%)	0 (0.0%)	0 (0.0%)
		Day 42	5 (62.5%)	1 (12.5%)	0 (0.0%)	0 (0.0%)	0 (0.0%)
	Glucose	Screening	8 (100.0%)	0 (0.0%)	0 (0.0%)	0 (0.0%)	0 (0.0%)
		Baseline	6 (75.0%)	0 (0.0%)	0 (0.0%)	0 (0.0%)	0 (0.0%)
		Day 6	8 (100.0%)	0 (0.0%)	0 (0.0%)	0 (0.0%)	0 (0.0%)
		Day 14	8 (100.0%)	0 (0.0%)	0 (0.0%)	0 (0.0%)	0 (0.0%)
		Day 30	7 (87.5%)	1 (12.5%)	0 (0.0%)	0 (0.0%)	0 (0.0%)
		Day 42	6 (75.0%)	0 (0.0%)	0 (0.0%)	0 (0.0%)	0 (0.0%)
	Hemoglobin	Screening	8 (100.0%)	0 (0.0%)	0 (0.0%)	0 (0.0%)	0 (0.0%)
		Baseline	6 (75.0%)	0 (0.0%)	0 (0.0%)	0 (0.0%)	0 (0.0%)
		Day 6	7 (87.5%)	1 (12.5%)	0 (0.0%)	0 (0.0%)	0 (0.0%)
		Day 14	7 (87.5%)	0 (0.0%)	0 (0.0%)	0 (0.0%)	0 (0.0%)
		Day 30	8 (100.0%)	0 (0.0%)	0 (0.0%)	0 (0.0%)	0 (0.0%)
		Day 42	5 (62.5%)	1 (12.5%)	0 (0.0%)	0 (0.0%)	0 (0.0%)

Source Listing: 10.3

Table 11.2
Summary of Laboratory Data - Qualitative

Safety Population (N = 8)

			I-131-CLR1404 N (%)				
Laboratory Type	Parameter	Visit	None	Trace	+	++	+++
Urinalysis	Ketones	Screening	7 (87.5%)	1 (12.5%)	0 (0.0%)	0 (0.0%)	0 (0.0%)
		Baseline	6 (75.0%)	0 (0.0%)	0 (0.0%)	0 (0.0%)	0 (0.0%)
		Day 6	7 (87.5%)	0 (0.0%)	0 (0.0%)	0 (0.0%)	0 (0.0%)
		Day 14	8 (100.0%)	0 (0.0%)	0 (0.0%)	0 (0.0%)	0 (0.0%)
		Day 30	8 (100.0%)	0 (0.0%)	0 (0.0%)	0 (0.0%)	0 (0.0%)
		Day 42	6 (75.0%)	0 (0.0%)	0 (0.0%)	0 (0.0%)	0 (0.0%)
	Bilirubin	Screening	5 (62.5%)	0 (0.0%)	0 (0.0%)	0 (0.0%)	0 (0.0%)
		Baseline	5 (62.5%)	0 (0.0%)	0 (0.0%)	0 (0.0%)	0 (0.0%)
		Day 6	5 (62.5%)	0 (0.0%)	0 (0.0%)	0 (0.0%)	0 (0.0%)
		Day 14	5 (62.5%)	0 (0.0%)	0 (0.0%)	0 (0.0%)	0 (0.0%)
		Day 30	5 (62.5%)	0 (0.0%)	0 (0.0%)	0 (0.0%)	0 (0.0%)
		Day 42	5 (62.5%)	0 (0.0%)	0 (0.0%)	0 (0.0%)	0 (0.0%)
	Casts	Screening	5 (62.5%)	0 (0.0%)	0 (0.0%)	0 (0.0%)	0 (0.0%)
		Baseline	3 (37.5%)	0 (0.0%)	0 (0.0%)	0 (0.0%)	0 (0.0%)
		Day 6	5 (62.5%)	0 (0.0%)	0 (0.0%)	0 (0.0%)	0 (0.0%)
		Day 14	5 (62.5%)	0 (0.0%)	0 (0.0%)	0 (0.0%)	0 (0.0%)
		Day 30	5 (62.5%)	0 (0.0%)	0 (0.0%)	0 (0.0%)	0 (0.0%)
		Day 42	3 (37.5%)	0 (0.0%)	0 (0.0%)	0 (0.0%)	0 (0.0%)

Source Listing: 10.3

Table 11.2
Summary of Laboratory Data - Qualitative

Safety Population (N = 8)

Laboratory Type	Parameter	Visit	I-131-CLR1404 N (%) None	Amorphous Urates	Many Amorphous	Few Squamous	Rare Squamous
Urinalysis	Crystals	Screening	4 (50.0%)	1 (12.5%)	0 (0.0%)	0 (0.0%)	0 (0.0%)
		Baseline	5 (62.5%)	0 (0.0%)	0 (0.0%)	0 (0.0%)	0 (0.0%)
		Day 6	7 (87.5%)	0 (0.0%)	0 (0.0%)	0 (0.0%)	0 (0.0%)
		Day 14	7 (87.5%)	0 (0.0%)	0 (0.0%)	0 (0.0%)	0 (0.0%)
		Day 30	6 (75.0%)	0 (0.0%)	1 (12.5%)	0 (0.0%)	0 (0.0%)
		Day 42	5 (62.5%)	0 (0.0%)	0 (0.0%)	0 (0.0%)	0 (0.0%)
	Epithelial Cells	Screening	1 (12.5%)	0 (0.0%)	0 (0.0%)	0 (0.0%)	1 (12.5%)
		Baseline	1 (12.5%)	0 (0.0%)	0 (0.0%)	0 (0.0%)	1 (12.5%)
		Day 6	1 (12.5%)	0 (0.0%)	0 (0.0%)	1 (12.5%)	0 (0.0%)
		Day 14	1 (12.5%)	0 (0.0%)	0 (0.0%)	0 (0.0%)	1 (12.5%)
		Day 30	2 (25.0%)	0 (0.0%)	0 (0.0%)	0 (0.0%)	0 (0.0%)
		Day 42	1 (12.5%)	0 (0.0%)	0 (0.0%)	0 (0.0%)	1 (12.5%)

Source Listing: 10.3

Table 12
Summary of Concomitant Medications

Safety Population (N= 8)

Preferred Term	I-131-CLR1404 N (%)	Medications
NUMBER OF PATIENTS WITH CONCOMITANT MEDICATIONS	8 (100.0%)	61
POTASSIUM IODIDE	8 (100.0%)	8
ACETYLSALICYLIC ACID	3 (37.5%)	3
DOCUSATE SODIUM	3 (37.5%)	3
CLONALIN	2 (25.0%)	3
CALCIUM	2 (25.0%)	2
LISINOPRIL	2 (25.0%)	2
MULTIVITAMINS	2 (25.0%)	2
SIMVASTATIN	2 (25.0%)	2
LOMOTIL /00034001/	1 (12.5%)	2
ALFUZOSIN HYDROCHLORIDE	1 (12.5%)	1
BACITRACIN	1 (12.5%)	1
CALCIUM WITH VITAMIN D /01233101/	1 (12.5%)	1
CEFALEXIN	1 (12.5%)	1
CENTRUM SILVER /06027401/	1 (12.5%)	1
COLECALCIFEROL	1 (12.5%)	1
COTYLENOL	1 (12.5%)	1
DEXTROMETHORPHAN HYDROBROMIDE	1 (12.5%)	1
DIPHENHYDRAMINE	1 (12.5%)	1
DUTASTERIDE	1 (12.5%)	1
ENALAPRIL MALEATE	1 (12.5%)	1
ENOXAPARIN SODIUM	1 (12.5%)	1
ESCITALOPRAM OXALATE	1 (12.5%)	1
FISH OIL	1 (12.5%)	1

Source Listing: 11
Note: All percentages are determined from a denominator of all patients in the safety population.
If a patient has more than one medication with a given preferred term, it is counted for each medication in the count of medications for that preferred term, and counted once for that patient in the count of patients for that preferred term.

Table 12
Summary of Concomitant Medications

Safety Population (N= 8)

Preferred Term	I-131-CLR1404 N (%)	Medications
GABAPENTIN	1 (12.5%)	1
HYDROCORTISONE	1 (12.5%)	1
IBUPROFEN	1 (12.5%)	1
INFLUENZA VACCINE	1 (12.5%)	1
LEVOTHYROXINE SODIUM	1 (12.5%)	1
LORAZEPAM	1 (12.5%)	1
LOVASTATIN	1 (12.5%)	1
METFORMIN	1 (12.5%)	1
METOPROLOL SUCCINATE	1 (12.5%)	1
MORPHINE SULFATE	1 (12.5%)	1
OMEPRAZOLE	1 (12.5%)	1
OXYCOCET	1 (12.5%)	1
PREDNISONE	1 (12.5%)	1
PSEUDOEPHEDRINE	1 (12.5%)	1
RANIBIZUMAB	1 (12.5%)	1
RANITIDINE	1 (12.5%)	1
TAMSULOSIN HYDROCHLORIDE	1 (12.5%)	1
VICODIN	1 (12.5%)	1
VITAMIN E /01552201/	1 (12.5%)	1
ZOLEDRONIC ACID	1 (12.5%)	1

Source Listing: 11
Note: All percentages are determined from a denominator of all patients in the safety population.
If a patient has more than one medication with a given preferred term, it is counted for each medication in the count of medications for that preferred term, and counted once for that patient in the count of patients for that preferred term.

Table 13
Summary of Vital Signs

Safety Population (N = 8)

		I-131-CLR1404				
Parameter	Visit	N	Mean	Std Dev	Median	Range
Supine Systolic Blood Pressure (mmHg)	Screening	8	130.3	13.54	132.5	(109, 148)
	Baseline	8	124.3	12.28	121.0	(110, 141)
	5 Minutes	8	126.8	12.38	123.5	(116, 156)
	15 Minutes	8	125.4	17.37	124.0	(104, 157)
	30 Minutes	5	131.6	12.22	130.0	(117, 148)
	60 Minutes	8	130.8	9.33	126.5	(120, 147)
	4 - 6 Hours	8	121.3	11.16	123.5	(107, 140)
	Day 1	7	129.6	9.07	134.0	(117, 141)
	Day 2	8	125.9	6.90	125.5	(117, 139)
	Day 3	8	121.4	17.25	121.5	(101, 153)
	Day 6	8	124.1	9.76	125.0	(107, 138)
	Day 14	8	123.9	17.59	120.0	(95, 147)
	Day 30	8	129.1	18.92	132.5	(102, 150)
	Day 42	8	131.4	14.99	127.0	(110, 156)
Supine Systolic Blood Pressure (mmHg) Change from Baseline	5 Minutes	8	2.5	12.46	5.5	(-15, 16)
	15 Minutes	8	1.1	17.04	8.0	(-30, 17)
	30 Minutes	5	9.8	7.43	7.0	(5, 23)
	60 Minutes	8	6.5	14.36	4.0	(-12, 31)
	4 - 6 Hours	8	-3.0	7.91	-1.5	(-14, 8)
	Day 1	7	3.6	12.58	3.0	(-18, 18)
	Day 2	8	1.6	10.32	4.5	(-16, 13)
	Day 3	8	-2.9	10.23	-5.5	(-14, 16)
	Day 6	8	-0.1	10.60	2.5	(-20, 12)
	Day 14	8	-0.4	15.03	0.5	(-21, 28)
	Day 30	8	4.9	21.05	5.0	(-34, 36)
	Day 42	8	7.1	8.69	10.5	(-7, 15)

Source Listing: 12

Table 13
Summary of Vital Signs

Safety Population (N = 8)

Parameter	Visit	I-131-CLR1404 N	Mean	Std Dev	Median	Range
Supine Diastolic Blood Pressure (mmHg)	Screening	8	77.3	5.44	77.0	(70, 84)
	Baseline	8	71.3	7.23	71.0	(60, 83)
	5 Minutes	8	76.0	9.32	78.0	(60, 87)
	15 Minutes	8	75.4	9.97	76.0	(60, 88)
	30 Minutes	5	79.6	2.79	81.0	(75, 82)
	60 Minutes	8	77.4	8.16	79.0	(68, 92)
	4 - 6 Hours	8	72.1	11.66	70.5	(56, 89)
	Day 1	7	78.6	7.00	81.0	(67, 86)
	Day 2	8	74.3	5.18	72.5	(70, 86)
	Day 3	8	72.4	8.78	76.5	(55, 81)
	Day 6	8	75.4	9.04	74.0	(64, 88)
	Day 14	8	74.3	7.50	75.0	(64, 88)
	Day 30	8	73.9	9.48	75.0	(59, 89)
	Day 42	8	77.8	6.14	76.5	(70, 87)
Supine Diastolic Blood Pressure (mmHg) Change from Baseline	5 Minutes	8	4.8	4.71	5.5	(-2, 11)
	15 Minutes	8	4.1	10.53	2.5	(-15, 18)
	30 Minutes	5	4.6	7.06	6.0	(-2, 15)
	60 Minutes	8	6.1	7.04	7.5	(-9, 13)
	4 - 6 Hours	8	0.9	8.29	-3.0	(-6, 16)
	Day 1	7	5.7	7.78	7.0	(-10, 12)
	Day 2	8	3.0	5.81	3.5	(-6, 11)
	Day 3	8	1.1	7.14	1.0	(-10, 12)
	Day 6	8	4.1	9.63	6.5	(-11, 15)
	Day 14	8	3.0	10.53	2.5	(-10, 21)
	Day 30	8	2.6	9.52	4.0	(-11, 15)
	Day 42	8	6.5	2.73	6.0	(3, 10)

Source Listing: 12

Table 13
Summary of Vital Signs

Safety Population (N = 8)

Parameter	Visit	I-131-CLR1404 N	Mean	Std Dev	Median	Range
Pulse (bpm)	Screening	8	72.1	15.91	68.5	(56, 107)
	Baseline	8	75.6	12.65	74.5	(59, 101)
	5 Minutes	8	71.6	11.55	71.0	(58, 91)
	15 Minutes	8	73.1	15.45	71.5	(52, 101)
	30 Minutes	5	75.0	18.77	71.0	(53, 102)
	60 Minutes	8	69.1	14.11	69.5	(52, 96)
	4 - 6 Hours	8	79.1	11.68	78.0	(67, 104)
	Day 1	7	75.4	14.42	76.0	(55, 96)
	Day 2	8	78.8	17.61	74.0	(57, 116)
	Day 3	8	76.5	9.62	75.0	(65, 91)
	Day 6	8	75.3	14.16	75.5	(52, 94)
	Day 14	8	74.4	14.67	68.5	(61, 101)
	Day 30	8	79.0	12.60	81.5	(61, 100)
	Day 42	8	79.4	16.87	80.5	(54, 108)
Pulse (bpm) Change from Baseline	5 Minutes	8	-4.0	7.19	-5.0	(-15, 6)
	15 Minutes	8	-2.5	9.55	-2.0	(-21, 11)
	30 Minutes	5	-3.8	10.96	1.0	(-20, 6)
	60 Minutes	8	-6.5	6.26	-5.5	(-18, 4)
	4 - 6 Hours	8	3.5	4.93	4.0	(-4, 10)
	Day 1	7	-1.0	9.61	1.0	(-19, 10)
	Day 2	8	3.1	7.34	2.5	(-8, 15)
	Day 3	8	0.9	7.75	2.0	(-12, 11)
	Day 6	8	-0.4	9.32	0.5	(-16, 12)
	Day 14	8	-1.3	12.50	-4.5	(-20, 21)
	Day 30	8	3.4	11.06	5.0	(-14, 20)
	Day 42	8	3.8	13.64	-0.5	(-15, 28)

Source Listing: 12

Table 13
Summary of Vital Signs

Safety Population (N = 8)

		I-131-CLR1404				
Parameter	Visit	N	Mean	Std Dev	Median	Range
Temperature (oC)	Screening	8	36.61	0.236	36.65	(36.1, 36.8)
	Baseline	8	36.58	0.544	36.70	(35.7, 37.3)
	5 Minutes	8	36.75	0.278	36.70	(36.4, 37.3)
	15 Minutes	8	36.65	0.407	36.65	(36.1, 37.5)
	30 Minutes	5	36.46	0.152	36.50	(36.3, 36.6)
	60 Minutes	8	36.65	0.307	36.60	(36.3, 37.3)
	4 - 6 Hours	7	36.51	0.398	36.50	(35.9, 37.2)
	Day 1	7	36.57	0.525	36.30	(35.9, 37.4)
	Day 2	8	36.51	0.436	36.60	(35.8, 37.2)
	Day 3	8	36.34	0.338	36.45	(35.8, 36.7)
	Day 6	8	36.69	0.318	36.65	(36.3, 37.3)
	Day 14	7	36.57	0.395	36.70	(35.7, 36.8)
	Day 30	8	36.34	0.518	36.45	(35.6, 37.0)
	Day 42	8	36.34	0.661	36.60	(35.2, 37.0)
Temperature (oC) Change from Baseline	5 Minutes	8	0.18	0.471	0.05	(-0.4, 1.0)
	15 Minutes	8	0.08	0.446	0.05	(-0.5, 0.6)
	30 Minutes	5	0.14	0.434	0.00	(-0.3, 0.6)
	60 Minutes	8	0.08	0.456	0.05	(-0.5, 0.8)
	4 - 6 Hours	7	-0.06	0.399	-0.20	(-0.4, 0.6)
	Day 1	7	0.00	0.408	-0.10	(-0.5, 0.6)
	Day 2	8	-0.06	0.560	-0.15	(-0.8, 0.8)
	Day 3	8	-0.24	0.469	-0.40	(-0.7, 0.5)
	Day 6	8	0.11	0.344	0.00	(-0.2, 0.8)
	Day 14	7	-0.13	0.585	-0.20	(-0.9, 0.7)
	Day 30	8	-0.24	0.521	-0.30	(-1.0, 0.8)
	Day 42	8	-0.24	0.496	-0.30	(-0.9, 0.8)

Source Listing: 12

Table 13
Summary of Vital Signs

Safety Population (N = 8)

		I-131-CLR1404				
Parameter	Visit	N	Mean	Std Dev	Median	Range
Respiration Rate (bpm)	Screening	8	17.8	2.05	17.5	(15, 20)
	Baseline	8	19.5	2.98	20.0	(14, 24)
	5 Minutes	7	19.4	2.76	20.0	(16, 24)
	15 Minutes	7	18.4	3.15	18.0	(15, 24)
	30 Minutes	5	19.4	5.18	16.0	(15, 26)
	60 Minutes	8	18.3	2.71	19.0	(14, 22)
	4 - 6 Hours	8	18.3	3.11	20.0	(14, 22)
	Day 1	6	18.2	2.23	19.0	(15, 20)
	Day 2	8	18.5	4.11	20.0	(12, 24)
	Day 3	7	18.9	2.73	18.0	(16, 24)
	Day 6	8	18.5	2.00	18.0	(16, 22)
	Day 14	8	18.6	1.92	18.0	(16, 22)
	Day 30	8	19.4	2.45	20.0	(16, 24)
	Day 42	8	18.5	1.69	18.0	(17, 22)
Respiration Rate (bpm) Change from Baseline	5 Minutes	7	0.6	2.23	0.0	(-2, 4)
	15 Minutes	7	-0.4	1.99	0.0	(-3, 2)
	30 Minutes	5	0.2	2.05	1.0	(-2, 2)
	60 Minutes	8	-1.3	1.83	-2.0	(-4, 2)
	4 - 6 Hours	8	-1.3	2.12	-1.0	(-4, 2)
	Day 1	6	-1.2	2.86	-1.5	(-4, 2)
	Day 2	8	-1.0	3.85	-1.0	(-8, 4)
	Day 3	7	-0.3	2.21	0.0	(-3, 3)
	Day 6	8	-1.0	2.45	-2.0	(-4, 3)
	Day 14	8	-0.9	2.23	-2.0	(-3, 4)
	Day 30	8	-0.1	2.75	0.0	(-4, 4)
	Day 42	8	-1.0	2.88	-1.0	(-6, 3)

Source Listing: 12

Table 13
Summary of Vital Signs

Safety Population (N = 8)

Parameter	Visit	I-131-CLR1404 N	Mean	Std Dev	Median	Range
Saturated Oxygen (%)	Screening	7	96.9	1.46	97.0	(95, 99)
	Baseline	8	96.4	1.60	96.5	(94, 98)
	5 Minutes	8	96.5	2.07	97.0	(92, 98)
	15 Minutes	8	96.3	2.31	96.5	(93, 99)
	30 Minutes	5	96.2	1.79	97.0	(93, 97)
	60 Minutes	8	97.1	1.46	97.0	(95, 100)
	4 - 6 Hours	8	97.1	1.46	97.0	(95, 100)
	Day 1	7	97.6	1.72	98.0	(95, 100)
	Day 2	8	96.9	1.64	97.0	(95, 100)
	Day 3	7	96.6	0.98	97.0	(95, 98)
	Day 6	8	97.0	1.93	96.5	(94, 100)
	Day 14	7	97.0	1.29	97.0	(95, 99)
	Day 30	6	97.3	1.86	97.0	(95, 100)
	Day 42	6	97.2	0.75	97.0	(96, 98)
Saturated Oxygen (%) Change from Baseline	5 Minutes	8	0.1	1.46	0.0	(-2, 2)
	15 Minutes	8	-0.1	2.17	-0.5	(-3, 4)
	30 Minutes	5	-0.8	1.79	-1.0	(-3, 2)
	60 Minutes	8	0.8	1.39	1.0	(-1, 2)
	4 - 6 Hours	8	0.8	1.16	1.0	(-1, 2)
	Day 1	7	1.0	1.29	1.0	(-1, 3)
	Day 2	8	0.5	1.31	0.0	(-1, 2)
	Day 3	7	0.0	1.15	0.0	(-1, 2)
	Day 6	8	0.6	2.00	0.5	(-2, 4)
	Day 14	7	0.4	1.81	0.0	(-1, 4)
	Day 30	6	0.5	1.76	0.0	(-1, 3)
	Day 42	6	0.3	1.86	0.0	(-2, 3)

Source Listing: 12

Table 13
Summary of Vital Signs

Safety Population (N = 8)

		I-131-CLR1404				
Parameter	Visit	N	Mean	Std Dev	Median	Range
Weight (kg)	Screening	8	92.80	27.456	83.95	(61.1, 143.7)
	Baseline	6	86.25	21.324	84.30	(61.2, 122.5)
	Day 14	7	100.04	36.880	85.80	(61.9, 172.2)
Weight (kg) Change from Baseline	Day 14	7	4.14	10.772	0.50	(-1.2, 28.5)

Source Listing: 12
