# Supplementary material for: A Phase 1 Study of 131I-CLR1404 in Patients with Relapsed or Refractory Advanced Solid Tumors: Dosimetry, Biodistribution, Pharmacokinetics, and Safety
Source: PLoS One. 2014 Nov 17;9(11):e111652. doi: 10.1371/journal.pone.0111652 (PMC4234270; doi:10.1371/journal.pone.0111652)
Supplement: File S3 — Plasma Concentration DCL-08-001.pdf. (PDF) [file pone.0111652.s005.pdf]

Table A.1: Plasma Concentrations (ng/mL) of CLR1404 following a Single Dose Infusion of 10 mCi of I-131-CLR1404

Dose and Route	10 mCi I-131-CLR1404 Single Dose Administration via Intravenous Infusion												
Subject	Time After Infusion (hr)												
Number	Predose	0.0833	0.25	0.5	1	4 to 6	18 to 24	48	72	144	336	720	1008
001-101	0	36.2	66.9	63.7	64.8	44.0	15.0	25.3	27.1	26.7	19.6	NS	9.42
001-102	0	54.6	53.7	50.5	48.7	29.1	19.1	17.8	15.5	14.2	11.7	7.94	5.64
001-103	0	91.3	80.1	77.1	65.4	52.2	32.5	28.4	23.0	22.4	19.6	12.8	10.7
003-201	0	73.5	63.0	59.3	50.1	32.1	24.2	24.0	20.0	19.3	13.5	11.2	9.13
005-301	0	63.5	58.9	NS	55.0	36.4	20.0	17.9	14.1	14.7	12.4	8.88	7.03
005-302	0	70.1	69.0	NS	55.0	44.2	24.8	23.4	21.5	20.1	17.8	13.0	10.6
007-401	0	84.0	72.4	70.2	62.0	47.0	31.4	26.4	24.1	22.2	19.4	16.1	10.6
007-402	0	73.8	63.9	63.0	67.6	48.1	28.9	23.5	23.6	20.8	16.8	13.5	10.4
N	8	8	8	6	8	8	8	8	8	8	8	7	8
Mean	0	68.4	66.0	64.0	58.6	41.6	24.5	23.3	21.1	20.1	16.4	11.9	9.19
SD	0	17.2	8.1	9.1	7.3	8.2	6.2	3.8	4.4	4.1	3.3	2.8	1.89
Min	0	36.2	53.7	50.5	48.7	29.1	15.0	17.8	14.1	14.2	11.7	7.94	5.64
Median	0	71.8	65.4	63.4	58.5	44.1	24.5	23.8	22.3	20.5	17.3	12.8	9.91
Max	0	91.3	80.1	77.1	67.6	52.2	32.5	28.4	27.1	26.7	19.6	16.1	10.7
CV%	NA	25	12	14	12	20	25	16	21	21	20	24	21

NS No sample.
NA Not applicable.
Note: Nominal timepoints are presented.
