# Supplementary material for: A Phase 1 Study of 131I-CLR1404 in Patients with Relapsed or Refractory Advanced Solid Tumors: Dosimetry, Biodistribution, Pharmacokinetics, and Safety
Source: PLoS One. 2014 Nov 17;9(11):e111652. doi: 10.1371/journal.pone.0111652 (PMC4234270; doi:10.1371/journal.pone.0111652)
Supplement: Protocol S1 — Trial Protocol. (PDF) [file pone.0111652.s006.pdf]

CELLECTAR

Protocol No. DCL-08-001

A Phase 1, Multi-Center, Open-Label, Dosimetry Study of I-131-CLR1404 in Patients with Relapsed or Refractory Advanced Solid Tumors who have Failed Standard Therapy or for Whom no Standard Therapy Exists

Final
Amended 10 September 2009
Version 03

Cellectar, Inc.
3301 Agriculture Drive
Madison, WI 53716

Confidentiality Statement
This document and the information it contains is confidential and the proprietary property of Cellectar. The information is not to be disclosed or transmitted to any party without the written approval of Cellectar or its agents, and any such unauthorized use or disclosure is expressly prohibited.

TABLE OF CONTENTS

1. SIGNATURE PAGE **4**
2. INTRODUCTION **6**
3. RATIONALE FOR THE STUDY **6**
4. STUDY OBJECTIVES **7**
4.1 Primary Objectives 7
4.2 Secondary Objectives 7
5. STUDY DESIGN **8**
5.1 Study Summary 8
5.2 Number of Clinical Sites 9
6. PATIENT SELECTION **9**
6.1 Number of Patients 9
6.2 Recruitment Methods 9
6.3 Inclusion Criteria 10
6.4 Exclusion Criteria 10
7. STUDY DRUG ADMINISTRATION **12**
7.1 Description of Study Drug 12
7.2 Study Drug Dosages 13
7.3 Treatment Duration 13
7.4 Patient Replacement 13
7.5 Study Drug Accountability 14
7.6 Concomitant Medications and Treatments 14
7.7 Study Endpoints 15
7.7.1 Primary Endpoints *15*
7.7.2 Secondary Endpoints *15*
8. TREATMENT PLAN **16**
8.1 Schedule of Procedures and Assessments 16
8.2 Study Evaluations 19
8.2.1 Screening Period *19*
8.2.2 Treatment Period *19*
8.2.3 Clinical Assessments *24*
9. ADVERSE EVENT REPORTING **27**
9.1 Definitions 27
9.1.1 Adverse Event *27*
9.1.2 Unexpected Adverse Event *28*
9.1.3 Serious Adverse Event *28*
9.2 Expedited Reporting of Adverse Events 29
9.3 Documenting Adverse Events 29
9.4 Grading and Relatedness of Adverse Events 30

9.4.1 Grading of Severity of an Adverse Event *30*
9.4.2 Relatedness to Study Drug *31*

10. PATIENT WITHDRAWAL 31

10.1 Criteria for Patient Withdrawal 31
10.2 Dose Limiting Criteria 32
10.3 Study Discontinuation 32
10.4 Reporting and Follow-Up Of Pregnancy 33

11. DATA ANALYSIS 33

11.1 Case Report Forms 33
11.2 Data Quality Assurance 33
11.3 Sample Size 33
11.4 Dosimetry Calculation 34
11.5 Statistical Analysis 34

12. ADMINISTRATIVE PROCEDURES 34

12.1 Patient Informed Consent 34
12.2 Ethical Conduct of the Study and IRB Approval 35
12.3 Protocol Amendments 35
12.4 Monitoring 36
12.5 Pre-Study Documentation 36
12.6 Confidentiality 36
12.7 Source Documents 37
12.8 Record Retention 37
12.9 Publication Policy 38

APPENDIX A: INVESTIGATOR AGREEMENT 39

APPENDIX B: DECLARATION OF HELSINKI 40

APPENDIX C: ECOG PERFORMANCE STATUS* 45

APPENDIX D: WHOLE BODY SCANNING AND DOSIMETRY 46

APPENDIX E: GLOSSARY OF ABBREVIATIONS 51

1. SIGNATURE PAGE

Title: **A Phase 1, Multi-Center, Open-Label, Dosimetry Study of I-131-CLR1404 in Patients with Relapsed or Refractory Advanced Solid Tumors who have Failed Standard Therapy or for Whom no Standard Therapy Exists**

Protocol Number: **DCL-08-001**

Date: **10 September 2009**

Sponsor's Name and Address:
Cellectar, Inc.
3301 Agriculture Drive
Madison, WI 53716
Phone: (608) 441-8120
Fax: (608) 441-8121

Signature Authority:
Ernest Allen
Director, Clinical Operations; Cellectar, Inc.
3301 Agriculture Drive
Madison, WI 53716
Phone: (608) 441-8120

Signature: [signature] Date: 16 Sept 2009

Medical Monitor:
Robert Shepard, MD, FACP
Consulting Medical Monitor
Cellectar, Inc.
3301 Agriculture Drive
Madison, WI 53716
Phone: (919) 271-3805

Signature: Robt Shepard, MD, F.A.C.P. Date: 23 Sept 2009

Medical Director:
William R. Clarke, MD, M.Sc.
CEO; Cellectar, Inc.
3301 Agriculture Drive
Madison, WI 53716
Phone: (608) 441-8120

Signature: [signature] Date: 21 Sept 2009

Quality Assurance:

Jill Irwin
Manager, Regulatory Affairs; Cellectar, Inc.
3301 Agriculture Drive
Madison, WI 53716
Phone: (608) 441-8120

Signature

21 Sep 2009
Date

2. INTRODUCTION

During 2008, it is estimated that there were over 1.4 million new cases of cancers in the United States and over 500,000 lives were claimed. Worldwide, cancer of all types accounts for 3.5 million deaths annually. Treatment of neoplastic diseases represents an unsatisfied medical need worldwide[1].

Tumor treatment with radioactive isotopes has been used as a fundamental cancer therapy for decades. Selective delivery of effective doses of radioactive isotopes that destroy tumor tissue, spare surrounding normal tissue, do not accumulate in and damage vital organs (especially bone marrow, liver and kidneys), and that can be rapidly eliminated from the body remains a goal of targeted radiotherapies. Cellectar has developed a novel radioiodinated therapy compound which takes advantage of the unique chemistry of phospholipid ethers (PLEs) and their analogs[2]. Cellectar's novel compound (CLR1404) is radiolabeled with the isotope iodine-131 (I-131).

Radiolabeled CLR1404 has been evaluated in both xenograft and spontaneous (transgenic) tumor models in mice and rats. Overall, these studies demonstrated specific uptake and retention in 40 + malignant tumor models. Anti-tumor activity has been demonstrated in nine tumor models (breast, prostate, lung, glioma, ovarian, renal, colorectal, pancreatic and melanoma) in mice, in which I-131-CLR1404 caused profound effects on tumor growth, thereby confirming radiolabeled CLR1404 as a potential anti-cancer therapeutic agent.

Toxicology and safety pharmacology studies in rodents and monkeys have demonstrated that non-radiolabeled ("cold") CLR1404 is well tolerated at doses up to 4.0 mg/kg, approximately 1000X the anticipated human dose (assuming 0.30 mg total dose injected into a 70 kg adult) with no significant adverse effects.

3. RATIONALE FOR THE STUDY

I-131-CLR1404 is a small-molecule, phospholipid ether analog that combines lipid-like properties with a cancer therapeutic beta-emitting radioisotope. Cellectar believes that this compound will benefit individuals with cancer due to its selective retention and accumulation in malignant cells versus nonmalignant cells. Nonclinical experiments have demonstrated that I-131-CLR1404 significantly slows malignant tumor growth in several mouse tumor models. Survival studies with I-131-CLR1404 were conducted in glioma, ovarian, renal, colorectal and pancreatic tumor models and had significant effect on overall survival. Non-radiolabeled CLR1404 has been demonstrated to be safe in rodents, non-rodent, and nonhuman primate species at presumed therapeutic doses up to approximately 1000x the anticipated human dose.

1. Ries LAG, Melbert D, Krapcho M, Mariotto A, Miller BA, Feuer EJ, Clegg L, Horner MJ, Howlader N, Eisner MP, Reichman M, Edwards BK (eds). SEER Cancer Statistics Review, 1975-2005, National Cancer Institute. Bethesda, MD, http://seer.cancer.gov/csr/1975_2005/, based on November 2007 SEER data submission, posted to the SEER web site, 2008.
2. Pinchuk AN, Rampy MA, Longino MA, Skinner RW, Gross MD, et al. Synthesis and structure – Activity relationship effects on the tumor avidity of radioiodinated phospholipid ether analogues. J Med Chem 2006;49:2155-2165.

Given this promising preclinical evaluation, Cellectar is planning to evaluate I-131-CLR1404 in the clinical setting in patients with advanced cancers.

The primary objectives of the study will be dosimetry calculations and the determination of the millicurie dose which is expected to deliver 35-40cGy of radiation to bone marrow. This dose will be used as the starting dose in a subsequent maximum tolerated dose (MTD) study. The secondary objectives of the study will be to determine the safety profile and determine the pharmacokinetic profile of I-131-CLR1404.

I-131-CLR1404 is supplied as a ready-for-use radiopharmaceutical for intravenous dosing that is provided as a clear, faint yellow to yellow, sterile solution consisting of I-131-CLR1404 (18-(*p*-[^{131}I]-iodophenyl)octadecyl phosphocholine) and CLR1401 (18-(*para*-iodophenyl)octadecyl phosphocholine) in sodium chloride injection USP, ethanol USP/NF, polysorbate 20 NF, and sodium ascorbate USP. The study drug is packaged in a single-use glass vial and must be withdrawn through a 25mm, 0.22 or 0.45 micron sterile filter prior to administration. **The drug as supplied must be diluted to 10 mL with normal saline solution (normal saline for injection) and thoroughly mixed prior to administration via slow IV infusion.**

I-131-CLR1404 will be administered intravenously as a single dose infusion.

4. STUDY OBJECTIVES

4.1 PRIMARY OBJECTIVES

The primary objectives of the study are:

- To calculate whole body and organ radiation dosimetry of I-131-CLR1404
- To determine the millicurie (mCi) dose which is expected to deliver 35-40 centigray (cGy) of radiation to bone marrow, which will serve as a starting dose in a subsequent MTD study

4.2 SECONDARY OBJECTIVES

The secondary objectives of the study are:

- To determine the safety of I-131-CLR1404
- To determine the pharmacokinetic profile of I-131-CLR1404

5. STUDY DESIGN

5.1 STUDY SUMMARY

This will be a multi-center study evaluating dosimetry and safety for up to nine patients with refractory or relapsed advanced solid tumors who have failed standard therapy or for whom no standard therapy exists. Patients who are eligible will be infused with a single dose of 10 millicuries (mCi) of I-131-CLR1404. All patients will be prescribed thyroid protection medication to be taken 24 hours prior to injection of the study drug and for 14 days afterwards.

Patients who withdraw from the study prematurely will be strongly encouraged to continue their thyroid protective medication for the full 14 days post infusion.

Whole body imaging for dosimetry calculations will be performed at 15-35 minutes post injection; 4-6, 18-24, 48, 72, and 144 hours (Day 6) post injection, and on Day 14 post injection. At the Investigator's discretion, optional SPECT imaging may be obtained between Day 3 and 14 to further characterize the patient's tumor(s). Blood and urine will be collected for clinical laboratory evaluation pre-dose and on days 6, 14, 30 and 42. Blood will be drawn for evaluation of lipids pre-dose and at 72 hours post injection. Blood will be collected for pharmacokinetic analysis prior to dosing, and at 5, 15, 30, and 60 minutes; 4-6, 18-24, 48, and 72 hours; 6, 14, 30, and 42 days post infusion. Blood will collected for radiologic biodistribution at pre-injection, 15 and 60 minutes, 4-6, 18-24, 48, and 72 hours; 6, 14, 30 and 42 days post dose. Patients will be asked to collect all of their urine for 14 days post injection in order to evaluate the urinary clearance of I-131-CLR1404. Patients will be monitored at each visit for adverse events and the use of concomitant medications.

Patients must have a refractory or relapsed advanced solid tumor and have a sufficient window of time to complete the washout period of 2 weeks if they have received standard therapy or 4 weeks if they have received experimental therapy, the dosimetry data acquisition (14 days), and the follow-up safety assessment period (4 weeks) prior to beginning another cycle of a therapeutic regimen. If, after 30 days on study the patient is not experiencing any drug-related toxicity, the patient can continue further treatment with a clinically appropriate non-investigational therapy, at the discretion of the Investigator.

Figure 5.1 Study Design

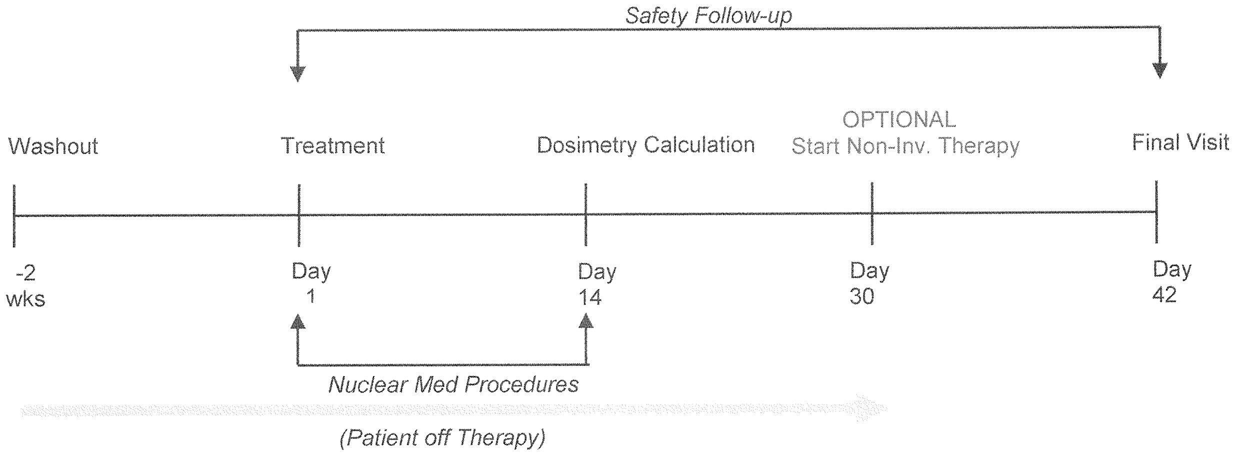

After all patients have completed the protocol, calculations will be performed using OLINDA-generated biodistribution curves to predict the injected dose of I-131-CLR1404 in mCi/m^2 that will deliver a radiation absorbed dose of 35-40cGy to total bone marrow. This OLINDA-calculated dose will be considered the starting dose for a subsequent Phase 1 maximum tolerated dose protocol.

5.2 NUMBER OF CLINICAL SITES

Approximately four clinical sites in the United States may participate in the study.

6. PATIENT SELECTION

6.1 NUMBER OF PATIENTS

Up to nine patients are expected to be enrolled.

6.2 RECRUITMENT METHODS

The methods used for recruitment of patients in the study will be devoid of any procedures that may be construed as coercive. The recruitment process will not involve any restrictions on sociodemographic factors including gender or ethnic characteristics of the patient population. However, the composition of the study patient population will depend on patient sources available to the clinical site.

Patients will be recruited through an existing database of oncology patients, clinic contacts, and referring physicians. Anticipated recruitment rate is approximately four patients per month.

6.3 INCLUSION CRITERIA

Patients may be enrolled into this protocol only if all of the following inclusion criteria are met:

- Patients must have relapsed or refractory advanced solid tumor and have exhausted standard treatment options or no standard therapy exists for the patient.
- Patients must have a sufficient window of time to complete the washout period of 2 weeks if patients have received standard therapy or 4 weeks if patients have received experimental therapy, dosimetry data acquisition,(14 days), and the follow up safety assessment period (4 weeks) prior to beginning another cycle of a therapeutic regimen.
- Patient is ambulatory with an ECOG performance status of 0 or 1 (Appendix C), and an estimated life expectancy of ≥ 4 months.
- Patient is 18 years or older
- Patient is judged by the Investigator to have the initiative and means to be compliant with the protocol and be within geographical proximity to make the required study visits.
- Patients or their legal representatives must have the ability to read, understand and provide written informed consent for the initiation of any study related procedures.
- Patients with brain metastasis are acceptable if the clinical condition has been stable for at least one month. For patients with brain metastasis or glioblastoma multiforme who require steroids, they must have been on a stable or tapering dose of corticosteroids for at least one month.
- Female patients of childbearing potential must have a negative serum pregnancy test within 24 hours of start of treatment.
- Patients must agree to use an effective method of contraception (e.g., oral contraceptives, double-barrier methods such as a condom and a diaphragm, intrauterine device, Norplant, Depo-Provera) during the study and for 90 days following the last dose of study medication.

6.4 EXCLUSION CRITERIA

Patients will be excluded from enrollment if any of the following apply:

- Patient or physician plans concomitant chemotherapy, therapeutic radiation treatment, and/or biological treatment for cancer including immunotherapy while on study. Of note, localized palliative radiation therapy for bone pain will be allowed during the study period if clinically indicated. Ongoing hormonal therapy may be continued.
- Patients who have had more than 25% of the total bone marrow irradiated.
- Patients with diffuse lung disease or interstitial spread of carcinoma.
- Patients in whom the bladder or rectum is within a prior radiation therapy field and a dose of greater than 45 Gy was administered.
- Patients with total therapeutic radiation dose in excess of 25 Gy to the kidney, 45 Gy to brain, 45 Gy to stomach, 18 Gy to lung, or 25 Gy to liver.
- Patients receiving prior total-body irradiation.

- Patients with extradural tumor in contact with the spinal cord, or tumor located where swelling in response to therapy may impinge upon the spinal cord.
- Prior radiation therapy or chemotherapy within 2 weeks of the start of the study. Of note, patients on biologics such as cetuximab (Erbitux®) are acceptable if the patient has completed a washout phase. (Washout is one half-life of the drug or 2 weeks, whichever is longest).
- Patient has another active medical condition(s) or organ disease(s) that may either compromise patient safety or interfere with the safety and/or outcome evaluation of the study drug.
- While this exclusion is not limited to the following abnormalities, if any of the following laboratory abnormalities are present, the patient should be excluded:
  - WBC < 3000/µL
  - Absolute neutrophil count < 1500/µL
  - Platelets < 150,000/µL
  - Hemoglobin ≤ 11.0 gm/dL
  - Total bilirubin > 1.5 x upper limit of normal for age
  - SGOT or SGPT > 3 x upper limit of normal for age if no liver metastases or >5 x upper limit of normal for age in the presence of liver metastases
  - Serum creatinine > 1.5 x upper limit of normal for age
  - INR ≥ 2.0
- Patient has been treated with an investigational drug, investigational biologic, or investigational therapeutic device within 30 days of initiating study treatment.
- Patients who have received severely marrow toxic drugs (e.g. nitrosoureas, mitomycin).
- Patients who have received prior treatment with Iodine-131 in the past five years.
- Patients receiving concurrent hemodialysis.
- Patients receiving blood transfusions within 60 days of study start.
- Patients receiving hematopoietic growth factor therapy within 60 days of study start.
- Patients receiving prior stem cell transplantation.
- Patients with clinically evident ascites or with peritoneal carcinomatosis.
- Patient has clinically significant cardiac co-morbidities including: congestive heart failure (New York Heart Association class III-IV heart disease), a LVEF< 40%, unstable angina pectoris, serious cardiac arrhythmia requiring medication or a pacemaker, myocardial infarction within the past six months.
- Patient has clinically significant pulmonary impairment defined as an S_aO_2 on room air of 93% or less.
- Concurrent or recent (within 1 month) use of thrombolytic agents, or full-dose anticoagulants (except to maintain patency of preexisting, permanent indwelling IV catheters). Of note, therapy with low-molecular weight heparin is acceptable as long as the INR < 2.0.

- Patients with uncontrolled hypertension as defined by SBP > 150 mm/Hg, DBP > 100 mm/Hg or patients with uncontrolled diabetes that would compromise patient safety or interfere with the safety and/or outcome evaluation of the study drug.
- Grade II-IV peripheral vascular disease or has had peripheral vascular surgery within the past year
- Less than 4 weeks since prior major surgery
- Known positive for HIV, Hepatitis C (active, previously treated or both), or is Hepatitis B core antigen positive
- Concurrent chronic use of aspirin (325 mg/day or more)
- Patient is pregnant or lactating.
- Patients with colostomy/ileostomy.
- Patients with poor venous access who will be unable to receive study drug into a peripheral venous catheter
- Prior allergic reactions to iodine, or other study agents
- Significant traumatic injury within the past 4 weeks
- Ongoing or active infection requiring antibiotics or with a fever >38.1°C (>101° F) within 3 days of the first scheduled day of dosing
- Patients who are hospitalized.

7. STUDY DRUG ADMINISTRATION

7.1 DESCRIPTION OF STUDY DRUG

I-131-CLR1404 is supplied as a ready-for-use radiopharmaceutical for intravenous dosing that is provided as a clear, faint yellow to yellow, sterile solution consisting of I-131-CLR1404 (18-(*p*-[^{131}I]iodophenyl)octadecyl phosphocholine) and CLR1401 (18-(*para*-iodophenyl)octadecyl phosphocholine) in sodium chloride injection USP, ethanol USP/NF, polysorbate 20 NF, and sodium ascorbate USP. The study drug is packaged in a single-use glass vial and must be withdrawn through a 25 mm, 0.22 or 0.45 micron sterile filter prior to administration. **The appropriate volume of study drug sufficient to give a measured 10 mCi dose must then be diluted to 10 ml with normal saline solution (normal saline for injection) and thoroughly mixed prior to administration via slow IV infusion.**

I-131-CLR1404 is radioactive and should only be prepared, assayed, and administered by personnel who are licensed and trained to handle radioactive materials. Proper radiation shielding should be used during storage, preparation, assay, and administration of I-131-CLR1404. The study drug will arrive from Cellectar in a lead shipping container, and should remain in the lead shipping container for storage, out of direct sunlight and at room temperature.

The physical half-life of iodine-131 is approximately 8.02 days. Iodine-131 decays with beta emissions of 334 keV (7.3%) and 606 keV (89.9%), and gamma emissions of 284.3 keV and 364.5 keV. The therapeutic radiation effects are primarily from beta emissions, while gamma emissions are useful for imaging.

7.2 STUDY DRUG DOSAGES

Each patient will receive a single dose of 10 mCi I-131-CLR1404 (total mass dose ranging from 0.24 mg to 0.43 mg depending on the day of calibration and injection) through a freely-running peripheral intravenous catheter over a period of at least 10 minutes. The dose selection of 10 mCi was based on organ and whole body radiation dose estimates for I-131-CLR1404 determined from dosimetry studies performed in normal nude mice. These studies indicate that a 10 mCi dose of I-131-CLR1404 provides an acceptable range of organ dosimetry, approximately 2.68 rem/mCi. (An effective dose of 26.8 rem per 10 mCi is an approximate equivalent to the effective dose from 10 CT scans of the abdomen and pelvis.) The physics of imaging I-131 gamma photons predict that 10 mCi will be an adequate dose to obtain good dosimetry images and calculations over a period of 14 days.

Vital signs, including SaO_2, will be monitored pre-infusion, 5, 15, 30, and 60 minutes post infusion, and 4-6 hours post infusion. Patients will be observed for toxicity until their final visit at Day 42 post infusion.

See Nuclear Medicine and Radiopharmacy Instruction Manual for details on infusion of study drug.

If a patient withdraws from the study prematurely, (s)he will be strongly encouraged to continue taking thyroid protection medications for 14 days after the infusion of I-131-CLR1404.

7.3 TREATMENT DURATION

Patients will be observed for safety until study completion on Day 42. However, after 30 days on study if the patient is not experiencing any drug-related toxicity, based on the Investigator's discretion, the patient can continue further treatment with a clinically appropriate non-investigational therapy. While on therapy all efforts must be made to collect additional safety assessments until study completion on Day 42.

7.4 PATIENT REPLACEMENT

Patients who withdraw from the study within 14 days of infusion will be replaced and will be encouraged to return to the clinical site for further follow-up care for the purposes of safety assessment. Patients will be strongly encouraged to continue their thyroid protection medication for 14 days after the infusion of study drug.

7.5 STUDY DRUG ACCOUNTABILITY

The clinical site staff must maintain a careful inventory of the study drug. Study drug use will be recorded on a study drug inventory form. At a minimum, this form will contain the following information:

- Patient number and initials for each patient receiving study drug
- Date, quantity and dose (mCi) of study drug received by the clinical site
- Date, quantity and dose (mCi) of study drug dispensed
- Date, quantity and dose (mCi) administered

At each monitoring visit, Cellectar's clinical monitor will reconcile the information on the study drug inventory form with the actual inventory of study drug used at each clinical site. All used and expired radioactive vials of I-131-CLR1404 will be stored at the clinical site in long half-life radioactive storage per the clinical site's standard operating procedures (SOPs) until background levels are reached, approximately 80 days. When the used vials are no longer radioactive, drug accountability will be conducted by the clinical monitor and the vials will be shipped to Cellectar for disposal or discarded by the clinical site as per their SOP.

7.6 CONCOMITANT MEDICATIONS AND TREATMENTS

All intercurrent medical conditions will be treated at the discretion of the Investigator according to acceptable community standards of medical care. All concomitant medications and treatments will be documented on the appropriate case report form.

The following medications are not permitted during the study:

- Any other investigational treatment
- Any cytotoxic chemotherapy
- Any other systemic antineoplastic therapy including, but not limited to, immunotherapy, or monoclonal antibody therapy. (Ongoing treatment with hormonal therapy may be continued during the trial.)
- Systemically administered radioisotope therapy.
- Prophylactic hematopoietic growth factors, particularly filgrastim, peg-filgrastim and oprelvekin, should be avoided if at all possible during the study. Of course, patient safety must remain paramount, and if in the Investigator's opinion, such therapy is required during the study, growth factors should be used according to current ASCO guidelines. Medroxyprogesterone may be given to patients as an appetite stimulant.

Palliative external beam radiation therapy for bone pain is allowed at the Investigator's discretion, consistent with allowable limits previously stated.

The metabolism of I-131-CLR1404 is not completely understood. There are no known interactions with other medications.

Previous human studies have demonstrated that I-131-NM404, a compound closely related to I-131-CLR1404, has a plasma half-life of 113 hours, and less than 10% urinary excretion over 96 hours. Preclinical studies indicate that CLR1404 is primarily excreted through the hepatobiliary system (33%) over several half-lives of the drug.

Thyroid Protection Premedication

To protect the thyroid from any possible uptake of radioactive iodine, thyroid protection is required. Thyroid protective agents should be initiated at least 24 hours prior to, the day of, and for 14 days after administration of I-131-CLR1404. Premedication regimens vary, but they should include one of the following:

- Saturated solution of potassium iodide (SSKI) 4 drops orally t.i.d.
- Lugol's solution 20 drops orally t.i.d.
- Potassium iodide tablets 130 mg orally q.d.

Patients should not receive I-131-CLR1404 if they have not yet received at least 3 doses of SSKI, three doses of Lugol's solution, or one 130 mg potassium iodide tablet (at least 24 hours prior to the infusion.).

7.7 STUDY ENDPOINTS

7.7.1 Primary Endpoints

The primary endpoints are:

- Whole body and organ radiation dosimetry of I-131-CLR1404
- Determination of the millicurie (mCi) dose which is expected to deliver 35-40 centigray (cGy) of radiation to bone marrow, which will serve as the starting dose for a subsequent MTD study

7.7.2 Secondary Endpoints

The secondary endpoints are:

- Confirmation of safety of I-131-CLR1404
- Determination of the pharmacokinetic profile of I-131-CLR1404

8. TREATMENT PLAN

8.1 SCHEDULE OF PROCEDURES AND ASSESSMENTS

			Day 0							Day	Day	Day	Day	Day	Day	Day
	Screening		Infusion Day							1	2	3	6	14	30	42**
**Parameters to be Measured**	-7 days	-24 hrs	pre-dose	infusion	5 min	15 min	30 min	60 min	4-6 hrs	18-24 hrs	48 ±6 hrs	72 ± 6 hrs	144 ± 6 hrs	14 ± 1 day	30 ± 2 days	(final visit) ± 2 days
Signing of Informed Consent Form[1]	x															
Demographics	x															
12-lead ECG	x		x		x			x	x	x			x	x		
Serum Pregnancy Test (if applicable)		x														x
Body Weight	x		x											x		
ECOG Performance Status	x		x											x	x	x
Histologic or Cytologic Confirmation of Cancer	x															
Medical History	x	x	x													
Concomitant Medications Assessment	x	x	x	x	x	x	x	x	x	x	x	x	x	x	x	x
Physical Examination	x		x										x	x	x	x
Temperature, Vital Signs, S_aO_2[2]	x		x		x	x	x	x	x	x	x	x	x	x	x	x
Adverse Events				x	x	x	x	x	x	x	x	x	x	x	x	x

			Day 0							Day	Day	Day	Day	Day	Day	Day
	Screening		Infusion Day							1	2	3	6	14	30	42**
**Parameters to be Measured**	-7 days	-24 hrs	pre-dose	infusion	5 min	15 min	30 min	60 min	4-6 hrs	18-24 hrs	48 ±6 hrs	72 ± 6 hrs	144 ± 6 hrs	14 ± 1 day	30 ± 2 days	(final visit) ± 2 days
Urinalysis with Microanalysis[3]	x		x										x	x	x	x
CBC w/diff & Platelets[4]	x		x										x	x	x	x
Serum Chemistry[5]	x		x										x	x	x	x
Serum Lipids (fasting)[6]			x									x				
Patient Takes Thyroid Protection Medication[7]		x	x							x	x	x	x	x		
Blood sampling for Pharmacokinetic Analysis[8]			x		x	x	x	x	x	x	x	x	x	x	x	x
Blood sampling for biodistribution[9]			x			x		x	x	x	x	x	x	x	x	x
10 mCi I-131-CLR1404 infusion				x												
I-131-CLR1404 whole body images						X[10]			x	x	x	x	x	x		
Optional SPECT Imaging[11]												x	x	x		
Total Urine Collection[12]						←	—	—	→	↔	↔	↔ 4	↔	↔		

1. The informed consent form must be signed before any study-related procedures are performed.
2. Vital signs include supine blood pressure, respiration, pulse, and SaO_2.
3. Urinalysis with microanalysis: Urinary protein, glucose, ketones, hemoglobin, bilirubin, pH, specific gravity; Micro: red blood cells, white blood cells, epithelial cells, casts, crystals.

4. Complete blood count (CBC): Red blood cell (RBC) with mean corpuscular volume (MCV), mean corpuscular hemoglobin (MCH), mean corpuscular hemoglobin concentration (MCHC); hemoglobin, hematocrit, white blood cell (WBC) count [with differential % neutrophils, lymphocytes, monocytes, eosinophils, and basophils], absolute neutrophil count, and platelet count
5. Serum chemistry: Sodium, potassium, total protein, bicarbonate, chloride, BUN, GGT, creatinine, glucose, calcium, SGOT, SGPT, alkaline phosphatase, total bilirubin, albumin
6. Serum lipids: fasting total cholesterol, HDL, LDL, triglycerides
7. Patient takes thyroid protection medication each day on Days -24 hrs through Day 14.
8. Pharmacokinetic analysis (PK) samples: One EDTA (lavender top) tube; processed per instructions from Covance
9. Biodistribution samples: One EDTA (lavender top) tube; processed in nuclear medicine department (or designee)
10. Whole body imaging may be obtained 15-35 minutes post injection on Day 0.
11. At the Investigator's discretion, optional SPECT imaging may be obtained between Days 3 and 14
12. Total urine collection: Collected and analyzed in the nuclear medicine department (or designee.) Patient is given containers for each time period, in the following increments: 0-24 hrs [Day 0-Day 1]; 25-48 hrs [Day 1-Day 2]; 49-72 hrs [Day 2-Day 3]; 73-96 hrs [Day 3-Day 4]; 97-144 hrs [Days 4-6]; Days 6-10; Days 10-14.

** Final Visit

8.2 STUDY EVALUATIONS

8.2.1 Screening Period

The screening evaluation will include a complete medical and medication history, physical and laboratory examinations (CBC, serum chemistry and urinalysis), ECG, verification of the criteria for a diagnosis of cancer, documentation of previous therapy for malignancies and a pregnancy test (if female of childbearing potential). All patients must provide informed consent prior to any procedures being done for the study and must satisfy the entry criteria.

Within 7 Days Prior to the Day of Treatment

- Signed Informed Consent Form (Consent must be obtained prior to performance of any study specific tests or evaluations)
- Demographics (age, gender, race and ethnicity if applicable)
- 12-lead electrocardiogram (ECG)
- Body weight and height
- Assessment of Eastern Cooperative Oncology Group (ECOG) performance status
- Histological or cytological confirmation of cancer (at any time in the past)
- Collection of medical history and list of current medications
- Documentation of previous treatment for malignancies
- Physical examination
- Temperature, vital signs (supine blood pressure [BP], pulse, respiratory rate), and pulse oxygen saturation (S_aO_2)
- Urinalysis with microscopic analysis: urinary protein, glucose, ketones, hemoglobin, bilirubin, pH, specific gravity, red cell count, white cell count, epithelial cell count, casts, crystals.
- Complete blood count (CBC): Red blood cell (RBC) with mean corpuscular volume (MCV), mean corpuscular hemoglobin (MCH), mean corpuscular hemoglobin concentration (MCHC); hemoglobin, hematocrit, white blood cell (WBC) count [with differential % neutrophils, lymphocytes, monocytes, eosinophils, and basophils], absolute neutrophil count, and platelet count
- Serum chemistry: Sodium, potassium, total protein, bicarbonate, chloride, blood urea nitrogen (BUN), Gamma Glutamyl Transferase (GGT), creatinine, glucose, calcium, Serum Glutamic-Oxaloacetic Transaminase (SGOT), Serum Glutamic Pyruvic Transaminase (SGPT), alkaline phosphatase, total bilirubin, albumin

8.2.2 Treatment Period

Day -1 (24 hrs Pre-Dose)

- Patient takes thyroid protection medication.
- Serum pregnancy test in female patients of childbearing potential. (Women who have undergone hysterectomy, bilateral oophorectomy, bilateral tubal ligation or have been

without menses for 12 months and have LH, FSH and estradiol measurements within the post-menopausal range are considered to be of non-child bearing potential).

- Medical and concomitant medication history assessment
- (Remind patient of requirement for fasting blood sample on Day 0)

Day 0 (Day of Treatment, Pre-Dose)

On the day of dosing, prior to infusion, the following tests and procedures will be done:

- 12-lead ECG
- Assessment of ECOG performance status
- Medical history assessment
- Concomitant medications assessment
- Physical exam
- Body weight
- Temperature, vital signs, and S_aO_2
- Urinalysis with microscopic analysis
- CBC with differential and platelets
- Serum chemistry
- Serum lipids
- Patient takes thyroid protection medication
- Blood sample for pharmacokinetic (PK) analysis (one EDTA lavender top tube)
- Blood sample for biodistribution (one EDTA lavender top tube)

See Laboratory and Biodistribution Manual, provided separately, for details about handling and processing PK and biodistribution samples.

Treatment

- I-131-CLR1404 will be administered via a slow infusion of at least 10 minutes duration into a peripheral vein.
- Assessment of adverse events and concomitant medications

See Nuclear Medicine and Radiopharmacy Instruction Manual for detailed information about dose preparation and infusion technique.

Day 0 (5 minutes Post Infusion)

- 12-lead ECG
- Concomitant medications assessment
- Temperature, vital signs, S_aO_2
- Assessment of adverse events
- Blood sample for PK analysis (one EDTA lavender top tube)

Day 0 (15 minutes Post Infusion)

- Concomitant medications assessment
- Temperature, vital signs, and S_aO_2
- Assessment of adverse events
- Blood sample for PK analysis (one EDTA lavender top tube)
- Blood sample for biodistribution (one EDTA lavender top tube)

Day 0 (15-35 minutes Post Infusion)

- I-131-CLR 1404 whole body scans
- After scans are complete, begin 24 hour urine collection.

See Appendix D, Whole Body Scanning and Dosimetry, as well as the Nuclear Medicine and Radiopharmacy Manual, for detailed instructions on scanning parameters.

Day 0 (30 minutes Post Infusion)

- Concomitant medications assessment
- Temperature, vital signs, and S_aO_2
- Assessment of adverse events
- Blood sample for PK analysis (one EDTA lavender top tube)

Day 0 (60 minutes Post Infusion)

- 12-lead ECG
- Concomitant medications assessment
- Temperature, vital signs, and S_aO_2
- Assessment of adverse events
- Blood sample for PK analysis (one EDTA lavender top tube)
- Blood sample for biodistribution (one EDTA lavender top tube)

Day 0 (4-6 hours Post Infusion)

- 12-lead ECG
- Concomitant medications assessment
- Temperature, vital signs, and S_aO_2
- Assessment of adverse events
- Blood sample for PK analysis (one EDTA lavender top tube)
- Blood sample for biodistribution (one EDTA lavender top tube)
- I-131-CLR1404 whole body scans

Day 1 (18-24 hrs)

- 12-lead ECG
- Concomitant medications assessment
- Temperature, vital signs, and S_aO_2

- Assessment of adverse events
- Patient takes thyroid protection medication
- Blood sample for PK analysis (one EDTA lavender top tube)
- Blood sample for biodistribution (one EDTA lavender top tube)
- I-131-CLR 1404 whole body scans
- Start next 24 hour urine collection

Day 2 (48 $\pm$ 6 hrs)

- Concomitant medications assessment
- Temperature, vital signs, and S_aO_2
- Assessment of adverse events
- Patient takes thyroid protection medication
- Blood sample for PK analysis (one EDTA lavender top tube)
- Blood sample for biodistribution (one EDTA lavender top tube)
- I-131-CLR1404 whole body scans
- Start next 24 hour urine collection
- (Remind patient of requirement for fasting blood sample on Day 3)

Day 3 (72 $\pm$ 6 hrs)

- Concomitant medications assessment
- Temperature, vital signs, and S_aO_2
- Assessment of adverse events
- Serum lipids
- Patient takes thyroid protective medication
- Blood sample for PK analysis (one EDTA lavender top tube)
- Blood sample for biodistribution (one EDTA lavender top tube)
- I-131-CLR1404 whole body scans
- Optional SPECT imaging may be obtained between Days 3 and 14 at the Investigator's discretion
- Start next 24 hour urine collection, and give patient containers and instructions for collecting urine over the weekend at home

Day 4 (96 hrs)

- Patient takes thyroid protection medication
- Patient continues 24 hour urine collection at home.

Day 5 (120 hrs)

- Patient takes thyroid protection medication
- Patient continues 24 hour urine collection at home.

Day 6 (144 $\pm$ 6 hrs)

- 12-lead ECG
- Concomitant medications assessment
- Physical examination
- Temperature, vital signs, and S_aO_2
- Assessment of adverse events
- Urinalysis with microscopic analysis
- CBC with differential and platelet count
- Serum chemistry
- Patient takes thyroid protection medication
- Blood sample for PK analysis (one EDTA lavender top tube)
- Blood sample for biodistribution (one EDTA lavender top tube)
- I-131-CLR1404 whole body scans
- Optional SPECT imaging may be obtained between Days 3 and 14 at the Investigator's discretion
- Begin Days 7-10 and 11-14 urine collection; give patient containers and instructions for collecting and storing urine at home

Days 7-13

The following activities are conducted on each day:

- Patient takes thyroid protection medication
- Patient continues 24 hour urine collection at home

Day 14 $\pm$ 1 Day

The following procedures should be conducted at this visit:

- 12-lead ECG
- Body weight
- ECOG performance status
- Concomitant medications assessment
- Physical examination
- Temperature, vital signs, and S_aO_2
- Assessment of adverse events
- Urinalysis with microscopic analysis
- CBC with differential and platelet count
- Serum chemistry
- Patient takes thyroid protection medicine (completed after this dose)
- Blood sample for PK analysis (one EDTA lavender top tube)
- Blood sample for biodistribution (one EDTA lavender top tube)
- I-131-CLR1404 whole body scans

- Optional SPECT imaging may be obtained between Days 3 and 14 at the Investigator's discretion.
- Total urine, collection is completed at this visit

Day 30

- Concomitant medications assessment
- Assessment of ECOG performance status
- Physical exam
- Temperature, vital signs, and S_aO_2
- Assessment of adverse events
- Urinalysis with microscopic analysis
- CBC with differential and platelet count
- Serum chemistry
- Blood sample for PK analysis (one EDTA lavender top tube)
- Blood sample for biodistribution (one EDTA lavender top tube)

Day 42 (Final Visit)

- Serum pregnancy test (if applicable)
- Concomitant medications assessment
- Assessment of ECOG performance status
- Physical exam
- Temperature, vital signs, and S_aO_2
- Assessment of adverse events
- Urinalysis with microscopic analysis
- CBC with differential and platelet count
- Serum chemistry
- Blood sample for PK analysis (one EDTA lavender top tube)
- Blood sample for biodistribution (one EDTA lavender top tube)

8.2.3 Clinical Assessments

8.2.3.1 ECG

A standard supine resting 12-lead electrocardiogram will be performed at screening, pre-infusion, 5 and 60 minutes post infusion, as well as 4-6 hrs, 18-24 hrs, 144 hrs, and 14 days post infusion. The screening ECG must be obtained within 7 days prior to infusion of I-131-CLR1404.

8.2.3.2 Serum Pregnancy Test

A serum pregnancy test (if applicable) will be obtained at Day -1 or pre-treatment, and on Day 42 post infusion. Women who have undergone hysterectomy, bilateral oophorectomy, and bilateral tubal ligation or have been without menses for 12 months and have LH, FSH and estradiol measurements within the post-menopausal range are considered to be of non-child bearing potential

8.2.3.3 ECOG Performance Status

Determination of the patient's performance status based on the ECOG rating scale will be made at screening, pre-treatment, and at Days 14, 30, and 42. The screening ECOG performance status must be determined within 7 days prior to infusion of I-131-CLR1404.

8.2.3.4 Medical History and Medication Review

A thorough review of the patient's medical history, taking into account all recent and pertinent medical conditions including prior cancer treatment, will be performed during the screening phase of the study. All current and recent medications will be reviewed and recorded in the case report form. The medical history and medication review must be obtained within 7 days prior to the infusion of I-131-CLR1404.

8.2.3.5 Physical Exam

A detailed physical exam, noting all abnormalities and sites of palpable neoplastic disease, will be performed during screening, pre-dose, and Days 6, 14, 30, and 42. The screening physical exam must be performed within 7 days prior to the infusion of I-131-CLR1404.

8.2.3.6 Temperature, Vital Signs and Oxygen Saturation

Temperature, vital signs (pulse, supine blood pressure and respirations per minute) and oxygen saturation (measured with pulse oximeter) will be recorded at screening, pretreatment, 5, 15, 30, and 60 minutes post infusion, 4-6 hours post infusion, at each imaging visit, and at Days 30 and 42.

8.2.3.7 Urinalysis with Microscopic Analysis

Urinalysis with microscopic analysis will be performed at screening, pre-treatment, Days 6, 14, 30, and 42. The test should include measurements of urinary protein, glucose, ketones, hemoglobin, bilirubin, pH, specific gravity, red cell count, white cell count, epithelial cells, casts, and crystals. The screening urinalysis must be performed within 7 days prior to infusion of I-131-CLR1404.

8.2.3.8 Hematology

Blood will be drawn for hematology measurements at screening, pre-treatment, Days 6, 14, 30 and 42. The following testing should be performed at each time point: CBC with RBC, MCV, MCH, MCHC; hemoglobin, hematocrit, WBC count (with differential % neutrophils, lymphocytes, monocytes, eosinophils, basophils,) absolute neutrophil count, and platelet count. Screening labs must be drawn within 7 days prior to infusion of I-131-CLR1404.

8.2.3.9 Chemistry

Blood will be drawn for evaluating chemistry values at screening, pre-treatment, Days 6, 14, 30 and 42. The following labs should be performed: Sodium, potassium, total protein, bicarbonate, chloride, BUN, GGT, creatinine, glucose, calcium, SGOT, SGPT, alkaline phosphatase, total bilirubin, and albumin.

8.2.3.10 Lipids

Blood will be drawn for evaluating serum lipids pre-treatment, and 72 hours post infusion. The following labs should be performed (fasting): total cholesterol, HDL, LDL and triglycerides.

8.2.3.11 Pharmacokinetic Analysis

Blood will be drawn for plasma PK analysis at pre-treatment, 5, 15, 30, and 60 minutes post infusion, at each imaging visit, and on Days 14, 30 and 42. Blood samples for plasma PK analysis will be collected into EDTA (lavender top) anticoagulation tubes at each time point. Samples should be centrifuged and plasma collected, equally divided into two separate aliquots, and placed in 2 ml cryovials. Shipping materials will be provided for frozen shipment to Cellectar. For detailed instructions on handling PK samples refer to the Covance laboratory manual and the Laboratory and Biodistribution Manual.

8.2.3.12 Biodistribution Analysis

Blood will be drawn for radiologic biodistribution analysis at pre-treatment, 5, 15, 30, and 60 minutes post infusion, at each imaging visit, and on Days 30, and 42. Samples for biodistribution analysis will be collected into EDTA (lavender top) anticoagulation tubes at each time point. Duplicate aliquots of anticoagulated whole blood will be removed from the sample for analysis. Measured aliquots of whole blood samples will be counted in a well counter in the nuclear medicine department (or designated area) and compared with a known standard of I-131. For detailed instructions on handling biodistribution samples refer to the Laboratory and Biodistribution Manual.

8.2.3.13 Total Urine Collection for Radiologic Analysis

The patient will be instructed to collect 100% of their urine for the first 14 days of the study. If after analysis of the Day 6 urine sample there is no measureable radioactivity in the urine, the patient may be instructed to discontinue further urine collection at that time point. The urine collection will begin at the first void after the 15-35 minute post infusion scan. Urine will be collected in the following incremental time points: 0-24 hours, 25-48 hours, 49-72 hours, 73-96 hours, Days 4-6, Days 6-10, and Days 10-14. Patients will be given containers in which to collect and store their urine, and instructions on how to accomplish the task successfully with minimal spillage. The urine will be brought to the nuclear medicine department (or designated area) for processing. The nuclear medicine research technologist or designee will measure the total volume for each time period, draw duplicate aliquots from each volume, and count representative samples in the well counter in comparison with a known standard of I-131. For detailed instructions on handling biodistribution samples refer to the Laboratory and Biodistribution Manual.

8.2.3.14 Whole Body Imaging

Planar whole body nuclear medicine scans will be obtained of the patient, from the anterior and posterior projection, at the following times post injection: 15 – 35 minutes, 4-6 hours, 18-24 hours, 48 $\pm$ 6 hours, 72 $\pm$ 6 hours, 144 $\pm$ 6 hours, and 14 $\pm$ 1 Days. These images will be acquired with a high energy parallel hole collimator, on a triple energy window with a 20% symmetric peak, at a minimum of 256 x 1024 matrix, and for a speed of no faster than 10 cm/min. With the exception of the scan acquired 15 minutes post injection, the patient should void their bladder prior to each imaging session. For details regarding patient positioning, camera quality control, and image acquisition, refer to **Appendix D: Whole Body Scanning and Dosimetry** as well as the Nuclear Medicine and Radiopharmacy Manual.

8.2.3.15 Optional SPECT Imaging

At the Investigator's discretion, SPECT imaging may be obtained between Days 3 and 14 to further characterize the patient's tumor(s).

9. ADVERSE EVENT REPORTING

9.1 DEFINITIONS

9.1.1 Adverse Event

An *adverse event* (AE) is any untoward medical occurrence in a patient or clinical investigation subject administered a medicinal product. The adverse event does not necessarily have to have a causal relationship with this treatment. An adverse event can therefore be any unfavorable and unintended sign (including an abnormal laboratory finding, for example), symptom, or disease temporally associated with the use of a medicinal product, whether or not considered related to the medicinal product.

Baseline signs and symptoms will be recorded. All adverse events that occur (whether treatment related or not) will be recorded at each visit for the duration of the study. Adverse events occurring after the signing of the informed consent but prior to the first infusion of I-131-CLR1404 will be recorded in the medical history section of the case report form (CRF). Any worsening of a baseline condition, in frequency or severity, will be recorded as an AE.

An untoward medical event which occurs outside the period of follow-up as defined in the protocol will not be considered an adverse event. Worsening of a medical condition for which the efficacy of the study drug is being evaluated will not be considered an adverse event.

Investigators should follow patients with adverse events until one of the following occurs:

- The event has subsided or disappeared.
- The condition has stabilized.
- The event is resolved.
- The patient is lost to follow up.
- 42 days have passed since the infusion of the study drug. (Adverse events which are deemed to be related to the study drug will be followed until the event has resolved, even if the event lasts longer than the 42 days of study duration).

9.1.2 Unexpected Adverse Event

An *unexpected adverse event* is one for which the nature or severity of the event is not consistent with the applicable product information, e.g., the Investigator's brochure.

9.1.3 Serious Adverse Event

A *serious adverse event* (SAE) is any untoward medical occurrence that at any dose:

- Results in death
- Is life-threatening (an event in which the patient was at risk of death at the time of the event; it does not refer to an event which hypothetically might have caused death if it were more severe)
- Requires in-patient hospitalization or prolongation of existing hospitalization
- Results in persistent or significant disability/incapacity
- Is a congenital anomaly or birth defect
- Other important medical events that may not be immediately life-threatening or result in death or hospitalization but may jeopardize the patient or may require intervention to prevent one of the other outcomes listed above. Examples of such events are intensive treatment in an emergency room for allergic bronchospasm; blood dyscrasias or convulsions that do not result in hospitalization; or development of drug dependency or drug abuse.

The term “severe” is often used to describe the intensity (severity) of an event. The event itself may be of relatively minor medical significance (such as a severe headache). This is not the same as “serious”, which is based on patient/event outcome - a criterion usually associated with events that pose a threat to a patient’s life or functioning.

9.2 EXPEDITED REPORTING OF ADVERSE EVENTS

All fatal or life–threatening adverse events must be reported to Cellectar Medical Monitor immediately by telephone or e-mail. Within 24 hours of first knowledge of the event, the SAE form must be faxed to the Medical Monitor whether full information regarding the event is known or not. If full information is not known, additional follow-up by the Investigator will be required.

All other SAEs must be reported to Cellectar Medical Monitor within 24 hours by phone, e-mail or fax. Within 24 hours of first knowledge of the event, the SAE form must be faxed to the Medical Monitor whether full information regarding the event is known or not. If full information is not known, additional follow-up by the Investigator will be required.

Follow-up reports must be sent to the Medical Monitor within 48 hours of first knowledge of follow-up information on SAEs.

Report all serious adverse events to:

Cellectar Medical Monitor
3301 Agriculture Drive
Madison, WI 53716
Fax: (608) 327-8140
Tel: (608) 630-4481

All SAEs will be evaluated by Cellectar Medical Monitor. If the requirements for expedited reporting are met, Cellectar will so report the adverse event to all regulatory authorities with jurisdiction over ongoing studies with the study drug, and to all other Investigators involved in clinical trials with the study drug. The Investigator must report all SAEs reported to regulatory authorities in an expedited manner to the local IRB.

9.3 DOCUMENTING ADVERSE EVENTS

The Investigator should elicit information regarding the occurrence of adverse events through open-ended questioning of the patient, physical examination and review of laboratory results.

All adverse events, whether serious or not, will be described in the source documents and the adverse event page of the case report form. All new events, as well as those that worsen in intensity or frequency relative to baseline, that occur after administration of study drug through the period of protocol-specified follow-up must be captured.

Information to be reported in the description of each adverse event includes but not limited to:

- Medical diagnosis of the event (if a medical diagnosis cannot be determined, a description of each sign or symptom characterizing the event should be recorded)
- The date of onset of the event
- The date of resolution of the event
- Whether the event is serious or not
- Action taken:
  - None
  - Change in the study drug administration (e.g., temporary interruption in dosing)
  - Drug treatment required (specify)
  - Non-drug treatment required (specify)
  - Hospitalization or prolongation of hospitalization required (complete SAE page)
  - Diagnostic procedure performed (specify)
  - Patient discontinued from the study (complete final visit section of the CRF)
- Outcome:
  - Event resolved
  - Event resolved with sequelae
  - Event ongoing
  - Patient died (notify Cellectar immediately, complete the SAE page and final visit section of the CRF)
- Patient condition is related or not related to the study drug
- Patient's condition is intermittent or continuing
- The grade of the event based on the NCI common toxicity criteria

9.4 GRADING AND RELATEDNESS OF ADVERSE EVENTS

9.4.1 Grading of Severity of an Adverse Event

The severity of each adverse event will be graded as mild, moderate, severe, life threatening, or death which corresponds to Grades 1, 2, 3, 4 and 5, respectively on the National Cancer Institute Common Toxicity Criteria for Adverse Events (NCI CTCAE) version 3.0. These criteria must be used in grading the severity of adverse events. The criteria can be found at: www.fda.gov/cder/cancer/toxicityframe.htm.

For those adverse events which are not listed as part of the NCI CTC, the same grading system should be used, where:

Grade 1: **Mild** corresponds to an event not resulting in disability or incapacity and which resolves without intervention;

Grade 2: **Moderate** corresponds to an event not resulting in disability or incapacity but which requires intervention;

Grade 3: **Severe** corresponds to an event resulting in temporary disability or incapacity and which requires intervention;

Grade 4: **Life-threatening** corresponds to an event in which the patient was at risk of death at the time of the event;

Grade 5: **Fatal** corresponds to an event that results in the death of the patient.

9.4.2 Relatedness to Study Drug

The Investigator must attempt to determine if an adverse event is in some way related to the use of the study drug. This relationship should be described as follows:

1. Related: The reaction follows a reasonably temporal sequence from administration of the drug and follows a known response pattern to a similar class of drugs.

2. Not Related: Concomitant illness, accident or event with no reasonable association with treatment, or the reaction has little or no temporal sequence from administration of the study drug, and/or a more likely alternative etiology exists.

If discernible at the time of completing an adverse event CRF, a specific disease or syndrome rather than individual associated signs and symptoms should be identified by the Investigator and recorded on the appropriate adverse event CRF. However, if an observed or reported sign, symptom, or clinically significant laboratory anomaly is not considered by the Investigator to be a component of a specific disease or syndrome, then it should be recorded as a separate adverse event on the appropriate CRF. (Clinically significant laboratory abnormalities are those that are identified as such by the Investigator and/or are those that require intervention).

10. PATIENT WITHDRAWAL

10.1 CRITERIA FOR PATIENT WITHDRAWAL

Patients may withdraw their consent to participate in the study at any time without prejudice to his/her future medical care by the physician or at the institution.

If a patient withdraws consent, the date and reason for consent withdrawal should be documented. Patient data will be included in the analysis up to the date of the consent withdrawal.

Wherever possible, the tests and evaluations listed for the final visit should be carried out. The sponsor should be notified of all study withdrawals in a timely manner.

Patients may be removed from the study if any one or more of the following events occur:

- Patient withdrawal of consent
- Dose limiting toxicity
- Discretion of Principal Investigator (reason to be documented on CRF)
- Patient becomes pregnant or begins breast-feeding
- Patient lost to follow-up (defined as the inability to contact the patient on two separate occasions over a period of 1 week)
- Administrative reasons (e.g., the patient is transferred to hospice care)
- An adverse event, which in the opinion of the Investigator, precludes further study participation or fulfills the protocol requirements for withdrawal (e.g., the development of dose limiting toxicity)
- Patient develops an intercurrent illness that precludes further participation, or requires a prohibited concomitant treatment

10.2 DOSE LIMITING CRITERIA

In general, Dose Limiting Toxicities (DLTs) will be considered as toxicities that are related to treatment with I-131-CLR1404 and that are:

1. Hematological

   a. Any CTCAE Grade 4 toxicity (except Grade 4 neutropenia without concurrent fever which must be present for ≥7 days to constitute a DLT)
   b. CTCAE Grade 3 neutropenia with fever
   c. CTCAE Grade 3 thrombocytopenia associated with bleeding

2. Non-Hematological

   a. Any CTCAE Grade 3 toxicity despite adequate supportive care (supportive care limited to nausea, vomiting and diarrhea only)
   b. Any CTCAE Grade 4 toxicity

If 2 patients experience a DLT, study enrollment will be stopped pending discussions with the Principal Investigator, Cellectar Medical Monitor and the Agency.

10.3 STUDY DISCONTINUATION

The Sponsor reserves the right to discontinue the study at any time. The Principal Investigator will be compensated for reasonable expenses incurred if it is necessary to terminate the study. Cellectar will not compensate the Principal Investigator for evaluation of cases in which the procedures and evaluations were conducted in a manner other than that specified in the protocol. Reasons for early study discontinuation may include, but are not limited to,

- Unacceptable toxicity of study drug
- Request to discontinue the study from a regulatory authority

- Protocol violations
- Poor enrollment
- Company business decision
- Difficulties in manufacturing the investigational product.

10.4 REPORTING AND FOLLOW-UP OF PREGNANCY

Cellectar's Medical Monitor must be immediately informed about any patients who become pregnant while on study. Pregnancies occurring up to 90 days after the completion of the study medication must also be reported to Cellectar.

The Investigator should inform the patient of the risks (if any) of continuing with the pregnancy and the possible effects on the fetus. Cellectar may recommend monitoring the pregnancy until its' conclusion.

Pregnancy occurring in the partner of a male patient participating in the study should also be reported to Cellectar. The partner should also be informed of the risks of continuing with the pregnancy, the possible effects on the fetus (if any), and recommendations for monitoring until conclusion of the pregnancy, if applicable.

11. DATA ANALYSIS

11.1 CASE REPORT FORMS

All data required by the study will be entered onto case report forms supplied by Cellectar. Only those clinical site staff so authorized may enter data onto the case report forms. All entries must be legible and made in black ink pen. If an entry error is made, a single line will be placed through the incorrect entry. The correct entry will then be made, with the correction dated and initialed by the person making the entry. Any corrections to data entered into the CRF must be made in such a way that the original entry is not obscured.

11.2 DATA QUALITY ASSURANCE

Case report forms will be checked for correctness against source document data by Cellectar's monitors. If any entries on the CRFs are incorrect, incomplete or illegible, the monitor will ask the Investigator or the study clinical site staff to make appropriate corrections. Once the CRF page is complete, it will be delivered to Cellectar for entry into an electronic database. Prior to this, the completed CRF will again be reviewed for completeness, consistency and legibility. Any discrepancies will be noted on a data clarification form (DCF) which will be sent to the clinical site. The discrepancy will be clarified on the DCF, which will then be returned to Cellectar's data entry center.

11.3 SAMPLE SIZE

As there is little information on the effect of I-131-CLR1404, this study is meant to be hypothesis generating and no formal sample size calculation will be performed.

11.4 DOSIMETRY CALCULATION

Biodistribution data will be analyzed using the OLINDA methodology to produce time/activity curves and generate organ specific radiation absorbed doses from a 10 mCi injection of I-131-CLR1404. This data, along with blood sampling drawn at specific time points post injection and total urine collection for up to 14 days, will be used to extrapolate and predict organ specific and total body radiation absorbed doses from therapeutic injections of I-131CLR1404. Dosimetry calculations will be instrumental in characterizing the dose escalation schema for subsequent Phase 1/2 protocols.

11.5 STATISTICAL ANALYSIS

All baseline patient characteristics will be summarized in a tabular format.

Safety data will be described for all patients receiving an infusion of I-131-CLR1404. Safety data will include values for hematology, serum chemistry, urinalysis and vital signs, results of ECG determinations and adverse events. The proportion of patients experiencing adverse events will be reported and SAEs will be summarized.

The I-131-CLR1404 plasma concentration-time data will be determined for each patient. The following PK parameters will be determined:

C_{max}
AUC $_{(0\text{-}144hr)}$
Plasma half-life $_{(t1/2)}$
Plasma clearance $_{(Cl)}$
Vd

12. ADMINISTRATIVE PROCEDURES

12.1 PATIENT INFORMED CONSENT

No study related procedures will be performed until a patient or their legal representative has given written informed consent to participate in the study. Cellectar will provide to the Investigator a sample informed consent form (ICF) that conforms to all the requirements for informed consent according to the International Conference on Harmonization Guidance on Good Clinical Practice (ICH GCP) and US FDA guidelines (21 CFR 50). However, it is up to the Investigator to provide a final ICF that may include additional elements required by the Investigator's institution or local regulatory authorities. The Institutional Review Board (IRB) for each clinical site must approve the ICF prior to study activation; changes to the ICF during the course of the study may also require IRB approval. The ICF must clearly describe the potential risks and benefits of the study, and each prospective participant must be given adequate

time to discuss the study with the Investigator or clinical site staff and to decide whether or not to participate. Each patient who agrees to participate in the study and who signs the ICF will be given a copy of the signed, dated, and witnessed document. A second copy of the signed, dated, and witnessed ICF will be retained by the Investigator in the study files.

12.2 ETHICAL CONDUCT OF THE STUDY AND IRB APPROVAL

The study will be conducted according to the principles of the 2000 version of the Declaration of Helsinki (Appendix B), ICH GCP, and the requirements of all local regulatory authorities regarding the conduct of clinical studies and the protection of human patients.

The Investigator will submit the protocol, the Investigator's Brochure, the ICF and any other material used to inform patients about the study to the local IRB for approval prior to enrolling any patient into the study. The IRB should be duly constituted as outlined in 21 CFR 50. Approval must be in the form of a letter signed by the Chairperson of the IRB or the Chairperson's designee, must be on IRB stationary and must include the protocol by name and/or designated number. If an Investigator is a member of the IRB, the approval letter must stipulate that the Investigator did not participate in the final vote, although the Investigator may participate in the discussion of the study. The Investigator will also inform the IRB of any serious adverse events that the Sponsor reports to regulatory authorities and will provide to the IRB a final summary of the results of the study at the conclusion of the study.

Any amendments to the protocol, other than administrative changes, will be submitted to the IRB for review and written approval will be received before implementation. Such written approval will be forwarded to Cellectar by the Investigator.

12.3 PROTOCOL AMENDMENTS

The protocol will only be modified by Cellectar. Changes to the protocol must be in the form of a written amendment; changes other than those of a simple administrative nature (e.g., a new telephone number for a Medical Monitor) must be submitted by the Investigator to the local IRB and such amendments will only be implemented after written approval of the requisite IRB. All amendments will also be submitted to local regulatory authorities by Cellectar as required by local regulation.

Protocol changes to eliminate an immediate hazard to a study patient may be implemented by the Investigator immediately. The Investigator must then immediately inform the IRB and Cellectar who will immediately notify local regulatory authorities.

If an amendment substantially alters the study design or increases the potential risk to the patient, the ICF must be revised and submitted to the IRB for review and approval. The revised ICF must be used to obtain consent from patients currently enrolled in the study if they are affected by the amendment, and the new ICF must be used to obtain consent from new patients prior to enrollment.

12.4 MONITORING

A clinical monitor will make regularly scheduled trips to the clinical site to review the progress of the study. The actual frequency of monitoring trips will depend on the enrollment rate and performance at each clinical site. At each visit, the monitor will review various aspects of the study including, but not limited to, screening and enrollment logs, compliance with the protocol and with the principles of ICH GCP, completion of case report forms, source data verification, study drug accountability, and storage, facilities and staff.

During scheduled monitoring visits, the Investigator and the clinical site staff must be available to meet with the study monitor in order to discuss the progress of the study, make necessary corrections to case report form entries, respond to data clarification requests and respond to any other study-related inquiries of the monitor.

In addition to the above, a representative from Cellectar's auditing staff or government inspectors may review the conduct/results of the study at the clinical site. The Investigator must promptly notify Cellectar of any audit requests by regulatory authorities.

12.5 PRE-STUDY DOCUMENTATION

Prior to initiating the study, the Investigator will provide to Cellectar the following documents:

- Signed FDA Form 1572
- Current (within 2 years), dated and signed curriculum vitae (CV) for the Principal Investigator and each sub-Investigator listed on the FDA Form 1572
- Copy of the Investigator's medical license from the state in which the study is being conducted; alternatively, the Investigator's medical license number on the Investigator's CV will suffice as evidence of a license to practice medicine
- Letter from the IRB stipulating approval of the protocol, the informed consent document and any other material provided to potential study participants with information about the study (e.g., advertisements)
- Copy of the IRB approved ICF
- Current IRB membership list for IRBs without a multiple project assurance number or an IRB organization number under the FederalWide Assurance program (http://www.hhs.gov/ohrp/humansubjects/assurance/filasurt.htm)
- Signed Investigator Protocol Agreement (Appendix A)
- Completed Financial Disclosure Form
- Current laboratory certification for the reference laboratory
- List of current laboratory normal values for the reference laboratory
- Radioactive Materials License

12.6 CONFIDENTIALITY

It is the responsibility of the Investigator to maintain confidentiality of all patients participating in the study to the extent possible. Case report forms and other documents submitted to Cellectar must never contain the name of a study participant. Each patient in the study will be identified by a unique identifier that will be used on all CRFs and any other material submitted to Cellectar. All case report forms and any identifying information must be kept in a secure location with access limited to the study staff directly participating in the study.

Personal medical information may be reviewed by representatives of Cellectar, of the IRB or of regulatory authorities in the course of monitoring the progress of the study. Every reasonable effort will be made to maintain such information as confidential.

The results of the study may be presented in reports, published in scientific journals, or presented at medical meetings; however, patient names will never be used in any reports about the study.

12.7 SOURCE DOCUMENTS

The Investigator will maintain patient records separate from case report forms in the form of clinical charts, medical records, original laboratory, radiology and pathology reports, pharmacy records, etc. The Investigator will document in the clinic chart or medical record the name and number of the study and the date on which the patient signed the informed consent prior to the patient's participation in the study. Source documents must completely reflect the nature and extent of the patient's medical care, and must be available for source document verification against entries in the case report forms when Cellectar's monitor visits the clinical site. All information obtained from source documents will be kept in strict confidentiality.

12.8 RECORD RETENTION

The Investigator will retain the records of the study for 2 years following the date that a marketing application for the study drug is approved, or if no marketing application is filed, or if such an application is not approved, for 2 years after the IND has been closed. Cellectar will notify Investigators when retention of study records is no longer required. All study records must be maintained in a safe and secure location that allows for timely retrieval, if needed.

Study records that must be retained include but are not limited to: copies of case report forms, signed ICFs, correspondence to and from the IRB, study drug dispensing and inventory records, source documents, clinic charts, medical records, laboratory results, radiographic reports and screening/enrollment logs.

Should the Investigator relocate or retire, or should there be any changes in the archival arrangements for the study records, Cellectar must be notified. The responsibility for maintaining the study records may be transferred to another suitable individual, but Cellectar must be notified of the identity of the individual assuming responsibility for maintaining the study records and the location of their storage. If no other individual at the clinical site is willing to assume this responsibility, Cellectar will assume responsibility for maintaining the study records.

12.9 PUBLICATION POLICY

All information and data obtained in the course of the study are the property of Cellectar and are considered confidential. Publication of information and data is governed by the contract between Cellectar and the clinical site.

For all publications, the order of authorship will be assigned by the Research and Publications Committee, consisting of selected representatives of Cellectar, selected external consultants and experts, and one Principal Investigator from each institution which has successfully enrolled and completed the protocol with at least one patient.

APPENDIX A: INVESTIGATOR AGREEMENT

I have reviewed the protocol entitled A Phase 1 Multi-Center, Open-Label, Dosimetry Study of I-131-CLR1404 in Patients with Relapsed or Refractory Advanced Solid Tumors who have Failed Standard Therapy or for Whom no Standard Therapy Exists and agree that it contains all the information necessary to conduct the study as required. I will conduct the study in accordance with the principles of ICH GCP and the Declaration of Helsinki.

I will maintain as confidential all written and verbal information provided to me by Cellectar, including but not limited to, the protocol, case report forms, Investigator's Brochure, material supplied at Investigator meetings, minutes of teleconferences, etc. Such material will only be provided as necessary to clinical site personnel involved in the conduct of the study, the IRB, or local regulatory authorities.

I will obtain written informed consent from each prospective study patient or each prospective study patient's legal representative prior to conducting any protocol-specified procedures. The ICF used will have the approval of the local IRB.

I will maintain adequate source documents and record all observations, treatments and procedures pertinent to study patients in their medical records. I will accurately complete the case report forms supplied by Cellectar in a timely manner. I will make certain that my facilities and records will be available for inspection by representatives of Cellectar, the local IRB or regulatory authorities. I will ensure that my staff and I are available to meet with representatives of Cellectar during regularly scheduled monitoring visits.

I will notify Cellectar within 24 hours of any serious adverse events. Following this notification, a written report describing the serious adverse event will be provided to Cellectar as soon as possible, but no later than three days following the initial notification.

I, as the Investigator, understand that I am responsible for all decisions relating to patient care.

**Investigator Name (Print)**	**Investigator Signature**	**Date**

Name of Institution

APPENDIX B: DECLARATION OF HELSINKI

Ethical Principles for Medical Research Involving Human Patients

Adopted by the 18th WMA General Assembly Helsinki, Finland, June 1964 and amended by the
29th WMA General Assembly, Tokyo, Japan, October 1975
35th WMA General Assembly, Venice, Italy, October 1983
41st WMA General Assembly, Hong Kong, September 1989
48th WMA General Assembly, Somerset West, Republic of South Africa, October 1996
52nd WMA General Assembly, Edinburgh, Scotland, October 2000

A. INTRODUCTION

1. The World Medical Association has developed the Declaration of Helsinki as a statement of ethical principles to provide guidance to physicians and other participants in medical research involving human patients. Medical research involving human patients includes research on identifiable human material or identifiable data.

2. It is the duty of the physician to promote and safeguard the health of the people. The physician's knowledge and conscience are dedicated to the fulfillment of this duty.

3. The Declaration of Geneva of the World Medical Association binds the physician with the words, "The health of my patient will be my first consideration," and the International Code of Medical Ethics declares that, "A physician shall act only in the patient's interest when providing medical care which might have the effect of weakening the physical and mental condition of the patient."

4. Medical progress is based on research which ultimately must rest in part on experimentation involving human patients.

5. In medical research on human patients, considerations related to the well-being of the human patient should take precedence over the interests of science and society.

6. The primary purpose of medical research involving human patients is to improve prophylactic, diagnostic and therapeutic procedures and the understanding of the etiology and pathogenesis of disease. Even the best proven prophylactic, diagnostic, and therapeutic methods must continuously be challenged through research for their effectiveness, efficiency, accessibility and quality.

7. In current medical practice and in medical research, most prophylactic, diagnostic and therapeutic procedures involve risks and burdens.

8. Medical research is subject to ethical standards that promote respect for all human beings and protect their health and rights. Some research populations are vulnerable and need special protection. The particular needs of the economically and medically disadvantaged must be recognized. Special attention is also required for those who cannot give or refuse

consent for themselves, for those who may be patient to giving consent under duress, for those who will not benefit personally from the research and for those for whom the research is combined with care.

9. Research Investigators should be aware of the ethical, legal and regulatory requirements for research on human patients in their own countries as well as applicable international requirements. No national ethical, legal or regulatory requirement should be allowed to reduce or eliminate any of the protections for human patients set forth in this Declaration.

B. BASIC PRINCIPLES FOR ALL MEDICAL RESEARCH

10. It is the duty of the physician in medical research to protect the life, health, privacy, and dignity of the human patient.

11. Medical research involving human patients must conform to generally accepted scientific principles, be based on a thorough knowledge of the scientific literature, other relevant sources of information, and on adequate laboratory and, where appropriate, animal experimentation.

12. Appropriate caution must be exercised in the conduct of research which may affect the environment, and the welfare of animals used for research must be respected.

13. The design and performance of each experimental procedure involving human patients should be clearly formulated in an experimental protocol. This protocol should be submitted for consideration, comment, guidance, and where appropriate, approval to a specially appointed ethical review committee, which must be independent of the Investigator, Cellectar or any other kind of undue influence. This independent committee should be in conformity with the laws and regulations of the country in which the research experiment is performed. The committee has the right to monitor ongoing studies. The researcher has the obligation to provide monitoring information to the committee, especially any serious adverse events. The researcher should also submit to the committee, for review, information regarding funding, sponsors, institutional affiliations, other potential conflicts of interest and incentives for patients.

14. The research protocol should always contain a statement of the ethical considerations involved and should indicate that there is compliance with the principles enunciated in this Declaration.

15. Medical research involving human patients should be conducted only by scientifically qualified persons and under the supervision of a clinically competent medical person. The responsibility for the human patient must always rest with a medically qualified person and never rest on the patient of the research, even though the patient has given consent.

16. Every medical research project involving human patients should be preceded by careful assessment of predictable risks and burdens in comparison with foreseeable benefits to

the patient or to others. This does not preclude the participation of healthy volunteers in medical research. The design of all studies should be publicly available.

17. Physicians should abstain from engaging in research projects involving human patients unless they are confident that the risks involved have been adequately assessed and can be satisfactorily managed. Physicians should cease any investigation if the risks are found to outweigh the potential benefits or if there is conclusive proof of positive and beneficial results.

18. Medical research involving human patients should only be conducted if the importance of the objective outweighs the inherent risks and burdens to the patient. This is especially important when the human patients are healthy volunteers.

19. Medical research is only justified if there is a reasonable likelihood that the populations in which the research is carried out stand to benefit from the results of the research.

20. The patients must be volunteers and informed participants in the research project.

21. The right of research patients to safeguard their integrity must always be respected. Every precaution should be taken to respect the privacy of the patient, the confidentiality of the patient's information and to minimize the impact of the study on the patient's physical and mental integrity and on the personality of the patient.

22. In any research on human beings, each potential patient must be adequately informed of the aims, methods, sources of funding, any possible conflicts of interest, institutional affiliations of the researcher, the anticipated benefits and potential risks of the study and the discomfort it may entail. The patient should be informed of the right to abstain from participation in the study or to withdraw consent to participate at any time without reprisal. After ensuring that the patient has understood the information, the physician should then obtain the patient's freely-given informed consent, preferably in writing. If the consent cannot be obtained in writing, the non-written consent must be formally documented and witnessed.

23. When obtaining informed consent for the research project the physician should be particularly cautious if the patient is in a dependent relationship with the physician or may consent under duress. In that case the informed consent should be obtained by a well-informed physician who is not engaged in the investigation and who is completely independent of this relationship.

24. For a research patient who is legally incompetent, physically or mentally incapable of giving consent or is a legally incompetent minor, the Investigator must obtain informed consent from the legally authorized representative in accordance with applicable law. These groups should not be included in research unless the research is necessary to promote the health of the population represented and this research cannot instead be performed on legally competent persons.

25. When a patient deemed legally incompetent, such as a minor child, is able to give assent to decisions about participation in research, the Investigator must obtain that assent in addition to the consent of the legally authorized representative.

26. Research on individuals from whom it is not possible to obtain consent, including proxy or advance consent, should be done only if the physical/mental condition that prevents obtaining informed consent is a necessary characteristic of the research population. The specific reasons for involving research patients with a condition that renders them unable to give informed consent should be stated in the experimental protocol for consideration and approval of the review committee. The protocol should state that consent to remain in the research should be obtained as soon as possible from the individual or a legally authorized surrogate.

27. Both authors and publishers have ethical obligations. In publication of the results of research, the Investigators are obliged to preserve the accuracy of the results. Negative as well as positive results should be published or otherwise publicly available. Sources of funding, institutional affiliations and any possible conflicts of interest should be declared in the publication. Reports of experimentation not in accordance with the principles laid down in this Declaration should not be accepted for publication.

C. ADDITIONAL PRINCIPLES FOR MEDICAL RESEARCH COMBINED WITH MEDICAL CARE

28. The physician may combine medical research with medical care, only to the extent that the research is justified by its potential prophylactic, diagnostic or therapeutic value. When medical research is combined with medical care, additional standards apply to protect the patients who are research patients.

29. The benefits, risks, burdens and effectiveness of a new method should be tested against those of the best current prophylactic, diagnostic, and therapeutic methods. This does not exclude the use of placebo, or no treatment, in studies where no proven prophylactic, diagnostic or therapeutic method exists.

30. At the conclusion of the study, every patient entered into the study should be assured of access to the best proven prophylactic, diagnostic and therapeutic methods identified by the study.

31. The physician should fully inform the patient which aspects of the care are related to the research. The refusal of a patient to participate in a study must never interfere with the patient-physician relationship.

32. In the treatment of a patient, where proven prophylactic, diagnostic and therapeutic methods do not exist or have been ineffective, the physician, with informed consent from the patient, must be free to use unproven or new prophylactic, diagnostic and therapeutic measures, if in the physician's judgment it offers hope of saving life, re-establishing

health or alleviating suffering. Where possible, these measures should be made the object of research, designed to evaluate their safety and efficacy. In all cases, new information should be recorded and, where appropriate, published. The other relevant guidelines of this Declaration should be followed.

APPENDIX C: ECOG PERFORMANCE STATUS*

Grade	Performance Status
0	Fully active, able to carry on all pre-disease performance without restriction
1	Restricted in physically strenuous activity but ambulatory and able to carry out work of a light or sedentary nature, e.g., light house work, office work
2	Ambulatory and capable of all self-care but unable to carry out any work activities. Up and about more than 50% of waking hours
3	Capable of only limited self-care, confined to bed or chair more than 50% of waking hours
4	Completely disabled. Cannot carry on any self-care. Totally confined to bed or chair
5	Dead

*As published in the Am J Clin Oncol:
Oken, M.M., Creech, R.H., Tormey, D.C., Horton, J., Davis, T.E., McFadden, E.T., Carbone, P.P.: Toxicity And Response Criteria Of The Eastern Cooperative Oncology Group. Am J Clin Oncol 5:649-655, 1982.

ECOG.org [homepage on the Internet], Eastern Cooperative Oncology Group, Robert Comis MD, Group Chair. [updated 2006 July 27; cited 2008 July 28]. Available from: http://www.ecog.org/.

APPENDIX D: WHOLE BODY SCANNING AND DOSIMETRY

Quality Control

Image quality and reproducibility is of primary importance in the collection of nuclear medicine data. Data collected for dosimetry calculation and for image review must adhere to strict quality control standards in order to guarantee the highest possible image quality. Because camera systems can vary, each camera that will be used for imaging patients must be qualified by Cellectar prior to a patient being enrolled into the study.

Pre-Study Quality Control (Prior to Patient Enrollment)

To qualify a camera for use, the following pre-study camera QC procedures must be performed, and documentation sent to Cellectar for review and approval. Camera qualification must be done prior to utilizing a given camera for the study.

1. I-131 energy correction performed per manufacturer's specified timelines.
2. Extrinsic resolution check with quadrant bar phantom using Co-57 sheet source on high energy collimator. Record smallest line of bars that can be visualized. Record the smallest line of bars that appear linear. Weekly flood images will be compared as a way to monitor extrinsic resolution over time.
3. A minimum of 3 million count extrinsic flood field uniformity with Co-57 sheet source on high energy collimator, with quantitative analysis to calculate differential uniformity. There should be no more than 5% variance between minimum and maximum pixel count.
4. Camera calibration testing of standard I-131 activity source. Specific instructions for this calibration will be provided at the time of pre-study qualification.
5. Calculation of attenuation coefficient for Co-57 source and I-131 source. Specific instructions for these calculations will be provided at the time of pre-study qualification.

Weekly Camera Quality Control

Once during each week that a patient is imaged perform the following quality control procedure documented for qualified camera:

Extrinsic resolution check with quadrant bar phantom using Co-57 sheet source on high energy collimator. Record the smallest line of bars that can be visualized. Record the smallest line of bars that appears linear. Note any changes from the previous observation. If there is any degradation of extrinsic resolution, corrective action is to be taken per clinical site SOPs.

Daily Camera Quality Control

On the day that any patient is scanned, the following quality control procedures must be documented:

1. A minimum of 3 million count extrinsic flood field uniformity with Co-57 sheet source on high energy collimator, with quantitative analysis to calculate differential uniformity. There should be no more than 5% variance between minimum and maximum pixel count.
2. Energy window verification for I-131 (screen capture of peak graph, if possible; if not possible, document that peak was verified.)

General Comments on Quality Control

1. If a camera becomes unusable due to a quality control breakdown on the day of a scheduled patient, call Cellectar to discuss the most appropriate option.
2. Ideally, the patient would be rescheduled and imaged on the same camera as was previously used or had already been qualified.
3. Do not use a camera with a known quality control failure to collect study data.

Note: The same camera must be used for all images on one patient. Different cameras may be used for different patients, as long as quality control is sufficiently documented for each camera.

Attenuation Scan: Pre-Injection Day 0

1. Acquire a "blank scan" with the Co-57 source only, anterior and posterior projection, on the Co-57 window, running the entire length of the table as if the patient were on the table.
2. Acquire anterior and posterior whole body image of patient PRIOR TO INJECTION of I-131-CLR1404 with patient between Co-57 sheet source and collimator, on the Co-57 window. Data will be collected from the detector in the anterior position, while the Co-57 sheet source is placed directly on the detector in the posterior position. See parameters listed in Table 4 below.
3. The same camera used for the attenuation scan must be used for all patient images.
4. Position the patient flatly supine, with head and neck facing straight forward, arms laying to the side of the body with no overlap to soft tissue, legs uncrossed. Attempt to reposition the patient as consistently as possible for all future imaging sessions.

Table 4

Parameters for Attenuation Scan	Description	Note
Energy window	20% symmetric, peaked on 122 keV (110-134 keV)	
Scan Speed	20 cm/min	Note faster than patient scans
Matrix	256 x 1024 minimum	
Attenuation Correction	NA	
Scatter Correction	NA	
Collimator	Parallel hole, high energy	Ultra high energy collimators for 511 keV SPECT will not be used for this protocol
Views	Anterior (place Co-57 source on posterior detector)	Acquire patient images with ant detector – place Co-57 sheet source on post detector
Data to be recorded		Data worksheet will be provided

Day 0: 15-35 min post injection

1. Patients should not void their bladder between injection and imaging time, because all activity must be accounted for in the immediate post injection images.
2. The same camera that was used for the attenuation scan must be used for all patient images.
3. Attempt to position the patient as consistently as possible for each imaging session: flatly supine, arms to the side, head and neck straight forward.
4. At the foot of the patient, visible to the camera but not touching the patient, place the known activity reference source of I-131. This source will be placed in the field of view for calibration purposes at each imaging session.
5. Acquire anterior/posterior whole body image of patient 15 minutes post injection, using parameters described in the table below.
6. Record any unusual image findings on the worksheet provided (i.e. hot injection site, image artifact, etc.)

Table 5

**Parameters for Patient Imaging**	**Description**	**Note**
I-131 calibration source	Place at foot of patient	Tape vial to bed to eliminate motion
Energy window	20% symmetric, peaked on 364 keV (328-400 keV)	
Scatter correction windows	20% symmetric, peaked on 298 keV and 436 keV	Triple Energy Window (TEW) method; applied during acquisition
Scan Speed	10 cm/min	Record scan speed in case report form; use consistent scan speed for all imaging sessions on Days 1-4.
Matrix	256 x 1024	
Attenuation Correction	NA	Will be applied post-processing at central lab
Collimator	Parallel hole, high energy	Ultra high energy collimators for 511 keV SPECT will not be used for this protocol
Views	Complete anterior and posterior	Include extremities; do not overlap arms with torso; scans to be centered on midline of body
Data to be recorded		Data worksheet will be provided

Day 0: 4-6 hours post injection

1. Have the patient void their bladder prior to imaging.
2. Acquire whole body planar images with calibration source using the parameters listed in Table 5.

Day 1: 18-24 hours post injection

1. Have the patient void their bladder prior to imaging.
2. Acquire the whole body planar images with calibration source using the parameters listed in Table 5.

Day 2: 48 hours ± 6 hours post injection

1. Have the patient void their bladder prior to imaging.

2. Acquire the whole body planar images with calibration source using the parameters listed in Table 5.

Day 3: 72 hours ± 6 hours post injection

1. Have the patient void their bladder prior to imaging.
2. Acquire the whole body planar images with calibration source using the parameters listed in Table 5.

Day 6: 144 hours ± 6 hours post injection

1. Have the patient void their bladder prior to imaging.
2. Acquire the whole body planar images with calibration source using the parameters listed in Table 5.

Day 14 ± 1 Day

1. Acquire the whole body planar images with calibration source using the parameters listed in Table 5.
2. Have the patient void their bladder prior to imaging.

Optional SPECT Imaging

At the Investigator's discretion, SPECT imaging may be performed between Days 3 and 14 to further characterize the patient's tumor(s).

Data Analysis

Dosimetry data analysis will be performed at a central laboratory. Instructions will be provided for preparing the nuclear medicine raw data and transmitting it to the core laboratory.

Cellectar Contact Info

For questions or clarification about nuclear medicine quality control, imaging or dosimetry procedures, contact Cellectar:

LisaAnn Trembath
608-630-4482 (cell)
ltrembath@cellectar.com

APPENDIX E: GLOSSARY OF ABBREVIATIONS

AE	adverse event
ASCO	American Society of Clinical Oncology
AST	aspartate aminotransferase
AUC	area under the concentration curve
BP	blood pressure
BSA	body surface area
CBC	complete blood count
cGy	centiGray
C_{max}	maximum plasma concentration
CRF	case report form
CT	Computed Tomography
CTC	Common Toxicity Criteria
CV	curriculum vitae
DBP	diastolic blood pressure
DCF	data clarification form
DHHS	Department of Health and Human Services
dL	deciliter(s)
DLT	dose-limiting toxicity
ECG	electrocardiogram
ECOG	Eastern Oncology Cooperative Group
FDA	Food and Drug Administration
g	gram(s)
Gy	gray
HIV	Human Immunodeficiency Virus
I-131	Iodine-131
ICF	informed consent form
ICH	International Conference on Harmonization
IND	investigational new drug
INR	International Normalized Ratio
IRB	Institutional Review Board
IV	intravenous
keV	kiloelectron volts
kg	kilogram(s)
LVEF	Left Ventricular Ejection Fraction

mCi	millicurie(s)
mg	milligram(s)
mL	milliliter(s)
MTD	maximum tolerated dose
m^2	meters squared
NCI	National Cancer Institute
OLINDA	Organ Level INternal Dose Assessment
PICC	peripherally inserted central catheter
PK	pharmacokinetics
PLE	phospholipid ether analog
q.d	every day
USP	U.S. Pharmacopeia
SAE	serious adverse event
S_aO_2	saturated oxygen
SBP	systolic blood pressure
SPECT	single photon emission computed tomography
SOPs	standard operating procedures
t.i.d	three times a day
V_d	volume of distribution
WMA	World Medical Association
